# Does Substrate Positioning Affect the Selectivity and Reactivity in the Hectochlorin Biosynthesis Halogenase?

**DOI:** 10.3389/fchem.2018.00513

**Published:** 2018-10-30

**Authors:** Amy Timmins, Nicholas J. Fowler, Jim Warwicker, Grit D. Straganz, Sam P. de Visser

**Affiliations:** ^1^The Manchester Institute of Biotechnology and School of Chemical Engineering and Analytical Science, University of Manchester, Manchester, United Kingdom; ^2^The Manchester Institute of Biotechnology and School of Chemistry, University of Manchester, Manchester, United Kingdom; ^3^Institute of Biochemistry, Graz University of Technology, Graz, Austria; ^4^Institute of Molecular Biosciences, Graz University, Graz, Austria

**Keywords:** nonheme iron, enzyme catalysis, reaction mechanism, QM/MM, density functional theory, halogenation, hydroxylation

## Abstract

In this work we present the first computational study on the hectochlorin biosynthesis enzyme HctB, which is a unique three-domain halogenase that activates non-amino acid moieties tethered to an acyl-carrier, and as such may have biotechnological relevance beyond other halogenases. We use a combination of small cluster models and full enzyme structures calculated with quantum mechanics/molecular mechanics methods. Our work reveals that the reaction is initiated with a rate-determining hydrogen atom abstraction from substrate by an iron (IV)-oxo species, which creates an iron (III)-hydroxo intermediate. In a subsequent step the reaction can bifurcate to either halogenation or hydroxylation of substrate, but substrate binding and positioning drives the reaction to optimal substrate halogenation. Furthermore, several key residues in the protein have been identified for their involvement in charge-dipole interactions and induced electric field effects. In particular, two charged second coordination sphere amino acid residues (Glu_223_ and Arg_245_) appear to influence the charge density on the Cl ligand and push the mechanism toward halogenation. Our studies, therefore, conclude that nonheme iron halogenases have a chemical structure that induces an electric field on the active site that affects the halide and iron charge distributions and enable efficient halogenation. As such, HctB is intricately designed for a substrate halogenation and operates distinctly different from other nonheme iron halogenases.

## Introduction

Enzymatic C–Cl bond formation is a rare process in Nature, yet over the past few decades a range of haloperoxidases and halogenases have been discovered (Gribble, 2003; Vaillancourt et al., 2006; van Pée et al., 2006; Butler and Sandy, 2009; Wagner et al., 2009; Weichold et al., 2016; Agarwal et al., 2017; Schnepel and Sewald, 2017; Timmins and de Visser, 2018). Their catalytic mechanism, however, is still subject to controversies and understanding the fundamental details of these processes may have an impact on biotechnological advances as well as drug development. Three different classes of halogenation enzymes appear in Nature, namely the heme haloperoxidases, the vanadium-dependent nonheme haloperoxidases and the α-ketoglutarate dependent nonheme iron halogenases (Gribble, 2003; Vaillancourt et al., 2006; van Pée et al., 2006; Butler and Sandy, 2009; Wagner et al., 2009; Weichold et al., 2016; Agarwal et al., 2017; Schnepel and Sewald, 2017; Timmins and de Visser, 2018).

Heme haloperoxidases, (Sundaramoorthy et al., 1995; Wagenknecht and Woggon, 1997; Green et al., 2004; Kim et al., 2006) such as chloroperoxidase bind H_2_O_2_ on an iron(III)-heme center, which is then converted into an iron(IV)-oxo heme cation radical species called Compound I, (Meunier et al., 2004; Denisov et al., 2005; Shaik et al., 2005; Rittle and Green, 2010) with the help of a proton shuttle machinery. Compound I subsequently reacts with chloride to form OCl^−^ products. The product drifts out of the active site and reacts with substrates through halogenation. A second class of haloperoxidases has a nonheme vanadium co-factor that also utilizes hydrogen peroxide and halide in a catalytic cycle to form OCl^−^ products (Messerschmidt and Wever, 1996; Chen and van Pée, 2008). The vanadium haloperoxidases have been characterized in marine algae and are believed to have functions related to natural product synthesis associated with defense mechanisms (Martinez et al., 2001).

The final class of halogenases are the α-ketoglutarate (αKG) dependent nonheme iron halogenases, (Blasiak et al., 2006; Buongiorno and Straganz, 2013; Huang and Groves, 2017) which show structural and functional similarities to the corresponding nonheme iron—α-ketoglutarate-dependent dioxygenases (Solomon et al., 2000; Bugg, 2001; Ryle and Hausinger, 2002; Costas et al., 2004; Abu-Omar et al., 2005; Bruijnincx et al., 2008). These nonheme iron halogenases contain an iron(II) center in the resting state that is bound to the side chains of two histidine residues. During the catalytic cycle, halide and αKG bind to the iron(II) ion prior to molecular oxygen binding. It is believed dioxygen attacks αKG to form succinate and a high-valent iron(IV)-oxo species similarly to the αKG-dependent hydroxylases (Schofield and Zhang, 1999; Bollinger et al., 2005). The iron(IV)-oxo species of several nonheme iron halogenases have been spectroscopically trapped and characterized and shown to react with substrates with a rate-determining hydrogen atom abstraction barrier (Galonić Fujimori et al., 2007; Neidig et al., 2007; Matthews et al., 2009; Wong et al., 2013; Srnec et al., 2016; Srnec and Solomon, 2017). The subsequent pathway leading to the halogenated product; however, is controversial as a thermodynamically much more favorable hydroxylation pathway is prevented. How the enzyme manages to perform this unfavorable thermodynamic reaction pathway is under much debate. One proposal suggested links to substrate binding and orientation (Matthews et al., 2006). Thus, in the antibiotic biosynthesis protein SyrB2 the substrate L-Thr is linked to an acyl-carrier protein (SyrB1) via a phosphopantetheinyl (PPT) bridge. Replacement of L-Thr by L-norvaline changed the chemoselectivity of substrate halogenation to hydroxylation due to substrate positioning in the active site (Matthews et al., 2006). On the other hand, computational modeling of the Borowski group proposed a rotation step in the iron(IV)-oxo(halide) group, whereby after hydrogen atom abstraction, the positions of the hydroxo and halide ions switched position leading to easier halide rebound (Borowski et al., 2010). Clearly, the mechanism of aliphatic halogenation remains controversial. Because of this, synthetic (biomimetic) model complexes of these nonheme iron halogenases and haloperoxidases have been developed and studied with experimental (Podgoršek et al., 2009; Comba and Wunderlich, 2010; Liu and Groves, 2010; Chatterjee and Paine, 2016; Puri et al., 2016; Wang et al., 2016) and computational approaches (Noack and Siegbahn, 2007; de Visser and Latifi, 2009; Kulik et al., 2009; Pandian et al., 2009; Quesne and de Visser, 2012; Senn, 2014; Huang et al., 2016; Timmins et al., 2018). Thus, if the halogenation pathway is dependent on substrate positioning then biomimetic models that lack the protein and do not bind substrates in fixed orientations may not be able to react via substrate halogenation efficiently.

A recently discovered halogenase (HctB) from *Lyngbya majuscula* is involved in the biosynthesis of hectochlorin, whereby a fatty acyl substrate is dihalogenated on a nonheme iron center (Ramaswamy et al., 2007; Pratter et al., 2014a). In contrast to the nonheme iron halogenase SyrB2, HctB activates a non-amino acid group as a substrate. This would give HctB biotechnological applicability that is beyond that of halogenases like SyrB2. As such, HctB appears to have certain flexibility in the activation of an alkyl chain; therefore, we decided to investigate its mechanism using computational methods and compare the mechanism and active site features with those described for SyrB2 previously. The mechanism of O_2_ activation for this mononuclear nonheme iron halogenase was characterized with stopped-flow and spectroscopic (circular dichroism and magnetic circular dichroism) studies (Pratter et al., 2014b).

**Figure 1:**
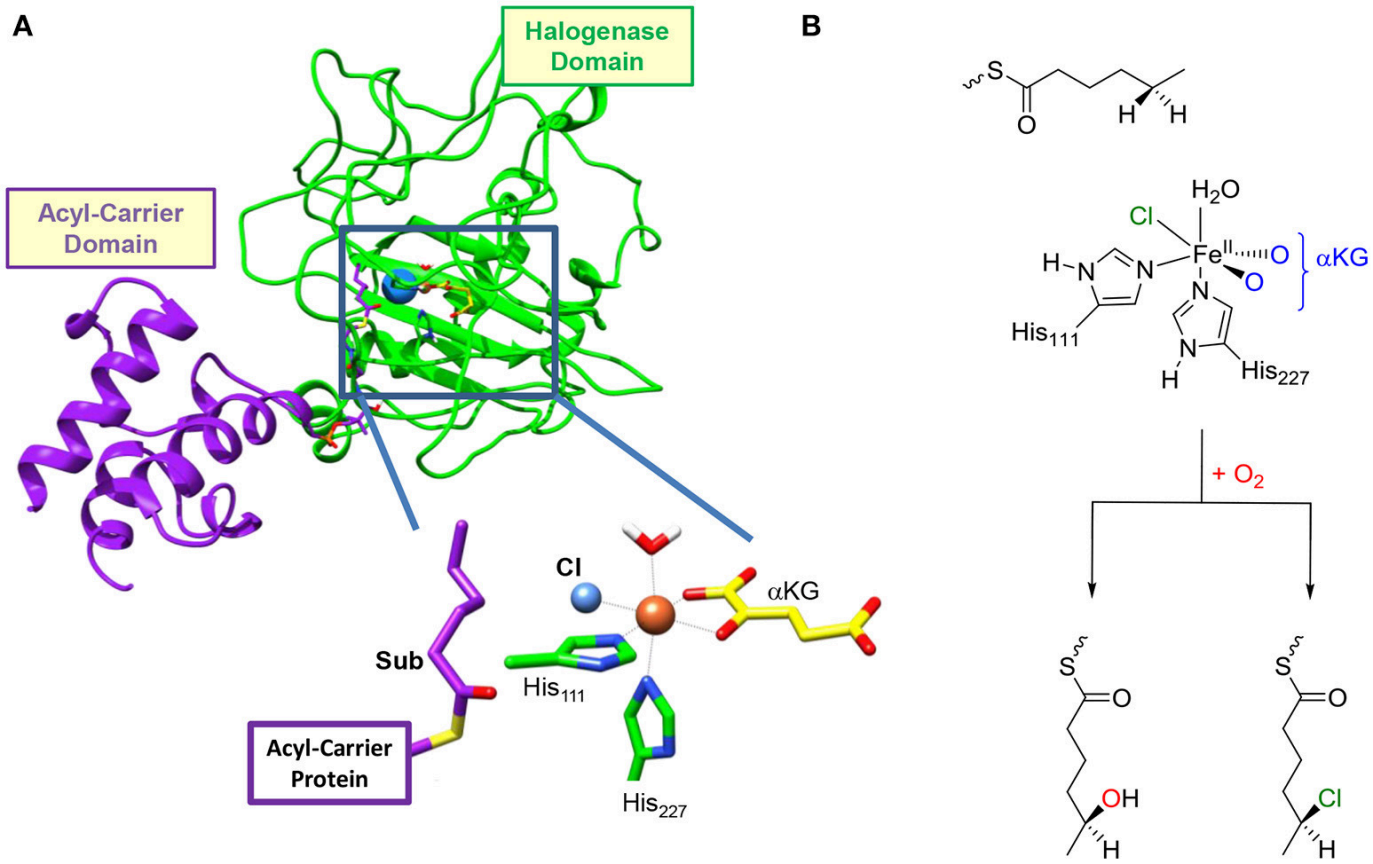
**(A)** Structure of the resting state of HctB as taken from Pratter et al. (2014b). The different domains of the secondary structure are identified and a close-up of the active site structure is given. **(B)** General reaction mechanism of the first halogenation step in HctB investigated here.

Functional characterization of the enzyme together with analysis of its primary structure reveals that it is a unique three-domain halogenase containing an acyl carrier protein (ACP), which binds the substrate covalently via a phosphopantetheinyl bridge (Figure 1). The other terminus of the ACP group is connected to the C-terminus of an acyl-Coenzyme-A subunit, while the N-terminus is linked to the halogenase domain. As such the protein has an intricate set-up and arrangement that enables dihalogenation of the tethered substrate. Interestingly, no evidence of substrate hydroxylation is available, but the activation of hexanoic acid by HctB apart from dihalogenation also gave products from oxygenation leading to vinyl-chloride and ketone products (Pratter et al., 2014a). HctB shows a certain degree of sequence similarity with SyrB2 (see Supporting Information for an overlay and sequence alignment), (Pratter et al., 2014a) and binds iron in a nonheme ligand configuration and utilizes α-ketoglutarate (αKG). The metal in HctB is linked to the protein via two histidine groups (His_111_ and His_227_) in a pentacoordinate environment. However, there are major differences in the overall structure of SyrB2 vs. HctB, which are currently understood and warrant a detailed computational study.

After αKG and halide binding, the metal binds molecular oxygen, and it has been hypothesized to react with αKG to form an iron(IV)-oxo species and succinate upon release of CO_2_. Unfortunately, the iron(IV)-oxo species in HctB has never been trapped and characterized and details of the halogenation mechanism are unknown. In the next stage of the catalytic cycle it is expected that a hydrogen atom abstraction occurs to form an iron(III)-hydroxo species, which is elusive as well. Technically, however, the halide should rebound to the substrate radical to form halogenated products, but thermodynamically it has been shown that hydroxo rebound is energetically favorable (Timmins and de Visser, 2015). How the enzyme avoids the low-energy hydroxylation pathway in favor of the higher-energy halogenation remains a matter of discussion. In order to understand the effect of substrate binding on the chemoselectivity of substrate halogenation vs. hydroxylation in HctB we employed a detailed molecular mechanics (MM) and quantum mechanics/molecular mechanics (QM/MM) study. We located two substrate entrance channels and have calculated the substrate halogenation and hydroxylation pathways with a substrate in these positions. The work shows that dramatic differences in halogenation vs. hydroxylation product ratios should be expected based upon substrate positioning. Moreover, our calculations predict that the protein induces an electric field effect that withdraws electron density from the halide toward the metal during the reaction in order to make the halogenation process favorable.

## Results

### Model selection and reactant benchmarking

So far, no computational modeling studies have been reported on HctB and little is known how different (if at all) it is to the well-studied SyrB2. Obviously, both nonheme iron halogenases utilize a different substrate although both are tethered to a carrier protein. Conversely, SyrB2 catalyzes a monohalogenation of the substrate, whereas HctB performs a dihalogenation instead. A comparison of the amino acid sequences of HctB vs. SyrB2 shows major deviations which must result in differences in secondary structure. Therefore, their mechanisms may be quite different and we decided to do a computational study on the mechanism of halogenation vs. hydroxylation of HctB and how it compares with previous experimental and computational studies of analogous nonheme iron halogenases. In particular, we focused our computational study on the first halogenation step of the fatty acyl tethered substrate of a HctB model and took the structure reported by Pratter et al. (2014a). Our set-up of QM/MM models was reviewed thoroughly recently; therefore, we will summarize the main issues only briefly (Hernández-Ortega et al., 2015; Quesne et al., 2016a; Hofer and de Visser, 2018). The model was altered from an iron(II)-water α-ketoglutarate bound structure into an iron(IV)-oxo with ligated succinate. Prior to the full set-up of the complete QM/MM chemical system, however, we investigated possible substrate binding positions of the tethered hexanoyl-PPT moiety. In particular, we searched for alternative substrate entrance channels into the active site.

**Figure 2:**
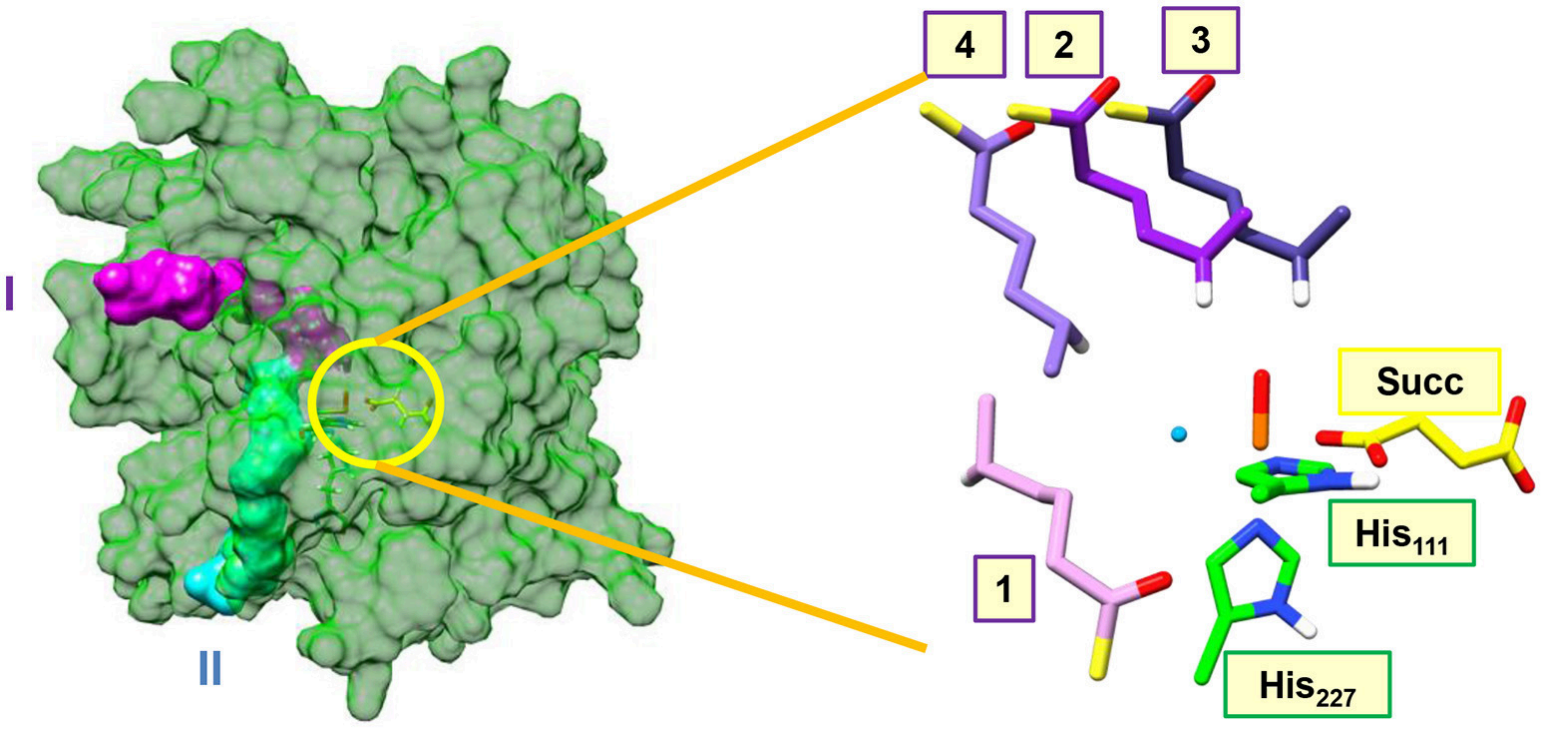
Substrate entrance channels I (in purple) and II (in cyan) into the active site of the halogenase domain of HctB. The yellow circle highlights the active site of the halogenase in the protein. The right-hand-side of the figure gives an overlay of the four substrate binding positions, where we only show the hexanoyl group and highlight the transferring hydrogen atom in white. Substrate binding position 1 is located in entrance channel II and substrate binding position 2, 3, and 4 are bound in entrance channel I.

Figure 2 shows highlights of the two substrate entrance channels we identified, which are narrow channels that should fit the linear terminal chain of the substrate-carrier protein. The acyl-carrier group of the substrate protein then latches onto the surface of the protein and inserts the tethered hexanoyl-PPT group into the active site. Thus, substrate entrance channel II represents the model from Pratter et al. (2014a) with substrate position **1**. In model **1** with the substrate entering through channel II, the substrate is located parallel to the iron(IV)-oxo group in a position very similar to the methylated DNA strand in the AlkB repair enzyme (Quesne et al., 2014). The substrate approaches the iron(IV)-oxo group in between the Val_113_ and Glu_223_ side chains in the corner where the halide group is also located. The entrance channel is located well below the iron(IV)-oxo group.

During analysis of the structure, we identified another entrance channel and attempted to latch the acyl carrier protein onto this and manually inserted the tethered hexanoyl-PPT chain. In the analogous halogenase SyrB2 a similar substrate entrance channel is seen in the same position, (Matthews et al., 2006) but due to the lack of a substrate bound crystal structure of HctB we will consider all possible substrate orientations and entrance channels. Entrance channel I is located above the iron(IV)-oxo group and inserts the tethered hexanoyl group into a large open space (possibly filled with water molecules) and hence we created three starting orientations for the substrate, namely models **2**, **3**, and **4**. In model **2** the terminus of the tethered hexanoyl group points down and hangs in between the Val_113_ and Arg_245_ residues, whereas in model **3** it is found in between Lys_126_ and Pro_127_. The final substrate position (**4**) through channel I is located on the side of the iron(IV)-oxo group and also brings the terminus of the tethered hexanoyl group into the active site nearby the Val_113_ and Glu_223_ residues. The substrate binding orientation through channel I model **4** brings it in close proximity to both the oxo and halide groups. These three substrate binding positions are distinctly different and guided by hydrogen bonding interactions of the thioester moiety of the hexanoyl-PPT moiety with amino acid residues aligning the channel walls. As such we do not expect easy interconversion between the three substrate binding models.

Subsequently, we investigated the reaction mechanism from each of these different substrate starting orientations and set up QM/MM models with the substrate bound in positions **1**, **2**, **3** and **4**. The set-up of the QM/MM models follows previously reported and benchmarked methods (Quesne et al., 2014, 2016a; Hernández-Ortega et al., 2015; Hofer and de Visser, 2018). Firstly, we added hydrogen atoms using known pK_a_ values and visually inspected polar residues for correct protonation states. Thereafter, we applied an iterative solvation procedure and followed this with an equilibration and heating run to a temperature of 298 K. During these set-up steps the protein backbone was fixed, but in the final molecular dynamics simulation all atoms were allowed to move. We initially ran MD simulations for a period of up to 200 ns; however, the run stabilized in all cases after about 5 ns. Clearly, HctB is a very rigid protein with substrate and active site in tight binding orientation with little flexibility as seen from the MD runs. Therefore, for all subsequent systems described here only a 10 ns MD simulation was taken, see Supporting Information Figure S1. From the MD simulations we selected a low energy snapshot after 5 ns as starting structures for the actual QM/MM calculations.

**Figure 3:**
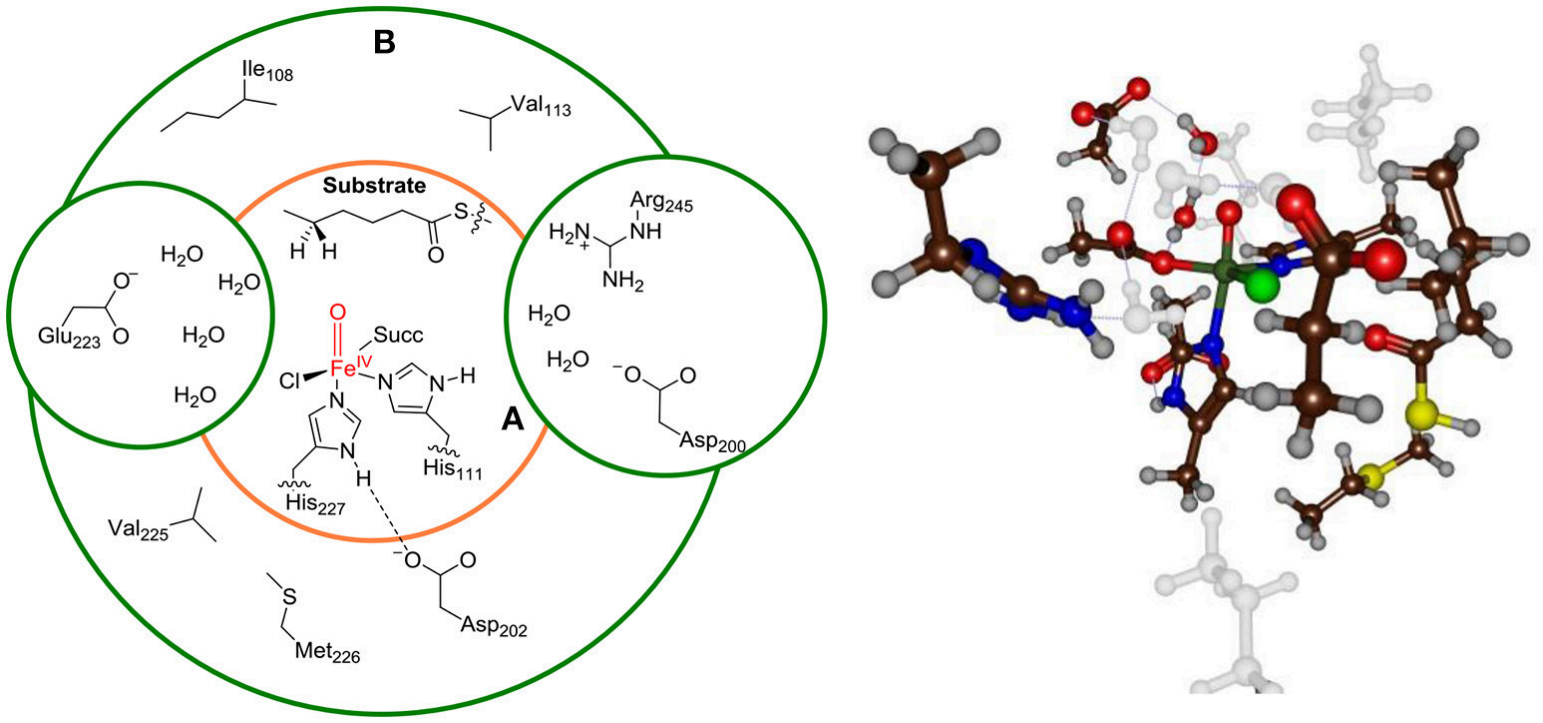
Description of QM region A and AB for Model **1**. Ball-and-stick model of QM region AB is shown on the right-hand-side.

Finally, we bisected the full chemical structure of protein, substrate and water layer into a QM and MM region and included key residues in the QM region that form covalent or hydrogen bonding interactions with substrate and oxidant. Initial, exploratory calculations were done with a small QM region containing only the iron(IV)-oxo(chloro) group and the first-coordination sphere of ligands to the metal, namely the imidazole groups of His_111_ and His_227_, the acetate terminus of succinate (Succ) and the thiohexanoic acid arm of the substrate as our minimal QM region **A**, see Figure 3. This model contains 48 QM atoms and is overall charge neutral. To test the effect of the second coordination sphere we also calculated the full mechanism with a larger QM region that in addition to QM region **A** included the amino acid side chains of residues within 6 Å of the iron(IV)-oxo-chloro structure, i.e. QM region **AB**. For model **1**, the large QM region **AB** contained the amino acid side chains of Ile_108_, Val_113_, Asp_200_, Asp_202_, Glu_223_, Val_225_, Met_226_, Arg_245_, and six water molecules and has a total of 160 atoms in the QM region.

Thereafter, QM/MM geometry optimizations of the iron(IV)-oxo(chloro) species (**Re**) were performed in the singlet, triplet and quintet spin states. Note that in the label of the structure we give the spin multiplicity in superscript before the label and the substrate binding position and the QM region (**A** or **AB**) in subscript after the label. The full set of results that were obtained is given in the Supporting Information, while we focus in the main text on the low-energy pathways only.

We started the work with extensive validation and benchmarking of the methods. Unfortunately, there are no experimental rate constants and spectroscopic data for the iron(IV)-oxo species. Previously, we calibrated thioanisole sulfoxidation free energies of activation of a biomimetic nonheme iron(IV)-oxo complex against experimental data and tested 50 different computational methods and techniques (Cantú Reinhard et al., 2016a). The best agreement with experiment was found for the PBE0/BS2 and B3LYP/BS2 methods with a solvent model included. In particular, free energies of activation were reproduced within 4 kcal mol^−1^ from experiment (Vardhaman et al., 2011, 2013; de Visser et al., 2014; Sainna et al., 2015; Barman et al., 2016a; Cantú Reinhard et al., 2016b). Furthermore, the methods reproduced experimental product distributions of bifurcation processes well (Ji et al., 2015; Kaczmarek et al., 2018). Finally, for the nonheme iron dioxygenase prolyl-4-hydroxylase six hydrogen atom abstraction barriers from substrate were investigated and the QM/MM predicted the correct regioselectivity and therefore the methods are expected to predict regio- and chemoselectivities well (Karamzadeh et al., 2010; Pratter et al., 2013; Timmins and de Visser, 2017; Timmins et al., 2017).

In agreement with the experimental studies on the iron(IV)-oxo species of the halogenase SyrB2, (Galonić Fujimori et al., 2007) we find the quintet spin state as the ground state for all chemical systems. Interestingly, the quintet spin state is below the triplet spin state by a considerable margin of well over 13 kcal mol^−1^; hence the triplet and singlet spin states of HctB will not play a major role during the reaction and are high in energy. Therefore, we will focus on the quintet spin state structures and mechanism only here. The Supporting Information gives all results of the alternative spin states investigated. Nevertheless, the reaction is expected to proceed through single-state-reactivity on the dominant quintet spin state surface in agreement with previous studies on pentacoordinated iron(IV)-oxo complexes reported before for nonheme iron enzymatic and model complexes (de Visser, 2006a,b, 2010; Hirao et al., 2006; Bernasconi and Baerends, 2008; Latifi et al., 2009; Ye and Neese, 2011; Ansari et al., 2013; Tang et al., 2013; Saouma and Mayer, 2014; Cantú Reinhard and de Visser, 2017a).

The quintet spin state of the iron(IV)-oxo(chloro) species in HctB has four unpaired electrons located in the $\pi_{\text{xy}}^{*}$, $\pi_{\text{xz}}^{*}$, $\pi_{\text{yz},}^{*}$ and $\sigma_{\text{x}2-\text{y}2}^{*}$ molecular orbitals and a virtual $\sigma_{\text{z}2}^{*}$ orbital. Thus, the $\pi_{\text{xz}}^{*}$ and $\pi_{\text{yz}}^{*}$ orbitals represent the antibonding interactions of the metal 3d_xz_/3d_yz_ atomic orbitals with a 2p_x_/2p_y_ orbital on the oxo group. These two orbitals are orthogonal and close in degeneracy in nonheme iron(IV)-oxo and located in the xz and yz molecular planes, whereby the z-axis is defined by the Fe–O axis. The $\pi_{\text{xy}}^{*}$ and $\sigma_{\text{x}2-\text{y}2}^{*}$ orbitals are both in the plane perpendicular to the Fe–O axis (xy-plane) and represent antibonding orbitals with ligands in the equatorial plane, namely the nitrogen of His_111_, the carboxylate of succinate and the halide atom. The final metal-type 3d molecular orbital is the $\sigma_{\text{z}2}^{*}$ orbital for the σ-type antibonding interaction along the Fe–O bond, which is virtual.

**Figure 4:**
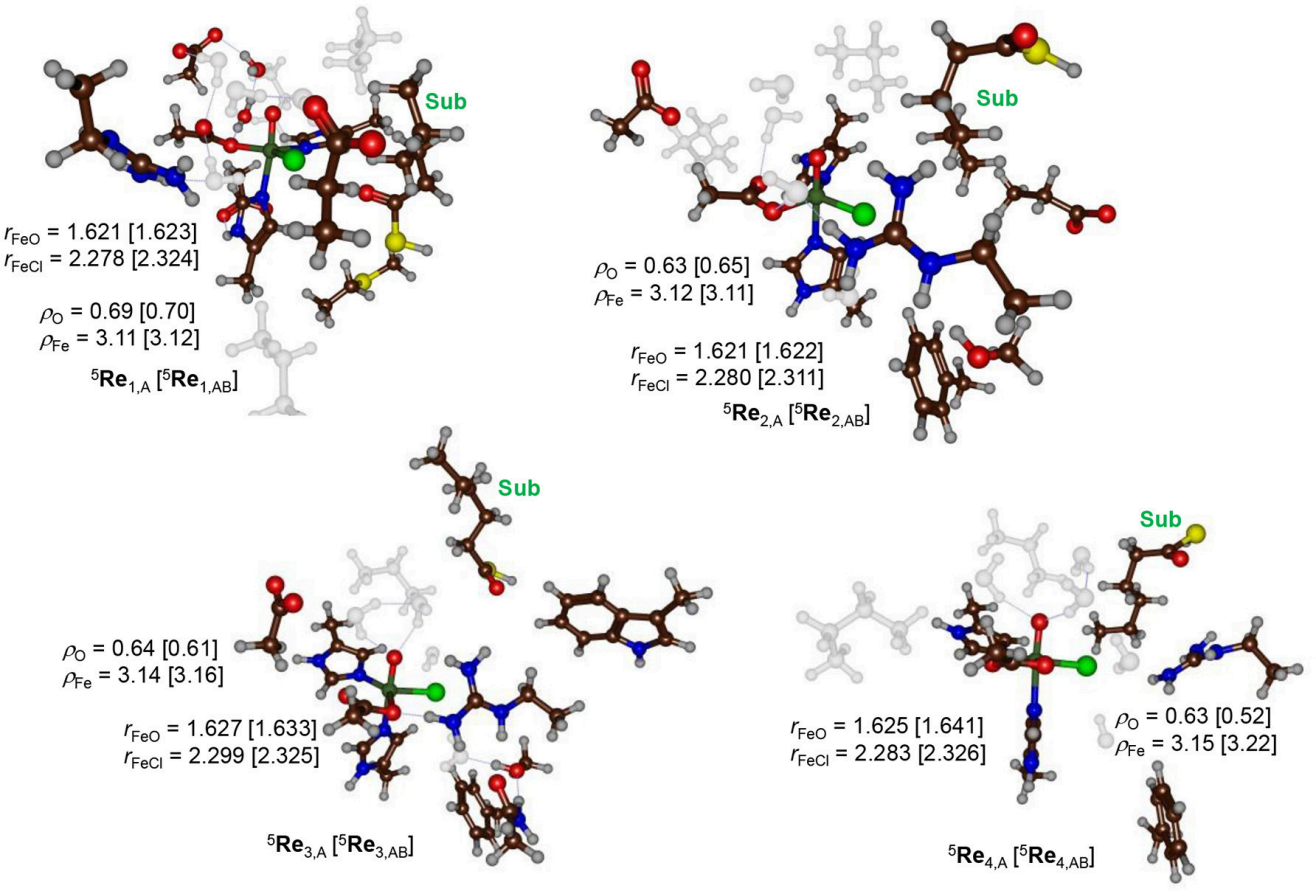
QM/MM optimized geometries of the quintet spin iron(IV)-oxo reactant complexes as calculated at UB3LYP/BS1 level of theory in Turbomole:Charmm. Structures were optimized with either QM region A or QM region AB. Reactant structures for models **1–4** were calculated and are grouped by model: top row model **1** and model **2**, bottom row model **3** and **4**. Bond lengths are given in angstroms. Water molecules and aliphatic residues are shaded.

During the equilibration and MD simulation as well as the subsequent QM/MM geometry optimization the positions of the hexanoyl substrate chains have moved slightly. In particular this was the case for model **3** where the terminus moved away from the direction of the iron(IV)-oxo(chloro) group. As a result, the distance between substrate and iron(IV)-oxo(chloro) is quite large (>5 Å) in model **3**. Figure 4 shows the eight different optimized geometries of the iron(IV)-oxo(chloro) reactant species as calculated with QM/MM for models **1**, **2**, **3** and **4** with either QM region **A** or **AB**. As can be seen, all optimized structures, regardless of the substrate binding position, give very similar optimized geometries with Fe–O distances ranging from 1.621 to 1.641 Å and Fe–Cl distances between 2.278 and 2.326 Å. These values match previous calculations on nonheme iron(IV)-oxo(chloro) and nonheme iron(IV)-oxo complexes excellently (de Visser, 2006a,b, 2010; Hirao et al., 2006, 2011; Matthews et al., 2006; Noack and Siegbahn, 2007; Bernasconi and Baerends, 2008, 2013; de Visser and Latifi, 2009; Kulik et al., 2009; Latifi et al., 2009; Pandian et al., 2009; Dey, 2010; Ye and Neese, 2011; Quesne and de Visser, 2012; Ansari et al., 2013; Liu et al., 2013; Tang et al., 2013; Usharani et al., 2013; Kumar et al., 2014; Saouma and Mayer, 2014; Senn, 2014; Huang et al., 2016; Zhao et al., 2016; Cantú Reinhard and de Visser, 2017a; Timmins et al., 2018).

The QM/MM results from Figure 4 show that the secondary environment and the substrate positioning have little or no effect on the Fe–O and Fe–Cl distances in the iron(IV)-oxo(chloro) species although it may affect the kinetics as shown below. In addition, no major electronic differences are seen for the eight optimized structures and all converge to a quintet spin ground state. To be specific, group spin densities of all structures are close with values of around ρ_Fe_ = 3.12 and ρ_O_ = 0.63. The orbital analysis and group spin densities implicate that most spin density is located along the Fe–O bond and point to an orbital occupation of ${\pi^{*}}_{\text{xy}}^{1}$ ${\pi^{*}}_{\text{xz}}^{1}$ ${\pi^{*}}_{\text{yz}}^{1}$ ${\sigma^{*}}_{\text{x}2-\text{y}2}^{1}$ for all optimized geometries. Our calculations are in agreement with experimental EPR and Mössbauer spectroscopy studies on the analogous structure in SyrB2 that also reported a high-spin ground state (Galonić Fujimori et al., 2007).

The only noticeable difference between the four substrate binding positions relates to the orientation of the active site Arg_245_ residue, which is locked in hydrogen bonding interactions to the dangling terminal carboxylate group of succinate via a bridging water molecule in model **1** and **2**. By contrast, in model **3** the Arg_245_ group forms a direct salt bridge with the carboxylate of the succinate moiety and also is in close proximity to the oxo group. Finally, in structure **4**, the Arg_245_ forms a salt bridge with Glu_223_, whereby it appears to close entrance channel II. It may very well be, therefore, that the Arg_245_ residue is involved in α-ketoglutarate binding and/or succinate release from the active site. Indeed, Arg_245_ is a conserved residue in most reported nonheme iron halogenases and therefore, is expected to play a key role in catalysis and/or substrate positioning. On the other hand, Glu_223_ is not conserved in the majority of reported nonheme iron halogenases and only found in HctB. It would be interesting to see how mutation of either Glu_223_ or Arg_245_ affects the enzyme function, chemical catalysis and product distributions. But that will have to await a future experimental study.

### Chemoselectivity patterns

**Scheme 1:**
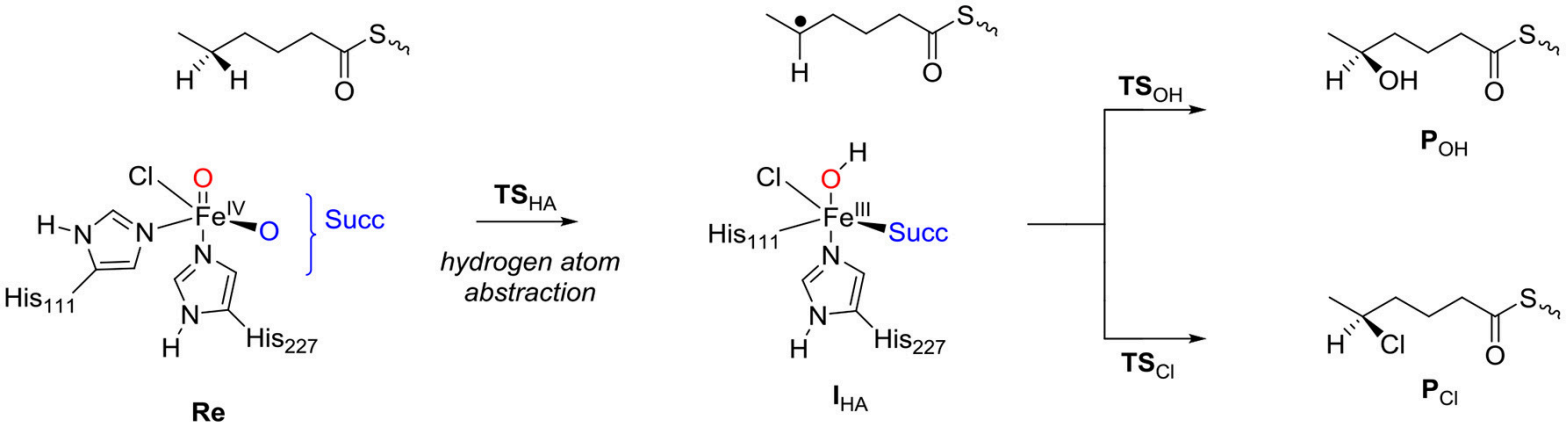
Reaction mechanism investigated in this work.

Next, the chemoselectivity of substrate halogenation vs. hydroxylation was investigated for the pathways described and defined as in Scheme 1. The reaction starts from the reactant complex of iron(IV)-oxo(chloro) with substrate (**Re**) and proceeds via a stepwise mechanism with an initial hydrogen atom abstraction transition state (**TS**_HA_) leading to an iron(III)-hydroxo complex and a substrate radical (**I**_HA_). In the next step the pathways diverge and either OH rebound (via transition state **TS**_OH_) or Cl rebound (via transition state **TS**_Cl_) occurs. These mechanisms then lead to the hydroxylated products (**P**_OH_) and halogenated products (**P**_Cl_), respectively. Technically, the reaction apart from being chemoselective is also enantioselective where only one of the two isomers is expected. Thus, due to tight substrate binding and positioning, the hydrogen atom abstraction will be selective from only one of the two hydrogen atoms from the ω-1 position of the substrate. Hence the hydrogen atom abstraction will guide the enantioselectivity as seen before on related enzymes (Karamzadeh et al., 2010; Pratter et al., 2013; Timmins and de Visser, 2017; Timmins et al., 2017). Nevertheless, in HctB the first halogenation of substrate is followed by a second halogen transfer through the binding of another molecule of O_2_ and Cl^−^ to repeat the cycle and the formation of the dihalogenated product.

We tested the mechanisms for the first halogenation step in HctB on all low lying spin states (singlet, triplet, quintet), for each of the substrate binding positions (**1**, **2**, **3**, **4**) and with different QM regions (**A** or **AB**). Previously, we found the septet spin state of nonheme iron(IV)-oxo to be at least 7 kcal mol^−1^ higher in energy than the quintet spin state and, therefore, we did not investigate this state further (Latifi et al., 2009). However, we tested the models and methods by using different optimization techniques (basis set, DFT method, snapshot).

Firstly, changing the basis set from SV(P) to TV(P) on all atoms had little effect on the optimized geometries and lowered the hydrogen atom abstraction barrier by only a small amount. In particular, geometry optimizations at UB3LYP/BS1 and UB3LYP/BS2 of ^5^**Re**_1_ and ^5^**TS**_HA, 1_ using model **1** and the large QM region **AB** gave hydrogen atom abstraction barriers of 23.5 kcal mol^−1^ with UB3LYP/BS2 and 21.5 kcal mol^−1^ with UB3LYP/BS2//UB3LYP/BS1. In addition, the optimized geometries obtained with UB3LYP/BS1 vs. UB3LYP/BS2 are very similar (vide infra). Therefore, we continued with geometry optimizations at UB3LYP/BS1 level of theory for the remainder of the project.

Secondly, choosing alternative snapshots from the MD simulation also had little effect on the optimized geometries of the reactant complexes of nonheme iron complexes and did not change spin state orderings and relative energies dramatically. This probably is as nonheme iron dioxygenases often have very rigid structures where the substrate is tightly bound (Aluri and de Visser, 2007; Kumar et al., 2011; Tchesnokov et al., 2016; Faponle et al., 2017). Previous QM/MM studies on analogous nonheme iron dioxygenases indeed reproduced experimental chemoselectivities excellently using the same methods and procedures described here (Aluri and de Visser, 2007; Karamzadeh et al., 2010; Kumar et al., 2011; Pratter et al., 2013; Quesne et al., 2014, 2016a; Hernández-Ortega et al., 2015; Tchesnokov et al., 2016; Faponle et al., 2017; Timmins and de Visser, 2017; Timmins et al., 2017; Hofer and de Visser, 2018). Moreover, changing the QM region, basis set or density functional method did not change the ordering of the transition states.

**Figure 5:**
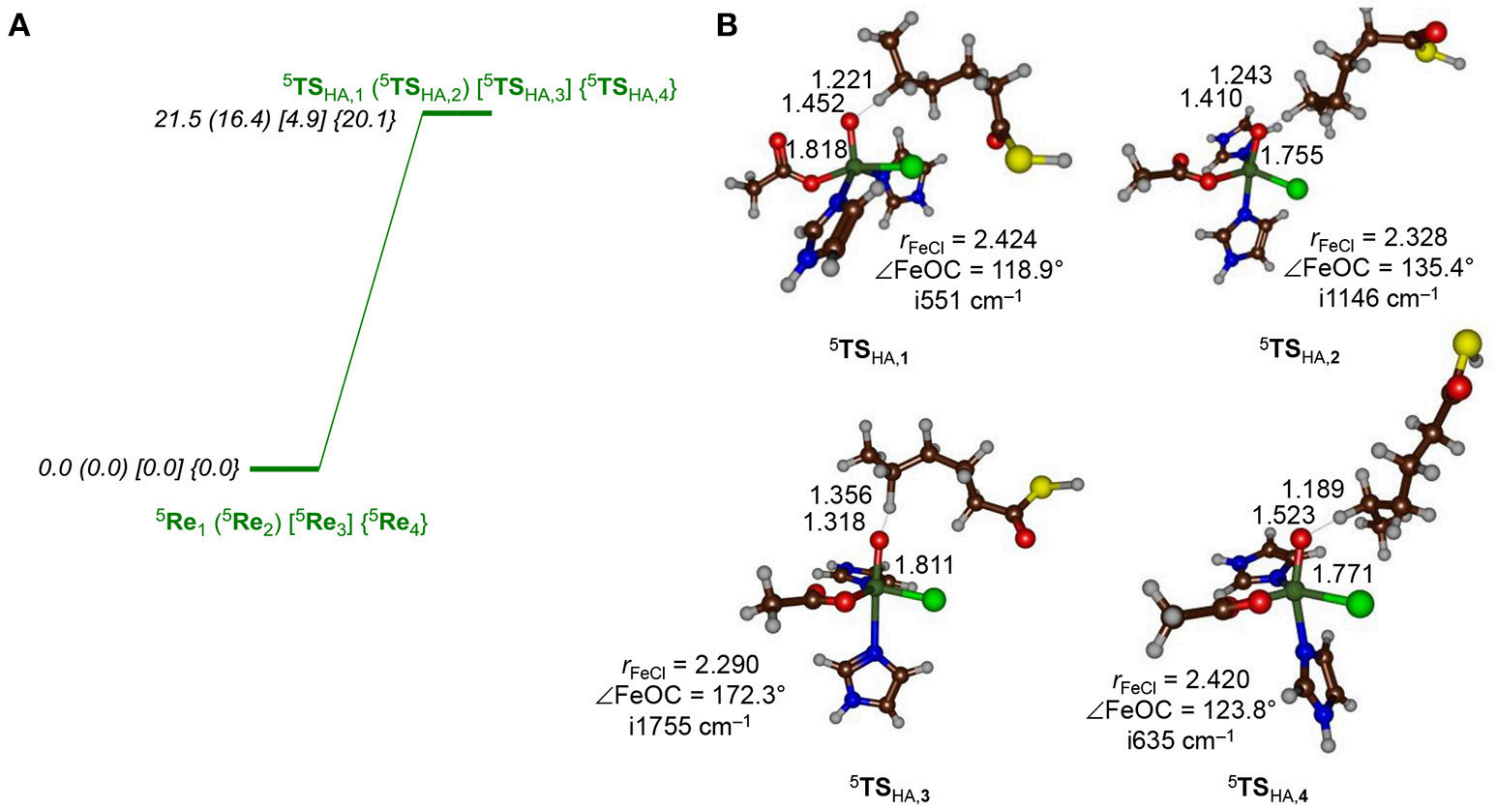
**(A)** Hydrogen atom abstraction barriers (structures and energies, in kcal mol^−1^) for hydrogen atom abstraction from the C5 position of the hexanoyl substrate terminus by the iron(IV)-oxo(chloro) species of HctB as calculated with QM/MM at UB3LYP/BS2/ /UB3LYP/BS1:Charmm with Turbomole using QM region AB. **(B)** Optimized geometries of the hydrogen atom abstraction barriers with bond lengths in angstroms, the Fe–O–C^5^ angle in degrees and the imaginary frequency in wave numbers.

Let us first focus on the hydrogen atom abstraction step, which is rate-determining in the overall reaction mechanism and optimized geometries for all four substrate binding positions are shown in Figure 5. Hydrogen atom abstraction from a quintet spin iron(IV)-oxo species with configuration ${\pi^{*}}_{\text{xy}}^{1}$ ${\pi^{*}}_{\text{xz}}^{1}$ ${\pi^{*}}_{\text{yz}}^{1}$ ${\sigma^{*}}_{\text{x}2-\text{y}2}^{1}$ leads to a sextet spin iron(III)-hydroxo with a nearby radical, or alternatively a quartet spin iron(III)-hydroxo with a nearby radical. Thus, the abstracted electron from the hydrogen atom either pairs up with the lowest ${\pi^{*}}_{\text{xy}}$ electron to form a quartet spin iron(III)-hydroxo species or enters the virtual ${\sigma^{*}}_{\text{z}2}$ orbital to give a sextet spin iron(III)-hydroxo species. Previously, (de Visser, 2006b; Bernasconi and Baerends, 2008; Ye and Neese, 2011; Ansari et al., 2013; Tang et al., 2013; Cantú Reinhard and de Visser, 2017a) it was shown that hydrogen atom abstraction by a quintet spin pentacoordinated nonheme iron(IV)-oxo species leads to electron transfer to the metal that preferentially fills the $\sigma_{\text{z}2}^{*}$ orbital with one electron, designated the ^5^σ-pathway. Alternatively, the electron can move into one of the lower-lying and singly occupied π^*^ orbitals: the so-called ^5^π-pathway. A spin density and molecular orbital analysis for all hydrogen atom abstraction transition states revealed that all are of ^5^σ-type leading to a radical intermediate with five unpaired electrons in metal-type orbitals (${\pi^{*}}_{\text{xy}}^{1}$ ${\pi^{*}}_{\text{xz}}^{1}$ ${\pi^{*}}_{\text{yz}}^{1}$ ${\sigma^{*}}_{\text{z}2}^{1}$ ${\sigma^{*}}_{\text{x}2-\text{y}2}^{1}$) and a down-spin electron on the substrate. Thus, in all transition states there is negative spin density on the substrate (ranging from −0.27 to −0.47) while the spin density on iron increases to 4.01–4.14. These values are in agreement with hydrogen atom abstraction reactions calculated before for nonheme iron dioxygenases and synthetic model complexes (Karamzadeh et al., 2010; Hirao et al., 2011; Pratter et al., 2013; Kumar et al., 2014; Zhao et al., 2016; Timmins and de Visser, 2017; Timmins et al., 2017). We attempted to find a transition state for the ^5^π-pathway for several models, but found them considerably higher in energy and we did not manage to optimize those structures with QM/MM. Filling of the $\sigma_{\text{z}2}^{*}$ orbital with one electron, as happens in the ^5^σ-pathway, usually means that the hydrogen atom abstraction is performed along the molecular z-axis, i.e., a linear Fe–O–C angle is seen in the transition state. However, due to constraints in substrate position and orientation and the location of amino acid residues in that region, in the protein the substrates are unable to approach the metal-oxo under this “ideal” angle. As a matter of fact, only in model **3** the substrate can approach the iron(IV)-oxo(chloro) from the top and an almost linear Fe–O–C^5^ angle is found, while the angle is considerably less for the other structures. As a result, the barrier is the lowest for model **3**. Nevertheless, even with the transition states in the bent orientation they are still favored in the ^5^σ-pathway and no ^5^π-pathway is found. Thus, even though the transition states are not in the geometric ideal position, the lowest energy pathway is the ^5^σ-pathway. In a previous study on a biomimetic model complex, we investigated hydrogen atom abstraction from dihydroanthracene by a pentacoordinated iron(IV)-oxo species, where the z-axis was blocked by ligands, so that the substrate had to approach from the side. For that system, analogously to what is seen here, the ^5^σ-pathway was still the lowest in energy (Latifi et al., 2013). Therefore, geometric constraints cannot overcome the orbital energy differences between the ^5^σ− and ^5^π pathways for the HctB active site.

As can be seen from Figure 5, the approach of the substrate on the iron(IV)-oxo center has a profound effect on the barrier height for hydrogen atom abstraction. In particular, pathway **3** appears to be catalytically most effective with a low hydrogen atom abstraction barrier of only 4.9 kcal mol^−1^ (UB3LYP/BS2//UB3LYP/BS1) for QM region **AB**. This is thanks to its geometric orientation, whereby substrate approach to the catalytic center is from the top and the donating hydrogen atom is aligned with the Fe–O axis. With substrates approaching through channels **1**, **2**, and **4**, however, the Fe–O–H angle is strongly bent and the hydrogen atom abstraction barriers are dramatically raised in energy, although they still should be accessible at room temperature.

All transition state geometries display short C–H and long O–H bonds as well as relatively long O–C distances. It may very well be that the enzyme has evolved to do this on purpose in order to guide the chemoselectivity of the reaction and prevent unwanted (more favorable) side reactions. Thus, alternative nonheme iron dioxygenases, such as taurine/αKG dioxygenase have the substrate located in a pocket nearby the iron(IV)-oxo species and a QM/MM study provided a substantially lower hydrogen atom abstraction barrier than found for HctB (Borowski et al., 2004; de Visser, 2006c; Nemukhin et al., 2006; Cicero et al., 2007; Sinnecker et al., 2007; Godfrey et al., 2008; Chen et al., 2011; Bushnell et al., 2012; Mai and Kim, 2016; Wójcik et al., 2016; Álvarez-Barcia and Kästner, 2017). All hydrogen atom abstraction transition states display a large imaginary frequency for the C–H–O stretch vibration; however, distinct differences in the magnitude are seen. In particular, the barriers ^5^**TS**_HA, 2_ and ^5^**TS**_HA, 3_ have imaginary frequencies well over i1,000 cm^−1^ and hence considerable amount of tunneling can be expected. Indeed, previous computational studies on hydrogen atom abstraction reactions by iron(IV)-oxo complexes predicted significant tunneling but also a huge change in barrier upon replacement of the transferring hydrogen atom by deuterium (Kumar et al., 2003, 2004; de Visser, 2006d; Quesne et al., 2016b; Cantú Reinhard et al., 2017b). In particular, the kinetic isotope effect was shown to linearly increase with the value of the imaginary frequency in the transition state.

Table 1 summarizes all hydrogen atom abstraction barriers for models **1**, **2**, and **4**. In general, for model **2** and **4** the UB3LYP/BS1 and UB3LYP/BS2//UB3LYP/BS1 energies are very close, whereas a change from the small QM region to the larger one leads to a drop of about 4 kcal mol^−1^ in the transition state energy. More dramatic changes are seen for model **1**. Thus, with the small QM region large variations in the transition state energy are found dependent on the calculation method and basis set. Moreover, the energies obtained for the small QM region are quite different from those for the larger QM region, where little fluctuation upon changing the basis set is observed. Therefore, the large QM region is the most suitable chemical system for our calculations and hence we used that for the rest of the work.

**Table 1:** Transition state energies of ^5^TS_HA_ as calculated with different QM sizes and basis sets for model **1**, **2**, and **4**^(*a*)^.

**QM region**	**QM method**	**ΔE^‡^_1_**	**ΔE^‡^_2_**	**ΔE^‡^_4_**
A	UB3LYP/BS1	24.5	19.8	21.2
A	UB3LYP/BS2//UB3LYP/BS1	27.4	20.4	23.3
AB	UB3LYP/BS1	22.9	16.2	16.2
AB	UB3LYP/BS2//UB3LYP/BS1	21.5	16.4	20.1
AB	UB3LYP/BS2	23.5		

* *In kcal mol^−1^*.

We even did a full geometry optimization at UB3LYP/BS2:Charmm with QM/MM for the same reaction mechanism using both QM region **A** and **AB** for model **1**. The UB3LYP/BS2 QM/MM optimized geometry of ^5^**TS**_HA, **1**_ is located at a value of 23.5 kcal mol^−1^ above the reactant complex, which is within 2 kcal mol^−1^ of the UB3LYP/BS1 and UB3LYP/BS2//UB3LYP/BS1 results. Moreover, the effect of the basis set on the structures of the optimized geometries is small as well and geometrically they are very close (Supporting Information). Thus, the C–H and H–O distances change from 1.22/1.45 Å calculated with UB3LYP/BS1:Charmm to 1.22/1.43 Å as obtained with UB3LYP/BS2:Charmm for model **1**.

There is quite a bit of fluctuation in the hydrogen atom abstraction barrier depending on the position of the substrate (Figure 5). The lowest barrier is found for model **3** and is 4.9 kcal mol^−1^ in energy (UB3LYP/BS2//UB3LYP/BS1:Charmm with QM region **AB**), while the substrate approach via models **1**, **2**, and **4** are higher in energy by 16.6, 11.5, and 15.2 kcal mol^−1^, respectively. The latter three, although somewhat higher in energy than model **3**, have barrier heights that are still within range of what would be an accessible hydrogen atom abstraction barrier at room temperature. It is very interesting to see that models **1**, **2**, and **4** give hydrogen atom abstraction barriers to within a couple of kcal mol^−1^ of each other and hence, the substrate entrance and positioning appears to have a small effect on the ability of the iron(IV)-oxo(chloro) species to react with substrate. As the substrate is connected to an acyl-carrier protein that is latched on the surface of the protein, the hydrogen atom abstraction barrier is dependent on the distance the substrate terminus can be inserted into the protein. We show here that in all four orientations (models **1**, **2**, **3**, and **4**) the substrate entrance leads to a viable hydrogen atom abstraction channel; however, the ease of hydrogen atom abstraction is strongly influenced by its positioning. Nevertheless, a low hydrogen atom abstraction barrier not necessarily correlates with the correct chemoselectivity of reaction, which is determined in the subsequent reaction step. Therefore, even though the hydrogen atom abstraction is rate determining, it does not decide the product distributions.

**Figure 6:**
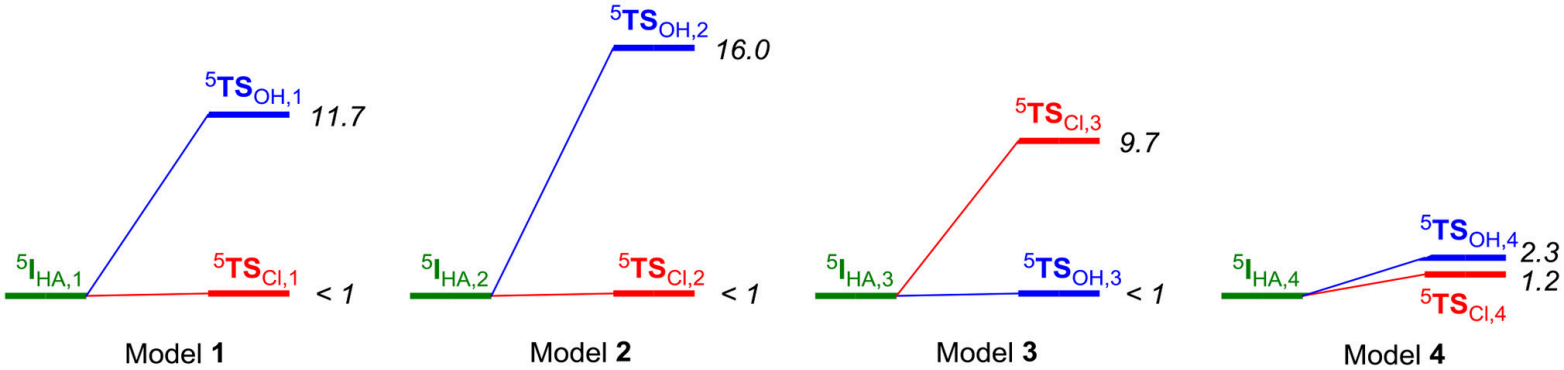
Energy diagram (in kcal mol^−1^) for the pathways leading to halogenation or hydroxylation products from the iron(III)-hydroxo(chloro) intermediate (^5^I_HA_) as calculated with QM/MM in Turbomole:Charmm. Energies (in kcal mol^−1^) obtained with QM/MM at UB3LYP/BS2//UB3LYP/BS1:Charmm and are the result of a full geometry optimization of the local minima and transition states. Energies are taken relative to the value of the radical intermediate state.

The hydrogen atom abstraction reaction leads to an iron(III)-hydroxo(chloro) radical intermediate (**I**_HA_) and proceeds to products through either OH or Cl rebound to the substrate radical to form the products **P**_OH_ and **P**_Cl_. For all four substrate position orientations (**1**, **2**, **3**, and **4**), we then investigated the mechanisms for either Cl rebound or OH rebound. As can be seen from Figure 6, dramatic changes in rebound as well as chemoselectivity of the reaction are obtained depending on the position of the substrate. The ordering and relative energies are not dependent on the choice of the basis set or the size of the QM region: all results point in the same direction and give the same conclusions (Supporting Information). With the substrate entering from a channel above the iron(IV)-oxo species, like through models **1** and **2**, the chemoselectivity is in favor of halogenation over hydroxylation by more than 10 kcal mol^−1^. In particular, the halide rebound step was found to have a negligible barrier in both cases; therefore, halide transfer will be fast in both cases.

A complete reversal of the chemoselectivity is seen in model **3**, but now the OH rebound is barrierless. By contrast, almost identical OH and Cl rebound barriers are found in model **4**. Clearly, the substrate position and orientation has a major effect in the bifurcation pathways and the chemoselectivity of the reaction. Moreover, the calculations presented in Figure 6 implicate that HctB has two viable substrate entrance channels (I and II in Figure 2) and models with the substrate in either of these entrance channels give low-energy hydrogen atom abstraction barriers and preferential halogenation over hydroxylation. However, substrate entrance through channel I in HctB can lead to the location of the substrate in various positions, whereby the one in model **4** probably will give a mixture of products and position **3** gives substrate hydroxylation. As such, substrate positioning and orientation is vital for efficient halogenation in HctB and we predict the enzyme to latch the acyl carrier into substrate entrance channel II and position the substrate in the orientation as shown in model **1**. The structure and orientation of the substrate in model **1** leads to a reaction mechanism with thermochemically accessible hydrogen atom abstraction barrier at room temperature and chemoselectivity preference for substrate halogenation as expected from a halogenase. Most likely, therefore, the other channel located from our structure analysis (substrate entrance channel I) could be involved in guiding halide, dioxygen or α-ketoglutarate reactants into the active site and release of succinate and CO_2_.

**Figure 7:**
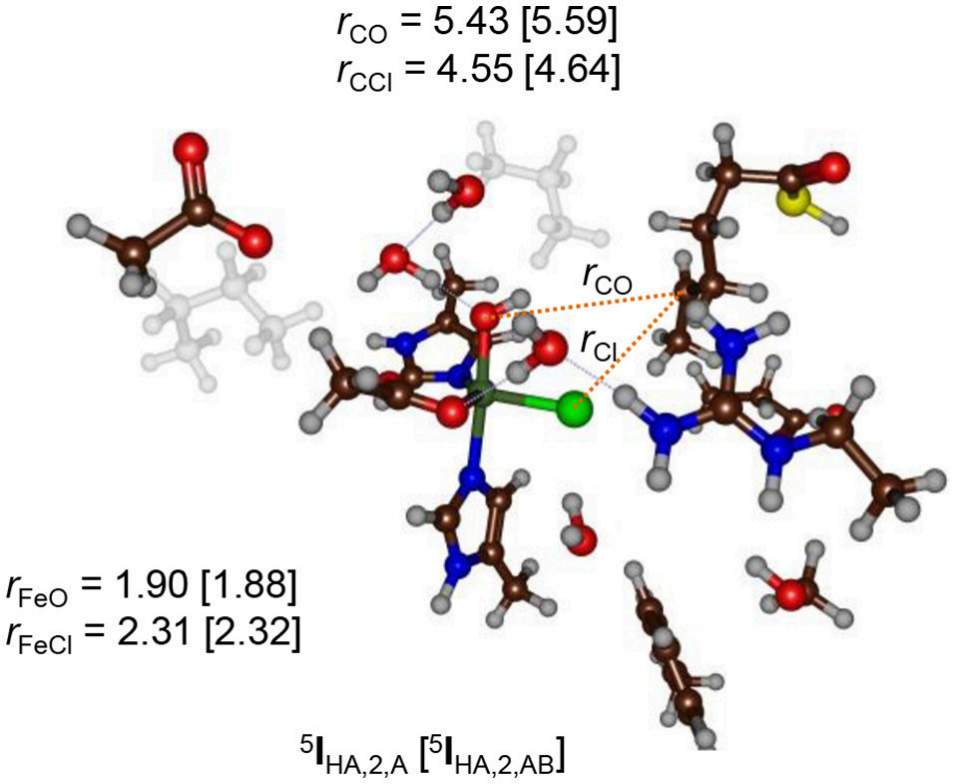
QM/MM optimized geometries of the radical intermediates (^5^I_HA_) of model **2** as calculated with UB3LYP/BS1:Charmm in Turbomole:Charmm. Data are given for QM region A (AB) with bond lengths in angstroms.

The hydrogen atom abstraction barriers reported above in Figure 5 already give a clue on the potential chemoselectivity of the reaction. Thus, in structure ^5^**TS**_HA, **1**_ and ^5^**TS**_HA, **2**_ the Fe–Cl bond has elongated from about 2.28 Å in the reactant complexes to 2.42 and 2.33 Å, respectively. In both cases, the substrate radical is positioned in the quadrant in between the hydroxo and chloride ligands. On the other hand, in ^5^**TS**_HA, **3**_ the radical is located above the hydroxo group and far away from chloride, thereby making chloride rebound unlikely. To understand the large chemoselectivity preference of halogenation over hydroxylation by model **2**, we show in Figure 7 the optimized geometries of the radical intermediates ^5^**I**_HA_ as obtained with QM region **A** and **AB**. The structures are very similar regarding of the size of the QM region chosen. The Fe–O bond has elongated from 1.62 Å in ^5^**Re**_2_ to 1.90 (1.88) Å in ^5^**I**_HA_ using QM region **A** (**AB**), respectively. This is due to occupation of the $\sigma_{\text{z}2}^{*}$ orbital with one electron in the radical intermediate, which is antibonding along the Fe–O bond and hence leads to its elongation. Although the radical has dissipated from the metal center slightly, it is nearer to the halide than to the hydroxo group by almost 1 Å (Figure 7). Moreover, the hydrogen atom of the hydroxo group points in the direction of the radical. However, for hydroxo rebound it needs to move out of the way to make the C–O bond formation possible. In the gas-phase and in apolar environments this O–H rotation costs little energy and hydroxo rebound tends to be low in energy as seen in many aliphatic hydroxylation studies reported previously (Ogliaro et al., 2000; Dey, 2010; Hirao et al., 2011; Bernasconi and Baerends, 2013; Liu et al., 2013; Usharani et al., 2013; Kumar et al., 2014; Zhao et al., 2016). By contrast, in the HctB models **1** and **2** the OH rebound barrier is high in energy due to the largely polar active site with lots of water molecules that prevent the OH group from rotation and keep it in a fixed orientation.

Recent QM/MM studies on the nonheme iron halogenase SyrB2 proposed a strong hydrogen bonding interaction between the hydroxo group in the radical intermediate with an active site Arg residue (Huang et al., 2016). In our optimized geometries of the radical intermediates (^5^**I**_HA_) no direct interaction was found for any substrate binding position, although in several cases a bridging hydrogen bonded water molecule was located in between, see Figure 7. The structure displayed in Figure 7 shows that the water channel that enters the active site pocket serves as a drive to prevent OH rebound and trigger an alternative reaction pathway, namely halogenation. A recent QM/MM study on a cytochrome P450 peroxygenase also had an optimized geometry for an iron(IV)-hydroxo(heme) with an active site whereby the hydroxo group underwent several hydrogen bonding interactions with crystal water molecules in the pocket (Faponle et al., 2016). Studies on the bifurcation pathways for substrate decarboxylation vs. hydroxylation also gave a preference for the energetically unfavorable decarboxylation mechanism similar to what is seen here.

Of course, it should be mentioned that the iron(II)-hydroxo complex with halogenated substrate **P**_Cl_ is not the final step in the catalytic cycle. One could envisage a proton transfer to generate iron(II)-water; however, our structure analysis did not characterize a proton-transfer channel, so that this pathway may not be feasible. Instead, we predict that after the formation of **P**_Cl_, another chloride ion enters the substrate binding pocket and binds to the iron(II) center. A subsequent hydrogen atom abstraction by the iron(II)-hydroxo(chloro) and rebound of halide then gives the iron(II)-water resting state and a dihalogenated substrate as a product.

## Discussion

To gain further insight into the factors that determine the bifurcation patterns of hydroxylation vs. halogenation, we analyzed the chemoselectivity-determining transition states and investigated the role of the protein environment on their ordering and relative energies. In particular, we compare our QM/MM results with small model DFT calculations from previous work, which should give an indication how the protein constraints to the active site structure and how the long-range electrostatic interactions through the protein affect the chemoselectivity of the reaction. Specifically, small gas-phase model complexes of the nonheme iron halogenase reaction mechanism failed to find preferential halogenation over hydroxylation, (de Visser and Latifi, 2009; Kulik et al., 2009; Pandian et al., 2009; Huang et al., 2016) and only the inclusion of the full enzymatic structure and the QM/MM approach led to the correct chemoselectivity for SyrB2 (Borowski et al., 2010).

**Figure 8:**
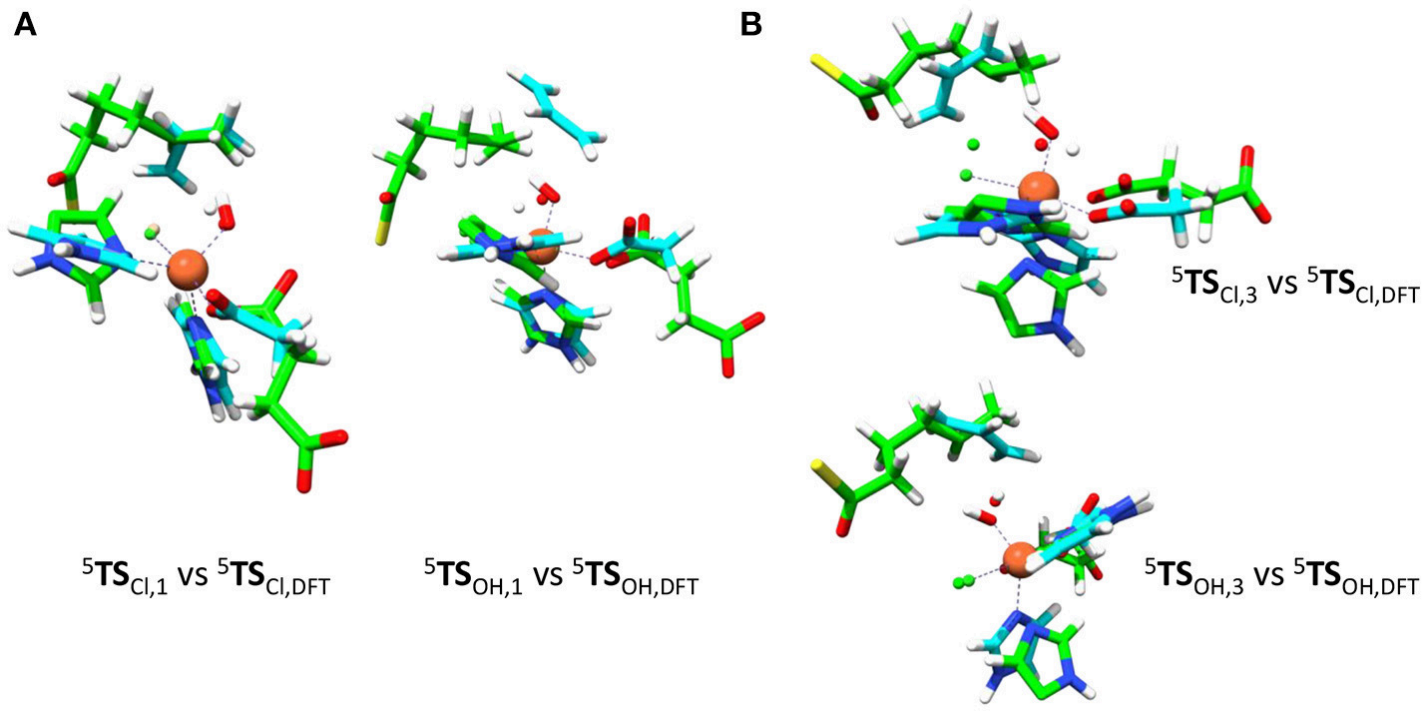
Structural differences of QM/MM optimized geometries and gas-phase DFT models. Overlay of OH and Cl rebound transition state structures (^5^TS_OH_ and ^5^TS_Cl_) for model 1 (left) and model 3 (right). DFT structures (in blue) taken from de Visser and Latifi (2009) and QM/MM structures are in green. **(A)** Overlay of model **1** structures. **(B)** Overlay of model **3** structures.

Therefore, we took the DFT optimized geometries from de Visser and Latifi (2009) calculated at UB3LYP/BS1 with a polarized continuum model with dielectric constant of ε = 5.7 included and perturb the small model complexes with external charges, electric fields and dipole moments and investigate the effects on the product distributions. As will become clear in the discussion, these external perturbations have a profound effect on the chemoselectivity of the reactions. Thus, we started with a detailed comparison of the QM/MM optimized geometries with the gas-phase DFT models from de Visser and Latifi (2009), see Figure 8. Of course, the gas-phase DFT model ignores the secondary coordination sphere of atoms and has large flexibility due to the absence of structural constraints. Indeed, we see from the optimized geometries that the substrate radical in the DFT model approaches the oxidant under an ideal angle (with little or no stereochemical repulsions of active site residues) to the iron(III)-hydroxide(halide) complex ^5^**I**_HA_.

Figure 8 shows overlays of ^5^**TS**_Cl_/^5^**TS**_OH_ structures of models **1** and **3** as compared to those obtained with the DFT model from (de Visser and Latifi, 2009). Overlays of models **2** and **4** with these structures are given in the Supporting Information and show a similar pattern. As can be seen the optimized ^5^**TS**_Cl, **1**_ structure overlaps the gas-phase DFT geometry almost perfectly with the substrate in the same location and under almost the same angle. However, the substrate location is far from the gas-phase orientation for ^5^**TS**_OH, **1**_. Therefore, the substrate binding pocket appears to be designed to accommodate the substrate for efficient halogen transfer and not OH rebound as would be expected of a halogenase enzyme. Despite the good geometric agreement between ^5^**TS**_Cl, **1**_ and the analogous DFT model, the barrier heights relative to their precursor complexes are quite different. Thus, an almost negligible Cl transfer barrier is found for model **1** (Figure 6), whereas the DFT model gave a much larger barrier of about 10 kcal mol^−1^ (de Visser and Latifi, 2009). Clearly, even though the ^5^**TS**_Cl, **1**_ structures are almost the same, the QM/MM barrier is well lower in energy. Accordingly, the protein must stabilize the transition state for halogen transfer dramatically through long-range electrostatic interactions.

We then investigated the overlays of ^5^**TS**_Cl, **3**_/^5^**TS**_OH, **3**_ with those obtained with the DFT model (Figure 8B). As can be seen, the OH rebound structure shows a reasonable match between the two models and hence rebound should stay low in energy. The overlay between the halogen transfer barriers of model **3** and the DFT model are much less good than seen for model **1** and explain why the chemoselectivity is reversed. The comparison of the gas-phase and QM/MM optimized structures, therefore, shows that the substrate should bind in a favorable position for halide rebound. In addition, as shown in Figure 7, water molecules prevent a low-energy OH-rotation pathway and lock the OH group in a non-rebound position. Consequently, substrate positioning in combination with a strong hydrogen bonding network around the hydroxo group destabilize OH rebound and drive the reaction toward halide transfer. A similar conclusion was reached by Mitchell et al. (2017) who reported experimental studies on protein engineering of the nonheme iron halogenase WelO5.

**Figure 9:**
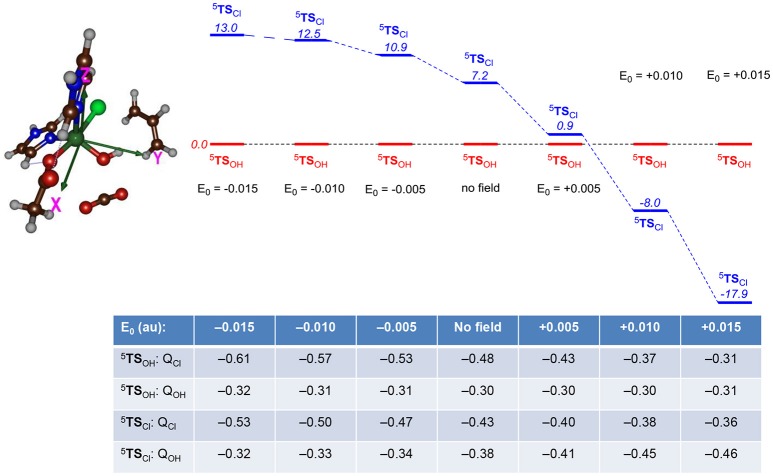
Relative energies (UB3LYP/BS2) between halide and OH rebound barriers (^5^TS_Cl_ vs. ^5^TS_OH_) as calculated with gas-phase DFT calculations in Gaussian with an applied electric field (with magnitude E_0_ in au) included. Relative energies (in italics) are in kcal mol^−1^ and the Table gives group charges (in atomic units) of transition states under the influence of an applied electric field. The electric field perturbations are along the x-axis (along the Fe—Cl bond).

Subsequently, we explored the contribution of the protein environment to the chemoselectivity patterns. We initially investigated perturbations affecting the energetics of the ^5^**TS**_Cl_ and ^5^**TS**_OH_ barriers for the small model complex and particularly focused on electric field effects using previously described procedures (Shaik et al., 2004). We took the gas-phase DFT model and calculated the electronic energy under the influence of an applied electric field with magnitude 0.0050, 0.0100, and 0.0150 au along each individual coordinate axis in both positive and negative directions. Thus, the x-axis is along the Fe–Cl bond, the y-axis along the Fe–OH bond and the z-axis along the Fe–N(His) bond. Relative energies of the two bifurcation transition states ^5^**TS**_Cl_ over ^5^**TS**_OH_ were investigated with electric field effects for the small model and Figure 9 shows the ordering and relative energies of ^5^**TS**_Cl_/^5^**TS**_OH_ through perturbations along the x-axis, i.e., along the Fe–Cl bond. Similar calculations were also performed along the y- and z-axis, whereby the y-axis is defined along the Fe–OH bond and the z-axis is located along the Fe–N(His) bond. The results for the field along the y- and z-axis is given in the Supporting Information; however, in those cases the energies did not change by more than 3 kcal mol^−1^ and in all cases ^5^**TS**_OH_ stayed well below ^5^**TS**_Cl_. The results in Figure 9 show that without an electric field or a field along the negative x-axis, the hydroxylation pathway will be favored over the halogenation pathway by more than 7 kcal mol^−1^. With an electric field pointing along the positive x-axis, by contrast, a dramatic change in relative energies between the two barriers is seen and a chemoselective halogenation over hydroxylation is observed.

To understand the chemoselectivity reversal through the addition of an electric field, we analyzed the unpaired group spin densities and charges of the individual complexes. The group charges under the addition of an electric field to the transition states are given at the bottom of Figure 9. A clear trend emerges from these calculations. Thus, in both **TS**'s charge density (Q) is removed from Cl upon increasing the electric field along the positive x-axis. In particular, the negative charge of the chloride atom decreases from Q_Cl_ = −0.61 with a field of E_0, X_ = −0.015 au to a value of Q_Cl_ = −0.31 with a field of +0.015 au, while an increase of the negative charge with 0.18 units is seen for the same field strengths for ^5^**TS**_Cl_. At the same time, the charge on the OH group stays virtually the same in ^5^**TS**_OH_, whereas it increases from −0.32 to −0.46 in ^5^**TS**_Cl_. Therefore, an applied electric field reduces the charge on chloride and puts more radical character on Cl and enables a Cl^∙^ transfer. At the same time, charge is transferred to iron that is reduced from iron (III) to iron (II).

As a result the ^5^**TS**_Cl_ barrier drops below the ^5^**TS**_OH_ barrier when a positive electric field is applied. It also appears that the ^5^**TS**_OH_ barrier is more strongly affected than the ^5^**TS**_Cl_ barrier to an external electric field and hence the chemoselectivity switch can occur due to destabilization of the hydroxyl rebound barrier. The calculation with an electric field of 0.015 au along the positive x-axis shows that the substrate group loses most of its spin density with respect to the unperturbed system, namely the spin density on the substrate moiety drops from ρ_Sub_ = −0.68 for the system without an electric field to ρ_Sub_ = −0.12 with an electric field of E_0, X_ = +0.015 au. At the same time also the Cl and OH groups lose radical character but to a much lesser extent than the substrate. These results are in line with our previous work on the chemoselectivity of aliphatic hydroxylation vs. epoxidation by a cytochrome P450 model Compound I complex, where we also showed a chemoselectivity switch upon addition of an electric field along a specific axis (Shaik et al., 2004). Further, DFT calculations on a Compound I model of cytochrome c peroxidase with an applied electric field identified radical character on the porphyrin in one direction, whereas with the electric field in the opposite direction a tryptophan radical was obtained (de Visser, 2005).

Our studies, therefore, highlight the importance of the shape and size of the protein of HctB that create an induced electric field on the active site and thereby favorably lead to substrate halogenation to substrate halogenation of the substrate while minimizing substrate hydroxylation. Evidently, an applied electric field from the dipole moment of the protein affects the charge distributions and spin densities of intermediates and transition states in the reaction mechanism and thereby affects the product distributions. In the HctB halogenase enzyme the protein perturbs the active site structure with an induced electric field in such a way that it stabilizes the halogenation pathway and lowers it below the hydroxylation pathway. This effect is the result of considerable destabilization of the OH rebound barrier and a smaller stabilization of the Cl rebound as based upon the changes of the charge distributions between no field and a positive electric field.

**Figure 10:**
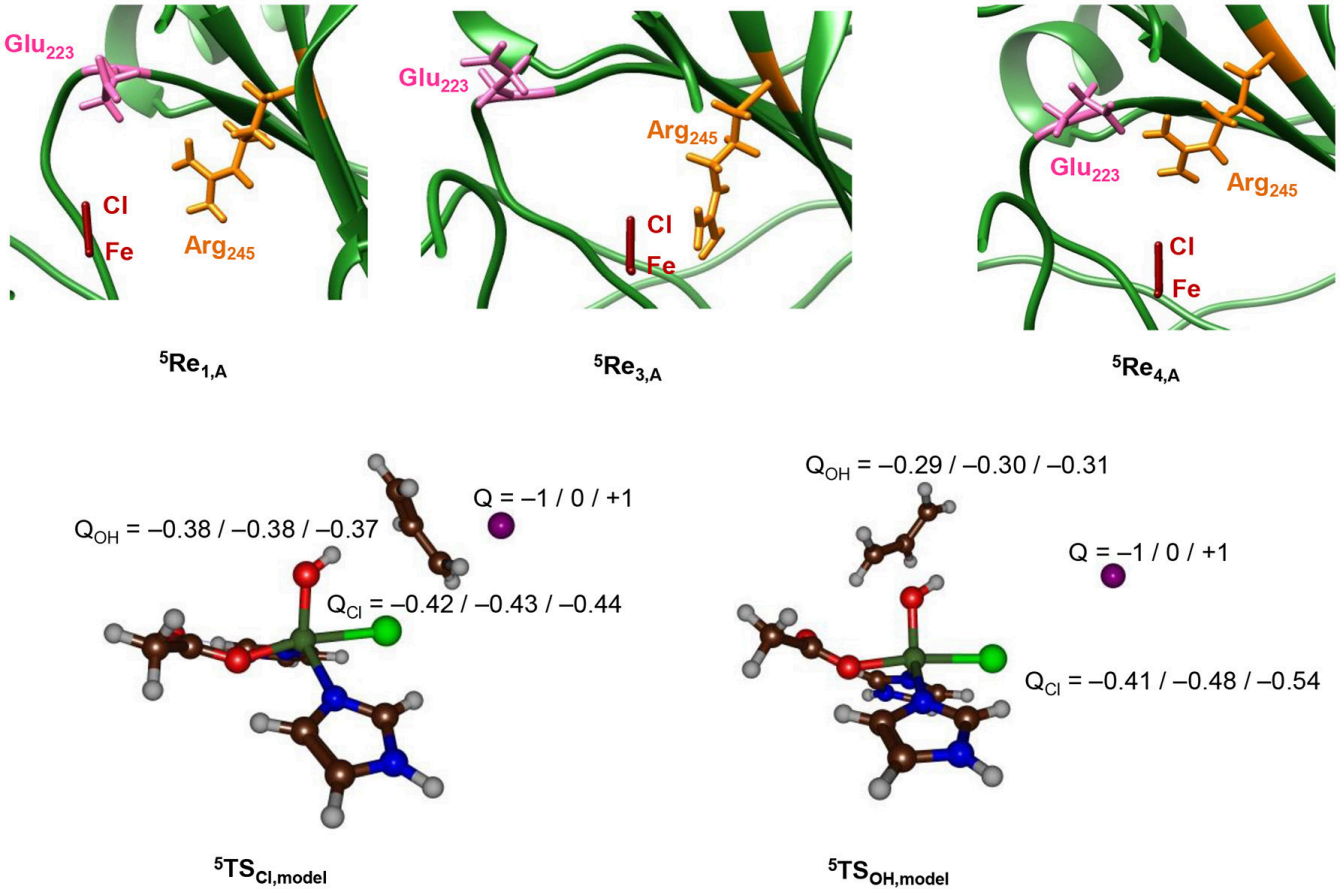
Top: Relative positions of Glu_223_ and Arg_245_ with respect to the Fe–Cl axis in ^5^Re optimized geometries with QM/MM. Optimized geometries calculated at UB3LYP/BS1: Charmm with Turbomole:Charmm. Structure displayed along the Fe–Cl axis by looking through the Fe–O bond. Bottom: Group charges (in atomic units) of the halogenation and hydroxylation transition states of the model system upon addition of a point charge (Q) in the position of the oxygen atoms of Glu_223_ in model **1**.

To find further evidence of environmental perturbations influencing the halogenase vs. hydroxylase activity of the protein, we searched for charged residues in the vicinity of the iron(IV)-oxo(chloride) complex in the pdb structures of ^5^**Re**_1_, ^5^**Re**_2_, ^5^**Re**_3_, and ^5^**Re**_4_. Nearby the halide atom we locate the Glu_223_ and Arg_245_ side chains, whereby the latter may be involved in α-ketoglutarate binding and succinate release. The top part of Figure 10 gives the relative orientations of the Glu_223_ and Arg_245_ amino acids with respect to the iron(IV)-oxo(chloride) in the reactant complexes. As can be seen, the three enzyme models show considerable differences in the position of these amino acids that may incur Coulombic interactions with the active site atoms resulting in chemoselectivity changes. Thus, in model **1** the carboxylate group of Glu_223_ is aligned to the axis that goes through the Fe–Cl bond and appears to be set up to push electron density away from Cl and onto iron. By contrast, in **4** the Glu_223_ and Arg_245_ side chains have formed a salt bridge and now the positively charged Arg is on the axis of the Fe–Cl bond. Consequently, in model **4** charge density will be withdrawn from iron and move to chloride instead making it more difficult to transfer the Cl to substrate. In model **3** the Glu_223_ is moved to the outside part of the protein and hence is located at a relatively large distance from the active center. In addition, the Arg_245_ is positioned much closer to the halide but on the side rather than along the axis. Indeed, model **3** shows a regioselective hydroxylation and no halogenation, whereas in model **1** a preference for halogenation over hydroxylation is seen.

To find out whether the Glu_223_ amino acid residue is near enough to the iron(IV)-oxo(chloride) to affect the charge distributions of the atoms, we took the DFT models of ^5^**TS**_OH_ and ^5^**TS**_Cl_ and included a point charge to the system in the position of the carboxylate oxygen atom of Glu_223_ from model **1**. Single point calculations with a point charge of Q = +1 and −1 were performed and the results are given at the bottom of Figure 10. Surprisingly, only small electronic changes are observed to ^5^**TS**_Cl_ upon addition of a point charge. By contrast, the hydroxyl rebound barrier sees major changes in the charge of the halogen atom under the influence of a point charge. Specifically, the halogen charge increases from −0.48 to −0.41 with a point charge with magnitude −1 included. In addition, the barrier ^5^**TS**_Cl_ drops below ^5^**TS**_OH_ by 4.6 kcal mol^−1^, whereas the energy difference is −20.0 kcal mol^−1^ with a point charge of magnitude +1 included. Therefore, a point charge located at a distance of over 5 Å from the reaction center can incur an electrostatic perturbation that affects the charge distributions in the catalytic reaction center and consequently the chemoselectivity of the reaction. This is an important caveat, given that contrary to the active site and core region of the halogenase domain, the structure of HctB is less well defined by the model, with the positioning of the two other domains and interactions of the halogenase domain not clear/not well defined.

**Figure 11:**
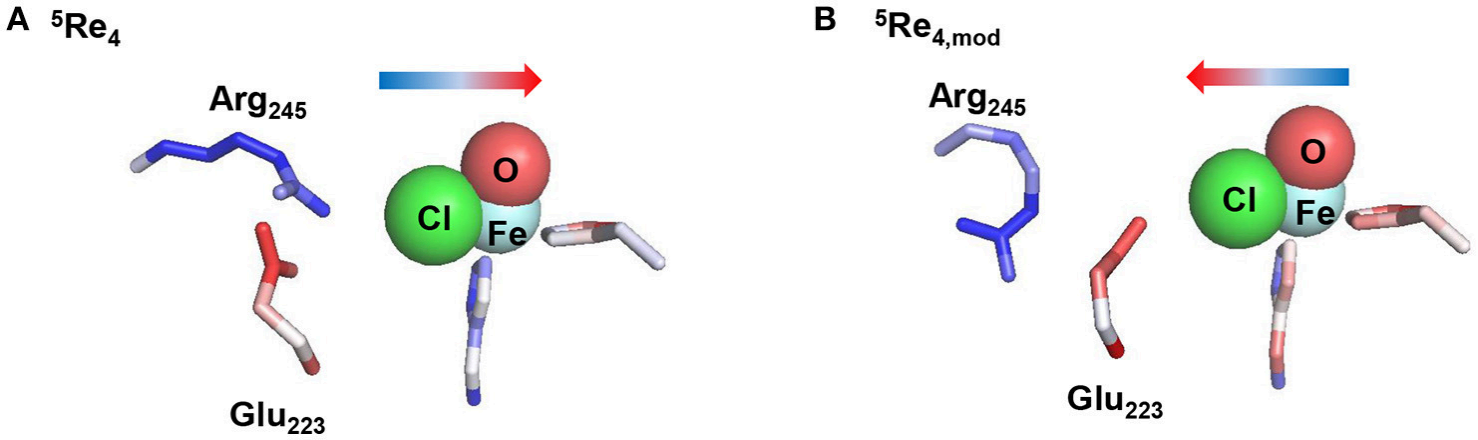
Induced electric field of the salt-bridge between Arg_245_ and Glu_223_ on the iron(IV)-oxo(chloride) active site. Electrostatic potential was calculated for model ^5^Re_4_ **(A)** and model ^5^Re_4,mod_ **(B)**, which is model **4** with rotated Arg_245_ and Glu_223_ residues with respect to the Cl–Fe axis. Color coding for the amino stick representations changes from red (negative potential) to blue (positive potential). The direction of the induced electric field is given by the arrow.

To finally test the effect of the salt bridge between Arg_245_ and Glu_223_ we performed a series of electrostatic potential calculations on model **4** whereby in one case the salt bridge was rearranged so that either Arg_245_ or Glu_223_ is closer to the chloride. Calculations of electrostatic potential fields were made with the Protein-sol server software package (Hebditch et al., 2017) and the results are given in Figure 11, displayed using PyMOL (Schrödinger, LLC). Thus, the salt-bridge between Arg_245_ and Glu_223_ in model **4** (lower panel of Figure 11) induces a change in negative charge on the halide that is pushed toward the iron. Swapping the salt-bridge and changing the position of the Arg and Glu residues has a dramatic effect on the charge donation to the active site and now electron density is withdrawn from iron and moved toward the chloride group. Therefore, the salt-bridge is essential for creating the right charge distribution between the iron and chloride group and induces an electric field effect on the active site that enables efficient substrate halogenation as predicted by the DFT studies reported above.

In summary, the QM/MM and DFT calculations presented here highlight that for effective substrate halogenation, radical character on the halogen atom is needed that can pair up with the substrate radical. Perturbations from the protein, i.e. from nearby located anionic amino acid residues, e.g., Glu_223_, can lead to a push-effect of charge density from the halogen to the metal to achieve this. At the same time, the active site Arg_245_ should not be aligned with the Fe–Cl bond. Our observations are in excellent agreement with recent computational studies on SyrB2 using model complexes (Wong et al., 2013; Srnec et al., 2016; Srnec and Solomon, 2017). In particular, it was proposed that ideal orbital overlap between the substrate radical and halogen atom would lower the halogen transfer transition state below the OH rebound barrier and thereby overcome the large hydroxylation driving force.

## Conclusions

In this work we describe a detailed MM, MD, QM/MM, and DFT study on the nonheme iron halogenase HctB for the first time. Our initial analysis of a structure from the literature revealed several substrate entrance channels. We, therefore, created four models with the substrate in different positions and ran QM/MM calculations on snapshots from these MD simulations. We confirm a reaction mechanism starting from an iron(IV)-oxo species that reacts via hydrogen atom abstraction followed by halogen rebound. Our calculated hydrogen atom abstraction barriers vary dramatically with substrate binding position and a low barrier is found for model **3**, while much higher barriers are seen for models **1** and **2**. Interestingly, two models give preferential halogenation, one model preferential hydroxylation and the fourth one is expected to give a mixture of products. The two models that give preferential halogenation in our analyses use distinct substrate access channels. We analyzed the individual structures and identify an active site Glu residue (Glu_223_), which is unique for HctB as other halogenases typically have a Ser or Lys in that position. Finally, we compared the QM/MM calculations with small gas-phase model complexes and find that even though the gas-phase structure would imply preferential hydroxylation over halogenation, a complete chemoselectivity reversal can be achieved through external perturbations. Calculations on model systems using either an applied electric field along the positive x-axis or with a point charge with magnitude Q = −1 give dominant halogenation, whereas in all other cases, hydroxylation is predicted. It follows, therefore, that the HctB enzyme structure is designed in such a way, so as to destabilize the hydroxylation pathway and give favorable halogenation products. Overall, our calculations show that substrate binding position is essential for an optimal halogenation reaction. This may come at a cost through higher hydrogen atom abstraction barriers, but the enlarged hydrogen atom abstraction barriers may hamper optimal hydroxo rebound and the formation of alcohol products.

## Methods

QM/MM calculations were performed on the mechanism of substrate hydroxylation vs. halogenation in the HctB halogenase using methods and procedures utilized previously on analogous enzymatic systems (Aluri and de Visser, 2007; Kumar et al., 2011; Hernández-Ortega et al., 2015; Quesne et al., 2016a; Tchesnokov et al., 2016; Faponle et al., 2017; Hofer and de Visser, 2018).

### Model building

The work started from the halogenase domain reported by Pratter et al. (2014a,b) which contains an *in silico* docked hexanoyl phosphopentatheinyl (PPT) moiety (channel II, Figure 2). Upon our analysis of the structure; however, another substrate entrance channel was discovered similar to that identified in SyrB2 (channel I, Figure 2) (Blasiak et al., 2006). We manually docked the substrate into each substrate entrance channel and replaced the iron(II)-water group by iron(IV)-oxo and shortened the Fe–O distance to 1.63 Å. In addition, α-ketoglutarate was replaced by succinate (Succ) and the acyl protein carrier was removed. The substrate in channel I was positioned in three different orientations relative to the iron(IV)-oxo. The substrates were positioned in such a way that stereochemical clashes with protein residues along the substrate entrance channels were minimized and after insertion of the substrate the system was solvated. During solvation and molecular dynamics simulations no additional water molecules entered the active site pocket. The structures were charge neutralized with 18 chloride and 13 magnesium ions on the surface of the protein. For example, model **1** contained a total of 32,458 atoms including 9,157 water molecules.

### QM/MM set-up

Hydrogen atoms were added to models **1**–**4** using the PDB2PQR software package at pH = 7, (Dolinsky et al., 2007) whereby all Glu/Asp side chains were in their basic form and all Arg/Lys residues in the acidic forms. The protonation states of histidine residues was decided upon visual inspection of their local environment and His_67_, His_111_, His_157_, His_185_, His_227_, and His_256_ were singly protonated at the N^δ^ position, whereas His_64_, His_163_, His_268_, and His_273_ were protonated at the N^ε^ position. Histidine residue His_8_ was chosen as being doubly protonated at both the N^δ^- and N^ε^-positions.

A constraint geometry optimization with all protein backbone atoms fixed was performed in Charmm, (Brooks et al., 1983) and subsequently the system was solvated in a sphere with a radius of 40 Å. An iterative solvation procedure (Supporting Information Figure S1) with fixed protein backbone was followed by an equilibration and heating procedure to 298 K. For all chemical systems described here, a full molecular dynamics simulation with the Charmm forcefield was performed without constraints for a minimum of 10 ns (Supporting Information Figure S2). All systems relaxed to stable structures and a random low-energy snapshot was chosen as the starting geometry for the QM/MM calculations.

### QM region

We selected a QM region containing the iron(IV)-oxo(chloro) group and included the imidazole groups of His_111_ and His_227_, the acetate terminus of succinate (Succ) and the thiohexanoic acid arm of the substrate as our minimal QM region **A**. In addition, a larger QM region was tested that included the full or partial amino acid R-groups within 6 Å of the iron(IV)-oxo(chloro) structure: QM region **AB**. For model **1**, the large QM region **AB** contained the amino acid side chains of Ile_108_, Val_113_, Asp_200_, Asp_202_, Glu_223_, Val_225_, Met_226_, Arg_245_, and six water molecules. QM region **AB** for Model **2** is described with the same amino acids except Asp_202_, Val_225_, and Met_245_ and in addition also includes Ser_141_ and Phe_221_ and four water molecules. Model **3** in its most elaborate form covers Ile_108_, Val_113_, Ser_141_, Asp_200_, Phe_221_, Asn_243_, Arg_245_, and four water molecules. Finally, the large QM region for Model **4** includes Ile_108_, Val_113_, Phe_221_, Arg_245_, and five water molecules.

### QM/MM calculations

Subsequent QM/MM calculations of the different substrate bound iron(IV)-oxo(chloro) enzyme intermediates were performed in Turbomole:Charmm and linked via ChemShell (Ahlrichs et al., 1989; Smith and Forester, 1996; Sherwood et al., 2003). Density functional theory methods at the unrestricted B3LYP level of theory (Lee et al., 1988; Becke, 1993) were applied to the QM region in Turbomole with an SV(P) basis set on all atoms: basis set BS1 (Schafer et al., 1992). Single point calculations on the optimized structures in QM/MM were performed with an all-electron Wachters-type basis set on iron and def2-TZV(P) on the rest of the atoms: basis set BS2 (Wachters, 1970; Hay, 1977; Krishnan et al., 1980; Bauschlicher et al., 1989). For several systems we tested a full geometry optimization of using basis set BS2 but found similar structures and relative energies to those obtained with BS1; hence BS1 was used for all systems. The MM region was described with the Charmm forcefield (Brooks et al., 1983). A detailed benchmark study comparing computational free energies of activation with experimental data from Eyring plots gave good agreement between experiment and theory for the methods described here (Cantú Reinhard et al., 2016a). Furthermore, these methods were also seen to excellently reproduce reduction potentials of copper proteins, (Fowler et al., 2017) and regio- and chemoselectivities of reaction mechanisms (Jastrzebski et al., 2014; Barman et al., 2016b; Yang et al., 2016).

Geometries were optimized at UB3LYP/BS1 in Turbomole:Charmm and a frequency calculation on the QM region only was done to test whether it was a local minimum or first order saddle point. Geometry scans with one degree of freedom fixed were performed along a specified reaction coordinate. The maximum of these scans were subjected to a full geometry optimization of a first-order saddle point and the starting and end points of the scans established the proposed reaction paths.

## Author contributions

AT and SdV devised the project. AT ran the QM/MM calculations. NF and JW performed the electrostatic calculations. AT, NF, GS, and SdV wrote the paper.

### Conflict of interest statement

The authors declare that the research was conducted in the absence of any commercial or financial relationships that could be construed as a potential conflict of interest.
