# Supplementary material for: Does Substrate Positioning Affect the Selectivity and Reactivity in the Hectochlorin Biosynthesis Halogenase?
Source: Front Chem. 2018 Oct 30;6:513. doi: 10.3389/fchem.2018.00513 (PMC6218459; doi:10.3389/fchem.2018.00513)
Supplement: Supplementary file 1 [file Data_Sheet_1.PDF]

Supplementary Material

Does substrate positioning affect the selectivity and reactivity in the hectochlorin biosynthesis halogenase?

Amy Timmins[1], Nicholas J. Fowler[2], Jim Warwicker[2], Grit D. Straganz[3,4], and Sam P. de Visser[1*]

[1] The Manchester Institute of Biotechnology and School of Chemical Engineering and Analytical Science, The University of Manchester, 131 Princess Street, Manchester, M1 7DN, United Kingdom

[2] The Manchester Institute of Biotechnology and School of Chemistry, The University of Manchester, 131 Princess Street, Manchester, M1 7DN, United Kingdom

[3] Graz University of Technology, Institute of Biochemistry, Petergasse 12, A-8010 Graz, Austria

[4] Graz University, Institute of Molecular Biosciences, Humboldtstrasse 59, A-8010 Graz, Austria

*** Correspondence:**
Corresponding Author
sam.devisser@manchester.ac.uk

Part I: Model set-up and MD

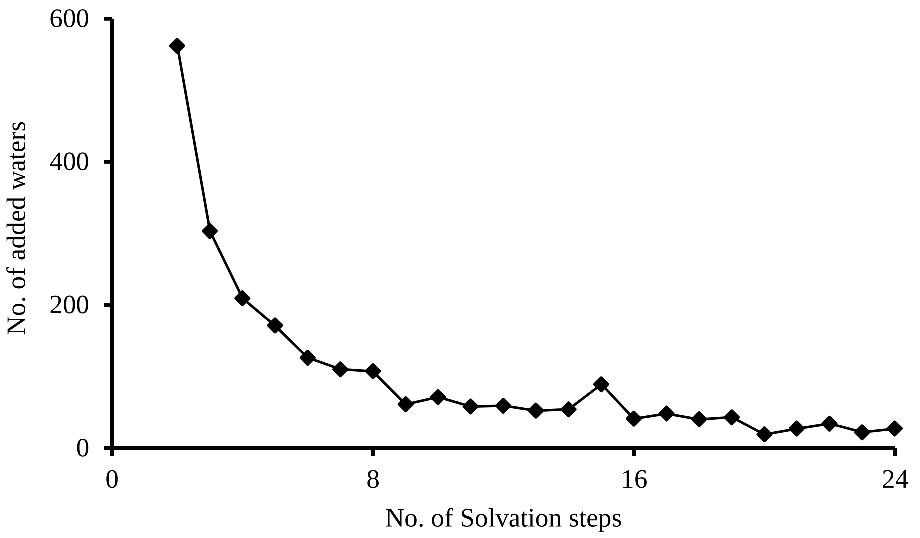

Supplementary Figure 1. Iterative solvation procedure of HctB, model **1**. For clarity, the first solvation step during which 6922 waters were added, has been omitted.

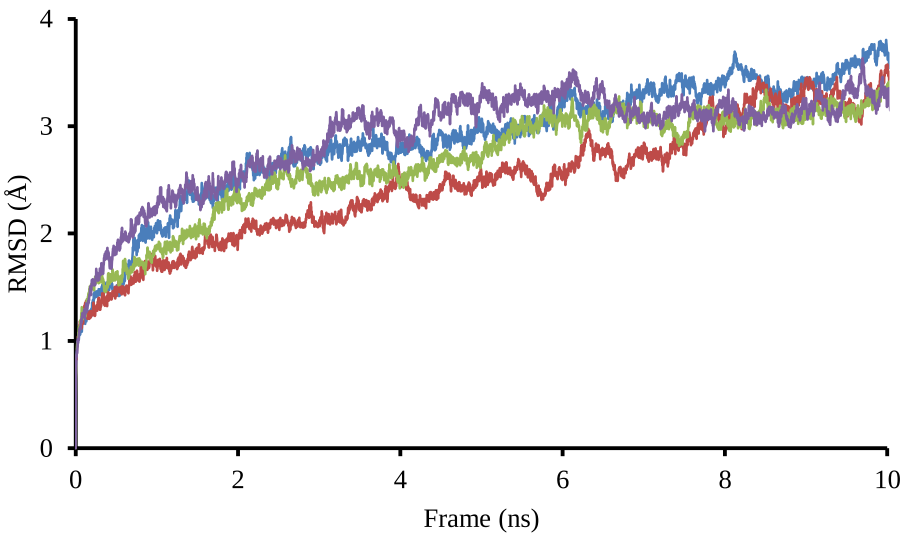

Supplementary Figure 2. MD simulations. Overlay of the first 10ns MD simulation for the four substrate binding models. RMSD plots of each HctB model as a function of time: model 1 (blue), model 2 (wine red), model 3 (green), model 4 (purple).

Part II: DFT calculations

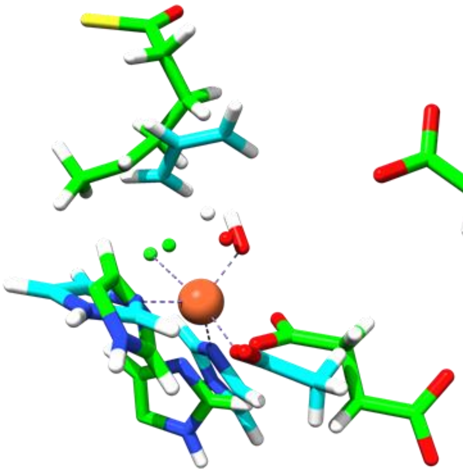

$^{5}\mathbf{TS}_{Cl,2}$ vs $^{5}\mathbf{TS}_{Cl,DFT}$

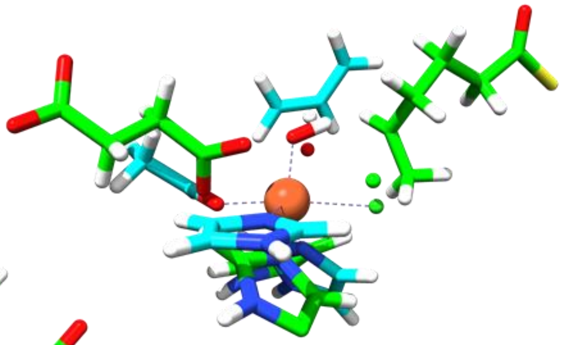

$^{5}\mathbf{TS}_{OH,2}$ vs $^{5}\mathbf{TS}_{OH,DFT}$

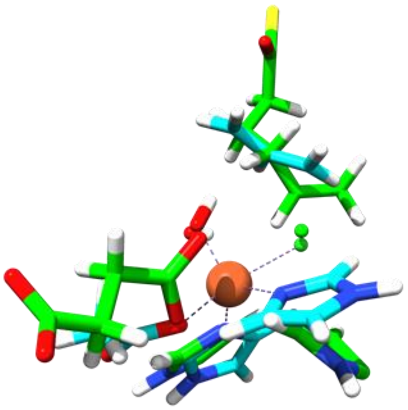

$^{5}\mathbf{TS}_{Cl,4}$ vs $^{5}\mathbf{TS}_{Cl,DFT}$

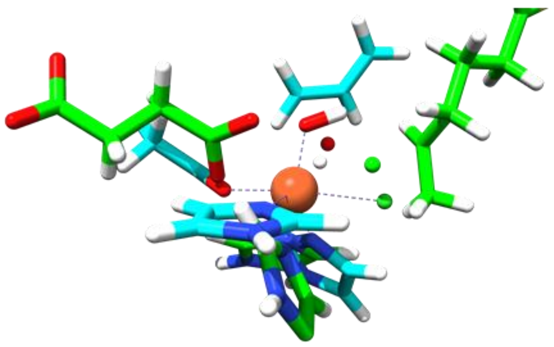

$^{5}\mathbf{TS}_{OH,4}$ vs $^{5}\mathbf{TS}_{OH,DFT}$

Supplementary Figure 3. Overlay of QM/MM optimized OH/Cl rebound barriers for model **2** (top) and model **4** (bottom) as compared to gas-phase DFT models from de Visser & Latifi (*J. Phys. Chem. B* **2009**, *113*, 12–14). QM/MM structures in green and DFT geometries in light blue.

Table S1 - Relative energies (in kcal mol^{-1}) of small model halogenation versus hydroxylation transition states under the influence of an applied electric field (given in au). Data show for perturbations along the y-axis and z-axis. A positive energy gap refers to preferential hydroxylation.

Axis/Applied Electric Field (in 10^{-3} au)	–15	–10	–5	no field	+5	+10	+15
y-axis: ΔE	8.8	7.0	6.4	7.2	8.4	9.5	10.5
z-axis: ΔE	5.6	6.3	6.8	7.2	7.3	7.4	7.4

Table S2 – Group spin densities of small model halogenation transition state under the influence of an applied electric field (given in au) along the x-, y-, and z-axis.

	Fe	His_1	His_2	Cl	CO2	Suc	OH	Sub
**No_field**	3.91	0.08	0.07	0.14	0.00	0.16	0.31	-0.68
**x+0.005**	3.85	0.07	0.06	0.14	0.00	0.12	0.25	-0.49
**x+0.010**	3.77	0.05	0.05	0.10	0.00	0.08	0.20	-0.25
**x+0.150**	3.71	0.05	0.05	0.08	0.00	0.07	0.17	-0.12
**x-0.005**	3.95	0.10	0.08	0.10	0.00	0.22	0.36	-0.81
**x-0.010**	3.96	0.10	0.08	0.06	0.00	0.29	0.39	-0.87
**x-0.015**	3.95	0.10	0.07	0.02	0.00	0.36	0.39	-0.89
**y+0.005**	3.96	0.10	0.09	0.12	0.00	0.20	0.33	-0.79
**y+0.010**	3.99	0.10	0.11	0.08	0.00	0.23	0.33	-0.84
**y+0.015**	4.00	0.11	0.13	0.04	0.00	0.25	0.31	-0.84
**y-0.005**	3.84	0.07	0.05	0.13	0.00	0.13	0.28	-0.51
**y-0.010**	3.76	0.06	0.04	0.09	0.00	0.10	0.24	-0.29
**y-0.015**	3.65	0.04	0.03	0.02	0.00	0.07	0.21	-0.02
**z+0.005**	3.89	0.07	0.07	0.14	0.00	0.15	0.32	-0.65
**z+0.010**	3.87	0.06	0.08	0.13	0.00	0.15	0.32	-0.62
**z+0.015**	3.85	0.05	0.08	0.13	0.00	0.14	0.32	-0.58
**z-0.005**	3.92	0.10	0.07	0.14	0.00	0.17	0.31	-0.70
**z-0.010**	3.93	0.12	0.06	0.13	0.00	0.17	0.30	-0.73
**z-0.015**	3.94	0.15	0.06	0.13	0.00	0.18	0.29	-0.76

Table S3 – Group charges of small model halogenation transition state under the influence of an applied electric field (given in au) along the x-, y-, and z-axis.

	Fe	His_1	His_2	Cl	CO2	Suc	OH	Sub
**No_field**	0.90	0.16	0.18	-0.43	-0.02	-0.55	-0.38	0.13
**x+0.005**	0.86	0.14	0.17	-0.40	-0.03	-0.61	-0.41	0.27
**x+0.010**	0.80	0.13	0.17	-0.38	-0.04	-0.66	-0.45	0.43
**x+0.015**	0.77	0.12	0.17	-0.36	-0.05	-0.70	-0.46	0.51
**x-0.005**	0.93	0.17	0.18	-0.47	-0.01	-0.48	-0.34	0.01
**x-0.010**	0.95	0.18	0.18	-0.50	-0.01	-0.41	-0.33	-0.06
**x-0.015**	0.95	0.17	0.17	-0.53	0.00	-0.34	-0.32	-0.09
**y+0.005**	0.94	0.17	0.22	-0.43	-0.02	-0.52	-0.37	0.01
**y+0.010**	0.96	0.18	0.26	-0.44	-0.02	-0.49	-0.38	-0.07
**y+0.015**	0.97	0.19	0.30	-0.43	-0.03	-0.46	-0.39	-0.13
**y-0.005**	0.86	0.14	0.14	-0.43	-0.02	-0.58	-0.39	0.28
**y-0.010**	0.80	0.13	0.10	-0.45	-0.02	-0.61	-0.41	0.45
**y-0.015**	0.73	0.11	0.06	-0.46	-0.02	-0.63	-0.42	0.63
**z+0.005**	0.89	0.12	0.20	-0.44	-0.01	-0.55	-0.37	0.16
**z+0.010**	0.87	0.08	0.21	-0.44	0.00	-0.54	-0.37	0.20
**z+0.015**	0.85	0.04	0.23	-0.45	0.01	-0.54	-0.37	0.23
**z-0.005**	0.91	0.20	0.16	-0.42	-0.03	-0.55	-0.38	0.10
**z-0.010**	0.92	0.25	0.14	-0.41	-0.04	-0.55	-0.38	0.07
**z-0.015**	0.92	0.30	0.13	-0.41	-0.05	-0.55	-0.38	0.03

Table S4 – Group spin densities of small model hydroxylation transition state under the influence of an applied electric field (given in au) along the x-, y-, and z-axis.

	Fe	His_1	His_2	Cl	CO2	Suc	OH	Sub
**No_field**	3.95	0.09	0.08	0.16	0.00	0.17	0.39	-0.85
**x+0.005**	3.96	0.09	0.09	0.20	0.00	0.15	0.39	-0.88
**x+0.010**	3.96	0.10	0.09	0.25	0.00	0.13	0.38	-0.90
**x+0.015**	3.95	0.10	0.09	0.30	0.00	0.11	0.36	-0.92
**x-0.005**	3.94	0.09	0.08	0.13	0.00	0.20	0.39	-0.82
**x-0.010**	3.92	0.08	0.07	0.10	0.00	0.22	0.38	-0.78
**x-0.015**	3.90	0.08	0.06	0.08	0.00	0.25	0.37	-0.75
**y+0.005**	3.99	0.10	0.10	0.18	0.00	0.20	0.36	-0.92
**y+0.010**	4.01	0.11	0.12	0.18	0.00	0.22	0.31	-0.94
**y+0.015**	4.02	0.11	0.14	0.18	0.00	0.23	0.25	-0.93
**y-0.005**	3.90	0.08	0.06	0.13	0.00	0.14	0.38	-0.69
**y-0.010**	3.82	0.07	0.05	0.10	0.00	0.10	0.33	-0.47
**y-0.015**	3.72	0.05	0.04	0.07	0.00	0.07	0.22	-0.19
**z+0.005**	3.95	0.08	0.09	0.16	0.00	0.17	0.40	-0.85
**z+0.010**	3.94	0.07	0.10	0.16	0.00	0.17	0.42	-0.85
**z+0.015**	3.93	0.06	0.10	0.16	0.00	0.17	0.43	-0.85
**z-0.005**	3.96	0.11	0.07	0.16	0.00	0.17	0.37	-0.85
**z-0.010**	3.96	0.13	0.07	0.16	0.00	0.17	0.36	-0.86
**z-0.015**	3.96	0.15	0.06	0.16	0.00	0.17	0.34	-0.86

Table S5 – Group charges of small model hydroxylation transition state under the influence of an applied electric field (given in au) along the x-, y-, and z-axis.

	Fe	His_1	His_2	Cl	CO2	Suc	OH	Sub
**No_field**	0.88	0.17	0.19	-0.48	0.00	-0.54	-0.30	0.08
**x+0.005**	0.88	0.17	0.20	-0.43	0.00	-0.58	-0.30	0.05
**x+0.010**	0.88	0.18	0.21	-0.37	0.00	-0.61	-0.30	0.02
**x+0.015**	0.87	0.18	0.22	-0.31	0.00	-0.64	-0.31	0.00
**x-0.005**	0.88	0.16	0.18	-0.53	0.00	-0.51	-0.31	0.12
**x-0.010**	0.87	0.15	0.17	-0.57	0.01	-0.47	-0.31	0.15
**x-0.015**	0.86	0.15	0.16	-0.61	0.01	-0.43	-0.32	0.19
**y+0.005**	0.91	0.18	0.23	-0.47	0.00	-0.52	-0.32	0.00
**y+0.010**	0.92	0.19	0.26	-0.47	0.00	-0.50	-0.35	-0.05
**y+0.015**	0.93	0.19	0.30	-0.48	0.00	-0.49	-0.37	-0.09
**y-0.005**	0.84	0.15	0.15	-0.50	0.00	-0.58	-0.30	0.24
**y-0.010**	0.79	0.13	0.11	-0.52	0.00	-0.61	-0.32	0.43
**y-0.015**	0.72	0.11	0.07	-0.54	0.00	-0.64	-0.35	0.63
**z+0.005**	0.88	0.13	0.21	-0.48	0.01	-0.54	-0.29	0.08
**z+0.010**	0.87	0.09	0.23	-0.48	0.02	-0.53	-0.28	0.08
**z+0.015**	0.85	0.05	0.26	-0.48	0.03	-0.52	-0.27	0.08
**z-0.005**	0.88	0.21	0.17	-0.48	0.00	-0.55	-0.31	0.09
**z-0.010**	0.88	0.25	0.15	-0.47	-0.01	-0.55	-0.33	0.08
**z-0.015**	0.88	0.30	0.13	-0.47	-0.02	-0.56	-0.34	0.08

Part III: Cartesian coordinates

Coordinates of small model $^5TS_{Cl}$

26	0.000000000	0.000000000	0.000000000
7	0.000000000	0.000000000	2.118112000
6	1.151793000	0.000000000	2.808069000
7	0.869163000	0.047932000	4.139223000
6	-0.516279000	0.079097000	4.309120000
6	-1.046960000	0.051272000	3.046722000
1	2.149409000	-0.025320000	2.369256000
1	1.559010000	0.062751000	4.872817000
1	-0.979836000	0.115995000	5.279252000
1	-2.070943000	0.076542000	2.721900000
7	-0.643858000	-1.647794000	-1.210589000
6	-1.873118000	-2.005463000	-1.596789000
7	-1.794139000	-3.096670000	-2.410461000
6	-0.450898000	-3.451083000	-2.549302000
6	0.253978000	-2.545302000	-1.800292000
1	-2.779535000	-1.502193000	-1.310814000
1	-2.576953000	-3.564827000	-2.837907000
1	-0.133857000	-4.286369000	-3.148810000
1	1.307358000	-2.443307000	-1.619177000
17	-2.545498000	0.592196000	0.159839000
6	1.254653000	2.157568000	-3.061868000
8	2.080255000	1.302891000	-3.043933000
8	0.485884000	3.046807000	-3.249611000
6	3.098680000	-0.539685000	0.057372000
8	1.802914000	-0.788989000	-0.106728000
6	3.970639000	-0.834612000	-1.144307000
1	3.906354000	-1.898734000	-1.402647000
1	5.008850000	-0.580306000	-0.925439000
1	3.612427000	-0.259362000	-2.002629000
8	0.251556000	1.643057000	-0.825680000
8	3.591386000	-0.115867000	1.134352000
6	-2.108729000	4.334109000	-1.251975000
1	-1.972004000	3.922797000	-2.248073000
1	-1.510613000	5.204185000	-1.001108000
6	-2.982993000	3.780922000	-0.350748000
1	-3.054936000	4.226310000	0.641178000
6	-3.795283000	2.650486000	-0.604667000
1	-3.850052000	2.235958000	-1.605139000
1	-0.442043000	2.328429000	-0.782957000
1	-4.586192000	2.374358000	0.080932000

Coordinates of small model $^5TS_{OH}$

26	0.000000000	0.000000000	0.000000000
7	0.000000000	0.000000000	2.120284000
6	1.126254000	0.000000000	2.849079000
7	0.797778000	-0.021352000	4.171177000
6	-0.593363000	-0.038276000	4.291784000
6	-1.079921000	-0.020391000	3.011126000
1	2.131987000	0.053410000	2.441099000
1	1.459515000	-0.022520000	4.930495000
1	-1.090932000	-0.059281000	5.245371000
1	-2.091234000	-0.007335000	2.646485000
7	-0.470914000	-1.644377000	-1.273347000
6	-1.673505000	-2.001229000	-1.738524000
7	-1.538823000	-3.087735000	-2.549168000
6	-0.188459000	-3.439102000	-2.603452000
6	0.465507000	-2.534968000	-1.808220000
1	-2.595174000	-1.496742000	-1.505545000
1	-2.291225000	-3.552208000	-3.031525000
1	0.167666000	-4.271419000	-3.184717000
1	1.505478000	-2.430862000	-1.563913000
17	-2.422423000	0.525455000	0.064216000
6	1.271004000	1.856264000	-3.280395000
8	1.507575000	0.698012000	-3.397288000
8	1.068404000	3.026632000	-3.256261000
6	3.102835000	-0.257861000	0.062138000
8	1.853651000	-0.685919000	-0.065176000
6	4.009650000	-0.542865000	-1.118531000
1	4.239219000	-1.615655000	-1.154776000
1	4.944705000	0.010613000	-1.017657000
1	3.501559000	-0.280110000	-2.050542000
8	0.434223000	1.673484000	-0.710267000
8	3.538994000	0.312053000	1.097599000
6	2.009908000	3.452165000	0.176058000
1	2.359220000	3.502045000	-0.848763000
1	2.455001000	2.694807000	0.808701000
6	1.118866000	4.414564000	0.668647000
1	0.844918000	4.354204000	1.721838000

6	0.542969000	5.411683000	-0.095686000	1	-0.215304000	2.402956000	-0.669997000
1	0.759599000	5.500822000	-1.156825000	1	-0.162472000	6.119186000	0.326759000

Table S6 – Group spin densities of small model hydroxylation transition state with either a positive, negative, or no point charge in the position of where either the proximal or distal oxygen of Glu_{223} would be located in model 1.

	Fe	His	His	Cl	CO_2	Succ	OH	Sub
**No_PC**	3.95	0.09	0.08	0.16	0.00	0.17	0.39	-0.85
**$PC_{+ve, distal}$**	3.95	0.09	0.08	0.14	0.00	0.19	0.39	-0.84
**$PC_{+ve, proximal}$**	3.96	0.09	0.08	0.13	0.00	0.20	0.38	-0.85
**$PC_{-ve, distal}$**	3.95	0.09	0.08	0.19	0.00	0.16	0.39	-0.86
**$PC_{-ve, proximal}$**	3.94	0.09	0.08	0.20	0.00	0.15	0.40	-0.85

Table S7 – Group charges of small model hydroxylation transition state with either a positive, negative, or no point charge in the position of where either the proximal or distal oxygen of Glu_{223} would be located in model 1.

	Fe	His	His	Cl	CO_2	Succ	OH	Sub
**No_PC**	0.88	0.17	0.19	-0.48	0.00	-0.54	-0.30	0.08
**$PC_{+ve, distal}$**	0.89	0.17	0.20	-0.53	0.00	-0.53	-0.31	0.10
**$PC_{+ve, proximal}$**	0.90	0.18	0.20	-0.54	0.01	-0.52	-0.31	0.09
**$PC_{-ve, distal}$**	0.87	0.16	0.18	-0.43	0.00	-0.56	-0.30	0.07
**$PC_{-ve, proximal}$**	0.86	0.16	0.18	-0.41	0.00	-0.57	-0.29	0.08

Table S8 – Group spin densities of small model halogenation transition state with either a positive, negative, or no point charge in the position of where either the proximal or distal oxygen of Glu_{223} would be located in model 1.

	Fe	His	His	Cl	CO_2	Succ	OH	Sub
**No_PC**	3.91	0.08	0.07	0.14	0.00	0.16	0.31	-0.68
**$PC_{+ve, distal}$**	3.90	0.08	0.07	0.13	0.00	0.16	0.32	-0.67
**$PC_{+ve, proximal}$**	3.90	0.08	0.07	0.13	0.00	0.17	0.32	-0.68
**$PC_{-ve, distal}$**	3.91	0.09	0.07	0.14	0.00	0.16	0.31	-0.68
**$PC_{-ve, proximal}$**	3.91	0.09	0.07	0.14	0.00	0.15	0.31	-0.67

Table S9 – Group charges of small model halogenation transition state with either a positive, negative, or no point charge in the position of where either the proximal or distal oxygen of Glu_{223} would be located in model 1.

	Fe	His	His	Cl	CO_2	Succ	OH	Sub
**No_PC**	0.90	0.16	0.18	-0.43	-0.02	-0.55	-0.38	0.13
**$PC_{+ve, distal}$**	0.90	0.13	0.19	-0.44	-0.02	-0.54	-0.37	0.14
**$PC_{+ve, proximal}$**	0.90	0.13	0.19	-0.44	-0.01	-0.53	-0.37	0.13
**$PC_{-ve, distal}$**	0.91	0.19	0.17	-0.42	-0.02	-0.56	-0.38	0.12
**$PC_{-ve, proximal}$**	0.90	0.19	0.17	-0.42	-0.02	-0.56	-0.38	0.13

Part IV: QMMM calculations

Model 1

Table S10 - Absolute energies (in au) of QM/MM optimized structures of HctB model 1 reaction at UB3LYP/BS1 in Turbomole:Charmm. All data for small region A.

	E_{QM}	E_{MM}	$E_{QM/MM}$	ZPE	G_{corr}	$E_{QM/MM+ZPE}$	$E_{QM/MM+Gcorr}$
	(au)	(au)	(au)	(au)	(au)	(au)	(au)
^{1}**Re$_{1}$**	-3187.48014	-170.52140	-3358.00154	0.37365	0.31807	-3357.62789	-3357.68347
^{3}**Re$_{1}$**	-3187.50726	-170.51622	-3358.02348	0.37327	0.31470	-3357.65021	-3357.70878
^{5}**Re$_{1}$**	-3187.52933	-170.49804	-3358.02737	0.37462	0.31756	-3357.65274	-3357.70981
^{5}**TS$_{HA,1}$**	-3187.49022	-170.54535	-3358.03558	0.36861	0.31259	-3357.66696	-3357.72299
^{5}**Int$_{1}$**	-3187.52450	-170.54936	-3358.07386	0.36711	0.30631	-3357.70674	-3357.76755
^{5}**TS$_{Cl,1}$**	-3187.52935	-170.53349	-3358.06284	0.36808	0.30933	-3357.69476	-3357.75351
^{5}**TS$_{OH,1}$**	-3187.50765	-170.54251	-3358.05017	0.37047	0.31315	-3357.67969	-3357.73702
^{5}**Pr$_{Cl,1}$**	-3187.58334	-170.59344	-3358.17678	0.37271	0.31399	-3357.80407	-3357.86279
^{5}**Pr$_{OH,1}$**	-3187.56622	-170.54640	-3358.11262	0.37651	0.32018	-3357.73612	-3357.79244

Table S11 - Relative energies (in kcal/mol) of QM/MM optimized structures of the HctB model 1 reaction at UB3LYP/BS1 in Turbomole:Charmm. All data for small QM region A.

	RE_{QM}	RE_{QM+ZPE}	$RE_{QM+Gcorr}$
	kcal/mol	kcal/mol	kcal/mol
**$^{1}Re_{1}$**	30.86	30.25	31.19
**$^{3}Re_{1}$**	13.85	13.00	12.06
**$^{5}Re_{1}$**	0.00	0.00	0.00
**$^{5}TS_{HA,1}$**	24.54	20.77	21.42
**$^{5}Int_{1}$**	3.03	-1.68	-4.03
**$^{5}TS_{Cl,1}$**	-0.01	-4.12	-5.18
**$^{5}TS_{OH,1}$**	13.60	11.00	10.84
**$^{5}Pr_{Cl,1}$**	-33.90	-35.09	-36.13
**$^{5}Pr_{OH,1}$**	-23.15	-21.97	-21.50

Table S12 - Absolute energies (in au) of QM/MM optimized structures of HctB model 1 reaction at UB3LYP/BS2 in Turbomole:Charmm. All data for small region A.

	E_{QM}	E_{MM}	$E_{QM/MM}$	ZPE	G_{corr}	$E_{QM/MM+ZPE}$	$E_{QM/MM+Gcorr}$
	(au)	(au)	(au)	(au)	(au)	(au)	(au)
**^{5}Re$_{1}$**	-3189.27526	-170.49804	-3359.77330	0.37462	0.31756	-3359.39868	-3359.45575
**^{5}TS$_{HA,1}$**	-3189.23162	-170.54535	-3359.77697	0.36861	0.31259	-3359.40836	-3359.46438
**^{5}Int$_{1}$**	-3189.27269	-170.54936	-3359.82205	0.36711	0.30631	-3359.45494	-3359.51575
**^{5}TS$_{Cl,1}$**	-3189.27830	-170.53349	-3359.81179	0.36808	0.30933	-3359.44371	-3359.50246
**^{5}TS$_{OH,1}$**	-3189.25409	-170.54251	-3359.79660	0.37047	0.31315	-3359.42613	-3359.48345
**^{5}Pr$_{Cl,1}$**	-3189.33326	-170.59344	-3359.92671	0.37271	0.31399	-3359.55399	-3359.61271
**^{5}Pr$_{OH,1}$**	-3189.30329	-170.54640	-3359.84969	0.37651	0.32018	-3359.47318	-3359.52951

Table S13 - Relative energies (in kcal/mol) of QM/MM optimized structures of the HctB model 1 reaction at UB3LYP/BS2 in Turbomole:Charmm. All data for small QM region A.

	RE_{QM}	RE_{QM+ZPE}	$RE_{QM+Gcorr}$
	kcal/mol	kcal/mol	kcal/mol
**$^{5}Re_{1}$**	0	0	0
**$^{5}TS_{HA,1}$**	27.39	23.62	24.27
**$^{5}Int_{1}$**	1.61	-3.10	-5.45
**$^{5}TS_{Cl,1}$**	-1.91	-6.01	-7.07
**$^{5}TS_{OH,1}$**	13.28	10.68	10.52
**$^{5}Pr_{Cl,1}$**	-36.40	-37.60	-38.63
**$^{5}Pr_{OH,1}$**	-17.59	-16.41	-15.94

Table S14 - Absolute energies (in au) of QM/MM optimized structures of HctB model 1 reaction at UB3LYP/BS1 in Turbomole:Charmm. All data for small region AB.

	E_{QM}	E_{MM}	$E_{QM/MM}$	ZPE	G_{corr}	$E_{QM/MM+ZPE}$	$E_{QM/MM+Gcorr}$
	(au)	(au)	(au)	(au)	(au)	(au)	(au)
**^{5}Re$_{1}$**	-5646.04830	-168.81966	-5814.86797	1.35772	1.22375	-5813.51024	-5813.64421
**^{5}TS$_{HA}$**	-5646.01178	-168.91296	-5814.92474	1.35300	1.22746	-5813.57174	-5813.69728
**^{5}Int$_{1}$**	-5646.03550	-168.92958	-5814.96508	1.35460	1.22317	-5813.61048	-5813.74191

Table S15 - Relative energies (in kcal/mol) of QM/MM optimized structures of the HctB model 1 reaction at UB3LYP/BS1 in Turbomole:Charmm. All data for small QM region AB.

	RE_{QM}	RE_{QM+ZPE}	$RE_{QM+Gcorr}$
	kcal/mol	kcal/mol	kcal/mol
**$^{5}Re_{1}$**	0.00	0.00	0.00
**$^{5}TS_{HA}$**	22.92	19.95	25.24
**$^{5}Int_{1}$**	8.03	6.07	7.67

Table S16 - Absolute energies (in au) of QM/MM optimized structures of HctB model 1 reaction at UB3LYP/BS2 in Turbomole:Charmm. All data for small region AB.

	E_{QM}	E_{MM}	$E_{QM/MM}$	ZPE	G_{corr}	$E_{QM/MM+ZPE}$	$E_{QM/MM+Gcorr}$
	(au)	(au)	(au)	(au)	(au)	(au)	(au)
**$^{5}Re_{1}$**	-5650.60936	-168.81966	-5819.42902	1.35772	1.22375	-5818.07130	-5818.20527
**$^{5}TS_{HA}$**	-5650.57502	-168.91296	-5819.48798	1.35300	1.22746	-5818.13497	-5818.26052
**$^{5}Int_{1}$**	-5650.60373	-168.92958	-5819.53330	1.35460	1.22317	-5818.17870	-5818.31013

Table S17 - Relative energies (in kcal/mol) of QM/MM optimized structures of the HctB model 1 reaction at UB3LYP/BS2 in Turbomole:Charmm. All data for small QM region AB.

	RE_{QM}	RE_{QM+ZPE}	$RE_{QM+Gcorr}$
	kcal/mol	kcal/mol	kcal/mol
**^{5}Re$_1$**	0.00	0.00	0.00
**^{5}TS$_{HA}$**	21.55	18.59	23.88
**^{5}Int$_1$**	3.53	1.57	3.17

Table S18 - Group spin densities of QM/MM optimized structures of the HctB model 1 reaction at UB3LYP/BS1 in Turbomole:Charmm. All data for small QM region A.

	His_{111}	His_{227}	SUB	SUC	Fe	O	Cl
**$^{1}Re_{1}$**	0.00	0.00	0.00	0.00	0.00	0.00	0.00
**$^{3}Re_{1}$**	0.08	0.00	0.00	-0.04	2.93	-0.93	-0.03
**$^{5}Re_{1}$**	0.06	-0.01	0.00	0.05	3.11	0.69	0.10
**$^{5}TS_{HA,1}$**	0.09	0.09	-0.33	0.07	4.14	-0.24	0.19
**$^{5}Int_{1}$**	0.08	0.11	-0.99	0.11	4.21	0.32	0.16
**$^{5}TS_{Cl,1}$**	0.06	0.08	-0.72	0.09	4.13	0.26	0.11
**$^{5}TS_{OH,1}$**	0.03	0.05	-0.22	0.05	3.93	0.16	0.01
**$^{5}Pr_{Cl,1}$**	0.02	0.03	0.00	0.03	3.82	0.10	0.00
**$^{5}Pr_{OH,1}$**	0.03	0.03	0.01	0.05	3.85	0.03	0.00

Table S19 - Group charges of QM/MM optimized structures of the HctB model 1 reaction at UB3LYP/BS1 in Turbomole:Charmm. All data for small QM region A.

	His_{111}	His_{227}	SUB	SUC	Fe	O	Cl
**$^{1}Re_{1}$**	0.43	0.00	0.00	-0.46	0.69	-0.43	-0.22
**$^{3}Re_{1}$**	0.25	0.29	-0.01	-0.59	0.75	-0.33	-0.36
**$^{5}Re_{1}$**	0.27	0.25	0.00	-0.57	0.75	-0.35	-0.35
**$^{5}TS_{HA,1}$**	0.29	0.28	0.16	-0.62	0.90	-0.55	-0.46
**$^{5}Int_{1}$**	0.29	0.31	0.29	-0.63	0.94	-0.73	-0.47
**$^{5}TS_{Cl,1}$**	0.25	0.27	0.50	-0.66	0.93	-0.77	-0.52
**$^{5}TS_{OH,1}$**	0.22	0.22	0.71	-0.69	0.86	-0.77	-0.55
**$^{5}Pr_{Cl,1}$**	0.16	0.20	0.54	-0.69	0.82	-0.86	-0.18
**$^{5}Pr_{OH,1}$**	-0.07	-0.30	-0.72	-0.79	-0.30	0.13	0.12

Table S20 - Group spin densities of QM/MM optimized structures of the HctB model 1 reaction at UB3LYP/BS1 in Turbomole:Charmm. All data for large QM region AB.

	2° Coord. Sphere	His_{111}	His_{227}	SUB	SUC	Fe	O	Cl
**$^{5}Re_{1}$**	0.00	0.06	-0.01	0.00	0.04	3.12	0.70	0.09
**$^{5}TS_{HA}$**	0.00	0.10	0.05	-0.33	0.05	4.03	-0.07	0.18
**$^{5}Int_{1}$**	-0.01	0.09	0.09	-0.99	0.08	4.19	0.33	0.21

Table S21 - Group charges of QM/MM optimized structures of the HctB model 1 reaction at UB3LYP/BS1 in Turbomole:Charmm. All data for large QM region AB.

	2° Coord. Sphere	His_{111}	His_{227}	SUB	SUC	Fe	O	Cl
**$^{5}Re_{1}$**	-1.74	0.26	0.17	-0.04	-0.47	0.75	-0.32	-0.36
**$^{5}TS_{HA}$**	-1.80	0.19	0.18	0.19	-0.58	0.88	-0.52	-0.38
**$^{5}Int_{1}$**	-1.86	0.19	0.15	0.32	-0.54	0.89	-0.67	-0.39

Table S22 – QM/MM relative energies for $^{5}TS_{HA}$ single point calculations on UB3LYP/BS1 optimized geometries for QM region A.

	B3LYP	PBE0	BP86
	kcal/mol	kcal/mol	kcal/mol
**$^{5}Re_{1}$**	0.00	0.00	0.00
**$^{5}TS_{HA}$**	21.55	18.63	34.69

Model 2

Table S23 - Absolute energies (in au) of QM/MM optimized structures of HctB model 2 reaction at UB3LYP/BS1 in Turbomole:Charmm. All data for small region A.

	E_{QM}	E_{MM}	$E_{QM/MM}$	ZPE	G_{corr}	$E_{QM/MM+ZPE}$	$E_{QM/MM+Gcorr}$
	(au)	(au)	(au)	(au)	(au)	(au)	(au)
**^{1}Re$_2$**	-3187.44124	-170.53911	-3357.98035	0.37560	0.31969	-3357.60475	-3357.66066
**^{3}Re$_2$**	-3187.48246	-170.54269	-3358.02515	0.37433	0.31413	-3357.65083	-3357.71102
**^{5}Re$_2$**	-3187.50342	-170.53922	-3358.04265	0.37526	0.31520	-3357.66739	-3357.72745
**^{5}TS$_{HA,2}$**	-3187.47186	-170.54572	-3358.01758	0.36810	0.30632	-3357.64948	-3357.71126
**^{5}Int$_2$**	-3187.51514	-170.57260	-3358.08774	0.36885	0.30713	-3357.71889	-3357.78061
**^{5}TS$_{Cl,2}$**	-3187.51675	-170.55789	-3358.07464	0.36915	0.30623	-3357.70549	-3357.76842
**^{5}TS$_{OH,2}$**	-3187.49036	-170.56162	-3358.05198	0.36865	0.31034	-3357.68333	-3357.74164
**^{5}Pr$_{Cl,2}$**	-3187.55854	-170.57298	-3358.13151	0.37316	0.31662	-3357.75835	-3357.81490
**^{5}Pr$_{OH,2}$**	-3187.59089	-170.60714	-3358.19802	0.37654	0.31439	-3357.82148	-3357.88363

Table S24 - Relative energies (in kcal/mol) of QM/MM optimized structures of the HctB model 2 reaction at UB3LYP/BS1 in Turbomole:Charmm. All data for small QM region A.

	RE_{QM}	RE_{QM+ZPE}	$RE_{QM+Gcorr}$
	kcal/mol	kcal/mol	kcal/mol
^{1}**Re$_2$**	39.02	39.24	41.84
^{3}**Re$_2$**	13.15	12.57	12.48
^{5}**Re$_2$**	0.00	0.00	0.00
^{5}**TS$_{HA,2}$**	19.81	15.31	14.24
^{5}**Int$_2$**	-7.35	-11.37	-12.42
^{5}**TS$_{Cl,2}$**	-8.36	-12.19	-13.99
^{5}**TS$_{OH,2}$**	8.20	4.05	5.15
^{5}**Pr$_{Cl,2}$**	-34.58	-35.90	-33.70
^{5}**Pr$_{OH,2}$**	-54.89	-54.08	-55.39

Table S25 - Absolute energies (in au) of QM/MM optimized structures of HctB model 2 reaction at UB3LYP/BS2 in Turbomole:Charmm. All data for small region A.

	E_{QM}	E_{MM}	$E_{QM/MM}$	ZPE	G_{corr}	$E_{QM/MM+ZPE}$	$E_{QM/MM+Gcorr}$
	(au)	(au)	(au)	(au)	(au)	(au)	(au)
^{5}**Re$_{2}$**	-3189.24846	-170.53922	-3359.78768	0.37526	0.31520	-3359.41242	-3359.47248
^{5}**TS$_{HA,2}$**	-3189.21591	-170.54572	-3359.76163	0.36810	0.30632	-3359.39353	-3359.45531
^{5}**Int$_{2}$**	-3189.26453	-170.57260	-3359.83712	0.36885	0.30713	-3359.46828	-3359.53000
^{5}**TS$_{Cl,2}$**	-3189.26713	-170.55789	-3359.82503	0.36915	0.30623	-3359.45588	-3359.51880
^{5}**TS$_{OH,2}$**	-3189.23911	-170.56162	-3359.80073	0.36865	0.31034	-3359.43208	-3359.49039
^{5}**Pr$_{Cl,2}$**	-3189.31009	-170.57298	-3359.88306	0.37316	0.31662	-3359.50990	-3359.56645
^{5}**Pr$_{OH,2}$**	-3189.33490	-170.60714	-3359.94204	0.37654	0.31439	-3359.56549	-3359.62764

Table S26 - Relative energies (in kcal/mol) of QM/MM optimized structures of the HctB model 2 reaction at UB3LYP/BS2 in Turbomole:Charmm. All data for small QM region A.

	RE_{QM}	RE_{QM+ZPE}	$RE_{QM+Gcorr}$
	kcal/mol	kcal/mol	kcal/mol
**^{5}Re$_2$**	0.00	0.00	0.00
**^{5}TS$_{HA,2}$**	20.42	15.93	14.85
**^{5}Int$_2$**	-10.08	-14.11	-15.15
**^{5}TS$_{Cl,2}$**	-11.72	-15.55	-17.35
**^{5}TS$_{OH,2}$**	5.86	1.72	2.82
**^{5}Pr$_{Cl,2}$**	-38.67	-39.99	-37.79
**^{5}Pr$_{OH,2}$**	-54.24	-53.44	-54.75

Table S27 - Absolute energies (in au) of QM/MM optimized structures of HctB model 2 reaction at UB3LYP/BS1 in Turbomole:Charmm. All data for small region AB.

	E_{QM}	E_{MM}	$E_{QM/MM}$	ZPE	G_{corr}	$E_{QM/MM+ZPE}$	$E_{QM/MM+Gcorr}$
	(au)	(au)	(au)	(au)	(au)	(au)	(au)
**^{5}Re$_2$**	-5015.59732	-169.35082	-5184.94813	1.22323	1.09238	-5183.72491	-5183.85576
**^{5}TS$_{HA,2}$**	-5015.57155	-169.39457	-5184.96612	1.21555	1.09178	-5183.75057	-5183.87434
**^{5}Int$_2$**	-5015.60219	-169.39801	-5185.00020	1.21591	1.09066	-5183.78429	-5183.90954
**^{5}Pr$_{OH,2}$**	-5015.67695	-169.40886	-5185.08581	1.22628	1.09854	-5183.85953	-5183.98727
**^{5}Pr$_{Cl,2}$**	-5015.63632	-169.41471	-5185.05103	1.22462	1.09664	-5183.82640	-5183.95439

Table S28 - Relative energies (in kcal/mol) of QM/MM optimized structures of the HctB model 2 reaction at UB3LYP/BS1 in Turbomole:Charmm. All data for small QM region AB.

	RE_{QM}	RE_{QM+ZPE}	$RE_{QM+Gcorr}$
	kcal/mol	kcal/mol	kcal/mol
**^{5}Re$_2$**	0.00	0.00	0.00
**^{5}TS$_{HA,2}$**	16.17	11.35	15.79
**^{5}Int$_2$**	-3.06	-7.65	-4.13
**^{5}Pr$_{OH,2}$**	-49.97	-48.05	-46.10
**^{5}Pr$_{Cl,2}$**	-24.48	-23.60	-21.80

Table S29 - Absolute energies (in au) of QM/MM optimized structures of HctB model 2 reaction at UB3LYP/BS2 in Turbomole:Charmm. All data for small region AB.

	E_{QM}	E_{MM}	$E_{QM/MM}$	ZPE	G_{corr}	$E_{QM/MM+ZPE}$	$E_{QM/MM+Gcorr}$
	(au)	(au)	(au)	(au)	(au)	(au)	(au)
**$^5Re_2$**	-5019.69157	-169.35082	-5189.04239	1.22323	1.09238	-5187.81916	-5187.95002
**$^5TS_{HA,2}$**	-5019.66543	-169.39457	-5189.06000	1.21555	1.09178	-5187.84446	-5187.96823
**$^5Int_2$**	-5019.67899	-169.39801	-5189.07701	1.21591	1.09066	-5187.86109	-5187.98634
**$^5Pr_{OH,2}$**	-5019.77402	-169.40886	-5189.18288	1.22628	1.09854	-5187.95660	-5188.08434
**$^5Pr_{Cl,2}$**	-5019.73855	-169.41471	-5189.15325	1.22462	1.09664	-5187.92863	-5188.05662

Table S30 - Relative energies (in kcal/mol) of QM/MM optimized structures of the HctB model 2 reaction at UB3LYP/BS2 in Turbomole:Charmm. All data for small QM region AB.

	RE_{QM}	RE_{QM+ZPE}	$RE_{QM+Gcorr}$
	kcal/mol	kcal/mol	kcal/mol
$^{5}\mathbf{Re_{2}}$	0.00	0.00	0.00
$^{5}\mathbf{TS_{HA,2}}$	16.41	11.59	16.03
$^{5}\mathbf{Int_{2}}$	7.90	3.31	6.82
$^{5}\mathbf{Pr_{OH,2}}$	-51.73	-49.82	-47.87
$^{5}\mathbf{Pr_{Cl,2}}$	-29.48	-28.60	-26.80

Table S31 - Group spin densities of QM/MM optimized structures of the HctB model 2 reaction at UB3LYP/BS1 in Turbomole:Charmm. All data for small QM region A.

	His_{111}	His_{227}	SUB	SUC	Fe	O	Cl
**$^{1}Re_{2}$**	0.00	0.00	0.00	0.00	0.00	0.00	0.00
**$^{3}Re_{2}$**	0.05	-0.02	0.00	0.06	2.83	-0.91	-0.01
**$^{5}Re_{2}$**	0.04	-0.02	0.00	0.08	3.12	0.63	0.14
**$^{5}TS_{HA,2}$**	0.00	0.00	0.03	0.00	0.00	0.00	0.00
**$^{5}Int_{2}$**	0.10	0.05	-0.99	0.14	4.20	0.28	0.22
**$^{5}TS_{Cl,2}$**	0.05	0.02	-0.42	0.07	3.98	0.16	0.14
**$^{5}TS_{OH,2}$**	0.06	0.03	-0.63	0.07	4.05	0.30	0.12
**$^{5}Pr_{Cl,2}$**	0.03	0.01	0.00	0.05	3.79	0.10	0.00
**$^{5}Pr_{OH,2}$**	0.03	0.03	0.00	0.05	3.81	0.00	0.07

Table S32 - Group charges of QM/MM optimized structures of the HctB model 2 reaction at UB3LYP/BS1 in Turbomole:Charmm. All data for small QM region A.

	His_{111}	His_{227}	SUB	SUC	Fe	O	Cl
**$^{1}Re_{2}$**	0.40	0.20	-0.01	-0.49	0.66	-0.47	-0.28
**$^{3}Re_{2}$**	0.29	0.25	-0.03	-0.53	0.70	-0.37	-0.35
**$^{5}Re_{2}$**	0.33	0.25	-0.02	-0.55	0.76	-0.41	-0.35
**$^{5}TS_{HA,2}$**	-0.66	-0.25	-0.09	-0.14	0.11	0.13	0.09
**$^{5}Int_{2}$**	0.29	0.20	0.31	-0.50	0.83	-0.74	-0.39
**$^{5}TS_{Cl,2}$**	0.21	0.16	0.65	-0.62	0.81	-0.82	-0.40
**$^{5}TS_{OH,2}$**	0.22	0.17	0.57	-0.63	0.82	-0.66	-0.50
**$^{5}Pr_{Cl,2}$**	0.18	0.16	0.52	-0.62	0.77	-0.85	-0.16
**$^{5}Pr_{OH,2}$**	0.19	0.19	0.51	-0.59	0.76	-0.58	-0.48

Table S33 - Group spin densities of QM/MM optimized structures of the HctB model 2 reaction at UB3LYP/BS1 in Turbomole:Charmm. All data for large QM region AB.

	2° Coord. Sphere	His_{111}	His_{227}	SUB	SUC	Fe	O	Cl
**$^5Re_2$**	0.00	0.04	-0.02	0.00	0.09	3.11	0.65	0.12
**$^5TS_{HA,2}$**	0.00	0.06	0.04	-0.40	0.10	3.97	0.12	0.11
**$^5Int_2$**	-0.02	0.11	0.05	-0.97	0.14	4.20	0.28	0.20
**$^5Pr_{OH,2}$**	0.00	0.03	0.02	0.01	0.05	3.80	0.10	0.00
**$^5Pr_{Cl,2}$**	0.00	0.03	0.04	0.00	0.05	3.82	0.00	0.06

Table S34 - Group charges of QM/MM optimized structures of the HctB model 2 reaction at UB3LYP/BS1 in Turbomole:Charmm. All data for large QM region AB.

	2o Coord. Sphere	His_{111}	His_{227}	SUB	SUC	Fe	O	Cl
**$^5Re_2$**	-1.13	0.36	0.23	-0.02	-0.50	0.73	-0.36	-0.33
**$^5TS_{HA,2}$**	-1.16	0.29	0.21	0.23	-0.55	0.88	-0.47	-0.45
**$^5Int_2$**	-1.12	0.33	0.20	0.34	-0.51	0.83	-0.67	-0.36
**$^5Pr_{OH,2}$**	-1.10	0.22	0.18	0.49	-0.62	0.77	-0.72	-0.20
**$^5Pr_{Cl,2}$**	-1.14	0.24	0.18	0.46	-0.56	0.75	-0.50	-0.44

Model 3

Table S35 - Absolute energies (in au) of QM/MM optimized structures of HctB model 3 reaction at UB3LYP/BS1 in Turbomole:Charmm. All data for small region A.

	E_{QM}	E_{MM}	$E_{QM/MM}$	ZPE	G_{corr}	$E_{QM/MM+ZPE}$	$E_{QM/MM+Gcorr}$
	(au)	(au)	(au)	(au)	(au)	(au)	(au)
$\mathbf{^{1}Re_{3}}$	-3187.47992	-170.43531	-3357.91522	0.42860	0.38712	-3357.48662	-3357.52810
$\mathbf{^{3}Re_{3}}$	-3187.53771	-170.42342	-3357.96113	0.37402	0.31283	-3357.58712	-3357.64830
$\mathbf{^{5}Re_{3}}$	-3187.55940	-170.44393	-3358.00334	0.37544	0.31127	-3357.62790	-3357.69207
$\mathbf{^{5}TS_{HA,3}}$	-3187.48460	-170.43750	-3357.92210	0.36746	0.30622	-3357.55464	-3357.61588
$\mathbf{^{5}Int_{3}}$	-3187.51106	-170.49084	-3358.00190	0.36941	0.30475	-3357.63249	-3357.69715
$\mathbf{^{5}TS_{Cl,3}}$	-3187.47774	-170.46614	-3357.94388	0.37226	0.31324	-3357.57162	-3357.63064
$\mathbf{^{5}TS_{OH,3}}$	-3187.52875	-170.44219	-3357.97094	0.36936	0.30771	-3357.60158	-3357.66322
$\mathbf{^{5}Pr_{Cl,3}}$	-3187.53931	-170.47115	-3358.01046	0.37401	0.31620	-3357.63645	-3357.69426
$\mathbf{^{5}Pr_{OH,3}}$	-3187.59227	-170.51166	-3358.10393	0.37536	0.31619	-3357.72857	-3357.78774

Table S36 - Relative energies (in kcal/mol) of QM/MM optimized structures of the HctB model 3 reaction at UB3LYP/BS1 in Turbomole:Charmm. All data for small QM region A.

	RE_{QM}	RE_{QM+ZPE}	$RE_{QM+Gcorr}$
	kcal/mol	kcal/mol	kcal/mol
**$^{1}Re_{3}$**	49.88	83.24	97.48
**$^{3}Re_{3}$**	13.61	12.72	14.60
**$^{5}Re_{3}$**	0.00	0.00	0.00
**$^{5}TS_{HA,3}$**	46.94	41.93	43.77
**$^{5}Int_{3}$**	30.34	26.55	26.25
**$^{5}TS_{Cl,3}$**	51.24	49.25	52.48
**$^{5}TS_{OH,3}$**	19.24	15.42	17.01
**$^{5}Pr_{Cl,3}$**	12.61	11.71	15.71
**$^{5}Pr_{OH,3}$**	-20.63	-20.67	-17.54

Table S37 - Absolute energies (in au) of QM/MM optimized structures of HctB model 3 reaction at UB3LYP/BS2 in Turbomole:Charmm. All data for small region A.

	E_{QM}	E_{MM}	$E_{QM/MM}$	ZPE	G_{corr}	$E_{QM/MM+ZPE}$	$E_{QM/MM+Gcorr}$
	(au)	(au)	(au)	(au)	(au)	(au)	(au)
**$^5Re_3$**	-3189.30723	-170.44393	-3359.75117	0.37544	0.31127	-3359.37572	-3359.43990
**$^5TS_{HA,3}$**	-3189.22830	-170.43750	-3359.66580	0.36746	0.30622	-3359.29834	-3359.35958
**$^5Int_3$**	-3189.24239	-170.49084	-3359.73323	0.36941	0.30475	-3359.36382	-3359.42848
**$^5TS_{Cl,3}$**	-3189.22374	-170.46614	-3359.68988	0.37226	0.31324	-3359.31762	-3359.37664
**$^5TS_{OH,3}$**	-3189.27670	-170.44219	-3359.71889	0.36936	0.30771	-3359.34953	-3359.41118
**$^5Pr_{Cl,3}$**	-3189.28913	-170.47115	-3359.76028	0.37401	0.31620	-3359.38628	-3359.44408
**$^5Pr_{OH,3}$**	-3189.34154	-170.51166	-3359.85319	0.37536	0.31619	-3359.47783	-3359.53701

Table S38 - Relative energies (in kcal/mol) of QM/MM optimized structures of the HctB model 3 reaction at UB3LYP/BS2 in Turbomole:Charmm. All data for small QM region A.

	RE_{QM}	RE_{QM+ZPE}	$RE_{QM+Gcorr}$
	kcal/mol	kcal/mol	kcal/mol
**$^{5}Re_{3}$**	0.00	0.00	0.00
**$^{5}TS_{HA,3}$**	49.53	44.52	46.36
**$^{5}Int_{3}$**	40.69	36.91	36.60
**$^{5}TS_{Cl,3}$**	52.39	50.39	53.63
**$^{5}TS_{OH,3}$**	19.16	15.34	16.93
**$^{5}Pr_{Cl,3}$**	11.36	10.46	14.45
**$^{5}Pr_{OH,3}$**	-21.53	-21.57	-18.44

Table S39 - Absolute energies (in au) of QM/MM optimized structures of HctB model 3 reaction at UB3LYP/BS1 in Turbomole:Charmm. All data for small region AB.

	E_{QM}	E_{MM}	$E_{QM/MM}$	ZPE	G_{corr}	$E_{QM/MM+ZPE}$	$E_{QM/MM+Gcorr}$
	(au)	(au)	(au)	(au)	(au)	(au)	(au)
**^{5}Re$_3$**	-5201.21542	-169.47945	-5370.69487	1.24532	1.11344	-5369.44955	-5369.58143
**^{5}Int$_3$**	-5201.24328	-169.43430	-5370.67759	1.24605	1.12242	-5369.43154	-5369.55517
**^{5}Pr$_{OH,3}$**	-5201.30921	-169.44598	-5370.75519	1.25435	1.13342	-5369.50084	-5369.62178
**^{5}Pr$_{Cl,3}$**	-5201.28111	-169.40671	-5370.68782	1.25124	1.13155	-5369.43658	-5369.55626

Table S40 - Relative energies (in kcal/mol) of QM/MM optimized structures of the HctB model 3 reaction at UB3LYP/BS1 in Turbomole:Charmm. All data for small QM region AB.

	RE_{QM}	RE_{QM+ZPE}	$RE_{QM+Gcorr}$
	kcal/mol	kcal/mol	kcal/mol
**^{5}Re$_3$**	0.00	0.00	0.00
**^{5}Int$_3$**	-17.48	-17.03	-11.85
**^{5}Pr$_{OH,3}$**	-58.85	-53.19	-46.32
**^{5}Pr$_{Cl,3}$**	-41.22	-37.50	-29.85

Table S41 - Absolute energies (in au) of QM/MM optimized structures of HctB model 3 reaction at UB3LYP/BS2 in Turbomole:Charmm. All data for small region AB.

	E_{QM}	E_{MM}	$E_{QM/MM}$	ZPE	G_{corr}	$E_{QM/MM+ZPE}$	$E_{QM/MM+Gcorr}$
	(au)	(au)	(au)	(au)	(au)	(au)	(au)
**^{5}Re$_3$**	-5205.48258	-169.47945	-5374.96203	1.24532	1.11344	-5373.71671	-5373.84859
**^{5}Int$_3$**	-5205.51108	-169.43430	-5374.94538	1.24605	1.12242	-5373.69934	-5373.82296
**^{5}Pr$_{OH,3}$**	-5205.57436	-169.44598	-5375.02035	1.25435	1.13342	-5373.76600	-5373.88693
**^{5}Pr$_{Cl,3}$**	-5205.55053	-169.40671	-5374.95724	1.25124	1.13155	-5373.70600	-5373.82569

Table S42 - Relative energies (in kcal/mol) of QM/MM optimized structures of the HctB model 3 reaction at UB3LYP/BS2 in Turbomole:Charmm. All data for small QM region AB.

	RE_{QM}	RE_{QM+ZPE}	$RE_{QM+Gcorr}$
	kcal/mol	kcal/mol	kcal/mol
**^{5}Re$_3$**	0.00	0.00	0.00
**^{5}Int$_3$**	-17.88	-17.43	-12.25
**^{5}Pr$_{OH,3}$**	-57.60	-51.93	-45.06
**^{5}Pr$_{Cl,3}$**	-42.64	-38.92	-31.27

Table S43 - Group spin densities of QM/MM optimized structures of the HctB model 3 reaction at UB3LYP/BS1 in Turbomole:Charmm. All data for small QM region A.

	His_{111}	His_{227}	SUB	SUC	Fe	O	Cl
**$^{1}Re_{3}$**	0.00	0.00	0.00	0.00	0.00	0.00	0.00
**$^{3}Re_{3}$**	0.03	-0.02	0.00	0.05	2.84	-0.88	-0.02
**$^{5}Re_{3}$**	0.04	-0.03	0.00	0.11	3.14	0.64	0.10
**$^{5}TS_{HA,3}$**	0.08	0.03	-0.47	0.13	4.03	0.01	0.19
**$^{5}Int_{3}$**	0.09	0.06	-1.00	0.14	4.21	0.29	0.20
**$^{5}TS_{Cl,3}$**	0.03	0.01	0.01	0.04	3.80	0.10	0.01
**$^{5}TS_{OH,3}$**	0.04	0.03	-0.34	0.07	3.97	0.14	0.09
**$^{5}Pr_{Cl,3}$**	0.03	0.01	0.01	0.06	3.80	0.09	0.00
**$^{5}Pr_{OH,3}$**	0.03	0.02	0.00	0.05	3.82	0.00	0.07

Table S44 - Group charges of QM/MM optimized structures of the HctB model 3 reaction at UB3LYP/BS1 in Turbomole:Charmm. All data for small QM region A.

	His_{111}	His_{227}	SUB	SUC	Fe	O	Cl
**$^{1}Re_{3}$**	0.39	0.24	0.00	-0.50	0.67	-0.45	-0.34
**$^{3}Re_{3}$**	0.30	0.30	0.00	-0.57	0.74	-0.37	-0.40
**$^{5}Re_{3}$**	0.30	0.28	0.00	-0.49	0.78	-0.48	-0.39
**$^{5}TS_{HA,3}$**	0.27	0.13	0.24	-0.47	0.86	-0.65	-0.38
**$^{5}Int_{3}$**	0.26	0.22	0.31	-0.49	0.86	-0.74	-0.42
**$^{5}TS_{Cl,3}$**	0.14	0.20	0.66	-0.67	0.80	-0.73	-0.39
**$^{5}TS_{OH,3}$**	0.19	0.15	0.70	-0.56	0.80	-0.75	-0.52
**$^{5}Pr_{Cl,3}$**	0.16	0.19	0.49	-0.59	0.80	-0.86	-0.18
**$^{5}Pr_{OH,3}$**	0.20	0.21	0.53	-0.60	0.77	-0.59	-0.51

Table S45 - Group spin densities of QM/MM optimized structures of the HctB model 3 reaction at UB3LYP/BS1 in Turbomole:Charmm. All data for large QM region AB.

	2° Coord. Sphere	His_{111}	His_{227}	SUB	SUC	Fe	O	Cl
^{5}**Re$_3$**	0.00	0.04	-0.02	0.00	0.10	3.16	0.61	0.10
^{5}**Int$_3$**	0.00	0.10	0.06	-0.96	0.15	4.21	0.29	0.15
^{5}**Pr$_{OH,3}$**	0.00	0.04	0.02	0.01	0.04	3.82	0.06	0.00
^{5}**Pr$_{Cl,3}$**	0.00	0.03	0.04	0.00	0.06	3.82	0.00	0.05

Table S46 - Group charges of QM/MM optimized structures of the HctB model 3 reaction at UB3LYP/BS1 in Turbomole:Charmm. All data for large QM region AB.

	2° Coord. Sphere	His_{111}	His_{227}	SUB	SUC	Fe	O	Cl
^{5}**Re$_3$**	-0.30	0.30	0.31	0.01	-0.41	0.77	-0.43	-0.33
^{5}**Int$_3$**	-0.26	0.23	0.23	0.28	-0.41	0.87	-0.67	-0.39
^{5}**Pr$_{OH,3}$**	-0.32	0.20	0.21	0.49	-0.56	0.77	-0.74	-0.17
^{5}**Pr$_{Cl,3}$**	-0.29	0.19	0.22	0.47	-0.50	0.75	-0.52	-0.43

Model 4

Table S47 - Absolute energies (in au) of QM/MM optimized structures of HctB model 4 reaction at UB3LYP/BS1 in Turbomole:Charmm. All data for small region A.

	E_{QM}	E_{MM}	$E_{QM/MM}$	ZPE	G_{corr}	$E_{QM/MM+ZPE}$	$E_{QM/MM+Gcorr}$
	(au)	(au)	(au)	(au)	(au)	(au)	(au)
**^{1}Re$_{4}$**	-3187.46563	-170.30746	-3357.77309	0.37575	0.32004	-3357.39733	-3357.45304
**^{3}Re$_{4}$**	-3187.46928	-170.31879	-3357.78806	0.37405	0.31342	-3357.41402	-3357.47464
**^{5}Re$_{4}$**	-3187.50026	-170.29865	-3357.79891	0.37405	0.31342	-3357.42486	-3357.48549
**^{5}TS$_{HA,4}$**	-3187.46650	-170.32230	-3357.78880	0.36969	0.31232	-3357.41911	-3357.47647
**^{5}Int$_{4}$**	-3187.49525	-170.33789	-3357.83314	0.36872	0.30641	-3357.46442	-3357.52674
**^{5}TS$_{Cl,4}$**	-3187.49337	-170.35049	-3357.84385	0.36880	0.30646	-3357.47506	-3357.53739
**^{5}TS$_{OH,4}$**	-3187.49261	-170.35401	-3357.84662	0.36851	0.30828	-3357.47811	-3357.53834
**^{5}Pr$_{Cl,4}$**	-3187.53516	-170.36160	-3357.89676	0.37226	0.30831	-3357.52450	-3357.58846
**^{5}Pr$_{OH,4}$**	-3187.58009	-170.37597	-3357.95606	0.37591	0.31968	-3357.58015	-3357.63638

Table S48 - Relative energies (in kcal/mol) of QM/MM optimized structures of the HctB model 4 reaction at UB3LYP/BS1 in Turbomole:Charmm. All data for small QM region A.

	RE_{QM}	RE_{QM+ZPE}	$RE_{QM+Gcorr}$
	kcal/mol	kcal/mol	kcal/mol
**$^{1}Re_{4}$**	21.73	22.80	25.89
**$^{3}Re_{4}$**	19.44	19.44	19.44
**$^{5}Re_{4}$**	0.00	0.00	0.00
**$^{5}TS_{HA,4}$**	21.19	18.45	20.50
**$^{5}Int_{4}$**	3.14	-0.20	-1.26
**$^{5}TS_{Cl,4}$**	4.33	1.03	-0.04
**$^{5}TS_{OH,4}$**	4.80	1.33	1.57
**$^{5}Pr_{Cl,4}$**	-21.90	-23.02	-25.11
**$^{5}Pr_{OH,4}$**	-50.09	-48.92	-46.17

Table S49 - Absolute energies (in au) of QM/MM optimized structures of HctB model 4 reaction at UB3LYP/BS2 in Turbomole:Charmm. All data for small region A.

	E_{QM}	E_{MM}	$E_{QM/MM}$	ZPE	G_{corr}	$E_{QM/MM+ZPE}$	$E_{QM/MM+Gcorr}$
	(au)	(au)	(au)	(au)	(au)	(au)	(au)
**^{5}Re$_{4}$**	-3189.24353	-170.29865	-3359.54217	0.37405	0.31342	-3359.16813	-3359.22875
**^{5}TS$_{HA,4}$**	-3189.20645	-170.32230	-3359.52875	0.36969	0.31232	-3359.15906	-3359.21643
**^{5}Int$_{4}$**	-3189.24330	-170.33789	-3359.58119	0.36872	0.30641	-3359.21247	-3359.27479
**^{5}TS$_{Cl,4}$**	-3189.23983	-170.35049	-3359.59032	0.36880	0.30646	-3359.22152	-3359.28385
**^{5}TS$_{OH,4}$**	-3189.23815	-170.35401	-3359.59216	0.36851	0.30828	-3359.22365	-3359.28388
**^{5}Pr$_{Cl,4}$**	-3189.28291	-170.36160	-3359.64452	0.37226	0.30831	-3359.27225	-3359.33621
**^{5}Pr$_{OH,4}$**	-3189.32617	-170.37597	-3359.70214	0.37591	0.31968	-3359.32623	-3359.38246

Table S50 - Relative energies (in kcal/mol) of QM/MM optimized structures of the HctB model 4 reaction at UB3LYP/BS2 in Turbomole:Charmm. All data for small QM region A.

	RE_{QM}	RE_{QM+ZPE}	$RE_{QM+Gcorr}$
	kcal/mol	kcal/mol	kcal/mol
$^{5}\mathbf{Re_4}$	0.00	0.00	0.00
$^{5}\mathbf{TS_{HA,4}}$	23.27	20.53	22.58
$^{5}\mathbf{Int_4}$	0.14	-3.20	-4.26
$^{5}\mathbf{TS_{Cl,4}}$	2.32	-0.97	-2.05
$^{5}\mathbf{TS_{OH,4}}$	3.37	-0.10	0.15
$^{5}\mathbf{Pr_{Cl,4}}$	-24.72	-25.83	-27.93
$^{5}\mathbf{Pr_{OH,4}}$	-51.86	-50.69	-47.94

Table S51 - Absolute energies (in au) of QM/MM optimized structures of HctB model 4 reaction at UB3LYP/BS1 in Turbomole:Charmm. All data for small region AB.

	E_{QM}	E_{MM}	$E_{QM/MM}$	ZPE	G_{corr}	$E_{QM/MM+ZPE}$	$E_{QM/MM+Gcorr}$
	(au)	(au)	(au)	(au)	(au)	(au)	(au)
**$^5Re_4$**	-4480.09035	-169.77748	-4649.86782	1.06795	0.95615	-4648.79988	-4648.91168
**$^5TS_{HA,4}$**	-4480.06447	-169.78653	-4649.85100	1.06575	0.95384	-4648.78525	-4648.89716
**$^5Int_4$**	-4480.09070	-169.80088	-4649.89158	1.06424	0.95087	-4648.82734	-4648.94071
**$^5Pr_{Cl,4}$**	-4480.17950	-169.81431	-4649.99381	1.07150	0.96116	-4648.92231	-4649.03264
**$^5Pr_{OH,4}$**	-4480.14984	-169.84821	-4649.99805	1.06891	0.96100	-4648.92914	-4649.03705

Table S52 - Relative energies (in kcal/mol) of QM/MM optimized structures of the HctB model 4 reaction at UB3LYP/BS1 in Turbomole:Charmm. All data for small QM region AB.

	RE_{QM}	RE_{QM+ZPE}	$RE_{QM+Gcorr}$
	kcal/mol	kcal/mol	kcal/mol
**$^5Re_4$**	0.00	0.00	0.00
**$^5TS_{HA,4}$**	16.24	14.86	14.79
**$^5Int_4$**	-0.22	-2.55	-3.53
**$^5Pr_{Cl,4}$**	-55.94	-53.72	-52.80
**$^5Pr_{OH,4}$**	-37.33	-36.73	-34.29

Table S53 - Absolute energies (in au) of QM/MM optimized structures of HctB model 4 reaction at UB3LYP/BS2 in Turbomole:Charmm. All data for small region AB.

	E_{QM}	E_{MM}	$E_{QM/MM}$	ZPE	G_{corr}	$E_{QM/MM+ZPE}$	$E_{QM/MM+Gcorr}$
	(au)	(au)	(au)	(au)	(au)	(au)	(au)
**^{5}Re$_4$**	-4483.51410	-169.77748	-4653.29158	1.06795	0.95615	-4652.22363	-4652.33543
**^{5}TS$_{HA,4}$**	-4483.48211	-169.78653	-4653.26864	1.06575	0.95384	-4652.20289	-4652.31480
**^{5}Int$_4$**	-4483.51522	-169.80088	-4653.31610	1.06424	0.95087	-4652.25185	-4652.36522
**^{5}Pr$_{Cl,4}$**	-4483.60778	-169.81431	-4653.42209	1.07150	0.96116	-4652.35059	-4652.46093
**^{5}Pr$_{OH,4}$**	-4483.57859	-169.84821	-4653.42680	1.06891	0.96100	-4652.35789	-4652.46580

Table S54 - Relative energies (in kcal/mol) of QM/MM optimized structures of the HctB model 4 reaction at UB3LYP/BS2 in Turbomole:Charmm. All data for small QM region AB.

	RE_{QM}	RE_{QM+ZPE}	$RE_{QM+Gcorr}$
	kcal/mol	kcal/mol	kcal/mol
^{5}**Re$_{4}$**	0.00	0.00	0.00
^{5}**TS$_{HA,4}$**	20.08	18.70	18.63
^{5}**Int$_{4}$**	-0.70	-3.02	-4.01
^{5}**Pr$_{Cl,4}$**	-58.78	-56.56	-55.64
^{5}**Pr$_{OH,4}$**	-40.47	-39.87	-37.42

Table S55 - Group spin densities of QM/MM optimized structures of the HctB model 4 reaction at UB3LYP/BS1 in Turbomole:Charmm. All data for small QM region A.

	His_{111}	His_{227}	SUB	SUC	Fe	O	Cl
**$^{1}Re_{4}$**	0.02	0.00	0.00	0.02	-0.41	0.36	0.02
**$^{3}Re_{4}$**	0.07	-0.01	0.00	0.04	2.90	-0.95	-0.05
**$^{5}Re_{4}$**	0.05	-0.02	0.00	0.10	3.15	0.63	0.09
**$^{5}TS_{HA,4}$**	0.05	0.04	-0.27	0.14	4.01	-0.14	0.18
**$^{5}Int_{4}$**	0.09	0.05	-0.99	0.15	4.21	0.27	0.22
**$^{5}TS_{Cl,4}$**	0.05	0.03	-0.60	0.10	4.07	0.17	0.18
**$^{5}TS_{OH,4}$**	0.04	0.03	-0.60	0.09	4.05	0.27	0.11
**$^{5}Pr_{Cl,4}$**	0.03	0.02	0.00	0.06	3.80	0.09	0.00
**$^{5}Pr_{OH,4}$**	0.02	0.03	0.00	0.05	3.83	0.00	0.06

Table S56 - Group charges of QM/MM optimized structures of the HctB model 4 reaction at UB3LYP/BS1 in Turbomole:Charmm. All data for small QM region A.

	His_{111}	His_{227}	SUB	SUC	Fe	O	Cl
$^{1}\mathbf{Re_4}$	0.35	0.25	-0.02	-0.36	0.60	-0.49	-0.33
$^{3}\mathbf{Re_4}$	0.25	0.28	-0.02	-0.45	0.70	-0.41	-0.34
$^{5}\mathbf{Re_4}$	0.28	0.27	-0.02	-0.49	0.75	-0.45	-0.34
$^{5}\mathbf{TS_{HA,4}}$	0.23	0.23	0.12	-0.45	0.85	-0.52	-0.46
$^{5}\mathbf{Int_4}$	0.27	0.21	0.31	-0.47	0.82	-0.75	-0.40
$^{5}\mathbf{TS_{Cl,4}}$	0.20	0.18	0.54	-0.55	0.81	-0.80	-0.39
$^{5}\mathbf{TS_{OH,4}}$	0.17	0.20	0.57	-0.56	0.82	-0.69	-0.51
$^{5}\mathbf{Pr_{Cl,4}}$	0.16	0.18	0.51	-0.60	0.76	-0.83	-0.19
$^{5}\mathbf{Pr_{OH,4}}$	0.21	0.21	0.48	-0.60	0.77	-0.58	-0.50

Table S57 - Group spin densities of QM/MM optimized structures of the HctB model 4 reaction at UB3LYP/BS1 in Turbomole:Charmm. All data for large QM region AB.

	2° Coord. Sphere	His_{111}	His_{227}	SUB	SUC	Fe	O	Cl
**$^5Re_4$**	0.00	0.05	-0.02	0.00	0.13	3.22	0.52	0.09
**$^5TS_{HA,4}$**	0.00	0.08	0.05	-0.31	0.15	4.10	-0.27	0.17
**$^5Int_4$**	-0.01	0.09	0.07	-0.87	0.16	4.19	0.21	0.15
**$^5Pr_{Cl,4}$**	0.00	0.03	0.02	0.00	0.05	3.82	0.07	0.00
**$^5Pr_{OH,4}$**	0.00	0.03	0.04	0.00	0.05	3.83	0.00	0.05

Table S58 - Group charges of QM/MM optimized structures of the HctB model 4 reaction at UB3LYP/BS1 in Turbomole:Charmm. All data for large QM region AB.

	2° Coord. Sphere	His_{111}	His_{227}	SUB	SUC	Fe	O	Cl
**$^5Re_4$**	0.91	0.30	0.29	-0.06	-0.40	0.75	-0.44	-0.31
**$^5TS_{HA,4}$**	0.95	0.26	0.22	0.11	-0.41	0.86	-0.49	-0.39
**$^5Int_4$**	0.94	0.26	0.23	0.37	-0.43	0.87	-0.69	-0.41
**$^5Pr_{Cl,4}$**	0.95	0.17	0.17	0.48	-0.56	0.80	-0.76	-0.16
**$^5Pr_{OH,4}$**	0.92	0.22	0.21	0.49	-0.51	0.77	-0.52	-0.44

Cartesian coordinates of optimized QM/MM structures.

$^{1}Re_{model\ 1,\ A}$

7	3.687311659	8.571291091	6.267357517
1	4.275269340	9.400581850	6.381670593
6	2.722132378	8.169636055	7.169875782
6	3.685352094	7.742657988	5.209228904
1	4.332758024	7.843200231	4.335010985
7	2.753447781	6.799000468	5.386823800
6	2.156285639	7.049080385	6.612257748
1	1.372367939	6.398259300	6.989949575
7	6.450847982	2.323972310	4.824163220
1	7.295349290	2.628622218	4.320133225
6	6.245861798	1.330661406	5.753214607
6	5.308972465	3.040198227	4.725307825
1	5.233304749	3.919174814	4.084885839
7	4.352965604	2.548404869	5.494567420
6	4.924739294	1.476730090	6.129340121
1	4.332556344	0.819348424	6.760004634
16	3.985133636	3.900012787	11.255006148
6	2.404366663	4.511967482	10.567009482
8	2.412853005	4.524739572	9.360776323
6	1.196834019	4.906973813	11.384629348
6	-0.073275830	4.944659076	10.525088775
6	-0.497195967	3.543727006	10.009064152
6	-1.984812281	3.213119567	10.204065424
6	-2.924311764	4.045357933	9.338787431
1	1.068957146	4.206933057	12.228085812
1	1.420844586	5.886074699	11.853668117
1	-0.872516942	5.364761919	11.158799862
1	0.083934026	5.638689003	9.677542893
1	-0.225689186	3.444093900	8.941278694
1	0.099446460	2.778338660	10.536818952
1	-2.149317422	2.144415262	9.976704371
1	-3.982090782	3.817484283	9.550960509
1	-2.766829038	3.852325863	8.263280811
1	-2.769262106	5.127896964	9.505837570
6	3.307639313	4.095813058	0.488369888
6	2.622094891	4.298032381	1.837654772
8	1.599367431	3.636013148	2.066082217
8	3.050399574	5.191973095	2.698762721
1	2.695707431	4.648848104	-0.251238684
1	3.169832117	3.031161562	0.236968673
26	1.897870533	5.588062023	4.150960148
8	0.561044467	6.203806780	3.653150331
17	1.456860136	3.964489082	5.479457177
1	2.342019402	8.728490823	8.025014828
1	6.849818571	0.433311366	5.887626051
1	4.008866167	4.133392877	12.614902512
1	-2.249637104	3.336454236	11.254171943
1	4.352096510	4.359114200	0.321489736

$^{3}Re_{model\ 1,\ A}$

7	3.747330435	8.425783545	6.394348383
1	4.136698007	9.370156370	6.345616683
6	2.827719501	8.020049579	7.337743966
6	3.998236382	7.399021691	5.558493429
1	4.670508901	7.455979658	4.702248672
7	3.280843814	6.330414977	5.910930192
6	2.538191399	6.713658777	7.018355031
1	1.861911373	6.011036774	7.500548966
7	6.387190182	2.417064653	4.610574333
1	7.199473375	2.273631577	4.001798893
6	6.085790612	1.832417145	5.820179733
6	5.433266988	3.289090195	4.285167532
1	5.453030012	3.889127437	3.381810637
7	4.486710137	3.316433408	5.216392741

6	4.873652982	2.393488097	6.182449271
1	4.239405982	2.190452084	7.041428551
16	4.037086152	3.962890533	11.396444490
6	2.452685309	4.522778144	10.684293507
8	2.511571428	4.555981683	9.480117106
6	1.206023584	4.866200336	11.461747450
6	-0.020972607	4.944866402	10.542877798
6	-0.438548083	3.569588179	9.952766604
6	-1.895316946	3.169130110	10.237048565
6	-2.927284248	4.004574560	9.487020879
1	1.047697048	4.121314692	12.260423194
1	1.399102180	5.821716363	11.988816631
1	-0.844431827	5.357472999	11.150274816
1	0.188905874	5.666443196	9.731190681
1	-0.255823576	3.565938459	8.862665160
1	0.225702389	2.785468910	10.357923201
1	-2.036599107	2.105933656	9.971555190
1	-3.956604870	3.748679847	9.787804699
1	-2.872850245	3.851737280	8.395698755
1	-2.776398664	5.084065700	9.675115181
6	3.420148682	4.036043922	0.484496962
6	2.801323833	4.056460136	1.885774621
8	1.692934033	3.496667866	1.979351971
8	3.397564244	4.600865947	2.895129909
1	2.740959822	4.596757007	-0.184242240
1	3.343837963	2.983216659	0.159626402
26	2.804649051	4.484788721	4.837324816
8	1.209450166	4.984711724	4.327324554
17	1.670800856	3.461731224	6.541829315
1	2.375642996	8.659705888	8.095724154
1	6.577762620	0.892419287	6.070049483
1	4.024986689	4.143573003	12.764492395
1	-2.091088181	3.236725391	11.307175579
1	4.440336758	4.373180509	0.301132473

^{5}Re$_{model\ 1,\ A}$

7	3.780739527	8.466035598	6.466109798
1	4.147080893	9.420559604	6.421195064
6	2.867244740	8.038257658	7.404657948
6	4.057253118	7.450283292	5.628576097
1	4.712841462	7.532385878	4.761695740
7	3.368894775	6.364387473	5.982915178
6	2.609272180	6.725245732	7.085832698
1	1.939497569	6.007638969	7.557486819
7	6.352750797	2.356090172	4.547461734
1	7.180956023	2.215546960	3.966897209
6	6.053318957	1.813010103	5.776014251
6	5.433733722	3.273630964	4.232459419
1	5.475460962	3.857505921	3.320026754
7	4.505322455	3.361173196	5.176516383
6	4.869369848	2.431129337	6.147266535
1	4.237963053	2.263537452	7.017564642
16	4.082733987	3.975926698	11.358874017
6	2.469637973	4.430022099	10.632401751
8	2.506342241	4.357714102	9.427961989
6	1.244843038	4.841530166	11.411177711
6	-0.006334924	4.903383732	10.524764585
6	-0.453723273	3.515452177	9.991970997
6	-1.939699899	3.190775867	10.213745345
6	-2.893743867	4.027999121	9.368817335
1	1.089585851	4.139652901	12.249542330
1	1.471831565	5.814698108	11.890718853
1	-0.807391267	5.338839869	11.146559939
1	0.179916566	5.599385802	9.684964821
1	-0.209241415	3.435608559	8.916840808
1	0.149151873	2.734877904	10.489361320
1	-2.110845993	2.123710678	9.983366919
1	-3.947635496	3.797373759	9.597664926
1	-2.752275087	3.840770648	8.290003151
1	-2.737911481	5.109855947	9.541735764
6	3.384838754	3.955078247	0.505790022
6	2.806936758	4.006534231	1.921943420
8	1.703516139	3.449478104	2.103142934
8	3.445443477	4.593598306	2.875344533
1	2.689437723	4.497553678	-0.162275299
1	3.324678455	2.894000793	0.204485339
26	2.795058062	4.675854459	4.778287410
8	1.481734842	5.497429110	4.310647400
17	1.585848275	3.373720449	6.198287720
1	2.399614985	8.676859608	8.154042545
1	6.542952277	0.880456225	6.056495624
1	4.061252783	4.165134378	12.725653674
1	-2.186468782	3.310638599	11.268641247
1	4.397713605	4.308615529	0.313012322

^{5}TS$_{model\ 1,\ A}$

7	4.121160257	8.292460158	6.711824474
1	4.810453545	8.996211775	6.989838940
6	2.822265943	8.194191430	7.173615007
6	4.323225806	7.337276708	5.780995185
1	5.281926391	7.174087324	5.290391205
7	3.216456146	6.621005498	5.601057621
6	2.271410065	7.161843565	6.451962017
1	1.269628769	6.745742614	6.492035467
7	6.385320905	2.002086510	4.352275785
1	7.156141076	1.830363498	3.704305541
6	6.138013003	1.451063496	5.590200158
6	5.454639277	2.927574338	4.091764228
1	5.457363741	3.542399914	3.196802826
7	4.584727438	3.003179416	5.093359068
6	4.995029056	2.073510696	6.034350022
1	4.438245825	1.944907189	6.955876598
16	3.834224509	3.770759937	11.215597398
6	2.348059299	4.952704115	10.821372619
8	2.536531751	6.099504790	10.582874295

6	1.039639906	4.206225801	10.803875765
6	-0.077787431	4.908230821	10.035424932
6	0.199795432	5.069678370	8.523304721
6	0.224130187	3.772964447	7.719087470
6	-1.138602413	3.163011704	7.503239531
1	1.227827317	3.185951152	10.432103109
1	0.756163025	4.070054503	11.866991143
1	-1.016194793	4.353840424	10.209343199
1	-0.232848303	5.907319139	10.482661185
1	-0.602686192	5.704520695	8.109807286
1	1.153394182	5.602956071	8.398374323
1	0.708515567	3.988043714	6.621183122
1	-1.081429817	2.267192130	6.860731772
1	-1.841049687	3.888128588	7.053555754
1	-1.587352525	2.839964615	8.463745843
6	3.379482760	4.097891359	0.651237448
6	2.797697921	4.169483464	2.085163412
8	1.654151940	3.676099007	2.193512268
8	3.423268098	4.694483588	3.086368979
1	2.695255477	4.672319343	-0.000446954
1	3.253351850	3.040755435	0.356132521
26	3.061887848	4.503619944	5.175024442
8	1.311072513	4.043256163	5.303550018
17	3.450122631	4.290933436	7.554920971
1	2.377103632	8.761888993	7.990693530
1	6.633961355	0.536673579	5.915766792
1	4.025422302	4.123138575	12.536059557
1	0.939380916	3.040018000	8.092280734
1	4.399526355	4.387507422	0.398847042

^{5}Int$_{\text{model 1, A}}$

7	3.793407559	8.473316871	6.395177361
1	4.291240211	9.364678274	6.402755062
6	2.773632755	8.133827879	7.263407141
6	3.986255502	7.458426886	5.531229767
1	4.719057426	7.477112934	4.725173530
7	3.138146263	6.460918500	5.790350689
6	2.369785147	6.885219010	6.863199278
1	1.599715217	6.247720887	7.285262262
7	6.411255702	2.012992441	4.356491126
1	7.163297818	1.818564692	3.692242543
6	6.175176185	1.468186566	5.600273476
6	5.497234855	2.954651549	4.109519050
1	5.489433501	3.561247239	3.209048204
7	4.649304271	3.049573914	5.130154974
6	5.054937799	2.111671488	6.066714793
1	4.499715721	1.991008534	6.990240178
16	3.471991315	3.585626663	11.241784787
6	2.211595298	4.975682256	10.768955273
8	2.516527782	6.122428468	10.743767331
6	0.903855178	4.351744655	10.363126585
6	-0.090361635	5.327789860	9.721713857
6	-0.916850348	4.694955736	8.585443312
6	-1.680276188	3.463706442	8.946682653
6	-2.634603260	3.444292812	10.088296322
1	1.149174771	3.519846121	9.678138238
1	0.478929022	3.874330821	11.264117977
1	-0.756521490	5.721752479	10.512863590
1	0.470034145	6.187952439	9.310808417
1	-1.638955922	5.465076504	8.229670367
1	-0.247559325	4.489943262	7.729596845
1	1.234066745	3.362868066	6.085697720
1	-2.977092295	2.420132907	10.293596150
1	-3.555546820	4.027192855	9.856094996
1	-2.211449229	3.866460394	11.020616685
6	3.412216786	4.087624855	0.614960702
6	2.794098944	4.169318239	2.026320468
8	1.643331033	3.696227491	2.097361117
8	3.401595077	4.675410945	3.056369868
1	2.721194105	4.619387865	-0.064728151
1	3.326356021	3.019452569	0.343463806
26	3.065726431	4.415848726	5.125142496
8	1.379008891	3.631210340	5.157241515
17	2.967922029	3.916989540	7.588638933
1	2.353873078	8.749474675	8.058924681
1	6.662788249	0.545506759	5.914858555
1	3.823173721	4.004687921	12.508831038
1	-1.807061050	2.710854706	8.168732019
1	4.428048283	4.401685213	0.375127883

^{5}TSCl$_{\text{model 1, A}}$

7	3.789864000	8.507035000	6.403037000
1	4.241572000	9.424270000	6.384634000
6	2.812036000	8.132929000	7.306934000
6	4.011125000	7.486480000	5.549345000
1	4.720229000	7.528553000	4.720930000
7	3.219483000	6.451991000	5.848131000
6	2.462058000	6.855970000	6.938395000
1	1.739608000	6.179148000	7.385174000
7	6.394651000	2.038576000	4.394818000
1	7.142460000	1.830102000	3.728214000
6	6.158139000	1.503693000	5.644681000
6	5.486358000	2.989009000	4.147038000
1	5.478223000	3.587001000	3.238370000
7	4.641083000	3.102749000	5.170095000
6	5.043834000	2.165503000	6.112634000
1	4.487716000	2.047306000	7.040108000
16	3.802064000	3.917598000	11.200810000
6	2.299195000	5.105788000	10.903409000
8	2.474290000	6.128894000	10.321056000
6	0.989002000	4.530602000	11.375006000

6	-0.267342000	5.091530000	10.704782000
6	-0.359010000	4.883784000	9.189922000
6	-0.184339000	3.487351000	8.699379000
6	-0.832951000	3.076748000	7.436507000
1	1.044223000	3.439118000	11.262156000
1	0.946540000	4.706209000	12.469491000
1	-1.131979000	4.603049000	11.183811000
1	-0.365293000	6.166098000	10.939494000
1	-1.349887000	5.249287000	8.840411000
1	0.384724000	5.532494000	8.677469000
1	1.197106000	3.539081000	6.012877000
1	-1.891602000	2.794758000	7.652562000
1	-0.356429000	2.190086000	6.980020000
1	-0.870879000	3.898249000	6.695127000
6	3.421526000	4.095576000	0.546528000
6	2.803200000	4.176564000	1.955173000
8	1.652098000	3.695354000	2.028839000
8	3.416137000	4.687675000	2.975814000
1	2.732696000	4.623824000	-0.140814000
1	3.346746000	3.024890000	0.275504000
26	3.046185000	4.468064000	5.043537000
8	1.334646000	3.722499000	5.064210000
17	2.671439000	3.756257000	7.702967000
1	2.375133000	8.753365000	8.091752000
1	6.641562000	0.574935000	5.953673000
1	4.013126000	4.188871000	12.539517000
1	0.242674000	2.701308000	9.325127000
1	4.440913000	4.418222000	0.326267000

^{5}TSOH$_{model\ 1,\ A}$

7	3.619210000	8.603646000	6.279404000
1	3.842078000	9.597618000	6.167861000
6	2.968438000	8.087132000	7.382478000
6	3.898925000	7.597054000	5.424184000
1	4.407062000	7.735923000	4.466624000
7	3.449709000	6.438459000	5.910735000
6	2.882933000	6.734827000	7.139347000
1	2.519326000	5.927752000	7.773764000
7	6.385046000	2.031407000	4.396154000
1	7.118952000	1.809847000	3.718344000
6	6.163657000	1.507320000	5.653430000
6	5.486037000	2.994852000	4.161392000
1	5.474399000	3.590181000	3.250054000
7	4.661435000	3.125827000	5.197717000
6	5.066915000	2.189006000	6.138740000
1	4.532026000	2.102677000	7.083939000
16	3.586860000	3.665009000	11.224409000
6	2.124109000	4.832855000	10.777891000
8	2.277476000	6.004284000	10.602439000
6	0.810662000	4.083479000	10.709529000
6	-0.329908000	4.817964000	9.996879000
6	-0.196198000	5.235515000	8.513490000
6	-0.356712000	4.258259000	7.400557000
6	-0.552896000	2.827432000	7.638377000
1	1.003613000	3.079268000	10.306135000
1	0.504932000	3.932879000	11.766129000
1	-1.249626000	4.217015000	10.123541000
1	-0.516754000	5.751597000	10.562080000
1	-1.016797000	5.960674000	8.322623000
1	0.722425000	5.827043000	8.335374000
1	1.761432000	3.399576000	6.529227000
1	-0.460209000	2.223485000	6.722866000
1	-1.601125000	2.715262000	8.016396000
1	0.126792000	2.438019000	8.413237000
6	3.359086000	4.074679000	0.536641000
6	2.751551000	4.166572000	1.950254000
8	1.620528000	3.633853000	2.074115000
8	3.339317000	4.758366000	2.930534000
1	2.681361000	4.618980000	-0.150359000
1	3.272394000	3.007211000	0.258925000
26	3.120655000	4.537159000	4.998100000
8	1.409881000	3.794090000	5.691264000
17	2.869660000	3.530177000	8.302092000
1	2.487908000	8.681611000	8.161868000
1	6.649256000	0.576840000	5.953705000
1	3.812114000	4.058705000	12.529989000
1	-0.708607000	4.681608000	6.457843000
1	4.383477000	4.387414000	0.325493000

^{5}PrCl$_{model\ 1,\ A}$

7	3.588725000	8.571927000	6.306923000
1	3.901277000	9.546304000	6.250517000
6	2.806685000	8.080479000	7.329554000
6	3.797819000	7.586036000	5.400688000
1	4.379036000	7.717946000	4.484022000
7	3.186742000	6.465888000	5.780639000
6	2.561315000	6.761909000	6.983151000
1	1.971275000	6.007544000	7.505358000
7	6.356469000	2.068190000	4.413045000
1	7.106530000	1.851260000	3.752414000
6	6.103416000	1.530065000	5.654964000
6	5.444869000	3.019663000	4.155746000
1	5.450302000	3.615404000	3.244280000
7	4.584699000	3.135384000	5.160430000
6	4.973734000	2.196266000	6.101630000
1	4.408005000	2.057867000	7.022941000
16	4.384865000	4.629814000	11.447219000
6	2.657042000	5.199839000	11.019518000
8	2.583489000	6.072060000	10.195247000
6	1.489957000	4.413730000	11.563649000

6	0.103591000	4.919592000	11.156945000
6	-0.317165000	4.875667000	9.681888000
6	-0.265361000	3.508136000	9.002471000
6	-1.005956000	3.454362000	7.678882000
1	1.655037000	3.364365000	11.263434000
1	1.544801000	4.398884000	12.666233000
1	-0.620602000	4.316505000	11.728036000
1	-0.024363000	5.948717000	11.535513000
1	-1.382293000	5.190635000	9.638474000
1	0.257940000	5.596006000	9.072332000
1	1.449869000	3.299470000	6.297417000
1	-2.082627000	3.645888000	7.857515000
1	-0.913022000	2.457206000	7.213315000
1	-0.616366000	4.194509000	6.958623000
6	3.390127000	4.117546000	0.421612000
6	2.799496000	4.235891000	1.828575000
8	1.669102000	3.713228000	1.982870000
8	3.424959000	4.829987000	2.783035000
1	2.713439000	4.644447000	-0.279024000
1	3.317108000	3.043540000	0.167061000
26	2.946525000	4.519519000	4.773584000
8	1.408213000	3.574128000	5.366591000
17	1.567783000	3.145331000	8.629637000
1	2.378898000	8.699931000	8.120150000
1	6.595072000	0.601631000	5.951697000
1	4.349071000	4.488608000	12.821648000
1	-0.654931000	2.737736000	9.670666000
1	4.416783000	4.437819000	0.234079000

^{5}PrOH$_{model\ 1,\ A}$

7	3.497731978	8.730058712	6.265117235
1	3.461034711	9.742491452	6.125834639
6	3.265466680	8.122598643	7.485168636
6	3.835803076	7.792607298	5.363357720
1	4.093934473	8.001052995	4.324341036
7	3.821000591	6.584417765	5.930830474
6	3.504900864	6.787387657	7.261458033
1	3.485823426	5.948121467	7.950098179
7	6.422627991	1.899883820	4.279250589
1	7.166577288	1.677577458	3.615462279
6	6.186071886	1.400098351	5.540215652
6	5.545997130	2.880429524	4.033372407
1	5.568841019	3.479546220	3.126411165
7	4.717665960	3.036085890	5.061018829
6	5.097339825	2.096661306	6.007284823
1	4.579842285	2.030793910	6.961464588
16	3.141893764	3.391817395	11.540394010
6	1.974016240	4.753869944	10.930646964
8	2.253821012	5.915612421	10.939063957
6	0.680787362	4.161414881	10.395624492
6	-0.033294600	5.060717695	9.371784151
6	0.766175641	5.293521775	8.065735637
6	0.527955474	4.382523085	6.824813094
6	-0.201584490	3.080634555	7.130512049
1	0.888458427	3.155791213	9.998128466
1	0.039120502	4.012992855	11.287974193
1	-1.042994202	4.655663347	9.190244419
1	-0.182024046	6.037744863	9.866801051
1	0.670657856	6.344890409	7.752013164
1	1.816812268	5.175889711	8.335005289
1	2.400533732	3.638677407	6.994260813
1	-0.236800818	2.436009611	6.236741735
1	-1.240494936	3.275011228	7.447072674
1	0.311237652	2.511458544	7.920643268
6	3.375529907	4.143751664	0.564274258
6	2.839863056	4.269531413	1.998652753
8	1.696423910	3.797388454	2.189080891
8	3.513979016	4.828030130	2.945654007
1	2.688108377	4.696808333	-0.102117879
1	3.253483313	3.077855205	0.300277725
26	3.474912088	4.733664353	4.997519893
8	1.850778177	4.031944952	6.218608503
17	3.499424678	3.397451757	8.502490312
1	2.724065754	8.606700277	8.297940413
1	6.675541886	0.492451570	5.893293201
1	3.559735491	3.874256286	12.763917052
1	-0.051117691	4.932472859	6.082993137
1	4.398953573	4.431485565	0.323690537

^{5}Re$_{model\ 1,\ AB}$

6	5.884549277	10.458287450	2.838902604
1	5.922396515	9.715257874	2.020897216
6	6.651512476	9.917779934	4.056711331
1	6.291865240	8.908420216	4.319772554
1	6.499701276	10.550514054	4.952033547
1	7.739304581	9.852247692	3.885401632
6	4.392298333	10.652020548	3.212785921
1	4.073768497	9.710354475	3.693871954
1	4.288789327	11.427773614	3.998971415
6	3.397340026	10.935836907	2.082191764
1	3.616813893	11.874694181	1.541564945
1	2.376577815	11.016022490	2.495425564
1	3.397460965	10.112359461	1.346200844
6	2.205285219	8.867147354	8.467722647
1	1.514604010	8.177385898	8.968283271
1	1.608362598	9.675564490	8.015188716
7	3.598446543	8.445214257	6.337148704
1	3.886289798	9.419871917	6.210583588
6	2.839013869	8.004562556	7.406135807
6	3.835697754	7.419728667	5.499703969
1	4.317164615	7.521624138	4.524811519
7	3.280976399	6.305682142	5.978654577

6	2.645933686	6.661779065	7.160408002
1	2.083942933	5.933906787	7.742814735
6	-2.031416731	8.229945026	6.984694829
1	-2.931569997	8.320811358	6.352805816
6	-0.960080594	9.153797553	6.396192180
1	-1.295926671	10.202017190	6.305555314
1	-0.694492216	8.801981626	5.385536761
1	-0.031162306	9.157411066	6.988707500
6	-1.566255022	6.766765694	6.962556222
1	-2.324767393	6.062889043	7.344256688
1	-0.626139698	6.606965494	7.529845843
1	-1.340188252	6.466081206	5.923602606
6	2.718060947	7.487824164	-1.075721519
1	2.980233729	6.720256713	-1.822344711
1	3.209615181	7.217992631	-0.116452592
6	1.230998083	7.391797125	-0.737881988
8	0.702961619	6.255411849	-0.858926273
8	0.620441951	8.379265230	-0.228548447
6	6.863139613	6.249558879	2.866720706
1	6.483828039	7.097436497	3.452007471
1	6.020562123	5.802670650	2.320019133
6	7.499044013	5.168674379	3.753650732
8	7.864891863	4.103590376	3.177408866
8	7.584838520	5.410800032	4.977641863
6	-1.171348245	-1.589089999	8.740183095
1	-1.589120702	-2.325048614	8.027319856
1	-1.701258844	-1.746046808	9.694340508
6	-1.397935259	-0.171304423	8.209207337
1	-0.973946557	0.579162993	8.902754278
1	-0.899437887	-0.014527376	7.236235149
6	-2.897818595	0.131352763	8.064251808
8	-3.266212603	0.779683250	7.032151968
8	-3.658991458	-0.282825623	8.963905668
6	5.736139962	-2.308348431	8.970092486
1	6.565284049	-1.937762666	8.344308823
6	6.209840432	-3.611411978	9.644948294
1	5.446671439	-4.062331331	10.307775824
1	6.441013244	-4.372576786	8.878784147
1	7.122095405	-3.461792667	10.247898313
6	4.538656367	-2.574141515	8.041419651
1	4.848555079	-3.275418139	7.248480194
1	4.165022092	-1.665708462	7.535559328
1	3.686583167	-3.043476284	8.569195253
6	8.409868693	2.176440735	9.714373947
1	9.365756653	1.996937441	10.238061866
1	8.632026309	2.507274435	8.687641256
6	7.592916130	3.243226625	10.461976498
1	6.806146456	3.672114636	9.816504706
1	7.084451191	2.843824360	11.358187451
16	8.715397687	4.569366946	11.005404824
6	7.598440227	5.577369451	12.028522218
1	7.330405595	5.059784857	12.966721894
1	6.684823835	5.849219679	11.475845091
1	8.140960120	6.504541084	12.278109424
6	6.766459491	0.583104496	6.107727337
1	6.634501571	-0.010556245	5.184919708
1	6.189401945	0.014812343	6.850109952
7	6.359019150	2.553724973	4.627452272
1	7.253384021	2.656124782	4.120665518
6	6.052562493	1.869526646	5.785059853
6	5.374894070	3.405537437	4.345562319
1	5.379332740	4.035997232	3.469472923
7	4.397610828	3.336256393	5.238679316
6	4.810277196	2.366466491	6.155609353
1	4.175621539	2.072569057	6.987429707
6	-3.244436917	-2.165765203	1.265292590
1	-3.294823301	-2.010905117	0.176609425
1	-3.526695860	-1.214427655	1.753492369
6	-1.822665911	-2.551502969	1.664101732
1	-1.739529932	-2.773234930	2.739745191
1	-1.500705894	-3.465538655	1.131483677
7	-0.820561351	-1.508230604	1.360905917
1	-0.564181842	-1.399740219	0.374988260
6	0.125443086	-1.073886501	2.213653765
7	-0.053879647	-1.133004061	3.536733770
1	-0.977859653	-1.243060197	3.965919115
1	0.756733663	-1.070787835	4.167708164
7	1.292589198	-0.584402695	1.772862367
1	1.483980909	-0.454415119	0.778413949
1	1.795948905	0.024985437	2.429528756
16	4.003618422	3.767778309	11.323250259
6	2.417208822	4.317419483	10.599075681

8	2.444699770	4.215219025	9.396305399
6	1.228349678	4.818057520	11.380902628
6	-0.055861501	4.856958409	10.538979371
6	-0.561719697	3.451071213	10.126134961
6	-2.078409814	3.239209889	10.256949244
6	-2.919122351	4.060839194	9.283552031
1	1.084125621	4.187745259	12.275752718
1	1.501745209	5.817976250	11.775055958
1	-0.818752993	5.354525466	11.161919340
1	0.106570858	5.487522357	9.644867769
1	-0.240045754	3.231479938	9.091721506
1	-0.057760490	2.696876632	10.753889692
1	-2.299240862	2.168469368	10.093101691
1	-3.997714116	3.899098390	9.452764998
1	-2.712696809	3.784921556	8.236253962
1	-2.717968373	5.142057784	9.390263428
6	3.358810933	4.009497433	0.508128113
6	2.814549857	3.973485683	1.934682960
8	1.763063624	3.316237755	2.116855530
8	3.395579904	4.595890908	2.900259732
1	2.658419327	4.620429566	-0.093646572
1	3.258809493	2.974577417	0.138545017
26	2.714031029	4.616018181	4.820367236
8	1.400702560	5.432802397	4.336422809
17	1.439797635	3.296881219	6.241275328
8	2.168378228	1.218265650	3.838514469
1	2.099200389	1.988132297	3.228230451
1	1.796977578	1.523720856	4.688973245
8	-1.882609204	2.825713941	6.111361917
1	-2.347841458	1.993536911	6.454095654
1	-1.093704575	2.980909208	6.659881896
8	-0.229160620	5.380944820	1.542981119
1	0.294876883	4.638772744	1.906910463
1	0.119628936	5.581624375	0.633744404
8	-1.048544658	2.905333278	3.568321027
1	-1.272491671	2.868700855	4.540107106
1	-0.082881725	2.844894505	3.496532767
8	1.197624890	7.802198535	2.449118358
1	0.743082409	6.934015270	2.524617224
1	0.945378902	8.127634913	1.553518789
8	3.905123639	7.393609600	2.266284258
1	3.905902842	6.423479824	2.376415436
1	2.947575682	7.637428165	2.374492994
1	6.384374858	11.320945623	2.398377250
1	2.842878482	9.341378759	9.213810259
1	-2.293051144	8.568333112	7.987245702
1	3.097668056	8.458518725	-1.394653837
1	7.521290132	6.598622581	2.071070194
1	-0.130191775	-1.894368708	8.844420499
1	5.505857343	-1.566636400	9.734880364
1	7.853998214	1.238977404	9.698951938
1	7.806481696	0.409907899	6.384176385
1	-4.015981934	-2.899911259	1.497273577
1	4.040030558	4.056499252	12.672198739
1	-2.386260340	3.450966547	11.280889917
1	4.377158440	4.347105040	0.315626738

$^{5}TS_{model\ 1,\ AB}$

6	5.875553370	10.454629777	2.822720581
1	5.914617208	9.719457689	1.997576999
6	6.634608348	9.897063401	4.036560854
1	6.262997012	8.888710221	4.285013483
1	6.483118063	10.523798233	4.936586117
1	7.722216892	9.826822874	3.866704170
6	4.383275861	10.643877838	3.196071598
1	4.067438378	9.692370968	3.660258589
1	4.280214491	11.407423484	3.994442568
6	3.387996069	10.944681512	2.070519437
1	3.607477492	11.891134850	1.543510979
1	2.367558570	11.018577120	2.485903792
1	3.385372535	10.132608312	1.321930304
6	2.133685171	8.880559764	8.445405496
1	1.421304499	8.213255633	8.946430616
1	1.561191357	9.704066061	7.990063370
7	3.368471921	8.420655405	6.225502412
1	3.578939402	9.400359855	6.017469156
6	2.756727844	7.998380422	7.394029782
6	3.626153549	7.355260119	5.442717358
1	4.029801784	7.422975069	4.427217380
7	3.225017578	6.239669402	6.053609814
6	2.679725574	6.630791218	7.264599351
1	2.262634284	5.899449056	7.949702476
6	-2.298403470	8.801076992	7.181010693
1	-3.262047772	9.011594518	6.680913305
6	-1.345590432	9.970364208	6.889140129
1	-1.673039588	10.918827605	7.350518368
1	-1.258715943	10.126813488	5.800439919
1	-0.325746560	9.771428102	7.259010726
6	-1.728632659	7.495915284	6.620603329
1	-2.408391226	6.635317409	6.737577732
1	-0.751665664	7.236110665	7.068953087
1	-1.533641712	7.617060747	5.540906689
6	2.714661920	7.511040149	-1.072377732
1	2.980330680	6.733903353	-1.807510248
1	3.185074062	7.243523269	-0.101946098
6	1.216700217	7.454684878	-0.781397557
8	0.641724682	6.356829129	-0.982191895
8	0.637358833	8.447554966	-0.238592983
6	6.872604779	6.196617992	2.858985055
1	6.523113358	7.019954787	3.496779787
1	6.006703040	5.791727449	2.319505175
6	7.527399018	5.075995477	3.677613549
8	7.807414567	4.000862848	3.070374380
8	7.733792612	5.300016892	4.889999204
6	-1.190990029	-1.591711940	8.775257286
1	-1.605709663	-2.332391086	8.066814153
1	-1.701014724	-1.748182070	9.740484362
6	-1.428089890	-0.178666623	8.242317714
1	-1.022783621	0.567853438	8.950065550
1	-0.912910591	-0.013733335	7.279820394
6	-2.921914507	0.128677280	8.072756750
8	-3.278796274	0.782089064	7.042166177
8	-3.691175336	-0.287173899	8.966553327
6	5.803106332	-2.370168959	9.022423295
1	6.593770798	-1.981848052	8.358097531
6	6.370465762	-3.601356838	9.752860373
1	5.664841715	-4.037355932	10.485129691
1	6.601234436	-4.399946786	9.025587272
1	7.304049250	-3.365603917	10.290020271
6	4.608811782	-2.765294901	8.142448870
1	4.959703405	-3.447030987	7.350352381
1	4.124849098	-1.908087793	7.642441569
1	3.832003594	-3.316923780	8.705817769
6	8.401027157	2.180308349	9.591619912
1	9.371109170	2.013954866	10.094732668
1	8.596396836	2.463425982	8.545549766
6	7.636511075	3.297852804	10.312444025
1	6.755973081	3.631730940	9.738475552
1	7.279847292	2.987664251	11.308735568
16	8.765421156	4.710823068	10.580513747
6	7.920329064	5.651903675	11.892330949
1	7.792845094	5.038158674	12.802370590
1	6.948929051	6.050911756	11.564367655
1	8.572342719	6.507911911	12.134707174
6	6.873989943	0.436608040	6.044961841
1	6.772100255	-0.185605946	5.137541728
1	6.297740441	-0.136718567	6.784974720
7	6.379105533	2.439415062	4.584546385

1	7.257566554	2.624045047	4.060717711
6	6.126333304	1.697111274	5.723933880
6	5.350552171	3.262715911	4.365349617
1	5.300564479	3.950790366	3.532100421
7	4.407143052	3.104722063	5.284861991
6	4.882042511	2.126959286	6.147143528
1	4.287146745	1.788461899	6.986555675
6	-3.323138375	-2.184038667	1.254980714
1	-3.313028523	-2.077438969	0.159126673
1	-3.621427742	-1.212223711	1.689815296
6	-1.934196710	-2.564698353	1.745137300
1	-1.882741732	-2.619211129	2.843878892
1	-1.629373173	-3.557049954	1.363207452
7	-0.898736616	-1.617037522	1.307300045
1	-0.663638733	-1.636395784	0.311202557
6	0.096819756	-1.150056745	2.079564140
7	-0.085442059	-0.920712744	3.381214181
1	-1.018064498	-0.915253582	3.807799789
1	0.714680154	-0.827255247	4.020100325
7	1.304789379	-0.921754032	1.547320182
1	1.401185085	-0.991322489	0.531205601
1	1.934315621	-0.278323282	2.070851914
16	4.599607130	4.530141900	11.680658192
6	3.003343423	4.661652728	10.850446130
8	3.150596439	4.764196060	9.654300454
6	1.645724253	4.683451476	11.517494458
6	0.539187838	4.628056995	10.465089304
6	0.477098090	3.278710637	9.715550313
6	0.022165584	3.356319360	8.263589904
6	-1.135811040	4.291771484	7.984278150
1	1.563082044	3.850704771	12.236505155
1	1.589059143	5.603720298	12.129888962
1	-0.421337382	4.832601983	10.969072038
1	0.711781935	5.450980625	9.748863032
1	1.447230888	2.755285672	9.756599206
1	-0.227760481	2.610727677	10.251545217
1	0.984477713	3.643005749	7.562213060
1	-1.427822656	4.275969579	6.927563609
1	-0.912983221	5.332432888	8.262835948
1	-2.019533923	3.964024475	8.571339257

6	3.284481564	4.082029949	0.607201026
6	2.799959074	3.866700804	2.060905869
8	1.913122888	2.975207709	2.197493867
8	3.240434416	4.547262086	3.042685858
1	2.635983592	4.850622088	0.146277138
1	3.055893497	3.143081619	0.081371273
26	2.686106780	4.334065875	5.272158355
8	2.129669441	3.488763546	6.727844046
17	0.574684534	5.080088890	4.672656509
8	2.786448259	0.667120909	3.253164214
1	3.697357766	0.604852628	2.859769021
1	2.455344525	1.573361153	3.026529510
8	-1.916606398	2.484568275	5.437330302
1	-2.358630678	1.835130702	6.058769382
1	-0.981638450	2.521158411	5.695156459
8	-0.300973085	5.744956044	1.506124664
1	-0.460279927	4.801496902	1.736493079
1	-0.062688798	5.802832488	0.546144783
8	-0.844734030	3.214261796	2.649715297
1	-0.893159162	3.646405120	3.527676658
1	0.118404844	3.020488153	2.551704982
8	1.241064643	7.833026655	2.418934225
1	0.771368605	6.961571836	2.418843503
1	1.036832875	8.191182739	1.524724329
8	3.891200182	7.264165410	2.282511801
1	3.824159727	6.302279501	2.463143846
1	2.944942226	7.566861771	2.351364387
1	6.379315437	11.319576145	2.391259245
1	2.791226666	9.330175992	9.189416077
1	-2.504048285	8.759964810	8.250630866
1	3.103623719	8.476516509	-1.395845554
1	7.522358239	6.569316364	2.067162040
1	-0.144315224	-1.884716658	8.857033830
1	5.530340744	-1.603916625	9.748042765
1	7.837695332	1.247648349	9.621113876
1	7.909428542	0.297574804	6.355773648
1	-4.094571000	-2.919529526	1.483042598
1	4.382999419	4.406900207	13.037949692
1	-0.176140616	2.357030213	7.876080972
1	4.321794964	4.341153437	0.395297162

^{5}**Int**$_{\mathbf{model\ 1,\ AB}}$

6	5.886402000	10.473958000	2.830355000
1	5.921452000	9.736530000	2.004946000
6	6.651406000	9.916637000	4.044112000
1	6.286763000	8.903565000	4.291137000
1	6.498392000	10.541657000	4.947243000
1	7.740969000	9.851706000	3.873189000
6	4.392900000	10.667939000	3.207720000
1	4.074279000	9.718728000	3.677520000
1	4.292102000	11.436595000	4.004026000
6	3.394422000	10.965894000	2.080901000
1	3.613459000	11.912135000	1.549829000
1	2.372746000	11.040406000	2.497784000
1	3.391718000	10.150272000	1.333786000
6	2.204127000	8.875970000	8.499576000
1	1.514106000	8.184019000	9.003664000
1	1.601147000	9.684707000	8.052032000
7	3.480480000	8.483689000	6.282939000
1	3.694583000	9.471301000	6.111644000
6	2.841223000	8.022607000	7.425433000
6	3.742419000	7.447254000	5.461081000
1	4.162630000	7.547055000	4.453648000
7	3.318624000	6.308904000	6.017096000
6	2.749200000	6.659526000	7.235551000
1	2.298779000	5.910828000	7.883502000
6	-2.124158000	8.235808000	7.189034000
1	-3.032269000	8.268385000	6.558459000
6	-1.146521000	9.281117000	6.630874000
1	-1.572789000	10.301879000	6.617589000
1	-0.876332000	9.018692000	5.592074000
1	-0.204905000	9.332462000	7.204111000
6	-1.531748000	6.818264000	7.131756000
1	-2.226008000	6.037720000	7.497120000
1	-0.582563000	6.729573000	7.700079000
1	-1.272561000	6.557518000	6.091263000
6	2.717201000	7.510835000	-1.071391000
1	2.982036000	6.735023000	-1.810966000
1	3.198510000	7.243202000	-0.104473000
6	1.219252000	7.437349000	-0.759687000
8	0.664395000	6.320805000	-0.919820000
8	0.627427000	8.437121000	-0.239232000
6	6.885944000	6.205676000	2.866716000
1	6.548629000	7.024219000	3.520174000
1	6.010042000	5.814134000	2.329194000
6	7.535345000	5.061902000	3.665181000
8	7.842588000	4.013901000	3.024759000
8	7.701635000	5.248325000	4.893243000
6	-1.172779000	-1.504146000	8.743437000
1	-1.603442000	-2.241429000	8.038100000
1	-1.688226000	-1.645635000	9.710311000
6	-1.388528000	-0.086478000	8.197674000
1	-0.970648000	0.673371000	8.888565000
1	-0.874211000	0.059028000	7.229130000
6	-2.884158000	0.229556000	8.023019000
8	-3.227516000	0.897909000	6.991912000
8	-3.668533000	-0.189855000	8.906858000
6	5.856167000	-2.368632000	9.040721000
1	6.660343000	-1.983031000	8.387785000
6	6.412260000	-3.596782000	9.789311000
1	5.693995000	-4.026969000	10.515062000
1	6.653898000	-4.401752000	9.070024000
1	7.339035000	-3.357291000	10.339244000
6	4.676151000	-2.774205000	8.142820000
1	5.043524000	-3.461897000	7.361255000
1	4.193100000	-1.923919000	7.626810000
1	3.891532000	-3.325644000	8.698793000
6	8.436002000	2.207750000	9.666791000
1	9.393865000	2.051262000	10.198511000
1	8.657218000	2.499032000	8.625929000
6	7.623602000	3.306887000	10.370878000
1	6.766668000	3.639834000	9.759113000
1	7.220724000	2.972625000	11.344242000
16	8.697890000	4.746597000	10.727095000
6	7.707505000	5.640945000	11.972300000
1	7.608215000	5.046529000	12.899943000
1	6.710124000	5.911738000	11.586753000
1	8.252653000	6.573035000	12.205299000
6	6.959070000	0.416726000	6.096665000
1	6.852007000	-0.249924000	5.220477000
1	6.386603000	-0.119117000	6.867796000

7	6.481153000	2.309532000	4.507665000
1	7.360965000	2.421695000	3.979994000
6	6.227881000	1.670633000	5.709523000
6	5.474825000	3.153204000	4.242134000
1	5.434575000	3.768581000	3.351620000
7	4.553694000	3.111783000	5.190997000
6	5.008329000	2.186023000	6.124191000
1	4.429154000	1.976931000	7.020731000
6	-3.355843000	-2.193355000	1.251717000
1	-3.337577000	-2.093534000	0.153370000
1	-3.661217000	-1.218875000	1.679971000
6	-1.968406000	-2.567182000	1.755744000
1	-1.919967000	-2.593764000	2.857368000
1	-1.661545000	-3.570553000	1.400206000
7	-0.936830000	-1.627175000	1.292605000
1	-0.699901000	-1.681300000	0.297288000
6	0.072026000	-1.153755000	2.049769000
7	-0.098260000	-0.891830000	3.348259000
1	-1.022361000	-0.912063000	3.794866000
1	0.716048000	-0.780337000	3.969477000
7	1.276131000	-0.945155000	1.498934000
1	1.348722000	-1.017063000	0.478345000
1	1.900256000	-0.279315000	2.007767000
16	4.290702000	4.134182000	11.464746000
6	2.681624000	4.548077000	10.718246000
8	2.777927000	4.573432000	9.512131000
6	1.409033000	4.833733000	11.481491000
6	0.182713000	4.962815000	10.567419000
6	-0.253521000	3.628154000	9.910527000
6	-1.557228000	3.719577000	9.175794000
6	-2.859304000	3.673870000	9.901647000
1	1.251371000	4.043771000	12.238861000
1	1.581317000	5.760842000	12.064873000
1	-0.647117000	5.344937000	11.189041000
1	0.384856000	5.710222000	9.774520000
1	0.551248000	3.273903000	9.239796000
1	-0.339281000	2.875943000	10.725075000
1	1.411116000	3.765497000	6.921063000
1	-3.673420000	4.165388000	9.335445000
1	-2.794205000	4.128739000	10.910249000

1	-3.192807000	2.621322000	10.049173000
6	3.305560000	4.074016000	0.602430000
6	2.812058000	3.902211000	2.056240000
8	1.872016000	3.086830000	2.223806000
8	3.323278000	4.568510000	3.033463000
1	2.640016000	4.810463000	0.109038000
1	3.097920000	3.110144000	0.108902000
26	2.843914000	4.429091000	5.108430000
8	2.244071000	3.403478000	6.570600000
17	0.674992000	5.146196000	4.709714000
8	2.616967000	0.662393000	3.227600000
1	3.573883000	0.596913000	2.968047000
1	2.335527000	1.585616000	3.016511000
8	-1.769300000	2.748667000	5.640139000
1	-2.260299000	2.000666000	6.107790000
1	-0.897991000	2.805025000	6.069527000
8	-0.287728000	5.750075000	1.613952000
1	-0.553969000	4.854382000	1.916790000
1	-0.038437000	5.715529000	0.656628000
8	-1.017107000	3.174812000	2.888727000
1	-1.237185000	3.294242000	3.845708000
1	-0.038754000	3.085511000	2.856980000
8	1.247799000	7.870691000	2.431910000
1	0.770604000	7.000675000	2.443318000
1	1.039753000	8.219920000	1.532766000
8	3.911774000	7.327506000	2.290150000
1	3.869025000	6.361153000	2.451353000
1	2.958431000	7.612454000	2.364742000
1	6.390674000	11.339212000	2.395833000
1	2.843719000	9.348956000	9.247220000
1	-2.412456000	8.502281000	8.207681000
1	3.103564000	8.479028000	-1.395569000
1	7.534359000	6.580597000	2.072514000
1	-0.131466000	-1.820696000	8.828456000
1	5.569898000	-1.597137000	9.758084000
1	7.882485000	1.266879000	9.678892000
1	7.997667000	0.287651000	6.407181000
1	-4.125948000	-2.932461000	1.480684000
1	4.212955000	4.233399000	12.841113000
1	-1.578367000	3.624077000	8.088505000

1	4.340144000	4.348845000	0.388261000

^{5}Re$_{\text{model 1, A,BS2}}$

7	3.734987386	8.564342496	6.301000564
1	4.377687712	9.335373539	6.454058088
6	2.718894608	8.208126048	7.160922860
6	3.712251820	7.765716950	5.227274636
1	4.378812082	7.844692378	4.381794445
7	2.719618882	6.882795733	5.353388227
6	2.103282594	7.149454599	6.564120346
1	1.267777643	6.562130126	6.893985551
7	6.441848990	2.349443404	4.845857625
1	7.289186910	2.644490090	4.356143510
6	6.218616620	1.408683177	5.820766898
6	5.311804722	3.062291017	4.693731850
1	5.253304506	3.902485648	4.022344174
7	4.342338021	2.612844259	5.468030541
6	4.901426453	1.571353285	6.163273755
1	4.301021470	0.957679278	6.806553012
16	3.983279648	3.905945531	11.262648029
6	2.395566499	4.481857556	10.581539885
8	2.404149246	4.469409799	9.376942529
6	1.195327203	4.889774240	11.391116194
6	-0.077606569	4.924449257	10.541415406
6	-0.522864567	3.519865404	10.070602990
6	-2.016834209	3.228755617	10.238462193
6	-2.913941367	4.047048581	9.323871258
1	1.070804716	4.203425242	12.230951672
1	1.429841033	5.860092139	11.844999945
1	-0.858356031	5.365014569	11.163808826
1	0.080741452	5.582005732	9.680874116
1	-0.231915824	3.375158858	9.025939201
1	0.037115497	2.773877918	10.638407442
1	-2.193884031	2.168417371	10.041835480
1	-3.967755392	3.831539855	9.497221046
1	-2.715803704	3.832198135	8.272508112
1	-2.761339518	5.119231475	9.473588022
6	3.269350740	4.153885684	0.467895370
6	2.582448138	4.351115685	1.810257424
8	1.564749635	3.678470361	2.036971917
8	2.997446584	5.246384783	2.672251432
1	2.684129366	4.735060820	-0.252554705
1	3.106087034	3.110273323	0.200162729
26	1.850082727	5.693628280	4.113955852
8	0.505336216	6.305934496	3.619541442
17	1.458712621	4.066862088	5.423564962
1	2.340110141	8.759675588	8.021377825
1	6.798087997	0.502199015	5.995600277
1	4.011790723	4.135011318	12.623186014
1	-2.305526715	3.392030251	11.276761456
1	4.322004968	4.390604612	0.313135705

^{5}TS$_{\text{model 1, A,BS2}}$

7	4.117149622	8.284317782	6.712400854
1	4.805150770	8.990319005	6.989395600
6	2.823152019	8.187319481	7.174402274
6	4.320141978	7.328311948	5.783405878
1	5.279139976	7.166309963	5.293403049
7	3.215142114	6.611310420	5.604416690
6	2.272157633	7.154317923	6.454402222
1	1.272159051	6.739317646	6.495400320
7	6.379139438	2.007341669	4.354405512
1	7.149137929	1.836343232	3.705404113
6	6.134142997	1.456340701	5.590402224
6	5.448138160	2.932338828	4.098412080
1	5.449136119	3.546338745	3.204413294
7	4.581142193	3.006337953	5.100413625
6	4.992145055	2.081343779	6.032397654
1	4.435144847	1.952344902	6.953396413
16	3.833148797	3.772332753	11.211391391
6	2.348151397	4.953330957	10.815390074
8	2.537150179	6.097324866	10.576388514
6	1.042154678	4.206331197	10.799393073
6	-0.074843565	4.907329891	10.031393834
6	0.204158308	5.068327942	8.520395029
6	0.224150079	3.773331347	7.719402759
6	-1.133840580	3.165334327	7.502400536
1	1.230154882	3.187331812	10.428395370
1	0.758154975	4.071332583	11.862392831
1	-1.011843136	4.354330612	10.205394701

1	-0.229843762	5.906329906	10.478392423
1	-0.597841045	5.703327321	8.106394701
1	1.157157251	5.601327311	8.395395949
1	0.709150251	3.988331422	6.621402365
1	-1.077844218	2.269334251	6.861401795
1	-1.834838866	3.890335114	7.054400829
1	-1.581841245	2.843336400	8.463400343
6	3.380152413	4.099331970	0.660418334
6	2.802159774	4.169331166	2.096423526
8	1.660163118	3.678334204	2.209431328
8	3.425155821	4.691325591	3.099426703
1	2.695150154	4.673333019	0.011421989
1	3.252149850	3.042332960	0.365418047
26	3.062151092	4.504331608	5.175401846
8	1.311150466	4.043331444	5.304401892
17	3.449148680	4.292332523	7.553397083
1	2.378568934	8.757501865	7.990064735
1	6.630420212	0.542032779	5.915697894
1	4.024954600	4.124220240	12.531896351
1	0.941947632	3.041829389	8.090534645
1	4.399425584	4.388441959	0.404361836

^{5}Re$_{\text{model 1, AB,BS2}}$

6	5.883048234	10.453271045	2.838193618
1	5.921636464	9.716514119	2.023007231
6	6.650624903	9.914503009	4.058576866
1	6.292488595	8.910092866	4.319025308
1	6.499838296	10.548691520	4.949390058
1	7.735687803	9.851458588	3.885218447
6	4.390282586	10.647843011	3.215091377
1	4.074546599	9.711925841	3.692723290
1	4.289075828	11.425396264	3.996932310
6	3.395322586	10.932823835	2.081535838
1	3.616412195	11.872079114	1.543101242
1	2.379452787	11.014990298	2.493682046
1	3.396575461	10.113479439	1.347902662
6	2.202520193	8.862542576	8.464166941
1	1.516385295	8.177956007	8.965411553
1	1.609852883	9.672759363	8.015720111
7	3.596404469	8.439785692	6.335321074
1	3.884596240	9.414905556	6.210759772
6	2.839158022	7.997699350	7.400458349
6	3.831805269	7.415137684	5.501070300
1	4.315003736	7.520864398	4.528867678
7	3.281065645	6.307858654	5.977035247
6	2.646256865	6.662903556	7.157695587
1	2.086058103	5.936441462	7.739343433
6	-2.029676717	8.228037384	6.981780020
1	-2.929214131	8.320066245	6.353797981
6	-0.956949738	9.151975215	6.394467178
1	-1.295012867	10.198760729	6.305832836
1	-0.694386513	8.802346901	5.388541303
1	-0.033386205	9.156437237	6.986930343
6	-1.563492405	6.763397025	6.961450983
1	-2.322297395	6.064377523	7.342395302
1	-0.628343086	6.606876259	7.527731283
1	-1.340120084	6.466106078	5.927145278
6	2.716508611	7.483932568	-1.072654958
1	2.979065978	6.721913339	-1.819635387
1	3.208023648	7.217685819	-0.118559310
6	1.230230829	7.388379217	-0.735055520
8	0.707149633	6.260757280	-0.852737164
8	0.623938845	8.370910537	-0.227924161
6	6.857070881	6.249181406	2.868462466
1	6.483886862	7.093596072	3.450235764
1	6.023417490	5.804550187	2.321914826
6	7.497702416	5.164335353	3.757850611
8	7.857639705	4.110624877	3.183487091
8	7.578755971	5.407442228	4.969351280
6	-1.172299563	-1.588304896	8.738367419
1	-1.587906648	-2.322995644	8.028267856
1	-1.699987903	-1.745371705	9.690980259
6	-1.397120607	-0.166849221	8.207734922
1	-0.975429618	0.576815791	8.899900579
1	-0.900479265	-0.015143111	7.238391366
6	-2.900141075	0.131604641	8.063481522
8	-3.260369078	0.779657071	7.035543545
8	-3.651639461	-0.278787561	8.957140486
6	5.736241151	-2.306950450	8.967538988

1	6.562857692	-1.939050562	8.344737912
6	6.209449995	-3.610416220	9.644369358
1	5.448275836	-4.060913654	10.306083378
1	6.440179234	-4.371171969	8.880078748
1	7.119693819	-3.462165890	10.246284301
6	4.537374991	-2.572509808	8.038812194
1	4.847857063	-3.273149963	7.249613591
1	4.165808825	-1.668426205	7.536932617
1	3.688640624	-3.041616263	8.567479263
6	8.409367568	2.178294216	9.712100570
1	9.363594519	1.997946243	10.236305775
1	8.630893536	2.506165438	8.690384669
6	7.590410873	3.245271271	10.460938125
1	6.807524862	3.670147684	9.818469954
1	7.084635059	2.844577358	11.354982579
16	8.705900538	4.566820979	11.000982002
6	7.596306987	5.575873568	12.026150165
1	7.330146304	5.060931621	12.964012570
1	6.687054904	5.847933704	11.477042016
1	8.139362560	6.502094004	12.276566772
6	6.760365702	0.582883617	6.106955410
1	6.632960956	-0.009375412	5.187009968
1	6.190201733	0.016970635	6.847090969
7	6.354129113	2.557177691	4.628418756
1	7.248831224	2.655966230	4.123107110
6	6.043576650	1.875444467	5.782995660
6	5.371058228	3.405502051	4.349084190
1	5.378223325	4.033456949	3.472545014
7	4.401208122	3.336845674	5.235934839
6	4.813233196	2.366493606	6.152655094
1	4.177736078	2.074370662	6.982929012
6	-3.242030007	-2.162451787	1.264963362
1	-3.294595781	-2.011197006	0.179290321
1	-3.525906513	-1.215993516	1.751617843
6	-1.821053217	-2.551053290	1.665535987
1	-1.739918718	-2.772126235	2.737236124
1	-1.501627234	-3.463820485	1.132122394
7	-0.816892544	-1.505813529	1.363236869
1	-0.564426370	-1.400164270	0.378616468
6	0.125240824	-1.071720700	2.215012724
7	-0.053404319	-1.130340723	3.536366198

1	-0.974316573	-1.242374653	3.963643378
1	0.753504687	-1.070933486	4.164670655
7	1.291227424	-0.580961926	1.775492056
1	1.482693601	-0.454456416	0.781919585
1	1.793556071	0.022390468	2.427255109
16	3.997157628	3.770434091	11.313360783
6	2.419147425	4.315118038	10.589416307
8	2.444674513	4.215704450	9.401718851
6	1.229529860	4.817404446	11.379299263
6	-0.055770588	4.857605799	10.535752120
6	-0.559290858	3.449203387	10.122512425
6	-2.076423775	3.236982339	10.253449096
6	-2.917745844	4.061047875	9.280823313
1	1.084657322	4.189472261	12.272508433
1	1.501322195	5.814708998	11.772788306
1	-0.816821735	5.353332441	11.159374776
1	0.107046594	5.485573804	9.646443082
1	-0.240733362	3.231504539	9.094487187
1	-0.059166324	2.699137801	10.751359897
1	-2.298026209	2.170269079	10.092316222
1	-3.994890892	3.899191721	9.451883150
1	-2.713473795	3.785676131	8.238253696
1	-2.718005764	5.138920197	9.389026995
6	3.354617393	4.007768695	0.510349191
6	2.813177869	3.974101153	1.941479978
8	1.769353869	3.321804234	2.121593429
8	3.391081542	4.593200931	2.900534217
1	2.659073501	4.618095926	-0.091524433
1	3.258297688	2.977795122	0.140777082
26	2.706597433	4.615021327	4.820316541
8	1.408913036	5.427260054	4.338588266
17	1.447258839	3.305365867	6.232078043
8	2.165417545	1.227028444	3.841576664
1	2.099117493	1.984707202	3.232089222
1	1.798615863	1.523605246	4.683346625
8	-1.876766029	2.824384917	6.114442162
1	-2.345344911	1.996884560	6.452811087
1	-1.098239360	2.979653714	6.657036865
8	-0.221311239	5.377047197	1.544384544
1	0.292548641	4.642918308	1.904551386
1	0.119225523	5.580634321	0.638067882

8	-1.040853664	2.905347327	3.572779462
1	-1.269603754	2.869186201	4.535611471
1	-0.088099968	2.845322503	3.498214709
8	1.196928615	7.795245310	2.447379556
1	0.745376869	6.938297467	2.524183098
1	0.945738319	8.125266543	1.557735591
8	3.898833643	7.386998112	2.269500968
1	3.904803826	6.427943423	2.375584681
1	2.952742680	7.634285768	2.373662662
1	6.382396703	11.316891485	2.399014665
1	2.839681791	9.338075862	9.209794539
1	-2.292272650	8.566648137	7.984004274
1	3.096124737	8.454321069	-1.392506589
1	7.517133450	6.597312619	2.073988189
1	-0.131221405	-1.893446654	8.843781369
1	5.505745342	-1.566353237	9.733342337
1	7.853916689	1.240557797	9.698242047
1	7.800774826	0.410841521	6.382668484
1	-4.013325750	-2.896898563	1.496821368
1	4.037584066	4.055901748	12.662887199
1	-2.384664024	3.450171051	11.276975280
1	4.372676049	4.345832792	0.317122479

^{5}TSe$_{\text{model 1, AB,BS2}}$

6	5.874232099	10.450423733	2.825061010
1	5.913232113	9.715423469	2.000060714
6	6.632232369	9.892423533	4.038061442
1	6.261232238	8.885423173	4.285061532
1	6.479232314	10.518423756	4.937061763
1	7.718232759	9.823423508	3.868061383
6	4.381231565	10.637423797	3.198061140
1	4.066231452	9.686423461	3.661061308
1	4.278231526	11.399424070	3.995061425
6	3.387231212	10.938423906	2.073060742
1	3.606231290	11.884424242	1.547060551
1	2.368230848	11.012423931	2.487060891
1	3.384231211	10.128423616	1.325060472
6	2.137230765	8.872423167	8.437063011
1	1.425230508	8.206422933	8.938063191
1	1.565230561	9.695423464	7.982062850
7	3.361231199	8.404423004	6.227062225
1	3.572231276	9.382423350	6.021062148
6	2.758230988	7.987422855	7.385062636
6	3.619231291	7.342422623	5.442061946
1	4.023231435	7.414422649	4.428061582
7	3.220231148	6.228422227	6.048062158
6	2.679230958	6.622422365	7.258062594
1	2.262230808	5.893422103	7.943062836
6	-2.292770818	8.797423140	7.179062564
1	-3.254771161	9.008423217	6.678062384
6	-1.340770480	9.965423561	6.887062462
1	-1.667770597	10.913423896	7.348062626
1	-1.252770446	10.121423617	5.800062071
1	-0.321770116	9.767423490	7.258062594
6	-1.722770613	7.492422677	6.620062367
1	-2.401770859	6.633422367	6.737062408
1	-0.747770269	7.233422582	7.069062526
1	-1.527770543	7.613422717	5.542061978
6	2.718230972	7.508422679	-1.061940378
1	2.982231066	6.732422402	-1.796940641
1	3.191231140	7.243422588	-0.093940032
6	1.223230439	7.448422663	-0.763940274
8	0.650230233	6.352422268	-0.958940344
8	0.643230229	8.437423016	-0.220940079
6	6.863232452	6.197422214	2.861061023
1	6.514232325	7.020422506	3.497061251
1	6.000232144	5.795422070	2.319060830
6	7.506232679	5.074421815	3.685061317
8	7.783232780	4.000421429	3.081061098
8	7.706232754	5.298421893	4.894061745
6	-1.187770425	-1.585580565	8.771063133
1	-1.602770570	-2.324580832	8.063062879
1	-1.699770607	-1.740580624	9.734063479
6	-1.420770507	-0.172580063	8.238062944
1	-1.015770361	0.572420206	8.946063198
1	-0.903770325	-0.008580001	7.279062601
6	-2.910771039	0.139420049	8.066062880
8	-3.258771164	0.792420282	7.036062514
8	-3.678771315	-0.275580096	8.957063200

6	5.802232073	-2.365580845	9.020063222
1	6.592232353	-1.977580709	8.357062984
6	6.369232276	-3.595581286	9.750063481
1	5.665232026	-4.031581439	10.482063743
1	6.599232357	-4.394581569	9.024063226
1	7.302232606	-3.361581202	10.287063674
6	4.608231644	-2.759580988	8.141062907
1	4.959231770	-3.440581227	7.349062623
1	4.124231474	-1.903580679	7.642062731
1	3.832231368	-3.311581180	8.704063107
6	8.395233000	2.183420779	9.588063423
1	9.365233344	2.020420724	10.089063602
1	8.588233066	2.465420880	8.543063051
6	7.628232726	3.298421177	10.307063679
1	6.747232412	3.629421298	9.736063478
1	7.274232599	2.987421067	11.304064038
16	8.752233128	4.711421684	10.572063776
6	7.912232827	5.651422018	11.885064245
1	7.789232782	5.039421798	12.795064568
1	6.942232482	6.050422161	11.561064129
1	8.565233059	6.507422322	12.125064331
6	6.866232453	0.445420159	6.044062160
1	6.765232418	-0.176580061	5.137061833
1	6.291232248	-0.128580044	6.782062425
7	6.370232273	2.448420876	4.587061639
1	7.248232587	2.632420938	4.064061449
6	6.117232186	1.706420608	5.723062045
6	5.343231908	3.269421169	4.370061559
1	5.292231890	3.957421412	3.539061266
7	4.402231572	3.109421109	5.289061891
6	4.876231742	2.138420763	6.144062192
1	4.281231527	1.799420641	6.982062495
6	-3.318771186	-2.179580778	1.258060451
1	-3.307771184	-2.072580741	0.163060058
1	-3.617771291	-1.208580431	1.693060602
6	-1.929770687	-2.558580916	1.749060626
1	-1.879770671	-2.612580935	2.847061014
1	-1.623770578	-3.549581268	1.367060487
7	-0.894770321	-1.609580573	1.313060467
1	-0.659770235	-1.628580582	0.318060112
6	0.098230035	-1.141580405	2.085060746
7	-0.082770032	-0.912580327	3.383061210
1	-1.014770364	-0.907580327	3.809061358
1	0.715230255	-0.818580291	4.020061435
7	1.304230468	-0.913580329	1.553060554
1	1.399230500	-0.983580350	0.538060193
1	1.932230690	-0.270580096	2.075060741
16	4.597231642	4.528421619	11.668064166
6	3.003231074	4.660421666	10.837063873
8	3.149231124	4.760421698	9.644063446
6	1.649230592	4.683421673	11.505064111
6	0.543230196	4.627421654	10.454063732
6	0.481230171	3.279421169	9.707063469
6	0.022230006	3.356421200	8.264062951
6	-1.128770405	4.291421534	7.979062849
1	1.566230558	3.852421374	12.225064364
1	1.593230568	5.603422001	12.117064330
1	-0.415770146	4.833421726	10.958063913
1	0.716230257	5.449421944	9.739063479
1	1.450230518	2.756420985	9.749063484
1	-0.222770080	2.612420933	10.243063659
1	0.984230349	3.643421301	7.562062699
1	-1.421770509	4.276421528	6.925062474
1	-0.904770321	5.329421906	8.258062949
1	-2.010770716	3.964421416	8.566063058
6	3.282231174	4.086421458	0.620060219
6	2.802231002	3.874421384	2.077060740
8	1.916230682	2.986421065	2.219060793
8	3.240231159	4.551421625	3.058061092
1	2.634230941	4.853421732	0.160060057
1	3.052231088	3.147421127	0.097060035
26	2.686230962	4.334421546	5.272061881
8	2.130230761	3.489421244	6.728062405
17	0.581230208	5.078421812	4.674061670
8	2.785230992	0.684420243	3.264061164
1	3.690231320	0.614420221	2.862061020
1	2.455230879	1.586420565	3.031061082
8	-1.901770681	2.491420888	5.440061941
1	-2.347770839	1.840420659	6.056062164
1	-0.969770348	2.520420902	5.699062036
8	-0.276770100	5.739422051	1.519060545
1	-0.455770162	4.799421712	1.739060620

1	-0.059770020	5.804422073	0.556060199
8	-0.830770297	3.220421149	2.659060949
1	-0.879770316	3.650421305	3.535061263
1	0.128230046	3.023421080	2.562060917
8	1.252230447	7.818422792	2.422060864
1	0.772230275	6.956422486	2.422060864
1	1.041230370	8.184422923	1.534060550
8	3.887231389	7.256422594	2.298060823
1	3.816231366	6.295422247	2.468060882
1	2.943231052	7.560422700	2.355060843
1	6.376553592	11.316202100	2.393588810
1	2.792330894	9.323282114	9.182473874
1	-2.500218343	8.757501971	8.248379704
1	3.104794354	8.473786367	-1.388603848
1	7.516613740	6.568260242	2.071352611
1	-0.141889407	-1.880771832	8.855100835
1	5.529604008	-1.600371519	9.746834124
1	7.834563053	1.249205363	9.619016344
1	7.901552230	0.304114863	6.354244516
1	-4.090336105	-2.915425100	1.484529041
1	4.382309869	4.406879648	13.025776879
1	-0.171740830	2.359136783	7.869263719
1	4.319075014	4.343883356	0.403876183

^{1}Re$_{\text{model 2, A}}$

7	-1.917982000	-5.266088000	7.389109000
1	-1.633823000	-6.136235000	7.853732000
6	-2.466295000	-4.142589000	7.970374000
6	-1.955138000	-5.132222000	6.054617000
1	-1.590546000	-5.882549000	5.361959000
7	-2.480065000	-3.951719000	5.730583000
6	-2.817504000	-3.323982000	6.919532000
1	-3.294312000	-2.346662000	6.913840000
7	-3.929440000	-5.856102000	1.487118000
1	-3.866052000	-6.549846000	0.732989000
6	-5.084760000	-5.465414000	2.141371000
6	-2.884966000	-5.208870000	2.055153000
1	-1.854661000	-5.352465000	1.736389000
7	-3.295407000	-4.395930000	3.020118000
6	-4.668524000	-4.544813000	3.086693000
1	-5.274511000	-3.957063000	3.777033000
16	-4.853280000	5.505525000	7.219587000
6	-3.382903000	5.558280000	6.092401000
8	-3.122642000	6.428555000	5.305852000
6	-2.543873000	4.320783000	6.332923000
6	-2.713511000	3.320017000	5.153916000
6	-4.131223000	2.782815000	4.871783000
6	-4.703015000	1.797455000	5.904114000
6	-6.130729000	1.349561000	5.579933000
1	-1.487848000	4.644890000	6.366469000
1	-2.801361000	3.853369000	7.300665000
1	-2.332997000	3.826729000	4.249764000
1	-2.027559000	2.472633000	5.345218000
1	-4.840337000	3.625966000	4.735742000
1	-4.096168000	2.264196000	3.892937000
1	-4.049245000	0.902465000	5.927795000
1	-6.499364000	0.635133000	6.340892000
1	-6.182745000	0.843703000	4.597655000
1	-6.830533000	2.208250000	5.554495000
6	1.524309000	-4.092083000	2.322917000
6	0.281550000	-4.058642000	3.196139000
8	-0.739059000	-3.357259000	2.741978000
8	0.240257000	-4.653207000	4.270874000
1	2.393266000	-3.972944000	2.995942000
1	1.535774000	-3.283934000	1.572111000
26	-2.028281000	-2.877344000	4.135061000
8	-1.082630000	-1.874410000	4.902576000
17	-3.568521000	-1.447182000	3.417170000
1	-2.507319000	-3.937822000	9.041893000
1	-6.102614000	-5.720635000	1.840372000
1	-5.885013000	6.117565000	6.533142000
1	-4.685119000	2.229324000	6.906577000
1	1.614036000	-5.054429000	1.815371000

^{3}Re$_{\text{model 2, A}}$

7	-1.856266000	-5.289870000	7.556840000
1	-1.532934000	-6.112398000	8.075809000
6	-2.458325000	-4.163918000	8.069837000

6	-1.918595000	-5.244582000	6.218300000
1	-1.515306000	-6.021118000	5.574344000
7	-2.507679000	-4.119822000	5.823720000
6	-2.865709000	-3.437897000	6.970539000
1	-3.395752000	-2.489625000	6.897715000
7	-3.940992000	-5.887024000	1.403662000
1	-3.907028000	-6.566289000	0.632259000
6	-5.075424000	-5.485596000	2.088248000
6	-2.870330000	-5.286140000	1.962767000
1	-1.848255000	-5.439729000	1.621923000
7	-3.250488000	-4.491817000	2.958522000
6	-4.627422000	-4.599149000	3.047355000
1	-5.202949000	-4.009393000	3.761748000
16	-4.842802000	5.513670000	7.229338000
6	-3.368112000	5.575217000	6.110057000
8	-3.116598000	6.436621000	5.310881000
6	-2.512570000	4.353730000	6.373987000
6	-2.636245000	3.351158000	5.191301000
6	-4.049156000	2.834906000	4.852332000
6	-4.668453000	1.844175000	5.851236000
6	-6.112364000	1.467483000	5.506609000
1	-1.463913000	4.696772000	6.436174000
1	-2.786591000	3.881939000	7.334688000
1	-2.212141000	3.850798000	4.302924000
1	-1.970283000	2.496005000	5.415541000
1	-4.740849000	3.690204000	4.703043000
1	-3.989413000	2.327999000	3.868993000
1	-4.052664000	0.923140000	5.843680000
1	-6.514388000	0.740916000	6.238535000
1	-6.180043000	0.999412000	4.506795000
1	-6.777435000	2.353862000	5.510486000
6	1.629019000	-4.140118000	2.479931000
6	0.259608000	-3.960092000	3.096557000
8	-0.632059000	-3.263227000	2.473876000
8	-0.036155000	-4.466308000	4.202947000
1	2.385803000	-4.183958000	3.284562000
1	1.862495000	-3.299716000	1.804552000
26	-2.054870000	-3.161747000	4.024018000
8	-1.085084000	-1.992429000	4.867033000
17	-3.443036000	-1.441462000	3.463095000
1	-2.506649000	-3.928907000	9.134825000
1	-6.102945000	-5.732869000	1.814711000
1	-5.876785000	6.119148000	6.540463000
1	-4.638183000	2.241284000	6.867677000
1	1.674555000	-5.073287000	1.915230000

$^{5}\mathbf{Re}_{\text{model 2, A}}$

7	-1.870507000	-5.243572000	7.492548000
1	-1.533848000	-6.076734000	7.988158000
6	-2.469020000	-4.132112000	8.041936000
6	-1.956656000	-5.168803000	6.155906000
1	-1.557302000	-5.924392000	5.486011000
7	-2.558809000	-4.036846000	5.799010000
6	-2.897030000	-3.382071000	6.967844000
1	-3.428428000	-2.433247000	6.928391000
7	-3.998821000	-5.867320000	1.381310000
1	-3.954382000	-6.538239000	0.603053000
6	-5.138309000	-5.494385000	2.073657000
6	-2.938250000	-5.259804000	1.952047000
1	-1.913985000	-5.394214000	1.610371000
7	-3.329602000	-4.490027000	2.961787000
6	-4.703990000	-4.623608000	3.050785000
1	-5.291068000	-4.054798000	3.772605000
16	-4.870870000	5.502954000	7.219524000
6	-3.394656000	5.553833000	6.100864000
8	-3.126075000	6.425632000	5.318285000
6	-2.565632000	4.309685000	6.339650000
6	-2.737591000	3.320604000	5.151212000
6	-4.154982000	2.789580000	4.861582000
6	-4.716135000	1.778086000	5.872882000
6	-6.146981000	1.339833000	5.550525000
1	-1.507139000	4.625048000	6.380874000
1	-2.831611000	3.836835000	7.302317000
1	-2.359731000	3.839580000	4.253501000
1	-2.052485000	2.470570000	5.331681000
1	-4.866996000	3.633699000	4.748674000
1	-4.122963000	2.293384000	3.870874000
1	-4.062404000	0.883283000	5.861132000
1	-6.511638000	0.609065000	6.297784000
1	-6.206071000	0.855519000	4.557887000
1	-6.845428000	2.200054000	5.549267000

6	1.522023000	-4.102648000	2.339417000
6	0.177618000	-3.998273000	3.035861000
8	-0.727769000	-3.220329000	2.527518000
8	-0.060420000	-4.634465000	4.079832000
1	2.314190000	-4.084039000	3.112050000
1	1.680851000	-3.264102000	1.641493000
26	-2.178766000	-3.044618000	4.011449000
8	-1.274378000	-1.973191000	4.825181000
17	-3.664031000	-1.465602000	3.305406000
1	-2.499129000	-3.913574000	9.111094000
1	-6.162494000	-5.742578000	1.788680000
1	-5.897087000	6.120028000	6.529325000
1	-4.686813000	2.182712000	6.886382000
1	1.600899000	-5.046516000	1.796595000

^{5}TS$_{\text{model 2, A}}$

7	-1.967086000	-5.233085000	7.345245000
1	-1.644027000	-6.114039000	7.762644000
6	-2.480951000	-4.131078000	7.995560000
6	-2.059474000	-5.032253000	6.018212000
1	-1.730477000	-5.755635000	5.278564000
7	-2.577770000	-3.831224000	5.763894000
6	-2.858170000	-3.266264000	6.992239000
1	-3.308676000	-2.278793000	7.054201000
7	-4.055434000	-5.773101000	1.346758000
1	-3.971850000	-6.455679000	0.584831000
6	-5.222768000	-5.420306000	2.000347000
6	-3.033389000	-5.103373000	1.927376000
1	-1.996617000	-5.209098000	1.612848000
7	-3.474774000	-4.316473000	2.899641000
6	-4.842452000	-4.502264000	2.957219000
1	-5.470383000	-3.938877000	3.648020000
16	-4.858570000	5.224287000	7.124023000
6	-3.371910000	5.357686000	6.037265000
8	-3.149527000	6.210010000	5.219172000
6	-2.390121000	4.250305000	6.406237000
6	-1.932087000	3.404566000	5.183939000
6	-2.755011000	2.133870000	4.857196000
6	-2.569918000	0.951114000	5.823616000
6	-3.839620000	0.508807000	6.525657000
1	-1.512734000	4.789904000	6.810190000
1	-2.786202000	3.618328000	7.219441000
1	-1.908868000	4.066568000	4.301415000
1	-0.881445000	3.106694000	5.358675000
1	-3.834605000	2.377707000	4.791967000
1	-2.478687000	1.807902000	3.839447000
1	-2.143549000	-0.062287000	5.243554000
1	-3.648071000	-0.267996000	7.283853000
1	-4.579631000	0.114065000	5.804132000
1	-4.307282000	1.369955000	7.049037000
6	1.397374000	-4.002503000	2.206222000
6	0.142967000	-3.901655000	3.075128000
8	-0.795301000	-3.071230000	2.684523000
8	0.049926000	-4.551246000	4.121156000
1	2.258222000	-3.813165000	2.874699000
1	1.409249000	-3.245402000	1.404457000
26	-2.358138000	-2.675592000	3.984733000
8	-1.676871000	-1.350803000	4.912596000
17	-3.920191000	-1.244640000	3.018501000
1	-2.494769000	-3.958335000	9.073398000
1	-6.232540000	-5.703411000	1.697068000
1	-5.872610000	5.944706000	6.521534000
1	-1.735356000	1.091253000	6.513277000
1	1.531785000	-4.993668000	1.768856000

^{5}Int$_{\text{model 2, A}}$

7	-1.877647000	-5.278535000	7.523479000
1	-1.555666000	-6.113381000	8.025509000
6	-2.455114000	-4.151260000	8.059337000
6	-1.950775000	-5.207694000	6.185514000
1	-1.571137000	-5.981469000	5.522457000
7	-2.525907000	-4.066811000	5.813918000
6	-2.859812000	-3.401092000	6.976399000
1	-3.369924000	-2.440452000	6.931648000
7	-3.970755000	-5.829825000	1.257085000
1	-3.957918000	-6.472765000	0.455232000
6	-5.081680000	-5.478894000	2.001766000
6	-2.886431000	-5.252948000	1.820403000

1	-1.875492000	-5.375730000	1.435642000
7	-3.232968000	-4.527888000	2.875694000
6	-4.604740000	-4.655083000	3.001887000
1	-5.160216000	-4.118210000	3.772344000
16	-4.918449000	5.443840000	7.198509000
6	-3.412344000	5.608162000	6.150977000
8	-3.181968000	6.446825000	5.319583000
6	-2.467882000	4.491399000	6.540007000
6	-2.421054000	3.375839000	5.463121000
6	-3.764884000	2.736355000	5.058521000
6	-4.564967000	2.062013000	6.131973000
6	-5.791123000	1.281071000	5.785831000
1	-1.463254000	4.941480000	6.631045000
1	-2.752386000	4.077762000	7.522068000
1	-1.945607000	3.797387000	4.560461000
1	-1.736023000	2.594178000	5.844048000
1	-4.396108000	3.504288000	4.547098000
1	-3.548236000	1.995784000	4.255666000
1	-1.470251000	-0.992070000	5.071219000
1	-6.288755000	0.877023000	6.686599000
1	-5.555332000	0.421205000	5.118286000
1	-6.537750000	1.893418000	5.230939000
6	1.633100000	-4.121995000	2.464463000
6	0.249125000	-3.901003000	3.024102000
8	-0.591697000	-3.154388000	2.397234000
8	-0.117313000	-4.422373000	4.109755000
1	2.360481000	-4.179674000	3.295557000
1	1.909493000	-3.289692000	1.795787000
26	-1.971437000	-3.054655000	4.035810000
8	-0.914407000	-1.786459000	4.968198000
17	-3.509520000	-1.437558000	3.429331000
1	-2.490260000	-3.922836000	9.126273000
1	-6.113430000	-5.723352000	1.741963000
1	-5.937694000	6.083000000	6.518150000
1	-4.264496000	2.136027000	7.178875000
1	1.676656000	-5.059841000	1.907405000

^{5}TSCl$_{model\ 2,\ A}$

7	-1.929062000	-5.258958000	7.521283000
1	-1.607972000	-6.114963000	7.987778000
6	-2.432980000	-4.121768000	8.103727000
6	-2.046257000	-5.140761000	6.185740000
1	-1.715916000	-5.907849000	5.488851000
7	-2.583355000	-3.969214000	5.863640000
6	-2.837256000	-3.325926000	7.053767000
1	-3.281773000	-2.333168000	7.057348000
7	-3.986831000	-5.746901000	1.333980000
1	-3.919704000	-6.445412000	0.584167000
6	-5.136558000	-5.395431000	2.017585000
6	-2.950512000	-5.064413000	1.874429000
1	-1.924307000	-5.162941000	1.525698000
7	-3.362432000	-4.269078000	2.854873000
6	-4.727724000	-4.464001000	2.955360000
1	-5.330169000	-3.903825000	3.670991000
16	-5.045322000	5.188258000	7.079622000
6	-3.534985000	5.211283000	6.003832000
8	-3.198840000	6.087580000	5.255911000
6	-2.739739000	3.939436000	6.273227000
6	-2.623374000	3.029488000	5.022698000
6	-3.893292000	2.290722000	4.540764000
6	-4.360697000	1.101533000	5.327746000
6	-5.813507000	0.849880000	5.480420000
1	-1.719756000	4.269718000	6.547202000
1	-3.159160000	3.392002000	7.135760000
1	-2.243331000	3.652302000	4.194927000
1	-1.832337000	2.284437000	5.225371000
1	-4.748015000	2.991096000	4.425686000
1	-3.692385000	1.923573000	3.513640000
1	-1.376446000	-0.886182000	5.174116000
1	-6.031249000	-0.129827000	5.930689000
1	-6.363584000	0.956920000	4.528509000
1	-6.214608000	1.632864000	6.169332000
6	1.578674000	-4.078223000	2.411511000
6	0.182101000	-3.838267000	2.961149000
8	-0.615968000	-3.045645000	2.346176000
8	-0.195676000	-4.400145000	4.023750000
1	2.296086000	-4.128497000	3.252434000
1	1.868512000	-3.252653000	1.740521000
26	-2.005021000	-2.891128000	4.112082000
8	-0.819313000	-1.657785000	4.979846000
17	-3.574732000	-0.933062000	3.799689000

1	-2.449257000	-3.916104000	9.175735000
1	-6.152077000	-5.686667000	1.742510000
1	-5.997387000	5.970999000	6.454192000
1	-3.711465000	0.685532000	6.100536000
1	1.636398000	-5.021154000	1.864416000

^{5}TSOH$_{\text{model 2, A}}$

7	-1.911564000	-5.300451000	7.491311000
1	-1.597382000	-6.169462000	7.940243000
6	-2.491580000	-4.207646000	8.089654000
6	-1.989213000	-5.146857000	6.156607000
1	-1.608745000	-5.878080000	5.448911000
7	-2.561554000	-3.986364000	5.849226000
6	-2.889720000	-3.396446000	7.050592000
1	-3.374107000	-2.425314000	7.080151000
7	-3.994184000	-5.854240000	1.301772000
1	-3.951893000	-6.519416000	0.520493000
6	-5.133692000	-5.479978000	1.991506000
6	-2.931342000	-5.269809000	1.902143000
1	-1.903662000	-5.420833000	1.573846000
7	-3.319074000	-4.515880000	2.919487000
6	-4.693389000	-4.632166000	2.986771000
1	-5.279314000	-4.067578000	3.713014000
16	-4.770560000	5.494726000	7.393514000
6	-3.572067000	5.079139000	5.965295000
8	-3.049320000	5.921673000	5.297222000
6	-3.400990000	3.579825000	5.886700000
6	-2.342598000	3.048980000	4.914999000
6	-2.568117000	1.544689000	4.580933000
6	-2.426719000	0.634828000	5.755264000
6	-3.639816000	0.093434000	6.409278000
1	-3.251541000	3.210677000	6.920836000
1	-4.409951000	3.203988000	5.613432000
1	-2.383231000	3.626954000	3.974965000
1	-1.328224000	3.206404000	5.329110000
1	-3.565983000	1.411759000	4.124387000
1	-1.846122000	1.246671000	3.804224000
1	-0.780970000	-0.893297000	4.472428000
1	-3.432918000	-0.390872000	7.375070000
1	-4.142895000	-0.630840000	5.734976000
1	-4.388259000	0.897368000	6.585179000
6	1.535551000	-4.051925000	2.373434000
6	0.164417000	-3.854945000	3.006890000
8	-0.660240000	-3.019981000	2.475968000
8	-0.158030000	-4.476747000	4.045052000
1	2.296244000	-4.043000000	3.177618000
1	1.757459000	-3.238149000	1.663217000
26	-2.185830000	-2.909854000	4.025628000
8	-1.235642000	-1.550408000	5.027617000
17	-3.701628000	-1.378983000	3.058504000
1	-2.532929000	-4.001668000	9.160928000
1	-6.157612000	-5.724198000	1.702182000
1	-5.815745000	6.041518000	6.673180000
1	-1.472687000	0.633612000	6.285910000
1	1.614006000	-5.009826000	1.855713000

^{5}PrCl$_{\text{model 2, A}}$

7	-1.768456000	-5.291797000	7.631414000
1	-1.368329000	-6.057860000	8.175648000
6	-2.411283000	-4.183437000	8.121497000
6	-1.887709000	-5.300865000	6.292106000
1	-1.457045000	-6.079236000	5.664162000
7	-2.559897000	-4.235441000	5.875201000
6	-2.899138000	-3.528596000	7.009866000
1	-3.443845000	-2.588480000	6.933285000
7	-4.098051000	-5.685595000	1.213301000
1	-4.050296000	-6.355491000	0.435998000
6	-5.216824000	-5.396961000	1.971780000
6	-3.050467000	-5.010626000	1.734956000
1	-2.040261000	-5.060354000	1.334140000
7	-3.429170000	-4.278371000	2.780151000
6	-4.783730000	-4.513161000	2.941770000
1	-5.362936000	-4.018121000	3.722519000
16	-4.789247000	5.432133000	7.289153000
6	-3.362556000	5.515729000	6.085688000
8	-3.106206000	6.445878000	5.371546000
6	-2.574612000	4.224154000	6.128789000
6	-2.913561000	3.377585000	4.869249000

6	-4.362140000	2.863861000	4.743542000
6	-4.693020000	1.591408000	5.529296000
6	-6.178365000	1.281721000	5.599667000
1	-1.502385000	4.489306000	6.089188000
1	-2.772917000	3.667958000	7.061246000
1	-2.676108000	4.004468000	3.994472000
1	-2.216018000	2.521661000	4.836904000
1	-5.082218000	3.640073000	5.067284000
1	-4.587492000	2.667668000	3.679066000
1	-1.862282000	-1.022905000	5.132592000
1	-6.358297000	0.334449000	6.138104000
1	-6.629615000	1.211081000	4.595040000
1	-6.688810000	2.094496000	6.152454000
6	1.668755000	-4.128774000	2.416072000
6	0.259863000	-3.907091000	2.908743000
8	-0.560960000	-3.211454000	2.232088000
8	-0.115902000	-4.436663000	4.002093000
1	2.365796000	-4.194289000	3.271413000
1	1.972826000	-3.303786000	1.751125000
26	-1.944903000	-3.255454000	4.073012000
8	-1.216521000	-1.676241000	4.818154000
17	-3.869296000	0.158501000	4.600245000
1	-2.456095000	-3.927515000	9.181808000
1	-6.234952000	-5.697854000	1.717530000
1	-5.835072000	6.048642000	6.628517000
1	-4.303045000	1.652548000	6.547110000
1	1.706383000	-5.071861000	1.867497000

^{5}PrOH$_{model\ 2,\ A}$

7	-1.958947000	-5.325410000	7.558519000
1	-1.714132000	-6.192796000	8.051490000
6	-2.415604000	-4.147670000	8.095118000
6	-1.989442000	-5.228006000	6.217768000
1	-1.675805000	-6.034596000	5.557329000
7	-2.420568000	-4.027486000	5.845906000
6	-2.703623000	-3.338979000	7.009026000
1	-3.095045000	-2.319758000	6.969231000
7	-4.162776000	-5.771215000	1.113242000
1	-4.160366000	-6.384425000	0.288440000
6	-5.259071000	-5.462208000	1.895648000
6	-3.072117000	-5.214664000	1.681222000
1	-2.066162000	-5.296837000	1.273684000
7	-3.406388000	-4.549821000	2.778660000
6	-4.772910000	-4.687178000	2.926453000
1	-5.315533000	-4.191990000	3.733424000
16	-4.867654000	5.423500000	7.241078000
6	-3.372610000	5.598576000	6.172895000
8	-3.165255000	6.442659000	5.341602000
6	-2.409718000	4.485221000	6.523236000
6	-2.368576000	3.409703000	5.405306000
6	-3.666842000	2.617912000	5.156280000
6	-4.035296000	1.554944000	6.216990000
6	-5.449628000	1.008857000	5.988959000
1	-1.412011000	4.951577000	6.605000000
1	-2.666582000	4.040765000	7.500068000
1	-2.062187000	3.914843000	4.471421000
1	-1.556802000	2.705743000	5.659027000
1	-4.520958000	3.312773000	5.033336000
1	-3.566056000	2.085673000	4.190349000
1	-3.248421000	-0.062178000	5.375816000
1	-5.701068000	0.261022000	6.762061000
1	-5.536805000	0.525470000	4.996430000
1	-6.200937000	1.820648000	6.036874000
6	1.701465000	-4.094494000	2.543232000
6	0.300459000	-3.794106000	3.033610000
8	-0.516591000	-3.136342000	2.310831000
8	-0.098119000	-4.205725000	4.169042000
1	2.399645000	-4.156615000	3.397392000
1	2.032476000	-3.289177000	1.866482000
26	-2.111028000	-3.278435000	3.883682000
8	-3.067603000	0.466827000	6.186311000
17	-3.244128000	-1.289802000	3.475126000
1	-2.487201000	-3.944449000	9.165323000
1	-6.288093000	-5.729958000	1.648287000
1	-5.897843000	6.051414000	6.566757000
1	-4.026686000	2.019385000	7.204909000
1	1.729864000	-5.046200000	2.009178000

^{5}Re$_{model\ 2,\ AB}$

6	3.209629738	-8.429504090	7.097542284

1	3.666948437	-8.549599464	6.094152655
6	2.065915934	-9.448509513	7.228515527
1	1.350286579	-9.326365418	6.396411159
1	1.500217316	-9.317525078	8.169257159
1	2.403161431	-10.500081546	7.202549424
6	2.740644877	-6.963291990	7.219127145
1	2.261552874	-6.791401620	8.203767014
1	3.612008170	-6.282593329	7.181694628
6	1.752656893	-6.526963679	6.135739181
1	1.487320070	-5.469012588	6.276008449
1	2.193894139	-6.617025492	5.125606770
1	0.816984181	-7.116593009	6.173977990
6	-2.498585117	-3.837637903	9.521098069
1	-3.202448179	-3.004750585	9.538236818
1	-1.539756535	-3.483584064	9.930455057
7	-1.972088664	-5.303794360	7.440656770
1	-1.714431224	-6.194099616	7.885462935
6	-2.418707018	-4.148241426	8.053649837
6	-2.059979872	-5.162293051	6.109616616
1	-1.770945867	-5.933655460	5.403140356
7	-2.515701112	-3.953874416	5.808258227
6	-2.751212743	-3.312960079	7.007058314
1	-3.150928619	-2.303471692	7.038037456
6	-1.332854634	0.522519231	9.178923836
1	-0.721433471	1.439146949	9.251744060
6	-0.381407857	-0.632034442	9.520745525
1	0.031111087	-0.545637064	10.541274730
1	0.476508513	-0.625265836	8.822009720
1	-0.870635468	-1.618279903	9.438781669
6	-1.873872239	0.426874422	7.749551447
1	-2.576575942	-0.413570102	7.613809937
1	-1.039731512	0.279042831	7.039283203
1	-2.398317882	1.343716404	7.437317386
6	-8.783877726	0.906572583	-0.791758958
1	-8.776631988	1.556157336	0.106204397
1	-9.162371135	-0.090981995	-0.494677676
8	-7.506139179	0.845814520	-1.401930966
1	-6.865351246	0.471446456	-0.751206402
6	5.626016324	-6.054334951	4.546569181
1	5.522953456	-5.922048021	3.455962875

1	4.866491653	-6.796625610	4.860085280
6	5.236033859	-4.723787705	5.249089259
8	5.950298825	-4.233522039	6.151317281
8	4.139675438	-4.230211305	4.853766554
6	-11.474138643	-3.911658851	-0.273549167
1	-11.478637517	-4.354073262	0.736956658
1	-11.821185005	-4.703694696	-0.963637321
6	-10.074122532	-3.451358731	-0.642222628
6	-9.113604895	-3.242831224	0.360556136
1	-9.374680084	-3.426859163	1.407013782
6	-7.831854761	-2.777463416	0.045777954
1	-7.106156496	-2.609746042	0.847428770
6	-7.492120453	-2.500199049	-1.281572617
1	-6.499710955	-2.114727836	-1.530646484
6	-9.707753093	-3.198929123	-1.975672887
1	-10.429296749	-3.352232937	-2.785315126
6	-8.439870212	-2.709191338	-2.286410843
1	-8.206923895	-2.462010195	-3.322947961
6	-9.570456230	0.515760034	6.803468152
1	-10.351500518	0.357918394	7.564214440
1	-8.601281952	0.546185641	7.330658309
6	-9.822902263	1.837680177	6.080357930
1	-9.385462997	2.665755258	6.650015968
1	-9.303360697	1.895172354	5.106065889
6	-11.275387198	2.273384621	5.818369185
8	-12.232296232	1.491566067	6.065058664
8	-11.404698083	3.407528721	5.291095630
6	-6.451071993	-5.853874322	1.743152494
1	-6.804069922	-5.131722900	0.979754753
1	-7.083139498	-5.629731233	2.611624167
7	-3.895616076	-5.828049149	1.377265363
1	-3.858193867	-6.466858536	0.576538623
6	-5.032001813	-5.521587782	2.110427292
6	-2.838193089	-5.203276655	1.930496322
1	-1.826264925	-5.278889538	1.543410842
7	-3.217791165	-4.501628840	2.988325818
6	-4.581061504	-4.694391363	3.118059161
1	-5.161963337	-4.190907294	3.888402257
6	-5.675174255	3.919444017	-1.139280130
1	-6.295000678	3.021488784	-1.325808395

1	-5.075325131	4.087449486	-2.054260307
6	-4.710955162	3.646746810	0.018355217
1	-4.081031830	4.525579767	0.244971362
1	-5.254076035	3.389799293	0.948776278
7	-3.843598360	2.518054209	-0.362846727
1	-4.102946600	2.002758634	-1.212970877
6	-2.888246128	1.962672415	0.398254141
7	-2.469936307	2.564433579	1.526098449
1	-2.520674155	3.588244206	1.641593791
1	-1.730250661	2.108132837	2.059239712
7	-2.386718708	0.761472070	0.080670452
1	-2.600072783	0.322346205	-0.819203741
1	-1.565044656	0.383552967	0.595251463
16	-4.818075898	5.534959752	7.245404266
6	-3.375830172	5.570683064	6.078123013
8	-3.120543055	6.454820637	5.308142959
6	-2.568394486	4.309162794	6.249538458
6	-2.862917827	3.339081009	5.070493520
6	-4.294111305	2.799110449	4.915603429
6	-4.742485214	1.776420555	5.967197196
6	-6.177466781	1.295576451	5.758997059
1	-1.502702877	4.592258059	6.206048375
1	-2.775073461	3.836927652	7.225261441
1	-2.581957384	3.872835445	4.148665128
1	-2.159287195	2.494174006	5.167355029
1	-5.023287163	3.632645109	4.874825459
1	-4.345301864	2.312988643	3.923205563
1	-4.063411958	0.906457042	5.918965812
1	-6.452848330	0.546167342	6.521863135
1	-6.305898285	0.825085351	4.767465423
1	-6.898479636	2.130756562	5.827679789
6	1.587667322	-4.075703285	2.409490898
6	0.230814448	-3.894467856	3.053079999
8	-0.657827350	-3.159523463	2.470438412
8	-0.057596223	-4.430045922	4.146164818
1	2.366331451	-4.083986079	3.195579537
1	1.788054681	-3.255137997	1.702709361
26	-2.097092041	-3.023517004	4.012962152
8	-1.243512347	-1.893859841	4.799430978
17	-3.578852474	-1.467204905	3.173308340
8	-4.320617036	-2.577130869	0.335787809
1	-3.440531860	-2.391704955	-0.038583022
1	-4.200807074	-2.451678496	1.306798576
8	0.753508540	-3.049194896	6.621627128
1	0.544731028	-3.550360730	5.802099017
1	-0.107256084	-2.708751550	6.928509921
8	-0.421238965	-0.346990047	1.721836943
1	-0.579609608	-1.271551873	2.005088215
1	0.564324546	-0.274962679	1.606152436
8	1.342925734	-0.266494038	5.509324978
1	1.040119992	-1.141158143	5.826267505
1	2.158491583	-0.463727242	4.993484673
1	4.053943776	-8.676601470	7.741082500
1	-2.816421890	-4.641998381	10.184461719
1	-2.112292932	0.606307082	9.936236753
1	-9.518665943	1.365917205	-1.452936784
1	6.626062056	-6.434411700	4.755177711
1	-12.260725613	-3.160223507	-0.342055993
1	-9.556867503	-0.328605535	6.114316896
1	-6.758925287	-6.823876603	1.352781851
1	-6.349195562	4.768347202	-1.024711456
1	-5.858966854	6.118845579	6.552604883
1	-4.650942371	2.188648637	6.972062976
1	1.646911657	-5.026871339	1.880494898

^{5}TS$_{\text{model 2, AB}}$

6	3.221705000	-8.448119000	7.117569000
1	3.678518000	-8.558520000	6.110931000
6	2.077128000	-9.470592000	7.239882000
1	1.361043000	-9.341592000	6.406967000
1	1.510040000	-9.347296000	8.182729000
1	2.415130000	-10.523535000	7.205169000
6	2.755224000	-6.982926000	7.256911000
1	2.279037000	-6.819453000	8.246223000
1	3.629163000	-6.301886000	7.224527000
6	1.765180000	-6.533626000	6.184601000
1	1.504473000	-5.477523000	6.338177000
1	2.199191000	-6.607175000	5.168822000
1	0.824681000	-7.118699000	6.218928000
6	-2.438382000	-3.794304000	9.390467000
1	-3.122465000	-2.943996000	9.449237000

1	-1.470731000	-3.471711000	9.809136000
7	-2.038725000	-5.193453000	7.210614000
1	-1.810016000	-6.120279000	7.603515000
6	-2.374305000	-4.037360000	7.901199000
6	-2.137087000	-4.958559000	5.886755000
1	-1.932372000	-5.710590000	5.129917000
7	-2.483468000	-3.695538000	5.659787000
6	-2.642988000	-3.115627000	6.905423000
1	-2.956770000	-2.079342000	7.000029000
6	-1.035757000	0.575887000	9.367502000
1	-0.421930000	1.482033000	9.523989000
6	-0.147220000	-0.606024000	9.773561000
1	0.153397000	-0.552878000	10.835972000
1	0.780378000	-0.600656000	9.169462000
1	-0.644077000	-1.581971000	9.611411000
6	-1.429499000	0.504108000	7.890228000
1	-2.087761000	-0.354848000	7.668414000
1	-0.524042000	0.391206000	7.263652000
1	-1.941451000	1.418865000	7.545350000
6	-8.799705000	0.961254000	-0.791949000
1	-8.802056000	1.629553000	0.093586000
1	-9.164024000	-0.036434000	-0.474463000
8	-7.515806000	0.907702000	-1.395177000
1	-6.869557000	0.629973000	-0.699506000
6	5.650238000	-6.058937000	4.572808000
1	5.529170000	-5.943181000	3.480530000
1	4.885703000	-6.786041000	4.913844000
6	5.299218000	-4.709360000	5.262044000
8	6.026869000	-4.233492000	6.164822000
8	4.219045000	-4.182864000	4.861778000
6	-11.598267000	-3.945412000	-0.232425000
1	-11.585549000	-4.351725000	0.794451000
1	-11.981240000	-4.751435000	-0.888218000
6	-10.210212000	-3.507470000	-0.664697000
6	-9.244541000	-3.153180000	0.293960000
1	-9.470106000	-3.269595000	1.359636000
6	-8.012836000	-2.613354000	-0.094003000
1	-7.277116000	-2.332522000	0.666101000
6	-7.735683000	-2.406334000	-1.450801000
1	-6.800417000	-1.927089000	-1.749907000
6	-9.884792000	-3.367326000	-2.025249000
1	-10.609336000	-3.652827000	-2.797291000
6	-8.669925000	-2.801340000	-2.411622000
1	-8.474297000	-2.643525000	-3.473747000
6	-9.525378000	0.528041000	6.784731000
1	-10.277881000	0.390802000	7.582127000
1	-8.531192000	0.562486000	7.271938000
6	-9.799193000	1.838166000	6.039786000
1	-9.324903000	2.679042000	6.566222000
1	-9.322242000	1.862685000	5.039080000
6	-11.265069000	2.278695000	5.823067000
8	-12.218910000	1.494964000	6.082077000
8	-11.407274000	3.418416000	5.306139000
6	-6.589319000	-5.816039000	1.581357000
1	-6.993355000	-5.132538000	0.804475000
1	-7.232581000	-5.616958000	2.450299000
7	-4.063029000	-5.537730000	1.156676000
1	-3.997854000	-6.116832000	0.309330000
6	-5.197599000	-5.386196000	1.939073000
6	-3.050977000	-4.848901000	1.733447000
1	-2.048123000	-4.789071000	1.315138000
7	-3.462634000	-4.250036000	2.845462000
6	-4.796207000	-4.581987000	2.989507000
1	-5.405217000	-4.196478000	3.807487000
6	-5.719228000	3.937154000	-1.165298000
1	-6.332148000	3.037604000	-1.372121000
1	-5.102483000	4.121632000	-2.067420000
6	-4.774688000	3.670841000	0.006011000
1	-4.205735000	4.582373000	0.271842000
1	-5.331419000	3.360727000	0.914708000
7	-3.825322000	2.617654000	-0.379678000
1	-4.066678000	2.038878000	-1.194871000
6	-2.677170000	2.336927000	0.259402000
7	-2.300826000	3.070123000	1.323868000
1	-2.658923000	4.016175000	1.479678000
1	-1.350004000	2.955696000	1.700400000
7	-1.902290000	1.321276000	-0.120597000
1	-2.176996000	0.673152000	-0.866761000
1	-1.118524000	1.008018000	0.489877000
16	-5.082001000	5.227238000	7.087004000

6	-3.510800000	5.319420000	6.120841000
8	-3.170407000	6.189073000	5.365093000
6	-2.667052000	4.115026000	6.517715000
6	-2.207139000	3.267862000	5.306038000
6	-3.264811000	2.408538000	4.578324000
6	-3.703066000	1.057131000	5.147942000
6	-4.962167000	0.511900000	4.500042000
1	-1.766766000	4.532019000	7.009201000
1	-3.193964000	3.502033000	7.269255000
1	-1.772472000	3.970681000	4.572863000
1	-1.376199000	2.627532000	5.646856000
1	-4.195117000	3.007444000	4.445133000
1	-2.903726000	2.233735000	3.548233000
1	-2.855263000	0.144359000	4.920282000
1	-5.165377000	-0.522707000	4.818686000
1	-4.877326000	0.510759000	3.398564000
1	-5.842860000	1.131081000	4.771485000
6	1.608300000	-4.052417000	2.466649000
6	0.345753000	-3.893901000	3.314285000
8	-0.571046000	-3.081286000	2.875144000
8	0.245407000	-4.509845000	4.388741000
1	2.471383000	-3.971838000	3.155534000
1	1.677417000	-3.265314000	1.696415000
26	-2.320068000	-2.588771000	3.820569000
8	-2.290627000	-1.102330000	4.727788000
17	-2.950127000	-1.347419000	1.820466000
8	-4.837967000	-2.323918000	-0.356032000
1	-4.371296000	-2.060409000	-1.186479000
1	-4.149591000	-2.300001000	0.349632000
8	0.755690000	-3.100976000	6.729917000
1	0.561619000	-3.646720000	5.922911000
1	-0.113457000	-2.907609000	7.134597000
8	-0.221692000	0.194195000	1.747506000
1	-0.744705000	-0.632643000	1.813571000
1	0.742859000	-0.044980000	1.695752000
8	1.522209000	-0.563832000	5.639816000
1	1.221436000	-1.413189000	6.035745000
1	2.382524000	-0.773209000	5.205509000
1	4.067907000	-8.695953000	7.761219000
1	-2.776274000	-4.607825000	10.035276000
1	-1.880871000	0.652687000	10.054269000
1	-9.541775000	1.396689000	-1.463881000
1	6.650800000	-6.447055000	4.772819000
1	-12.358033000	-3.165973000	-0.316064000
1	-9.542638000	-0.332357000	6.113031000
1	-6.841056000	-6.814579000	1.218990000
1	-6.394118000	4.786136000	-1.040724000
1	-6.023151000	6.002960000	6.436798000
1	-3.757757000	1.011385000	6.237290000
1	1.680318000	-5.032850000	1.991961000

^{5}Int$_{\text{model 2, AB}}$

6	3.182393000	-8.485278000	7.113400000
1	3.638574000	-8.584157000	6.105147000
6	2.079976000	-9.552571000	7.249275000
1	1.362737000	-9.469569000	6.411721000
1	1.506176000	-9.433931000	8.188164000
1	2.456987000	-10.592480000	7.236013000
6	2.644843000	-7.040694000	7.240325000
1	2.164955000	-6.891108000	8.229257000
1	3.480694000	-6.314530000	7.194150000
6	1.623942000	-6.660186000	6.161201000
1	1.253598000	-5.637263000	6.338295000
1	2.078019000	-6.679795000	5.150769000
1	0.744664000	-7.333437000	6.170099000
6	-2.499068000	-3.852300000	9.519426000
1	-3.206777000	-3.019108000	9.520370000
1	-1.529073000	-3.482454000	9.896394000
7	-1.862899000	-5.329548000	7.487272000
1	-1.514114000	-6.164348000	7.970586000
6	-2.447576000	-4.215071000	8.061903000
6	-1.990307000	-5.257726000	6.153125000
1	-1.619890000	-6.022482000	5.475381000
7	-2.604188000	-4.130807000	5.807548000
6	-2.905146000	-3.478685000	6.988129000
1	-3.410682000	-2.515927000	6.985662000
6	-1.207391000	0.494031000	9.113794000
1	-0.670827000	1.456930000	9.027642000
6	-0.149479000	-0.541662000	9.531796000
1	0.349007000	-0.265867000	10.479731000
1	0.638742000	-0.605409000	8.755996000

1	-0.574718000	-1.555667000	9.654577000
6	-1.820715000	0.163536000	7.746480000
1	-2.275728000	-0.843052000	7.731416000
1	-1.016541000	0.170404000	6.985288000
1	-2.588723000	0.885914000	7.412753000
6	-8.804041000	0.909752000	-0.784485000
1	-8.803995000	1.560150000	0.115183000
1	-9.183741000	-0.090206000	-0.490414000
8	-7.519910000	0.851281000	-1.385446000
1	-6.879959000	0.497840000	-0.719649000
6	5.624494000	-6.074713000	4.545835000
1	5.527347000	-5.934129000	3.453723000
1	4.866308000	-6.825703000	4.847707000
6	5.224281000	-4.751423000	5.259638000
8	5.960242000	-4.242121000	6.134813000
8	4.101866000	-4.278659000	4.898207000
6	-11.471241000	-3.908550000	-0.286326000
1	-11.470439000	-4.358508000	0.722818000
1	-11.812765000	-4.699940000	-0.982663000
6	-10.073562000	-3.431090000	-0.653210000
6	-9.114834000	-3.219304000	0.353623000
1	-9.374816000	-3.415166000	1.400001000
6	-7.835621000	-2.737596000	0.043925000
1	-7.110288000	-2.571161000	0.848297000
6	-7.496875000	-2.445976000	-1.282888000
1	-6.507263000	-2.045354000	-1.527617000
6	-9.707608000	-3.165155000	-1.986449000
1	-10.427167000	-3.321066000	-2.799620000
6	-8.442343000	-2.658700000	-2.292074000
1	-8.208998000	-2.400961000	-3.327969000
6	-9.548720000	0.522595000	6.803328000
1	-10.315126000	0.373253000	7.584194000
1	-8.564899000	0.555426000	7.309228000
6	-9.812411000	1.841718000	6.072410000
1	-9.359480000	2.675116000	6.627993000
1	-9.306832000	1.888510000	5.087081000
6	-11.273484000	2.276795000	5.826451000
8	-12.229848000	1.492460000	6.075835000
8	-11.410525000	3.414901000	5.303547000
6	-6.527979000	-5.853581000	1.650624000
1	-6.904048000	-5.143804000	0.884638000
1	-7.149183000	-5.635636000	2.530711000
7	-3.989197000	-5.806730000	1.221673000
1	-3.975532000	-6.424241000	0.402153000
6	-5.104876000	-5.517585000	1.993139000
6	-2.910485000	-5.224465000	1.786088000
1	-1.903565000	-5.306174000	1.381932000
7	-3.257984000	-4.563910000	2.879855000
6	-4.622128000	-4.738980000	3.026788000
1	-5.180189000	-4.266474000	3.835691000
6	-5.709221000	3.902298000	-1.156334000
1	-6.336092000	3.007017000	-1.340666000
1	-5.103700000	4.063812000	-2.070444000
6	-4.755322000	3.639449000	0.011404000
1	-4.130348000	4.527856000	0.225299000
1	-5.310426000	3.402475000	0.942570000
7	-3.870681000	2.509567000	-0.328310000
1	-4.123669000	1.939733000	-1.146389000
6	-2.838736000	2.091638000	0.424312000
7	-2.485205000	2.789535000	1.520684000
1	-2.741387000	3.778032000	1.620630000
1	-1.587692000	2.581010000	1.969214000
7	-2.189754000	0.954063000	0.140150000
1	-2.347169000	0.444251000	-0.737800000
1	-1.403138000	0.623075000	0.734170000
16	-4.892895000	5.489907000	7.251983000
6	-3.402173000	5.622394000	6.170051000
8	-3.166319000	6.480449000	5.360373000
6	-2.494882000	4.454892000	6.478195000
6	-2.572872000	3.391715000	5.350463000
6	-3.955490000	2.780677000	5.041643000
6	-4.640651000	2.021502000	6.136530000
6	-5.748076000	1.069964000	5.818036000
1	-1.463303000	4.847558000	6.523794000
1	-2.744532000	4.013459000	7.458092000
1	-2.192283000	3.863650000	4.427201000
1	-1.863329000	2.587013000	5.614631000
1	-4.633770000	3.586388000	4.660372000
1	-3.823167000	2.106570000	4.170199000
1	-1.103887000	-1.054295000	5.096309000

1	-6.109790000	0.544436000	6.720892000
1	-5.430454000	0.303708000	5.079076000
1	-6.625160000	1.584577000	5.358675000
6	1.552966000	-4.111695000	2.343903000
6	0.161481000	-3.909561000	2.899540000
8	-0.643227000	-3.076479000	2.341743000
8	-0.237928000	-4.528373000	3.921537000
1	2.289492000	-4.148789000	3.171488000
1	1.810785000	-3.281739000	1.665364000
26	-2.038413000	-3.125404000	4.030102000
8	-0.921637000	-2.009200000	5.051271000
17	-3.453201000	-1.437994000	3.288653000
8	-4.357204000	-2.487733000	0.375455000
1	-3.502211000	-2.274884000	-0.043418000
1	-4.181896000	-2.419023000	1.342906000
8	1.003230000	-3.116974000	6.481657000
1	1.301620000	-3.733722000	5.785900000
1	0.167033000	-2.711915000	6.091480000
8	-0.248392000	-0.195398000	1.890554000
1	-0.443832000	-1.148424000	2.032138000
1	0.733503000	-0.151236000	1.743177000
8	1.683957000	-0.345866000	5.672559000
1	1.464530000	-1.234552000	6.026905000
1	2.520251000	-0.503034000	5.170791000
1	4.040108000	-8.705535000	7.751817000
1	-2.809483000	-4.642516000	10.205696000
1	-1.950864000	0.601107000	9.905973000
1	-9.536256000	1.368639000	-1.451605000
1	6.627992000	-6.448823000	4.757490000
1	-12.266310000	-3.163033000	-0.348258000
1	-9.549769000	-0.329766000	6.121240000
1	-6.820897000	-6.836374000	1.276349000
1	-6.375015000	4.759170000	-1.036943000
1	-5.916228000	6.099705000	6.551120000
1	-4.492066000	2.312048000	7.178293000
1	1.620328000	-5.056884000	1.801830000

^{5}PrCl$_{model\ 2,\ AB}$

6	3.213938141	-8.395026901	7.102702456
1	3.679668625	-8.492209795	6.101296574
6	2.072600046	-9.419938201	7.205689911
1	1.344461464	-9.260184114	6.391926196
1	1.520525048	-9.334000901	8.159410043
1	2.411779960	-10.468695334	7.126486587
6	2.736110770	-6.929641313	7.245964342
1	2.233632789	-6.780504209	8.222407325
1	3.608567216	-6.248944599	7.244496008
6	1.775355898	-6.468894207	6.144881872
1	1.495897520	-5.413171530	6.299660671
1	2.242506414	-6.535619685	5.145437047
1	0.845076789	-7.065687148	6.135729196
6	-2.513621297	-3.852714724	9.544717519
1	-3.233973310	-3.032182885	9.525205495
1	-1.555859790	-3.461209233	9.927525744
7	-1.784898022	-5.321962534	7.555039020
1	-1.367238566	-6.099946029	8.066860403
6	-2.447791541	-4.243157912	8.097870656
6	-1.926547950	-5.298445103	6.219786493
1	-1.499656853	-6.055563743	5.567114594
7	-2.630798915	-4.242371417	5.844390915
6	-2.969528442	-3.581410385	7.005576671
1	-3.525272077	-2.648671887	6.976789090
6	-1.206346525	0.482924157	9.140375808
1	-0.712805951	1.458551264	8.984036417
6	-0.100741293	-0.486269145	9.576863725
1	0.420320483	-0.140319820	10.486993331
1	0.656264769	-0.572838706	8.775606495
1	-0.488666714	-1.502725473	9.772182529
6	-1.845429819	0.045077133	7.820864151
1	-2.205912468	-0.996418519	7.859645382
1	-1.098342190	0.087081551	7.009726876
1	-2.695440943	0.677734866	7.515221173
6	-8.788130253	0.931879569	-0.791597988
1	-8.767753436	1.596936015	0.094554264
1	-9.168581659	-0.057518551	-0.471280882
8	-7.515242954	0.850035682	-1.408370047
1	-6.871604708	0.507949309	-0.742191825
6	5.631921598	-6.041454014	4.567310574
1	5.520026725	-5.896397963	3.478874784
1	4.867475992	-6.778488018	4.879389507
6	5.271968068	-4.714009827	5.292352638

8	6.037589793	-4.233629303	6.162007778
8	4.161924696	-4.211634162	4.958543877
6	-11.464976029	-3.882737235	-0.261304423
1	-11.458502990	-4.313692757	0.754533201
1	-11.807777252	-4.686106728	-0.940400498
6	-10.072876907	-3.409984204	-0.645845047
6	-9.111319114	-3.163104379	0.347088612
1	-9.364213810	-3.326275766	1.398768206
6	-7.838624377	-2.684228264	0.015791051
1	-7.112170781	-2.489924560	0.810352945
6	-7.510275535	-2.426472175	-1.317894015
1	-6.529090357	-2.019971717	-1.579546356
6	-9.715843461	-3.181903650	-1.986129080
1	-10.437424679	-3.366560876	-2.789185830
6	-8.458811789	-2.675570744	-2.312970349
1	-8.235915336	-2.445809471	-3.355392839
6	-9.471414993	0.518146925	6.726677644
1	-10.235657750	0.415090873	7.516383722
1	-8.483221375	0.537663527	7.222233209
6	-9.699225269	1.815997658	5.948361597
1	-9.210896678	2.651186119	6.469589763
1	-9.198874104	1.805910736	4.961135787
6	-11.146492543	2.303043032	5.704589626
8	-12.125288094	1.551202268	5.968845213
8	-11.222821366	3.461785116	5.215922345
6	-6.644248773	-5.842024186	1.597087826
1	-7.070741081	-5.166302135	0.828882870
1	-7.257238929	-5.640244599	2.484145457
7	-4.123298487	-5.669649597	1.128562888
1	-4.083717045	-6.309069967	0.329175433
6	-5.241542112	-5.424308610	1.906012243
6	-3.079722185	-4.992568471	1.645240664
1	-2.078631481	-5.007831573	1.224527754
7	-3.456584452	-4.311049437	2.719348493
6	-4.799392258	-4.580221811	2.902145122
1	-5.373477034	-4.138032433	3.714253756
6	-5.713189179	3.868259671	-1.162273405
1	-6.343352461	2.974334165	-1.330298363
1	-5.120195371	4.019506963	-2.084191366
6	-4.744325957	3.619392892	-0.007172622
1	-4.112388964	4.507133220	0.172626567
1	-5.279029860	3.401906212	0.937868850
7	-3.865602793	2.481626759	-0.333438511
1	-4.181620308	1.838419470	-1.066860514
6	-2.727632599	2.174554927	0.309809128
7	-2.232024931	3.004834492	1.229802603
1	-2.629074617	3.923920010	1.424927850
1	-1.290523391	2.879521932	1.614097694
7	-2.083729596	1.022590890	0.064090080
1	-2.285285187	0.452829932	-0.764011131
1	-1.287204306	0.719363356	0.651029439
16	-5.015712599	5.428449586	7.209773794
6	-3.447309959	5.620515548	6.284993762
8	-3.168174781	6.449746097	5.461696649
6	-2.515497796	4.525932025	6.761642850
6	-2.012276459	3.669637610	5.580407251
6	-3.059932379	3.075348831	4.612710077
6	-3.751693697	1.764448216	5.002812893
6	-4.770111923	1.828086178	6.130793131
1	-1.648853812	5.027374507	7.227709606
1	-2.997751528	3.917484442	7.540375089
1	-1.357521432	4.318933242	4.976085113
1	-1.365555851	2.869925679	5.982812338
1	-3.829196261	3.828376730	4.364631010
1	-2.528552583	2.872408208	3.672683231
1	-1.377465699	-0.846730690	4.280994249
1	-5.237495843	0.842213855	6.291801103
1	-5.560398250	2.565782245	5.912799669
1	-4.274132249	2.117623854	7.074182538
6	1.596236977	-4.035460853	2.337414274
6	0.187816981	-3.764600388	2.826618569
8	-0.588480233	-3.000643958	2.169665049
8	-0.219727380	-4.309100673	3.896223439
1	2.298212718	-4.071466915	3.190887608
1	1.905917806	-3.238966896	1.643753570
26	-2.137037340	-3.255114041	4.025912776
8	-1.717599557	-1.585130944	4.808356230
17	-4.742307607	1.248837823	3.465744489
8	-4.220964049	-2.367609276	0.289093569
1	-3.407564916	-2.206473163	-0.223302305

1	-3.921642394	-2.603062327	1.187214714
8	0.268265390	-2.607700926	6.272570178
1	0.431155154	-3.324852999	5.629726904
1	-0.550471130	-2.151772667	5.896598082
8	-0.205318476	-0.210150118	1.908897772
1	-0.397312883	-1.176405137	1.953282530
1	0.783596325	-0.165231680	1.824506045
8	1.664718980	-0.372931972	5.637715475
1	1.256496934	-1.219327545	5.960122445
1	2.515215755	-0.637472590	5.219411269
1	4.054854728	-8.660194895	7.743493014
1	-2.817824289	-4.639696037	10.234781480
1	-1.937401751	0.596064073	9.940891226
1	-9.525267795	1.380285970	-1.457651488
1	6.630444993	-6.432571295	4.762363445
1	-12.259608605	-3.140343021	-0.335312661
1	-9.505102141	-0.352558564	6.071846024
1	-6.894942081	-6.842527244	1.244648015
1	-6.376679891	4.724012922	-1.037638870
1	-5.987511839	6.091131217	6.488104262
1	-3.005749296	0.997624434	5.211656293
1	1.649812311	-4.998148492	1.829054641

^{5}PrOH$_{\text{model 2, AB}}$

6	3.210379000	-8.423283000	7.104947000
1	3.668568000	-8.538802000	6.099414000
6	2.063353000	-9.443284000	7.229031000
1	1.343398000	-9.311053000	6.399965000
1	1.500356000	-9.322476000	8.174569000
1	2.400830000	-10.496183000	7.189076000
6	2.740571000	-6.952228000	7.233209000
1	2.262752000	-6.780886000	8.219868000
1	3.615004000	-6.272909000	7.195637000
6	1.747840000	-6.506019000	6.150867000
1	1.490007000	-5.441198000	6.286150000
1	2.186170000	-6.608959000	5.137824000
1	0.807022000	-7.090189000	6.200713000
6	-2.450367000	-3.794131000	9.478153000
1	-3.136671000	-2.944576000	9.506420000
1	-1.478096000	-3.459522000	9.880787000
7	-1.930810000	-5.240022000	7.390804000
1	-1.648855000	-6.119594000	7.842982000
6	-2.391054000	-4.090799000	8.002629000
6	-2.020986000	-5.094035000	6.055342000
1	-1.723722000	-5.868797000	5.350081000
7	-2.493388000	-3.893151000	5.743938000
6	-2.742058000	-3.257160000	6.948901000
1	-3.171405000	-2.254272000	6.976822000
6	-1.012622000	0.575443000	9.351409000
1	-0.422029000	1.506006000	9.449889000
6	-0.085619000	-0.559041000	9.808471000
1	0.248238000	-0.429839000	10.854442000
1	0.821576000	-0.576456000	9.174063000
1	-0.565139000	-1.553881000	9.727218000
6	-1.425403000	0.406925000	7.887385000
1	-2.017844000	-0.510576000	7.722524000
1	-0.520079000	0.342581000	7.252591000
1	-2.031139000	1.239973000	7.494875000
6	-8.799033000	0.970656000	-0.791730000
1	-8.812542000	1.634761000	0.096659000
1	-9.154956000	-0.031932000	-0.479789000
8	-7.510764000	0.932506000	-1.387217000
1	-6.865283000	0.686367000	-0.678619000
6	5.644939000	-6.063810000	4.568004000
1	5.532082000	-5.926316000	3.477111000
1	4.881193000	-6.801351000	4.887436000
6	5.285409000	-4.731788000	5.284827000
8	6.041121000	-4.256311000	6.167948000
8	4.183989000	-4.217402000	4.933675000
6	-11.472538000	-3.908006000	-0.287585000
1	-11.467467000	-4.359837000	0.720925000
1	-11.806485000	-4.699177000	-0.987778000
6	-10.083914000	-3.404507000	-0.652839000
6	-9.144927000	-3.140010000	0.359939000
1	-9.403703000	-3.342127000	1.405114000
6	-7.892984000	-2.586947000	0.059832000
1	-7.179119000	-2.377393000	0.863211000
6	-7.567153000	-2.278440000	-1.266226000
1	-6.612500000	-1.795389000	-1.500105000
6	-9.717394000	-3.143771000	-1.987212000
1	-10.420116000	-3.349451000	-2.804155000

6	-8.479891000	-2.569509000	-2.286087000
1	-8.244399000	-2.316336000	-3.323322000
6	-9.573278000	0.520129000	6.813639000
1	-10.351500000	0.367939000	7.581257000
1	-8.599139000	0.549026000	7.336033000
6	-9.824763000	1.842375000	6.084970000
1	-9.373714000	2.672627000	6.646884000
1	-9.312502000	1.890253000	5.103757000
6	-11.281853000	2.280370000	5.828889000
8	-12.240234000	1.497559000	6.075751000
8	-11.413971000	3.416828000	5.301429000
6	-6.648660000	-5.855693000	1.577175000
1	-7.119565000	-5.167981000	0.844404000
1	-7.211623000	-5.674589000	2.502232000
7	-4.186909000	-5.563720000	0.914002000
1	-4.212841000	-6.088001000	0.030742000
6	-5.228868000	-5.456315000	1.821368000
6	-3.096164000	-4.969891000	1.444239000
1	-2.138526000	-4.887480000	0.934077000
7	-3.369459000	-4.488504000	2.648871000
6	-4.695692000	-4.781350000	2.898754000
1	-5.198184000	-4.451383000	3.809222000
6	-5.688347000	3.934770000	-1.165494000
1	-6.300372000	3.033593000	-1.367183000
1	-5.074263000	4.118632000	-2.069710000
6	-4.746855000	3.678972000	0.012505000
1	-4.161306000	4.585754000	0.257957000
1	-5.311669000	3.402743000	0.926979000
7	-3.811334000	2.592645000	-0.330305000
1	-4.029329000	2.022869000	-1.157829000
6	-2.768070000	2.220928000	0.434087000
7	-2.476440000	2.926020000	1.545029000
1	-2.761782000	3.906936000	1.626291000
1	-1.575335000	2.762006000	2.011539000
7	-2.046524000	1.133795000	0.150071000
1	-2.186086000	0.591764000	-0.711006000
1	-1.281858000	0.803913000	0.776806000
16	-4.845139000	5.419161000	7.249682000
6	-3.378182000	5.493687000	6.103161000
8	-3.112024000	6.400372000	5.360702000
6	-2.560466000	4.233083000	6.274042000
6	-2.809374000	3.247140000	5.095733000
6	-4.222762000	2.655811000	4.940440000
6	-4.636921000	1.586446000	5.977293000
6	-6.093144000	1.155469000	5.781484000
1	-1.496362000	4.531693000	6.258693000
1	-2.777336000	3.754506000	7.245139000
1	-2.541489000	3.781233000	4.167032000
1	-2.089641000	2.416699000	5.207900000
1	-4.988308000	3.457523000	4.926234000
1	-4.267568000	2.179778000	3.940015000
1	-3.821808000	0.062565000	4.995180000
1	-6.358045000	0.389821000	6.532874000
1	-6.246672000	0.718079000	4.775559000
1	-6.791533000	2.007347000	5.888336000
6	1.672904000	-4.025853000	2.470773000
6	0.270445000	-3.666970000	2.922514000
8	-0.525077000	-3.053081000	2.144494000
8	-0.141705000	-3.969137000	4.088183000
1	2.369426000	-4.061635000	3.329647000
1	2.013677000	-3.254775000	1.758550000
26	-2.173660000	-3.153418000	3.760837000
8	-3.766021000	0.440277000	5.896967000
17	-3.167467000	-1.149152000	3.065444000
8	-4.637738000	-2.190553000	0.330690000
1	-4.100889000	-2.053861000	-0.482495000
1	-4.048516000	-2.059001000	1.108109000
8	0.458641000	-2.894539000	6.601634000
1	0.373782000	-3.384751000	5.747569000
1	-0.457079000	-2.692789000	6.880837000
8	-0.199605000	-0.091874000	1.882624000
1	-0.500794000	-1.023252000	1.948053000
1	0.788552000	-0.130614000	1.790859000
8	1.631319000	-0.533878000	5.710301000
1	1.287806000	-1.364310000	6.119801000
1	2.487535000	-0.772768000	5.286250000
1	4.054559000	-8.679447000	7.747990000
1	-2.780893000	-4.597617000	10.139157000
1	-1.852249000	0.666952000	10.043085000
1	-9.542450000	1.402021000	-1.464796000

1	6.646237000	-6.449931000	4.768194000
1	-12.271049000	-3.165999000	-0.347333000
1	-9.566828000	-0.327935000	6.126245000
1	-6.902058000	-6.856803000	1.223153000
1	-6.368588000	4.779593000	-1.041752000
1	-5.871613000	6.050569000	6.572963000
1	-4.568407000	2.025382000	6.974492000
1	1.708728000	-4.999475000	1.978287000

^{1}Re$_{\text{model 3, A}}$

7	-1.361443000	-4.820390000	7.573486000
1	-0.955464000	-5.586715000	8.129001000
6	-2.293046000	-3.918433000	8.025943000
6	-1.180206000	-4.619956000	6.254980000
1	-0.509459000	-5.202898000	5.638125000
7	-1.939562000	-3.610689000	5.831505000
6	-2.646412000	-3.155752000	6.930114000
1	-3.347367000	-2.323698000	6.858997000
7	-3.635055000	-6.244289000	2.643431000
1	-3.604082000	-7.216018000	2.319158000
6	-4.790646000	-5.497738000	2.638637000
6	-2.597929000	-5.464873000	2.999835000
1	-1.571391000	-5.829800000	3.038236000
7	-3.018783000	-4.232585000	3.269170000
6	-4.386133000	-4.240413000	3.054865000
1	-4.983816000	-3.347748000	3.231791000
16	-3.168932000	4.344127000	7.088481000
6	-1.575600000	4.033957000	6.138474000
8	-1.517147000	3.867562000	4.951112000
6	-0.408120000	4.022342000	7.091576000
6	0.110215000	5.462325000	7.361310000
6	1.366922000	5.475213000	8.254694000
6	2.679739000	5.159910000	7.517190000
6	3.859589000	4.893255000	8.458513000
1	-0.704135000	3.557376000	8.050313000
1	0.372713000	3.396059000	6.625364000
1	0.325436000	5.970190000	6.400213000
1	-0.698475000	6.038340000	7.844566000
1	1.449234000	6.477049000	8.720387000
1	1.230114000	4.755555000	9.087475000
1	2.541125000	4.273809000	6.866325000
1	3.653394000	4.020351000	9.107686000
1	4.055157000	5.764874000	9.114483000
1	4.790442000	4.686437000	7.897760000
6	1.788427000	-3.974235000	2.127514000
6	0.708977000	-3.729711000	3.178399000
8	-0.452652000	-3.297582000	2.733601000
8	0.939746000	-3.921658000	4.373840000
1	2.683815000	-3.406084000	2.440178000
1	1.492008000	-3.622282000	1.125110000
26	-1.724347000	-2.646750000	4.093184000
8	-0.848448000	-1.469148000	4.646394000
17	-3.343707000	-1.301378000	3.280057000
1	-2.613554000	-3.786141000	9.061095000
1	-5.796463000	-5.921027000	2.608236000
1	-3.937416000	5.067828000	6.196314000
1	2.924428000	5.994638000	6.857552000
1	2.035631000	-5.036345000	2.076739000

^{3}Re$_{\text{model 3, A}}$

7	-1.435317000	-5.009772000	7.604773000
1	-1.062332000	-5.801603000	8.159442000
6	-2.090730000	-3.930224000	8.141834000
6	-1.404321000	-4.886190000	6.265160000
1	-0.948164000	-5.629980000	5.612815000
7	-2.008189000	-3.749065000	5.899453000
6	-2.440569000	-3.138635000	7.063507000
1	-2.978380000	-2.191456000	7.037322000
7	-3.665419000	-6.200150000	2.380586000
1	-3.643633000	-7.168602000	2.038111000
6	-4.829291000	-5.480797000	2.540947000
6	-2.616262000	-5.447244000	2.735619000
1	-1.582930000	-5.787540000	2.673473000
7	-3.037099000	-4.251044000	3.144831000
6	-4.418168000	-4.254471000	3.028928000
1	-5.006911000	-3.383657000	3.315818000
16	-3.152283000	4.412434000	7.118204000
6	-1.570003000	4.079852000	6.168341000
8	-1.509952000	3.905129000	4.981675000
6	-0.403849000	4.065740000	7.123494000

6	0.136634000	5.502526000	7.364005000
6	1.394427000	5.514783000	8.254677000
6	2.696975000	5.134996000	7.530291000
6	3.872418000	4.888298000	8.481816000
1	-0.708277000	3.623523000	8.090635000
1	0.367432000	3.418062000	6.670869000
1	0.357592000	5.987539000	6.392584000
1	-0.662197000	6.100160000	7.837501000
1	1.504616000	6.531568000	8.680253000
1	1.239016000	4.832211000	9.115073000
1	2.537661000	4.222133000	6.922906000
1	3.649778000	4.049131000	9.168833000
1	4.085406000	5.784343000	9.098180000
1	4.798380000	4.637666000	7.931400000
6	1.883818000	-3.898609000	2.205230000
6	0.688927000	-3.632530000	3.115069000
8	-0.406590000	-3.184161000	2.561311000
8	0.752048000	-3.833344000	4.336318000
1	2.780813000	-3.461115000	2.679015000
1	1.753572000	-3.431908000	1.214227000
26	-1.836147000	-2.828220000	3.996133000
8	-0.959751000	-1.525763000	4.718696000
17	-3.280088000	-1.207399000	3.282832000
1	-2.232473000	-3.761493000	9.211040000
1	-5.830869000	-5.914961000	2.551349000
1	-3.937141000	5.100429000	6.212148000
1	2.959111000	5.930423000	6.830044000
1	2.039030000	-4.970695000	2.069932000

$^{5}\mathbf{Re}_{\mathbf{model\ 3,\ A}}$

7	-1.382817000	-4.957395000	7.679810000
1	-1.015088000	-5.749016000	8.240492000
6	-2.062615000	-3.890012000	8.202677000
6	-1.333228000	-4.827674000	6.343194000
1	-0.853443000	-5.543120000	5.684631000
7	-1.947842000	-3.704476000	5.967895000
6	-2.412620000	-3.102998000	7.120464000
1	-2.974663000	-2.170671000	7.084029000
7	-3.600161000	-6.279741000	2.534105000
1	-3.588009000	-7.248246000	2.191749000
6	-4.745556000	-5.518763000	2.601477000
6	-2.540527000	-5.537851000	2.885132000
1	-1.518163000	-5.913637000	2.890590000
7	-2.936064000	-4.308690000	3.215516000
6	-4.312333000	-4.282401000	3.047093000
1	-4.883152000	-3.382097000	3.269258000
16	-3.183615000	4.398607000	7.104111000
6	-1.592853000	4.072181000	6.158884000
8	-1.528513000	3.898065000	4.973056000
6	-0.428350000	4.059584000	7.116387000
6	0.110718000	5.495072000	7.364508000
6	1.370369000	5.502109000	8.253286000
6	2.674181000	5.142014000	7.520442000
6	3.852373000	4.881458000	8.465430000
1	-0.732699000	3.612410000	8.081177000
1	0.344142000	3.417234000	6.658743000
1	0.329675000	5.986343000	6.395851000
1	-0.687804000	6.088828000	7.843066000
1	1.475573000	6.512773000	8.694314000
1	1.221231000	4.805998000	9.103895000
1	2.518175000	4.240779000	6.894579000
1	3.633581000	4.029932000	9.138496000
1	4.063621000	5.767204000	9.097128000
1	4.778286000	4.645194000	7.908214000
6	1.792117000	-4.074152000	2.167240000
6	0.476454000	-3.813033000	2.872044000
8	-0.374601000	-3.016483000	2.329019000
8	0.184523000	-4.298473000	3.992691000
1	2.578988000	-3.494911000	2.688863000
1	1.745585000	-3.689209000	1.134849000
26	-1.654320000	-2.848249000	4.040662000
8	-0.701818000	-1.702923000	4.693700000
17	-3.195858000	-1.302373000	3.320812000
1	-2.212062000	-3.733702000	9.272723000
1	-5.758269000	-5.926010000	2.583375000
1	-3.955126000	5.101776000	6.198243000
1	2.931737000	5.952833000	6.836336000
1	2.072520000	-5.129205000	2.165957000

$^{5}TS_{model\ 3,\ A}$

7	-1.019540000	-4.700589000	7.986948000
1	-0.546625000	-5.387363000	8.583006000
6	-2.227293000	-4.094382000	8.257676000
6	-0.674572000	-4.418400000	6.718995000
1	0.237922000	-4.767997000	6.247109000
7	-1.589017000	-3.632198000	6.157040000
6	-2.566555000	-3.419005000	7.104243000
1	-3.428812000	-2.783129000	6.895638000
7	-3.533753000	-6.278457000	2.699862000
1	-3.513603000	-7.254928000	2.388920000
6	-4.667879000	-5.495542000	2.660926000
6	-2.480439000	-5.514772000	3.062372000
1	-1.466393000	-5.907318000	3.135191000
7	-2.855152000	-4.263938000	3.298436000
6	-4.220234000	-4.237228000	3.059461000
1	-4.791032000	-3.322432000	3.232913000
16	-3.744049000	4.215369000	6.734106000
6	-3.235637000	2.946114000	5.455456000
8	-3.735258000	2.782571000	4.380197000
6	-2.087905000	2.159693000	6.033161000
6	-0.730600000	2.827624000	5.730874000
6	0.430023000	2.189890000	6.508070000
6	1.027622000	0.816536000	6.175640000
6	1.929322000	0.363539000	7.309187000
1	-2.212368000	2.116899000	7.131001000
1	-2.134772000	1.140622000	5.608095000
1	-0.544994000	2.820653000	4.644966000
1	-0.784621000	3.887918000	6.041824000
1	1.297760000	2.880410000	6.405794000
1	0.170162000	2.226205000	7.583116000
1	0.205667000	-0.206948000	5.837529000
1	1.383862000	0.332456000	8.267871000
1	2.754096000	1.102137000	7.412004000
1	2.399194000	-0.618150000	7.154494000
6	1.683668000	-4.114288000	2.028430000
6	0.545092000	-3.653016000	2.912825000
8	-0.187165000	-2.673961000	2.522384000
8	0.303137000	-4.130855000	4.052538000
1	2.530872000	-3.429777000	2.217030000
1	1.411840000	-3.949220000	0.972551000
26	-1.268969000	-2.530016000	4.388848000
8	-0.263327000	-1.260734000	5.199705000
17	-3.083912000	-1.194245000	3.982495000
1	-2.665818000	-3.959401000	9.248251000
1	-5.678283000	-5.908451000	2.642091000
1	-4.255502000	5.258613000	5.985552000
1	1.550099000	0.818128000	5.217110000
1	2.032446000	-5.141146000	2.153653000

$^{5}Int_{model\ 3,\ A}$

7	-0.995871000	-4.702802000	7.881199000
1	-0.504357000	-5.366916000	8.486904000
6	-2.133754000	-3.997844000	8.197075000
6	-0.737863000	-4.520192000	6.575346000
1	0.118645000	-4.946602000	6.069521000
7	-1.645114000	-3.717529000	6.026908000
6	-2.523900000	-3.372767000	7.032346000
1	-3.360162000	-2.695521000	6.848940000
7	-3.582699000	-6.316825000	2.657751000
1	-3.588710000	-7.303071000	2.373511000
6	-4.706304000	-5.520025000	2.614318000
6	-2.516544000	-5.571665000	2.990672000
1	-1.506821000	-5.974345000	3.068233000
7	-2.882495000	-4.311180000	3.205498000
6	-4.249623000	-4.262850000	2.979854000
1	-4.802246000	-3.334896000	3.132988000
16	-3.580004000	4.344792000	7.003072000
6	-2.213923000	3.559066000	5.917552000
8	-2.428629000	3.055644000	4.856750000
6	-0.887478000	3.659536000	6.629630000
6	-0.242117000	5.047275000	6.382398000
6	1.013706000	5.322449000	7.252385000
6	2.165388000	4.390315000	7.042126000
6	2.219556000	3.037930000	7.675616000
1	-1.052698000	3.521486000	7.715264000
1	-0.247230000	2.845340000	6.245856000
1	0.009528000	5.139919000	5.308068000
1	-0.980316000	5.836021000	6.609528000
1	1.320306000	6.365558000	7.050164000

1	0.693795000	5.289830000	8.316477000
1	-1.076282000	-0.715294000	4.972574000
1	1.469183000	2.960258000	8.486728000
1	3.217224000	2.828139000	8.114693000
1	2.010835000	2.222115000	6.946126000
6	1.859636000	-4.063026000	2.086481000
6	0.577666000	-3.759926000	2.823947000
8	-0.304965000	-3.000166000	2.287743000
8	0.341047000	-4.189978000	3.983888000
1	2.671361000	-3.473533000	2.555424000
1	1.775756000	-3.735511000	1.037160000
26	-1.457173000	-2.819680000	4.100616000
8	-0.396049000	-1.375576000	4.740560000
17	-3.202570000	-1.290985000	3.898079000
1	-2.520610000	-3.828836000	9.203824000
1	-5.723058000	-5.917208000	2.599478000
1	-4.217099000	5.166355000	6.092349000
1	2.944879000	4.652471000	6.324190000
1	2.128181000	-5.120318000	2.128684000

$^{5}\mathbf{TSCl}_{\mathbf{model\ 3,\ A}}$

7	-1.096324000	-4.864474000	7.879481000
1	-0.446577000	-5.478702000	8.387077000
6	-2.002077000	-3.996027000	8.444524000
6	-1.323072000	-4.902609000	6.557825000
1	-0.725250000	-5.489843000	5.867921000
7	-2.314876000	-4.081046000	6.215297000
6	-2.752561000	-3.509158000	7.396462000
1	-3.568507000	-2.782474000	7.413641000
7	-3.755822000	-6.060142000	2.432731000
1	-3.669233000	-7.015951000	2.066853000
6	-4.946780000	-5.385889000	2.555112000
6	-2.749046000	-5.252121000	2.800134000
1	-1.705481000	-5.555911000	2.744259000
7	-3.213293000	-4.063949000	3.191538000
6	-4.589268000	-4.139187000	3.052376000
1	-5.234482000	-3.314045000	3.355884000
16	-4.567361000	5.010065000	6.531176000
6	-4.191388000	3.569822000	5.391401000
8	-4.632033000	3.448721000	4.281334000
6	-3.186925000	2.663746000	6.026406000
6	-1.799744000	3.226834000	5.639035000
6	-0.653946000	2.267388000	5.908322000
6	-0.299888000	1.198039000	4.924512000
6	0.737627000	0.276314000	5.348416000
1	-3.291611000	2.632907000	7.125737000
1	-3.308089000	1.658975000	5.599116000
1	-1.786490000	3.490092000	4.565032000
1	-1.618423000	4.155140000	6.209414000
1	0.289335000	2.862888000	5.899076000
1	-0.710500000	1.853114000	6.925379000
1	0.364261000	-0.817677000	5.330620000
1	1.067912000	0.471185000	6.385171000
1	1.583119000	0.285060000	4.637652000
1	0.414883000	-3.024553000	5.334765000
6	1.817674000	-4.510745000	2.271741000
6	0.778711000	-4.234084000	3.349784000
8	-0.288888000	-3.627210000	2.954999000
8	1.027940000	-4.509807000	4.540125000
1	2.610788000	-3.746137000	2.379414000
1	1.378643000	-4.404954000	1.265664000
26	-1.720731000	-2.915211000	4.413385000
8	-0.244236000	-2.312919000	5.505360000
17	-2.223621000	-0.158902000	4.455181000
1	-2.032196000	-3.747776000	9.507175000
1	-5.917747000	-5.884801000	2.563677000
1	-4.358828000	6.061971000	5.659235000
1	-0.291946000	1.508717000	3.878004000
1	2.282260000	-5.494807000	2.358613000

$^{5}\mathbf{TSOH}_{\mathbf{model\ 3,\ A}}$

7	-0.995527000	-4.694174000	8.041567000
1	-0.529190000	-5.386467000	8.638381000
6	-2.202252000	-4.089220000	8.313702000
6	-0.668947000	-4.430947000	6.760269000
1	0.242135000	-4.780780000	6.283876000
7	-1.588423000	-3.659063000	6.192705000
6	-2.555541000	-3.437145000	7.149154000

1	-3.427942000	-2.817092000	6.932672000
7	-3.572234000	-6.283586000	2.668618000
1	-3.553733000	-7.255449000	2.341270000
6	-4.707580000	-5.503100000	2.648101000
6	-2.520225000	-5.526544000	3.038690000
1	-1.501458000	-5.910647000	3.089974000
7	-2.900948000	-4.282489000	3.300336000
6	-4.265624000	-4.251557000	3.064905000
1	-4.834196000	-3.339458000	3.256665000
16	-3.839230000	4.264882000	6.739023000
6	-3.284301000	3.000405000	5.485846000
8	-3.740849000	2.833888000	4.391085000
6	-2.145404000	2.217147000	6.089106000
6	-0.787793000	2.861425000	5.736138000
6	0.396467000	2.173410000	6.430281000
6	1.021681000	0.900537000	5.956872000
6	1.910317000	0.230436000	6.938247000
1	-2.259967000	2.203722000	7.188426000
1	-2.205743000	1.188332000	5.690030000
1	-0.646538000	2.873386000	4.642402000
1	-0.795057000	3.914461000	6.072138000
1	1.270832000	2.862908000	6.300164000
1	0.236250000	2.142986000	7.524513000
1	-0.752356000	-1.122935000	6.121968000
1	2.547824000	1.005788000	7.417693000
1	2.562225000	-0.533418000	6.497063000
1	1.337643000	-0.224233000	7.764737000
6	1.865529000	-3.998984000	2.133353000
6	0.592773000	-3.654219000	2.884497000
8	-0.219685000	-2.800402000	2.400425000
8	0.327981000	-4.144734000	4.017589000
1	2.709978000	-3.504510000	2.652693000
1	1.822020000	-3.586320000	1.111276000
26	-1.405731000	-2.716639000	4.257537000
8	-0.229148000	-1.177894000	5.309830000
17	-3.285170000	-1.275356000	4.169307000
1	-2.641822000	-3.949109000	9.303101000
1	-5.718338000	-5.915279000	2.632565000
1	-4.293042000	5.311845000	5.959154000
1	1.232817000	0.811230000	4.889534000
1	2.065015000	-5.071499000	2.092367000

$^{5}PrCl_{model\ 3,\ A}$

7	-0.736365000	-4.606005000	8.151128000
1	-0.108939000	-5.118656000	8.774621000
6	-1.934356000	-4.021051000	8.484809000
6	-0.645020000	-4.637465000	6.809802000
1	0.213139000	-5.036274000	6.278720000
7	-1.712593000	-4.080289000	6.248409000
6	-2.523365000	-3.675484000	7.289001000
1	-3.465351000	-3.150802000	7.112980000
7	-3.743968000	-6.185401000	2.500074000
1	-3.714335000	-7.142975000	2.125371000
6	-4.892176000	-5.440951000	2.593142000
6	-2.700449000	-5.452602000	2.908777000
1	-1.680308000	-5.834806000	2.913878000
7	-3.101495000	-4.244951000	3.303535000
6	-4.471203000	-4.224177000	3.116689000
1	-5.068249000	-3.356152000	3.399658000
16	-4.357409000	4.807032000	7.099768000
6	-3.325731000	3.492589000	6.190182000
8	-3.774345000	2.878317000	5.267159000
6	-1.945233000	3.383792000	6.775630000
6	-0.956056000	4.199950000	5.886668000
6	0.459292000	3.622431000	5.807382000
6	0.606846000	2.170905000	5.276992000
6	0.950107000	1.179745000	6.372065000
1	-1.927559000	3.764081000	7.806605000
1	-1.679677000	2.318749000	6.767076000
1	-1.349518000	4.255130000	4.856926000
1	-0.894084000	5.236388000	6.266512000
1	1.046711000	4.308011000	5.170731000
1	0.925438000	3.655660000	6.811717000
1	-1.090746000	-0.800464000	5.036795000
1	0.269997000	1.276952000	7.231052000
1	1.973278000	1.389030000	6.740755000
1	0.899142000	0.145880000	5.997183000
6	1.736460000	-4.482470000	2.064212000
6	0.471028000	-4.166657000	2.820553000
8	-0.412697000	-3.452373000	2.261999000
8	0.294138000	-4.535239000	4.024836000

1	2.469187000	-3.687635000	2.298454000
1	1.527847000	-4.409155000	0.986950000
26	-1.421298000	-3.229169000	4.285300000
8	-0.608133000	-1.623273000	4.868335000
17	-1.013321000	1.558671000	4.473951000
1	-2.272233000	-3.784486000	9.495571000
1	-5.886412000	-5.891239000	2.570796000
1	-4.568286000	5.674802000	6.044884000
1	1.389685000	2.143339000	4.516619000
1	2.199952000	-5.446715000	2.281400000

^{5}PrCl$_{\text{model 3, A}}$

7	-0.788537000	-4.583302000	8.032470000
1	-0.257538000	-5.206209000	8.647540000
6	-1.987601000	-3.974883000	8.322936000
6	-0.531199000	-4.405608000	6.723141000
1	0.348235000	-4.798923000	6.223715000
7	-1.493475000	-3.691251000	6.147901000
6	-2.409709000	-3.410515000	7.137565000
1	-3.293103000	-2.800574000	6.938763000
7	-3.660101000	-6.209396000	2.476577000
1	-3.639532000	-7.175930000	2.130980000
6	-4.812398000	-5.467916000	2.592162000
6	-2.604725000	-5.448478000	2.807520000
1	-1.578706000	-5.814262000	2.778800000
7	-3.004824000	-4.234393000	3.174749000
6	-4.381256000	-4.231231000	3.051258000
1	-4.964350000	-3.352619000	3.327098000
16	-4.363109000	4.567161000	6.708538000
6	-3.653223000	3.282803000	5.508731000
8	-4.119630000	3.033883000	4.437257000
6	-2.423441000	2.685850000	6.124844000
6	-1.255513000	3.698930000	5.995462000
6	-0.094241000	3.450437000	6.964372000
6	0.982219000	2.480288000	6.485115000
6	1.962283000	2.164678000	7.594084000
1	-2.617356000	2.507579000	7.198446000
1	-2.247937000	1.715607000	5.624802000
1	-0.902693000	3.741509000	4.945897000
1	-1.657043000	4.695190000	6.238430000
1	0.421680000	4.415065000	7.135536000
1	-0.497012000	3.146039000	7.946650000
1	-0.378759000	1.249081000	5.578769000
1	1.427306000	1.726417000	8.453936000
1	2.491107000	3.071936000	7.936741000
1	2.715355000	1.430496000	7.259571000
6	1.767125000	-4.277769000	2.009410000
6	0.548914000	-3.867488000	2.804371000
8	-0.291519000	-3.072336000	2.269628000
8	0.341435000	-4.243069000	3.993220000
1	2.574364000	-3.556063000	2.234373000
1	1.544379000	-4.171772000	0.934816000
26	-1.519504000	-3.000669000	4.141925000
8	0.477028000	1.175252000	6.036560000
17	-2.165623000	-0.771977000	4.229592000
1	-2.394821000	-3.800099000	9.320627000
1	-5.810876000	-5.909227000	2.599251000
1	-4.553261000	5.632682000	5.849015000
1	1.511042000	3.000893000	5.684440000
1	2.149958000	-5.281557000	2.203349000

^{5}Re$_{\text{model 3, AB}}$

6	-1.036059927	9.322207059	-0.961591030
1	-0.419644369	9.500909046	-0.062736508
1	-1.431990070	10.304886263	-1.276117445
6	-2.170373533	8.396685856	-0.629816337
6	-3.423311998	8.405394579	-1.195790455
1	-3.788602535	9.004612469	-2.027047875
7	-4.230953619	7.473854482	-0.581210862
1	-5.196857877	7.291197239	-0.839089381
6	-3.519332422	6.840296762	0.410233534
6	-2.206536998	7.399546942	0.410491587
6	-1.280575192	6.951160612	1.369813685
1	-0.261919380	7.349179259	1.396382802
6	-1.671239767	5.981315587	2.286731201
1	-0.965901237	5.653368581	3.051781499
6	-3.909895225	5.853150175	1.322639237
1	-4.916799021	5.432701276	1.302963908

6	-2.967614460	5.422729540	2.253525959	1	-6.674127554	-3.118117344	0.991110066
1	-3.228807042	4.637459088	2.968716954	6	-6.522025545	-3.736360659	-1.069030363
6	-2.317261606	-3.698385809	9.623663108	1	-5.455578238	-3.955491857	-0.994778175
1	-3.130653172	-2.971809010	9.606814914	6	-8.567489196	-3.600741674	-2.358702302
1	-1.433808291	-3.205469389	10.064946371	1	-9.102483129	-3.717733398	-3.306748273
7	-1.400397167	-5.002727341	7.617043025	6	-7.203220098	-3.899223895	-2.279987890
1	-1.014534288	-5.786469491	8.171306043	1	-6.665302724	-4.259295125	-3.162574445
6	-2.109542925	-3.959993314	8.163477262	6	-6.130226154	-6.106842913	2.566814882
6	-1.360558994	-4.866947121	6.285119447	1	-6.825148978	-5.257994683	2.459284869
1	-0.874045968	-5.568824207	5.620229649	1	-6.302629054	-6.520206397	3.569918057
7	-2.007770414	-3.759188275	5.925413905	7	-3.560675437	-6.281997195	2.522124588
6	-2.490095008	-3.182544346	7.084358331	1	-3.539992338	-7.249316257	2.185352578
1	-3.090414687	-2.273395813	7.058660341	6	-4.724838502	-5.547677279	2.603888617
6	-1.211145634	0.639001478	9.229351671	6	-2.511238666	-5.527656239	2.870412045
1	-0.533959424	1.513699810	9.219386438	1	-1.486007525	-5.889996935	2.877875672
6	-0.297962929	-0.590585020	9.315301634	7	-2.927379003	-4.309717869	3.201460676
1	0.387024100	-0.535248537	10.179559114	6	-4.303058252	-4.302542881	3.031593360
1	0.330471758	-0.670101992	8.409250249	1	-4.893697354	-3.416068694	3.246746770
1	-0.866476944	-1.530749885	9.382815899	6	-1.285893936	-1.897405720	-4.706433305
6	-2.039862098	0.645558201	7.937696090	1	-1.378247310	-2.990075108	-4.819389904
1	-1.358296968	0.649628606	7.067495616	1	-0.240267770	-1.714135298	-4.432298884
1	-2.703225156	1.523746277	7.836443574	6	-2.167422708	-1.455619824	-3.537005407
1	-2.677866956	-0.248032055	7.839026885	8	-1.645232959	-1.179948095	-2.448560494
6	-6.230308463	0.129354764	-1.213486126	7	-3.510764259	-1.486986223	-3.713247218
1	-6.146447580	0.716289625	-0.278201376	1	-4.113795078	-1.059008312	-2.983788329
1	-6.977993085	-0.669354884	-1.046145336	1	-3.885230358	-1.470180876	-4.667008864
8	-4.966723915	-0.377942709	-1.596671961	6	-2.546683413	2.497553719	-1.382902461
1	-4.610957976	-0.994957561	-0.903926596	1	-3.366863820	1.839914766	-1.729705280
6	4.183404106	-4.943218740	5.607070043	1	-1.699575822	2.341329196	-2.075971894
1	4.133482054	-4.949481500	4.505568697	6	-2.125661971	2.069257318	0.019097155
1	3.156700244	-4.957278921	6.013747506	1	-1.315506298	2.697465247	0.424771350
6	4.903698365	-3.684916428	6.149008449	1	-2.969692238	2.122971571	0.732706857
8	5.589441757	-3.005803590	5.338232914	7	-1.643442943	0.686752465	-0.070578814
8	4.753804273	-3.456314931	7.372234585	1	-2.071061198	0.096436420	-0.791881119
6	-10.769346865	-2.909964690	-1.304500724	6	-0.707526281	0.102986587	0.663662313
1	-11.251393349	-3.165171541	-0.346168388	7	-0.157746135	0.665029992	1.738914038
1	-11.208722438	-3.573621441	-2.067935142	1	-0.323528688	1.651062979	2.024035280
6	-9.276006086	-3.142614906	-1.234362862	1	0.695680283	0.268708275	2.136722855
6	-8.577370205	-2.978993602	-0.026948865	7	-0.257649412	-1.134377520	0.287002879
1	-9.111794897	-2.621728378	0.860513016	1	-0.730213041	-1.516248021	-0.536068280
6	-7.213141944	-3.269885241	0.052173686	1	-0.154059108	-1.815275818	1.054517894

16	-3.200244499	4.401706761	7.105940502
6	-1.616134957	4.099525326	6.156790318
8	-1.559661108	3.959839440	4.966160960
6	-0.440584019	4.064872482	7.095860512
6	0.107898056	5.493789884	7.348316642
6	1.364839530	5.495503969	8.236471803
6	2.665443641	5.127791097	7.507322652
6	3.841745070	4.875027917	8.452980028
1	-0.732677673	3.610000059	8.058394446
1	0.314560568	3.417110448	6.620065531
1	0.328400638	5.986582551	6.382751744
1	-0.686467513	6.089385572	7.827875380
1	1.474737648	6.505228258	8.674642493
1	1.211872146	4.802950547	9.086977400
1	2.507129705	4.223063009	6.890535606
1	3.622159303	4.031435370	9.132869508
1	4.053809651	5.765234460	9.075367934
1	4.765689801	4.631780794	7.898973290
6	1.788971534	-4.090436744	2.168556074
6	0.493987952	-3.839547106	2.907445748
8	-0.393245605	-3.075136388	2.370564175
8	0.248703321	-4.297684981	4.045398453
1	2.583396026	-3.507271911	2.668732774
1	1.719177045	-3.717767678	1.134829908
26	-1.672262322	-2.879700201	4.053823028
8	-0.736842695	-1.738236501	4.747486336
17	-3.169456252	-1.277272591	3.292406591
8	0.671695970	0.645678594	5.329880518
1	-0.092381086	0.079919783	5.090129584
1	1.215612821	0.719866117	4.507872438
8	-4.741927260	-0.380841886	6.007019906
1	-5.404280669	-1.110960469	5.923355787
1	-4.093283781	-0.536674399	5.295838656
8	1.697580924	-1.852271462	6.129099589
1	1.522365003	-0.883718841	6.131575736
1	0.876239233	-2.207049432	5.733630037
8	-3.665769663	-2.045018883	0.126841966
1	-3.077867143	-2.817900062	-0.040332773
1	-3.651478501	-1.891109860	1.093146108
1	-0.385716630	9.034795337	-1.787735942
1	-2.536200253	-4.530368250	10.292945119
1	-1.830538401	0.717184146	10.122832160
1	-6.623137447	0.829023625	-1.951196228
1	4.754112900	-5.816064232	5.924086665
1	-11.127591713	-1.926447661	-1.608510926
1	-6.516689538	-6.858103856	1.878105679
1	-1.426791348	-1.482492492	-5.704462491
1	-2.875501434	3.530507650	-1.496710178
1	-3.970572303	5.108672378	6.205306108
1	2.923647430	5.930661169	6.816817199
1	2.075823508	-5.141994537	2.171634398

^{5}TS$_{\text{model 3, AB}}$

6	-1.051393000	9.355396000	-0.977771000
1	-0.441757000	9.562503000	-0.078683000
1	-1.468765000	10.324511000	-1.311563000
6	-2.164671000	8.407281000	-0.630891000
6	-3.428626000	8.380201000	-1.175379000
1	-3.825919000	8.967365000	-2.003324000
7	-4.206505000	7.436626000	-0.533108000
1	-5.176025000	7.237661000	-0.768398000
6	-3.466222000	6.839175000	0.463555000
6	-2.162516000	7.427304000	0.428014000
6	-1.210897000	7.038045000	1.388404000
1	-0.197129000	7.451547000	1.373054000
6	-1.575828000	6.117161000	2.370764000
1	-0.858382000	5.875692000	3.164477000
6	-3.820428000	5.880256000	1.421792000
1	-4.817274000	5.433220000	1.450653000
6	-2.862253000	5.528053000	2.374559000
1	-3.137187000	4.791534000	3.134897000
6	-2.600131000	-3.821121000	9.578631000
1	-3.632708000	-3.496155000	9.378334000
1	-2.043681000	-2.985699000	10.044729000
7	-0.794750000	-4.616822000	7.915173000
1	-0.206865000	-5.191687000	8.526496000
6	-2.033990000	-4.083905000	8.220877000
6	-0.555886000	-4.431268000	6.606588000
1	0.353327000	-4.754837000	6.114726000

7	-1.576624000	-3.797968000	6.035197000	7	-3.531384000	-6.388463000	2.766410000
6	-2.506425000	-3.573886000	7.029198000	1	-3.527763000	-7.384944000	2.529112000
1	-3.416348000	-3.000582000	6.836848000	6	-4.668783000	-5.607313000	2.655051000
6	-1.170031000	0.403991000	9.329127000	6	-2.491920000	-5.629657000	3.158636000
1	-0.681403000	1.342729000	9.004515000	1	-1.488046000	-6.021243000	3.319389000
6	-0.051734000	-0.548203000	9.780903000	7	-2.880421000	-4.370208000	3.321517000
1	0.579432000	-0.116380000	10.578857000	6	-4.231046000	-4.340625000	3.004078000
1	0.609192000	-0.787398000	8.924894000	1	-4.803789000	-3.417833000	3.072823000
1	-0.450216000	-1.516725000	10.145270000	6	-1.324267000	-1.868795000	-4.734035000
6	-1.939891000	-0.223481000	8.167363000	1	-1.456644000	-2.960171000	-4.829173000
1	-1.279448000	-0.368882000	7.295662000	1	-0.273206000	-1.721663000	-4.454169000
1	-2.802785000	0.379201000	7.848658000	6	-2.197750000	-1.377690000	-3.576544000
1	-2.320600000	-1.230234000	8.414812000	8	-1.682955000	-1.171312000	-2.470518000
6	-6.257889000	0.128172000	-1.213286000	7	-3.536985000	-1.296171000	-3.777230000
1	-6.175820000	0.718428000	-0.278032000	1	-4.130792000	-0.890212000	-3.025926000
1	-7.011364000	-0.668368000	-1.049762000	1	-3.911294000	-1.260340000	-4.733948000
8	-4.995709000	-0.386165000	-1.592129000	6	-2.672710000	2.490389000	-1.429378000
1	-4.665574000	-1.051476000	-0.926004000	1	-3.522415000	1.871021000	-1.778175000
6	4.211527000	-4.922422000	5.620063000	1	-1.829245000	2.289975000	-2.117686000
1	4.136839000	-4.928111000	4.518544000	6	-2.273307000	2.061717000	-0.027615000
1	3.195986000	-4.915822000	6.057163000	1	-1.429093000	2.665357000	0.340414000
6	4.957400000	-3.667055000	6.136617000	1	-3.104763000	2.170775000	0.697027000
8	5.643407000	-3.005764000	5.304265000	7	-1.845618000	0.665629000	-0.082852000
8	4.823336000	-3.412155000	7.357648000	1	-2.372910000	0.040494000	-0.699305000
6	-10.800605000	-2.911412000	-1.306298000	6	-0.783117000	0.135789000	0.512002000
1	-11.278897000	-3.161487000	-0.342800000	7	-0.047684000	0.798607000	1.399698000
1	-11.254203000	-3.571618000	-2.066573000	1	-0.246529000	1.768269000	1.702819000
6	-9.308526000	-3.166940000	-1.249837000	1	0.833113000	0.388404000	1.754286000
6	-8.595766000	-3.023667000	-0.046226000	7	-0.406811000	-1.137266000	0.172117000
1	-9.114664000	-2.654428000	0.847529000	1	-0.994663000	-1.560057000	-0.552151000
6	-7.237337000	-3.352125000	0.022463000	1	-0.221360000	-1.763963000	0.971914000
1	-6.686098000	-3.219028000	0.958669000	16	-4.361014000	4.752638000	6.845062000
6	-6.565515000	-3.831720000	-1.106893000	6	-4.367237000	3.434293000	5.580387000
1	-5.503459000	-4.080252000	-1.039573000	8	-4.485018000	3.568397000	4.386854000
6	-8.618245000	-3.636297000	-2.383669000	6	-4.155686000	2.090759000	6.238512000
1	-9.164868000	-3.736751000	-3.328839000	6	-3.050448000	1.340436000	5.474250000
6	-7.258920000	-3.967924000	-2.316788000	6	-1.638793000	1.908552000	5.633487000
1	-6.734771000	-4.332437000	-3.207717000	6	-0.594131000	1.112640000	4.861759000
6	-6.092491000	-6.126686000	2.568611000	6	0.848028000	1.460864000	5.172862000
1	-6.757305000	-5.255148000	2.437386000	1	-3.927894000	2.195719000	7.314732000
1	-6.311755000	-6.532780000	3.565948000	1	-5.098302000	1.515702000	6.149477000

1	-3.061523000	0.300105000	5.814481000
1	-3.322060000	1.312559000	4.405963000
1	-1.592748000	2.951493000	5.245808000
1	-1.367170000	1.974844000	6.703359000
1	-0.721782000	-0.076787000	5.039093000
1	1.021069000	1.536617000	6.262961000
1	1.111937000	2.441375000	4.723811000
1	1.531250000	0.693807000	4.770223000
6	1.817182000	-4.083249000	2.049227000
6	0.555058000	-3.759576000	2.813296000
8	-0.324632000	-2.987473000	2.299303000
8	0.339633000	-4.173140000	3.984735000
1	2.629919000	-3.453054000	2.461033000
1	1.696476000	-3.807654000	0.988401000
26	-1.488978000	-2.834999000	4.142592000
8	-0.637225000	-1.507690000	4.951923000
17	-3.106518000	-1.303756000	3.175628000
8	4.044034000	1.185929000	5.128307000
1	3.504615000	1.029106000	4.333855000
1	4.827187000	0.577364000	5.032555000
8	-4.603889000	-1.337438000	6.007111000
1	-5.487238000	-1.780989000	5.914771000
1	-4.232491000	-1.245717000	5.102015000
8	1.752316000	-1.766117000	6.345475000
1	1.967594000	-0.910768000	6.791315000
1	0.856196000	-1.667005000	5.928154000
8	-3.738278000	-2.076355000	0.095907000
1	-3.156133000	-2.854956000	-0.075872000
1	-3.655456000	-1.882546000	1.056415000
1	-0.391087000	9.065905000	-1.797499000
1	-2.633357000	-4.610487000	10.331999000
1	-1.850795000	0.630333000	10.151984000
1	-6.642756000	0.831909000	-1.953824000
1	4.769452000	-5.807675000	5.931206000
1	-11.151471000	-1.923542000	-1.610879000
1	-6.492610000	-6.872455000	1.879047000
1	-1.443147000	-1.462725000	-5.740385000
1	-2.946465000	3.542072000	-1.533237000
1	-4.272602000	5.907631000	6.091105000
1	-0.797808000	1.141833000	3.789646000
1	2.115192000	-5.130353000	2.130035000

^{5}Int$_{model\ 3,\ AB}$

6	-1.054832000	9.359781000	-0.982116000
1	-0.447021000	9.569812000	-0.082502000
1	-1.472397000	10.327504000	-1.319570000
6	-2.167345000	8.411318000	-0.634385000
6	-3.433224000	8.382041000	-1.173985000
1	-3.834633000	8.968239000	-2.000499000
7	-4.206639000	7.435857000	-0.529745000
1	-5.176093000	7.234221000	-0.762966000
6	-3.460772000	6.837295000	0.462191000
6	-2.158757000	7.429354000	0.421784000
6	-1.200074000	7.040423000	1.374470000
1	-0.188310000	7.458782000	1.355642000
6	-1.555186000	6.110659000	2.351561000
1	-0.832515000	5.865340000	3.139025000
6	-3.806485000	5.872570000	1.417870000
1	-4.802078000	5.423665000	1.455414000
6	-2.838552000	5.515798000	2.358908000
1	-3.102988000	4.770672000	3.114669000
6	-2.619820000	-3.811463000	9.568809000
1	-3.656876000	-3.497861000	9.373830000
1	-2.068768000	-2.970306000	10.029235000
7	-0.841804000	-4.645751000	7.898900000
1	-0.269947000	-5.235065000	8.512027000
6	-2.059982000	-4.071439000	8.208841000
6	-0.605899000	-4.475731000	6.587968000
1	0.288427000	-4.834485000	6.093268000
7	-1.606605000	-3.811192000	6.018194000
6	-2.522126000	-3.550551000	7.017026000
1	-3.416369000	-2.950902000	6.832578000
6	-1.174456000	0.433628000	9.320695000
1	-0.632252000	1.364547000	9.065843000
6	-0.113601000	-0.603068000	9.723477000
1	0.550651000	-0.234331000	10.525890000
1	0.523919000	-0.848384000	8.852909000
1	-0.562091000	-1.558606000	10.061678000
6	-1.966993000	-0.059973000	8.109385000

1	-1.311002000	-0.147665000	7.223938000
1	-2.803770000	0.605700000	7.847445000
1	-2.390383000	-1.068724000	8.271061000
6	-6.259263000	0.127580000	-1.209540000
1	-6.180448000	0.718841000	-0.274705000
1	-7.012427000	-0.669723000	-1.048208000
8	-4.994975000	-0.386011000	-1.583180000
1	-4.660094000	-1.035218000	-0.903969000
6	4.199477000	-4.927832000	5.639418000
1	4.126220000	-4.926838000	4.538003000
1	3.182560000	-4.936015000	6.074006000
6	4.925121000	-3.665510000	6.168006000
8	5.573126000	-2.965254000	5.336989000
8	4.809536000	-3.438789000	7.395792000
6	-10.801085000	-2.911662000	-1.306284000
1	-11.279491000	-3.161832000	-0.342881000
1	-11.254102000	-3.572038000	-2.066712000
6	-9.308798000	-3.165486000	-1.249553000
6	-8.595126000	-3.015642000	-0.047184000
1	-9.114076000	-2.644859000	0.845958000
6	-7.235459000	-3.338962000	0.020705000
1	-6.683037000	-3.199255000	0.955051000
6	-6.563642000	-3.821200000	-1.107631000
1	-5.500718000	-4.066092000	-1.040405000
6	-8.618657000	-3.637522000	-2.382303000
1	-9.166196000	-3.742915000	-3.326387000
6	-7.258278000	-3.965056000	-2.315875000
1	-6.734369000	-4.331887000	-3.206014000
6	-6.108291000	-6.114309000	2.550931000
1	-6.774882000	-5.246002000	2.408572000
1	-6.326530000	-6.509530000	3.552861000
7	-3.554378000	-6.386368000	2.743545000
1	-3.564603000	-7.386232000	2.517277000
6	-4.684755000	-5.596404000	2.628677000
6	-2.505539000	-5.636054000	3.118766000
1	-1.504640000	-6.035315000	3.279462000
7	-2.882390000	-4.371024000	3.269270000
6	-4.234299000	-4.329320000	2.960068000
1	-4.797569000	-3.399742000	3.022373000
6	-1.323027000	-1.865807000	-4.726432000
1	-1.457473000	-2.957474000	-4.817844000
1	-0.271486000	-1.718561000	-4.447984000
6	-2.195174000	-1.368900000	-3.569709000
8	-1.678220000	-1.156031000	-2.464473000
7	-3.533660000	-1.287425000	-3.768377000
1	-4.128913000	-0.885064000	-3.015483000
1	-3.910146000	-1.258099000	-4.724449000
6	-2.669229000	2.489205000	-1.428328000
1	-3.518376000	1.867817000	-1.775026000
1	-1.826511000	2.292649000	-2.118754000
6	-2.263683000	2.060427000	-0.028209000
1	-1.424793000	2.671443000	0.339797000
1	-3.096259000	2.157868000	0.697035000
7	-1.819173000	0.669831000	-0.095453000
1	-2.289918000	0.062244000	-0.773310000
6	-0.797349000	0.128599000	0.557707000
7	-0.140946000	0.759187000	1.529949000
1	-0.315420000	1.749181000	1.784445000
1	0.755452000	0.366896000	1.871959000
7	-0.388269000	-1.127217000	0.197078000
1	-0.948013000	-1.549427000	-0.549253000
1	-0.186249000	-1.769343000	0.978835000
16	-4.478536000	4.975265000	6.742440000
6	-4.556607000	3.626550000	5.503417000
8	-4.707566000	3.746560000	4.311928000
6	-4.334459000	2.288616000	6.166253000
6	-3.109064000	1.648590000	5.483636000
6	-1.779093000	2.373762000	5.736374000
6	-0.630811000	1.695073000	5.064099000
6	0.780195000	1.804191000	5.531023000
1	-4.197261000	2.381903000	7.258825000
1	-5.215205000	1.645618000	5.980577000
1	-3.038756000	0.609013000	5.821986000
1	-3.303613000	1.602505000	4.397551000
1	-1.851753000	3.422534000	5.355948000
1	-1.590480000	2.474387000	6.823516000
1	-0.685814000	-0.596181000	4.705400000
1	0.870294000	2.355329000	6.485372000
1	1.417537000	2.312563000	4.777946000
1	1.225493000	0.799001000	5.681003000
6	1.863360000	-4.102712000	2.074993000
6	0.567020000	-3.813654000	2.789420000

8	-0.310136000	-3.059286000	2.249386000
8	0.318003000	-4.242367000	3.951178000
1	2.653456000	-3.477615000	2.536308000
1	1.784165000	-3.802927000	1.016780000
26	-1.462486000	-2.895395000	4.100015000
8	-0.422134000	-1.526883000	4.858830000
17	-3.024038000	-1.270315000	3.262553000
8	3.900819000	0.970600000	5.216733000
1	3.350312000	0.228848000	4.885720000
1	4.818642000	0.609984000	5.074221000
8	-4.659459000	-1.067826000	6.008126000
1	-5.482723000	-1.614172000	5.918707000
1	-4.204754000	-1.086158000	5.138884000
8	1.724978000	-1.756215000	6.438713000
1	1.900714000	-0.929474000	6.947748000
1	0.859973000	-1.643465000	5.940219000
8	-3.759498000	-2.071045000	0.143519000
1	-3.159510000	-2.835066000	-0.036148000
1	-3.646214000	-1.851593000	1.093262000
1	-0.392803000	9.067919000	-1.799611000
1	-2.640033000	-4.600728000	10.322744000
1	-1.848157000	0.642819000	10.153840000
1	-6.642743000	0.830647000	-1.951431000
1	4.765674000	-5.810557000	5.942704000
1	-11.151922000	-1.923794000	-1.610905000
1	-6.503647000	-6.869625000	1.869054000
1	-1.442356000	-1.463150000	-5.734099000
1	-2.945735000	3.540199000	-1.531875000
1	-4.186385000	6.080947000	5.966302000
1	-0.788565000	1.347199000	4.041425000
1	2.158459000	-5.151111000	2.149441000

$^{5}PrCl_{model\ 3,\ AB}$

6	-1.060062354	9.370126191	-0.981487714
1	-0.454311649	9.579454370	-0.082565436
1	-1.479099651	10.335655538	-1.317379744
6	-2.168079809	8.419641517	-0.638243643
6	-3.435117853	8.395415275	-1.167836926
1	-3.841729607	8.988113416	-1.983919665
7	-4.198519192	7.433015249	-0.538647379
1	-5.163747977	7.225818750	-0.777595344
6	-3.439030018	6.807503626	0.421434073
6	-2.144513942	7.412042685	0.389240083
6	-1.172138672	6.998836382	1.312976556
1	-0.172646051	7.442181652	1.310703835
6	-1.493599764	6.004674481	2.233772947
1	-0.753836476	5.731996176	2.994693307
6	-3.754737355	5.790293691	1.328968075
1	-4.746085207	5.330654397	1.359429010
6	-2.761062610	5.381079496	2.218245627
1	-2.995488486	4.583131455	2.924910486
6	-2.396473329	-3.817524574	9.746124979
1	-3.367000263	-3.368044917	9.498314517
1	-1.771827127	-3.075954453	10.276575830
7	-0.506830332	-4.477570648	8.131693115
1	0.201275265	-4.810951209	8.784019668
6	-1.815627234	-4.143513127	8.419469911
6	-0.347560098	-4.453251906	6.797120232
1	0.588574554	-4.677232611	6.294823298
7	-1.477452814	-4.103716918	6.201410157
6	-2.397882115	-3.915808220	7.198561692
1	-3.409532440	-3.584926561	6.984807554
6	-1.121320092	0.409625472	9.348500823
1	-0.773608560	1.361117128	8.910273231
6	0.104352505	-0.287531115	9.950717138
1	0.562398796	0.261658893	10.791446000
1	0.876050941	-0.427218843	9.174059051
1	-0.158613525	-1.303082016	10.300467590
6	-1.676632157	-0.515791441	8.264228885
1	-0.933549203	-0.647589284	7.460162587
1	-2.607643716	-0.171460022	7.800883948
1	-1.873039277	-1.524426053	8.655348542
6	-6.271757917	0.093397255	-1.197592007
1	-6.210298583	0.671718237	-0.252161553
1	-7.031727124	-0.702438808	-1.068794070
8	-5.005401565	-0.407835853	-1.552760457
1	-4.636830479	-0.994856737	-0.834928778
6	4.193018966	-4.916867274	5.632647942
1	4.119439097	-4.910996439	4.533303231

1	3.176350972	-4.931541110	6.062757548	1	-1.195789942	2.652492383	0.163230940
6	4.909150436	-3.658029916	6.168334653	1	-2.813115114	2.185847538	0.735628768
8	5.561683114	-2.954960880	5.346800944	7	-1.667830950	0.655917719	-0.166865552
8	4.772949065	-3.424897011	7.392106117	1	-2.145829408	0.059955343	-0.846522570
6	-10.746644076	-2.903959047	-1.294287762	6	-0.657279128	0.090215339	0.487875700
1	-11.245898008	-3.168649294	-0.347403261	7	-0.001255939	0.709422333	1.462160020
1	-11.154841743	-3.577083615	-2.067226774	1	-0.175730297	1.694241138	1.725277913
6	-9.249753343	-3.103667815	-1.184421749	1	0.884546993	0.305405563	1.813219208
6	-8.587868775	-2.931975219	0.044036501	7	-0.274267329	-1.168622162	0.130894374
1	-9.158180485	-2.602149057	0.919862113	1	-0.835276712	-1.579136128	-0.617476559
6	-7.218858407	-3.191234944	0.160263039	1	-0.071289981	-1.828084051	0.909298237
1	-6.713182211	-3.047493898	1.120735664	16	-4.554213988	5.425552647	6.505503456
6	-6.485163174	-3.634403422	-0.945270759	6	-4.645070716	3.888147358	5.523886793
1	-5.415311168	-3.831655644	-0.849251071	8	-4.545675787	3.756793039	4.331400261
6	-8.499207386	-3.538632664	-2.290196333	6	-4.874807483	2.757415081	6.485046586
1	-9.001560233	-3.664763284	-3.255135859	6	-3.793249785	1.675701450	6.380678968
6	-7.131399870	-3.806196435	-2.174401519	6	-2.328323252	2.130137509	6.442644211
1	-6.562832731	-4.149063662	-3.043962424	6	-1.583249592	2.536226294	5.164449634
6	-6.134296305	-6.119193883	2.593883071	6	-0.078801885	2.368018113	5.350290265
1	-6.845856282	-5.280069576	2.518358126	1	-4.937620119	3.134179016	7.518350843
1	-6.272980412	-6.551656248	3.594584951	1	-5.846181857	2.308689094	6.218862174
7	-3.565091215	-6.225038736	2.346262901	1	-3.986046832	0.995300473	7.224417340
1	-3.548133063	-7.179124694	1.976999222	1	-3.952165867	1.073352445	5.475191198
6	-4.735606704	-5.539862035	2.593565009	1	-2.207518447	2.955412226	7.170296388
6	-2.513457432	-5.481726209	2.725651497	1	-1.755733096	1.293310281	6.849855407
1	-1.482935646	-5.825633762	2.662403063	1	-0.475899027	-0.829242953	4.088983174
7	-2.926736359	-4.320724184	3.215691845	1	0.168318567	1.314900761	5.569765952
6	-4.305334159	-4.340316093	3.132401267	1	0.249711970	2.986208617	6.207262895
1	-4.908777842	-3.529620993	3.529679007	1	0.492158242	2.693068429	4.467788320
6	-1.330064499	-1.866551707	-4.725416332	6	1.977043028	-4.060436739	2.128801712
1	-1.464654795	-2.955979525	-4.818849545	6	0.627236227	-3.758563301	2.752921588
1	-0.281169436	-1.722077248	-4.443103196	8	-0.187980470	-3.003642345	2.153406343
6	-2.203590492	-1.372021714	-3.574245558	8	0.335770675	-4.237215416	3.889523790
8	-1.691754534	-1.153573567	-2.470663783	1	2.757281707	-3.514408152	2.690891542
7	-3.541980060	-1.297092973	-3.770430895	1	1.998880606	-3.696627306	1.089851625
1	-4.130084217	-0.900591768	-3.014704833	26	-1.482914257	-3.176296599	4.333743648
1	-3.920216992	-1.262026656	-4.724149027	8	-0.692654595	-1.421069698	4.828422166
6	-2.642964832	2.454546356	-1.437194332	17	-2.126531891	1.397619214	3.758666020
1	-3.531037108	1.839332041	-1.677400221	8	3.819075699	0.988798948	5.194929878
1	-1.883167127	2.223625391	-2.204996521	1	3.303694206	0.210656647	4.888105125
6	-2.082589515	2.051048768	-0.085130781	1	4.748452709	0.653630271	5.081404330

8	-3.290733089	-1.662861906	5.184011650
1	-3.774302382	-1.372121035	4.378137185
1	-2.392267671	-1.217324699	5.161982636
8	1.488146682	-1.550008339	6.321561783
1	1.614045686	-0.711428894	6.814456925
1	0.617011740	-1.488092256	5.798501262
8	-3.773053868	-1.889808063	0.364804768
1	-3.176487997	-2.667833501	0.258820968
1	-3.979193997	-1.785723534	1.321311187
1	-0.398515803	9.079408377	-1.797518741
1	-2.557934817	-4.640029482	10.442891218
1	-1.850542296	0.627578737	10.128756341
1	-6.640641795	0.807749292	-1.933590088
1	4.756660915	-5.800645372	5.931497983
1	-11.118751722	-1.927330027	-1.603801852
1	-6.524368757	-6.863613687	1.899800967
1	-1.444629653	-1.461354840	-5.730780767
1	-2.909531161	3.506468731	-1.539512600
1	-3.959160066	6.330861555	5.650708613
1	-1.792977278	3.568085644	4.882762033
1	2.212779575	-5.124184638	2.159395899

^{5}PrOH$_{\text{model 3, AB}}$

6	-1.079019000	9.380010000	-1.001879000
1	-0.480422000	9.624218000	-0.104988000
1	-1.517357000	10.329483000	-1.363801000
6	-2.167010000	8.412253000	-0.632368000
6	-3.447001000	8.348509000	-1.133577000
1	-3.889504000	8.921942000	-1.947945000
7	-4.177373000	7.386425000	-0.460034000
1	-5.146181000	7.158591000	-0.666701000
6	-3.386803000	6.813813000	0.514911000
6	-2.103208000	7.438339000	0.427003000
6	-1.098840000	7.086940000	1.344815000
1	-0.100633000	7.531228000	1.275905000
6	-1.389523000	6.160881000	2.344888000
1	-0.630562000	5.942473000	3.107102000
6	-3.669902000	5.848643000	1.492185000
1	-4.648794000	5.368385000	1.575940000
6	-2.655574000	5.530193000	2.398918000
1	-2.866535000	4.779211000	3.165519000
6	-2.614324000	-3.796472000	9.567562000
1	-3.651992000	-3.492709000	9.357589000
1	-2.085704000	-2.955024000	10.052069000
7	-0.775994000	-4.557992000	7.929445000
1	-0.206206000	-5.144896000	8.546128000
6	-2.013786000	-4.015586000	8.217425000
6	-0.496400000	-4.336860000	6.634750000
1	0.411447000	-4.678913000	6.150477000
7	-1.483974000	-3.660790000	6.052008000
6	-2.440572000	-3.460159000	7.027772000
1	-3.343316000	-2.878218000	6.832287000
6	-1.233745000	0.417923000	9.310204000
1	-0.693478000	1.341011000	9.025633000
6	-0.168425000	-0.621659000	9.689746000
1	0.483033000	-0.276327000	10.511848000
1	0.478673000	-0.834790000	8.817891000
1	-0.610342000	-1.589095000	9.998041000
6	-2.073006000	-0.096780000	8.139740000
1	-1.447881000	-0.182530000	7.237908000
1	-2.926628000	0.557639000	7.896635000
1	-2.480201000	-1.104678000	8.337394000
6	-6.256281000	0.133689000	-1.196617000
1	-6.187552000	0.731811000	-0.265465000
1	-7.006130000	-0.666746000	-1.035564000
8	-4.985198000	-0.375074000	-1.555271000
1	-4.629267000	-0.971476000	-0.841696000
6	4.199978000	-4.928426000	5.633953000
1	4.133322000	-4.926540000	4.532381000
1	3.179239000	-4.943439000	6.058689000
6	4.916894000	-3.665015000	6.168970000
8	5.605216000	-2.986798000	5.353522000
8	4.745887000	-3.410662000	7.386981000
6	-10.788937000	-2.912687000	-1.306074000
1	-11.266202000	-3.164891000	-0.342616000
1	-11.237867000	-3.576309000	-2.066394000
6	-9.294413000	-3.155806000	-1.247968000
6	-8.584112000	-3.005165000	-0.043528000
1	-9.108357000	-2.644557000	0.850726000

6	-7.220461000	-3.312349000	0.024773000
1	-6.670292000	-3.171541000	0.960238000
6	-6.541077000	-3.778798000	-1.105795000
1	-5.474720000	-4.010393000	-1.039277000
6	-8.597183000	-3.614766000	-2.381835000
1	-9.141403000	-3.721133000	-3.327777000
6	-7.232957000	-3.925976000	-2.315412000
1	-6.704078000	-4.281916000	-3.207053000
6	-6.173689000	-6.105645000	2.509054000
1	-6.873384000	-5.261370000	2.381567000
1	-6.343514000	-6.505007000	3.519018000
7	-3.611697000	-6.307705000	2.420750000
1	-3.626139000	-7.293495000	2.135938000
6	-4.765347000	-5.554763000	2.520586000
6	-2.548637000	-5.569067000	2.767683000
1	-1.526671000	-5.948140000	2.778960000
7	-2.943629000	-4.348758000	3.106004000
6	-4.316199000	-4.315913000	2.945773000
1	-4.892419000	-3.418331000	3.170199000
6	-1.312564000	-1.860570000	-4.716134000
1	-1.449975000	-2.952676000	-4.801950000
1	-0.258893000	-1.713257000	-4.445028000
6	-2.176344000	-1.356877000	-3.554653000
8	-1.652597000	-1.133962000	-2.454381000
7	-3.517192000	-1.280960000	-3.744748000
1	-4.108459000	-0.879627000	-2.988758000
1	-3.899739000	-1.257577000	-4.698019000
6	-2.637709000	2.504947000	-1.413661000
1	-3.489497000	1.872592000	-1.733631000
1	-1.808429000	2.306581000	-2.119548000
6	-2.195850000	2.096434000	-0.017355000
1	-1.360042000	2.724341000	0.330209000
1	-3.015888000	2.188141000	0.723249000
7	-1.730048000	0.712804000	-0.083956000
1	-2.170863000	0.105395000	-0.782230000
6	-0.732419000	0.172900000	0.609291000
7	-0.120744000	0.801441000	1.611782000
1	-0.278621000	1.804042000	1.823899000
1	0.768920000	0.412031000	1.976049000
7	-0.307802000	-1.079299000	0.256007000
1	-0.853861000	-1.507847000	-0.496303000
1	-0.101620000	-1.727324000	1.035011000
16	-4.476475000	5.010299000	6.661096000
6	-4.466393000	3.646787000	5.430262000
8	-4.600573000	3.753164000	4.235771000
6	-4.206791000	2.324981000	6.107455000
6	-2.925360000	1.724573000	5.486569000
6	-1.622558000	2.396662000	5.923876000
6	-0.354443000	1.792785000	5.285415000
6	0.893455000	2.319031000	5.977292000
1	-4.119523000	2.428230000	7.203941000
1	-5.047617000	1.640598000	5.887649000
1	-2.908493000	0.665154000	5.760240000
1	-3.029359000	1.756505000	4.386844000
1	-1.630468000	3.471611000	5.652780000
1	-1.539161000	2.362491000	7.025843000
1	-0.983953000	-0.048970000	4.776265000
1	0.917394000	2.003180000	7.037570000
1	0.915912000	3.423699000	5.949588000
1	1.805128000	1.952551000	5.476796000
6	2.015654000	-4.030132000	2.135984000
6	0.714865000	-3.725892000	2.840410000
8	-0.188994000	-3.042707000	2.257731000
8	0.492662000	-4.116663000	4.021676000
1	2.836105000	-3.499062000	2.655117000
1	1.978280000	-3.665073000	1.096654000
26	-1.520444000	-3.119255000	4.021166000
8	-0.321077000	0.336330000	5.391360000
17	-2.633581000	-1.089874000	3.474217000
8	4.002527000	1.141902000	5.108085000
1	3.346650000	0.426484000	5.230421000
1	4.826695000	0.657046000	4.844041000
8	-4.749263000	-0.849617000	5.918123000
1	-5.491425000	-1.503141000	5.849834000
1	-4.154508000	-0.996912000	5.150629000
8	1.823013000	-1.038964000	6.465186000
1	2.172685000	-0.356507000	7.094524000
1	1.008520000	-0.621681000	6.075560000
8	-3.676555000	-1.962883000	0.273560000
1	-3.080040000	-2.727296000	0.084795000
1	-3.491509000	-1.696358000	1.198331000
1	-0.405968000	9.083039000	-1.808461000

1	-2.639184000	-4.592705000	10.313992000
1	-1.880522000	0.639971000	10.161167000
1	-6.639281000	0.830519000	-1.944617000
1	4.765133000	-5.811164000	5.939138000
1	-11.145816000	-1.926911000	-1.610455000
1	-6.556272000	-6.878411000	1.839557000
1	-1.437505000	-1.463147000	-5.725198000
1	-2.924294000	3.552426000	-1.525105000
1	-4.158560000	6.111130000	5.888196000
1	-0.325392000	2.107966000	4.240627000
1	2.225842000	-5.101307000	2.148977000

[1]Re$_{\text{model 4, A}}$

7	-2.251248861	-4.379491942	-10.356928898
1	-1.882816795	-5.260889166	-10.734583523
6	-3.586053746	-4.047443691	-10.419871399
6	-1.594932513	-3.462946766	-9.635780679
1	-0.536404059	-3.502884471	-9.397320912
7	-2.442818915	-2.526209296	-9.212590278
6	-3.693774130	-2.873012126	-9.693893845
1	-4.571045608	-2.274449516	-9.460581671
7	-0.163754709	1.960713994	-9.752555329
1	0.679184420	2.435715812	-10.083762702
6	-1.435825118	2.005999025	-10.280434289
6	-0.120573089	0.965564014	-8.844985406
1	0.790452954	0.716878345	-8.304865883
7	-1.297985351	0.361488906	-8.742150100
6	-2.131401539	1.013462005	-9.623865746
1	-3.183789574	0.748697038	-9.677120286
16	-9.155841341	-2.221958255	-0.998303903
6	-7.587735606	-2.061797426	-1.985222071
8	-6.749502286	-1.395939975	-1.447348825
6	-7.360225135	-2.830701219	-3.266228738
6	-7.084664629	-1.895258980	-4.453301236
6	-8.316786108	-1.087383431	-4.869596855
6	-8.134152778	-0.200669458	-6.101428800
6	-7.746056062	-0.947253872	-7.374978915
1	-6.472240433	-3.452769338	-3.056193631
1	-8.191103426	-3.516659796	-3.488470111
1	-6.244967181	-1.218585988	-4.207555290
1	-6.752343052	-2.526702834	-5.292388024
1	-9.152927930	-1.789771031	-5.059847633
1	-8.640286586	-0.446058913	-4.025401474
1	-9.080730891	0.341931817	-6.264791516
1	-8.400627110	-1.823262029	-7.536666922
1	-6.699174757	-1.300883607	-7.353901610
1	-7.853761857	-0.293991485	-8.259193949
6	1.777786521	-1.974160504	-6.103523415
6	0.490662094	-1.555086085	-6.768989275
8	-0.329500914	-0.778328932	-6.198668389
8	0.121470616	-2.041032183	-7.892628402
1	1.604355583	-2.970809564	-5.656194837
1	2.007863575	-1.286548280	-5.275325968
26	-1.789050069	-1.423220359	-7.657563746
8	-2.076656999	-2.662902320	-6.624168057
17	-3.751075157	-0.395485179	-7.161356531
1	-4.312347202	-4.778307609	-10.775403499
1	-1.805477927	2.642729877	-11.084174265
1	-10.160552221	-2.202625833	-1.944099828
1	-7.386610035	0.563172601	-5.887437008
1	2.623997648	-2.066292253	-6.784336008

[3]Re$_{\text{model 4, A}}$

7	-2.174671987	-4.388177688	-10.431959030
1	-1.760336509	-5.252726887	-10.796854101
6	-3.532592291	-4.173601203	-10.400792866
6	-1.552050508	-3.392039819	-9.786717604
1	-0.481129918	-3.351896427	-9.615495442
7	-2.439475230	-2.512396447	-9.332189591
6	-3.682801053	-2.986734555	-9.709439729
1	-4.588968084	-2.452201556	-9.438929166
7	-0.370779412	2.165283959	-9.567708937
1	0.418663301	2.761551755	-9.808230755
6	-1.588385987	2.099246798	-10.206620111
6	-0.324402884	1.193320229	-8.637552168
1	0.542119797	1.016259295	-8.005919732
7	-1.460708604	0.506026840	-8.627842845
6	-2.265272979	1.071557888	-9.590794438

1	-3.278731222	0.712252463	-9.739707202
16	-9.119306870	-2.173575621	-1.063920945
6	-7.561497681	-2.147720460	-2.072667394
8	-6.661275421	-1.565723030	-1.536516928
6	-7.417529226	-2.901181310	-3.374881389
6	-7.123240243	-1.955760238	-4.550255223
6	-8.309884473	-1.055633776	-4.905712170
6	-8.097123161	-0.141508574	-6.113097348
6	-7.792693956	-0.867236171	-7.420772787
1	-6.564137809	-3.581408702	-3.207508320
1	-8.293781249	-3.531312026	-3.587682374
1	-6.229921418	-1.343629910	-4.324808231
1	-6.869341105	-2.587355632	-5.415283915
1	-9.195546929	-1.694826906	-5.097726712
1	-8.570922297	-0.423696554	-4.033491599
1	-9.010794260	0.463523985	-6.235264341
1	-8.513313939	-1.685758416	-7.600757478
1	-6.776001128	-1.300418391	-7.436176967
1	-7.867643108	-0.173542255	-8.277391317
6	1.748688815	-2.117711766	-6.120165310
6	0.473678859	-1.588818262	-6.742052730
8	-0.239380023	-0.739149717	-6.134847597
8	0.039141648	-2.040644386	-7.850449880
1	1.572090196	-3.173376011	-5.842778983
1	1.965844489	-1.560705350	-5.195733386
26	-1.897233769	-1.235157861	-7.622388440
8	-2.254875773	-2.562370708	-6.518681552
17	-3.821510094	-0.313243557	-6.825989529
1	-4.231048940	-4.944980101	-10.725138715
1	-1.917756639	2.698919246	-11.055133663
1	-10.148189455	-2.168490493	-1.983552601
1	-7.293624141	0.562491513	-5.896670627
1	2.605727068	-2.096947560	-6.793308388

$^{5}\text{Re}_{\text{model 4, A}}$

7	-2.228888366	-4.314257813	-10.458292361
1	-1.792213596	-5.157216199	-10.850338284
6	-3.590198998	-4.145959323	-10.398800048
6	-1.628540772	-3.313735969	-9.801168324
1	-0.554434318	-3.241070460	-9.670414415
7	-2.536705908	-2.478343637	-9.302400166
6	-3.770976318	-2.980958448	-9.677026760
1	-4.694159864	-2.475401809	-9.399024392
7	-0.388562212	2.121743096	-9.452142886
1	0.376824505	2.764082646	-9.643148605
6	-1.560030633	1.995443590	-10.164012998
6	-0.342797646	1.142055765	-8.525251310
1	0.496997853	1.012538856	-7.844318832
7	-1.437474505	0.393467838	-8.587675191
6	-2.211501718	0.925709627	-9.594116253
1	-3.194629029	0.518342377	-9.805114033
16	-9.107591166	-2.191275616	-1.074359332
6	-7.536053918	-2.092518208	-2.059874994
8	-6.673173114	-1.480390039	-1.496779394
6	-7.333831055	-2.821619829	-3.367323421
6	-7.074818530	-1.846593867	-4.527811049
6	-8.315166207	-1.039961196	-4.920345831
6	-8.140899391	-0.122932971	-6.131567412
6	-7.730421356	-0.834519454	-7.418249061
1	-6.439691015	-3.446879426	-3.198293608
1	-8.170450855	-3.499237392	-3.596115728
1	-6.238055449	-1.172808393	-4.267082169
1	-6.732070404	-2.447929588	-5.383064726
1	-9.145736659	-1.744494969	-5.127574648
1	-8.645221370	-0.421403164	-4.061866586
1	-9.096467442	0.404833576	-6.291022530
1	-8.363921265	-1.721530211	-7.600878639
1	-6.675790668	-1.166194291	-7.396063924
1	-7.849849327	-0.165077758	-8.288803797
6	1.718223769	-2.193763707	-6.075240675
6	0.412157866	-1.752156244	-6.701433804
8	-0.374799042	-0.972946084	-6.072867373
8	0.019397283	-2.195142013	-7.820310103
1	1.618122108	-3.263957763	-5.813322559
1	1.893555463	-1.638785643	-5.140873809
26	-2.029516926	-1.330377465	-7.504512689
8	-2.481902831	-2.609601461	-6.615353599
17	-3.679406090	-0.010223445	-6.650769496
1	-4.262376344	-4.939271591	-10.725756587
1	-1.898565444	2.610258101	-10.997958215
1	-10.128870003	-2.205880718	-2.002326916

1	-7.408505786	0.649546134	-5.897168271
1	2.573992896	-2.095896836	-6.743184233

^{1}TS$_{model\ 4,\ A}$

7	-2.307115167	-4.363898544	-10.229556453
1	-1.882720326	-5.209765077	-10.626664311
6	-3.651764093	-4.099734113	-10.301865433
6	-1.708113086	-3.412031617	-9.501147610
1	-0.645563583	-3.414505579	-9.291756411
7	-2.597146771	-2.515450017	-9.073588273
6	-3.820522750	-2.940640502	-9.563465375
1	-4.737180043	-2.410381345	-9.324965083
7	-0.510945907	2.276726507	-9.474802373
1	0.005299938	3.152296654	-9.432988940
6	-1.486145868	1.936670503	-10.385829019
6	-0.385873737	1.265655346	-8.595852678
1	0.311820139	1.281152889	-7.766997479
7	-1.231435962	0.284505401	-8.879818530
6	-1.932908751	0.695891885	-9.992500878
1	-2.729429770	0.085834206	-10.406074081
16	-9.221290169	-2.106374683	-1.123879982
6	-7.700994360	-1.697855498	-2.111442481
8	-6.969115173	-0.912200695	-1.581714290
6	-7.374004567	-2.501059534	-3.349819147
6	-5.916202459	-2.313903559	-3.777641385
6	-5.646750589	-1.067860564	-4.650916711
6	-5.429101887	-1.368487159	-6.129648161
6	-5.848604733	-0.308330114	-7.115425311
1	-7.596651677	-3.557628662	-3.119475197
1	-8.081979955	-2.227503816	-4.158120684
1	-5.287839618	-2.302472256	-2.874276135
1	-5.634311590	-3.223016065	-4.331375781
1	-6.492814251	-0.355377141	-4.566321134
1	-4.777223954	-0.500687653	-4.271735478
1	-4.158844069	-1.658436487	-6.242755310
1	-5.513318615	0.693014182	-6.823237479
1	-6.956591019	-0.305002701	-7.188918458
1	-5.454560017	-0.525627523	-8.123363923
6	1.707696248	-2.113290913	-6.027471993
6	0.382098084	-1.654326431	-6.600988194
8	-0.205757937	-0.621203062	-6.189275885
8	-0.219646151	-2.341622482	-7.494089326
1	1.633760507	-3.184506233	-5.776978475
1	1.921065865	-1.556471911	-5.101615000
26	-1.992079938	-1.108201037	-7.496592832
8	-2.903267666	-2.080313201	-6.313674158
17	-2.941983660	0.821879891	-6.644013299
1	-4.336442039	-4.854114091	-10.689414504
1	-1.864861594	2.579582172	-11.180376884
1	-10.221935395	-2.112136492	-2.074155499
1	-5.860399368	-2.314537735	-6.456814964
1	2.546805679	-2.011905812	-6.715718859

^{3}TS$_{model\ 4,\ A}$

7	-2.084727820	-4.547429978	-10.087180552
1	-1.636308930	-5.401748752	-10.430419314
6	-3.416327356	-4.262356997	-10.304211652
6	-1.528244580	-3.550795073	-9.380970346
1	-0.492300294	-3.520944560	-9.056865722
7	-2.438422029	-2.618577603	-9.121954037
6	-3.622903348	-3.048394955	-9.688815887
1	-4.542730011	-2.486720312	-9.563220911
7	-0.600908072	2.348143237	-9.537581855
1	-0.017310399	3.178697846	-9.473840339
6	-1.564692642	2.088291675	-10.483690704
6	-0.598316760	1.332712417	-8.653307225
1	0.066727304	1.286810916	-7.797253088
7	-1.517640101	0.433017091	-8.974376456
6	-2.126270260	0.890275606	-10.112972370
1	-2.930948086	0.325569508	-10.565611100
16	-9.101242410	-2.178755484	-1.218626041
6	-7.527431514	-1.921587469	-2.153942394
8	-6.735091377	-1.273328633	-1.529905731
6	-7.223777157	-2.642233361	-3.451034843
6	-5.829423719	-2.272645159	-3.972917135
6	-5.776877900	-0.898389724	-4.674487250
6	-5.968937533	-0.947903554	-6.184331702
6	-6.291902060	0.346368597	-6.892253606

1	-7.307566961	-3.723921300	-3.233146206
1	-8.018050718	-2.437494149	-4.194170228
1	-5.128884097	-2.292353159	-3.124863571
1	-5.494840640	-3.062919394	-4.665995882
1	-6.551181724	-0.229475407	-4.244533789
1	-4.818488344	-0.392903947	-4.459837511
1	-4.874761677	-1.324885208	-6.638054377
1	-5.494977654	1.093923702	-6.736343632
1	-7.242847229	0.776151505	-6.514838773
1	-6.421377365	0.179634567	-7.975470862
6	1.640640748	-2.614171790	-6.374075497
6	0.404391412	-1.705592340	-6.417688857
8	0.178982769	-0.956879765	-5.454074571
8	-0.351879026	-1.769103833	-7.462535402
1	1.472294289	-3.547431935	-6.937497573
1	1.826314842	-2.860582345	-5.317873877
26	-2.293388410	-1.087906486	-7.718356438
8	-3.761264074	-1.874499780	-7.132121150
17	-2.615675696	0.601573258	-6.202503332
1	-4.118046900	-5.012914029	-10.667971384
1	-1.903175625	2.744796225	-11.285250660
1	-10.114721047	-2.196133765	-2.155058985
1	-6.618918730	-1.754153248	-6.524262869
1	2.523809615	-2.124480456	-6.784297960

$^{5}TS_{model\ 4,\ A}$

7	-2.223978024	-4.407870596	-10.323630562
1	-1.794318526	-5.260018521	-10.700166073
6	-3.577461786	-4.176206200	-10.365285750
6	-1.630258778	-3.437605690	-9.615168437
1	-0.567230888	-3.415197906	-9.405098711
7	-2.530870888	-2.561491898	-9.174295604
6	-3.753659082	-3.008073787	-9.646283736
1	-4.675924644	-2.475533944	-9.426946503
7	-0.465518337	2.289169100	-9.424510547
1	0.106969831	3.129218345	-9.424858216
6	-1.472306851	1.978431224	-10.310248442
6	-0.365751138	1.283736952	-8.531014008
1	0.364331985	1.283894683	-7.724062308
7	-1.263873643	0.342327874	-8.782399724
6	-1.965606846	0.766326483	-9.885802509
1	-2.785495872	0.177324550	-10.282745032
16	-9.353023617	-2.281170300	-1.034912131
6	-7.858017903	-1.406280305	-1.641405436
8	-7.173071854	-1.003107716	-0.738894701
6	-7.493651950	-1.313757151	-3.108467596
6	-6.019599153	-0.935164066	-3.303373726
6	-5.679663419	-0.501575300	-4.732361593
6	-5.618625263	-1.579689578	-5.808960705
6	-6.133229791	-1.123295913	-7.164162673
1	-7.752482465	-2.267745073	-3.602327947
1	-8.155108250	-0.543237854	-3.557542041
1	-5.790760590	-0.090195648	-2.633797625
1	-5.376745714	-1.775424019	-2.983372290
1	-6.427735594	0.248386344	-5.055945512
1	-4.720256494	0.042737794	-4.725442029
1	-4.484533097	-1.883423963	-5.986558283
1	-5.549630842	-0.256384938	-7.516270388
1	-7.191804961	-0.806363192	-7.090223917
1	-6.083085892	-1.924174559	-7.920321893
6	1.690393141	-2.375984231	-6.025509375
6	0.365249192	-1.882741700	-6.564807451
8	-0.395180341	-1.133761716	-5.864203751
8	-0.072754807	-2.223730967	-7.696487384
1	1.639185548	-3.478046166	-5.955861791
1	1.853424566	-1.978030711	-5.012235370
26	-2.002420305	-1.219933516	-7.397191050
8	-3.080486227	-2.247343972	-6.443127392
17	-2.918132101	0.784956485	-6.406202361
1	-4.265146523	-4.947435849	-10.712217799
1	-1.851161788	2.611964713	-11.112228226
1	-10.303001243	-2.154653682	-2.027828888
1	-6.077675478	-2.518340780	-5.498679948
1	2.535261487	-2.124287216	-6.666534660

$^{1}Int_{model\ 4,\ A}$

7	-2.266545550	-4.522482133	-10.219919622
1	-1.915915007	-5.410671892	-10.597313071
6	-3.578297838	-4.127003501	-10.350399438
6	-1.600064850	-3.626174801	-9.484899831

1	-0.555976314	-3.715440160	-9.203850217
7	-2.417272725	-2.641371733	-9.114475958
6	-3.660562617	-2.939641770	-9.642637441
1	-4.513717079	-2.303526038	-9.429174070
7	-0.517870567	2.228204277	-9.443092304
1	0.014341481	3.093881089	-9.415102770
6	-1.515901738	1.905592214	-10.334998835
6	-0.397654721	1.218140738	-8.562657194
1	0.332665907	1.212486405	-7.762018250
7	-1.274867729	0.258543822	-8.827196726
6	-1.987866570	0.679859084	-9.927341336
1	-2.802647374	0.082285332	-10.320345218
16	-9.096782726	-2.154410462	-1.354070016
6	-7.640332846	-1.730983254	-2.420929282
8	-6.848029311	-1.003115738	-1.891265952
6	-7.393995830	-2.455119535	-3.728936255
6	-6.366776529	-1.731329388	-4.609436999
6	-6.979613155	-0.686370843	-5.550648011
6	-7.781000716	-1.226590344	-6.693737471
6	-8.332284715	-0.283188469	-7.708495151
1	-7.048500381	-3.463162633	-3.420768804
1	-8.331878730	-2.642288866	-4.278663192
1	-5.619166193	-1.241268842	-3.959546085
1	-5.832502906	-2.475134606	-5.226864789
1	-7.601867343	0.021006114	-4.960580119
1	-6.149310313	-0.069537981	-5.955540347
1	-3.062502090	-2.759317092	-6.439173946
1	-8.904235747	0.540644086	-7.238250653
1	-8.981458520	-0.783830153	-8.446686253
1	-7.517375760	0.208249541	-8.282901291
6	1.804177505	-1.998483507	-6.067007952
6	0.519044690	-1.598993832	-6.744497328
8	-0.252777531	-0.706991409	-6.279359781
8	0.106311656	-2.204062523	-7.787208156
1	1.654438839	-3.001492769	-5.628679694
1	2.031595820	-1.306023326	-5.242438856
26	-1.705130865	-1.398869644	-7.683895352
8	-2.099525171	-2.649525355	-6.402586017
17	-3.732899485	-0.404029772	-7.263370136
1	-4.317978966	-4.831101358	-10.731431410
1	-1.889365443	2.549861604	-11.130931700
1	-10.158135040	-2.141471464	-2.235949522
1	-7.752803205	-2.288775810	-6.936707147
1	2.642198769	-2.070767965	-6.760234212

$^{3}\mathbf{Int}_{\mathbf{model\ 4,\ A}}$

7	-2.092030222	-4.437693407	-10.110616918
1	-1.645096786	-5.286709603	-10.470856648
6	-3.435295413	-4.175137442	-10.265531716
6	-1.524075431	-3.442654178	-9.411037467
1	-0.474578598	-3.406524792	-9.133938155
7	-2.437381588	-2.528865692	-9.095268263
6	-3.638253933	-2.974686359	-9.614063944
1	-4.561502478	-2.431475742	-9.436150726
7	-0.490062943	2.366737647	-9.573481732
1	0.116723155	3.182285620	-9.542330676
6	-1.502309069	2.124629456	-10.471500535
6	-0.445266380	1.340195587	-8.706115437
1	0.268536456	1.275587791	-7.891188649
7	-1.385047788	0.449373325	-8.996624756
6	-2.053754001	0.925876529	-10.094013071
1	-2.890026132	0.379766312	-10.511186918
16	-9.049094834	-2.137128189	-1.293417699
6	-7.583310917	-1.649953982	-2.360707180
8	-6.893519822	-0.790578374	-1.894469681
6	-7.237876911	-2.498550154	-3.562547782
6	-6.141127814	-1.885548742	-4.439799315
6	-6.650565895	-0.830591551	-5.436038967
6	-7.594488347	-1.313868595	-6.492760371
6	-8.202915853	-0.338300740	-7.443009592
1	-6.914681340	-3.466160057	-3.128624259
1	-8.142261684	-2.747007720	-4.145734523
1	-5.368714026	-1.442023985	-3.788402887
1	-5.648270858	-2.703020343	-4.991665473
1	-7.118896227	0.008188198	-4.879952673
1	-5.765653675	-0.363876956	-5.928730714
1	-3.856309869	-1.123689028	-6.192788717
1	-7.423775592	0.190793476	-8.035281751
1	-8.765484853	0.460124370	-6.917484741

1	-8.875584900	-0.823774621	-8.169914762
6	1.664863535	-2.590641019	-6.275187981
6	0.433106702	-1.687938706	-6.349285951
8	0.134020705	-0.979536129	-5.376799791
8	-0.257514101	-1.699383884	-7.444120441
1	1.477032134	-3.554259644	-6.779184939
1	1.872700207	-2.773629702	-5.211070404
26	-2.139350167	-0.967082456	-7.724302173
8	-3.518610793	-1.764526095	-6.846132534
17	-2.572766221	0.818107937	-6.331062404
1	-4.122422027	-4.937144669	-10.633321498
1	-1.863949023	2.782249041	-11.261956320
1	-10.096485573	-2.114453293	-2.191641558
1	-7.635336476	-2.373211533	-6.746120383
1	2.537028759	-2.134096400	-6.743121906

^{5}Int$_{\text{model 4, A}}$

7	-2.205605350	-4.443720915	-10.333244956
1	-1.793659044	-5.301831017	-10.716621998
6	-3.559691845	-4.205568970	-10.338912709
6	-1.584995146	-3.473392534	-9.653230516
1	-0.517983938	-3.450483207	-9.460655634
7	-2.471082219	-2.587580474	-9.206029988
6	-3.712611433	-3.031602658	-9.627814710
1	-4.617306590	-2.486477885	-9.372115845
7	-0.389610422	2.334328192	-9.425120847
1	0.213440003	3.150531821	-9.471362869
6	-1.435797817	2.033064446	-10.268754733
6	-0.287217101	1.343917248	-8.512103335
1	0.480370988	1.331655592	-7.740135257
7	-1.223957354	0.428671863	-8.706143996
6	-1.950999104	0.849587003	-9.796792224
1	-2.806836019	0.283793520	-10.151088986
16	-9.480765856	-2.337180018	-0.745323448
6	-8.091899102	-1.430242748	-1.592493181
8	-7.206738465	-1.083902990	-0.859904769
6	-8.050689445	-1.286127372	-3.097454036
6	-6.719298786	-1.800913286	-3.676489863
6	-6.480685842	-1.278543786	-5.101903402
6	-7.539331345	-1.622620522	-6.100752400
6	-7.918588526	-0.667391245	-7.181134382
1	-8.902572496	-1.787418081	-3.579843634
1	-8.151491403	-0.203904483	-3.303041969
1	-5.890744831	-1.471064391	-3.026664482
1	-6.724853628	-2.906843147	-3.665448733
1	-6.348864948	-0.180793328	-5.068474349
1	-5.489828680	-1.662384811	-5.435353190
1	-3.552055071	-2.628097587	-6.512051194
1	-7.022147825	-0.335965184	-7.748810419
1	-8.372752995	0.260941646	-6.774262902
1	-8.627556777	-1.108171292	-7.901439733
6	1.744406057	-2.237063016	-6.041416057
6	0.448473252	-1.775755718	-6.665865986
8	-0.338885650	-0.984780979	-6.036076446
8	0.061984997	-2.192640729	-7.789573599
1	1.644593951	-3.315482519	-5.818519418
1	1.919795961	-1.709288950	-5.091143308
26	-1.934155080	-1.334765747	-7.505774332
8	-2.596057137	-2.669059822	-6.331200038
17	-3.746648621	0.038392841	-7.069240816
1	-4.256784152	-4.966712347	-10.689327306
1	-1.824994968	2.648492983	-11.079828481
1	-10.598891903	-1.776852750	-1.328568774
1	-7.873757497	-2.658269363	-6.161233391
1	2.595368937	-2.114760327	-6.711469455

^{1}TSCl$_{\text{model 4, A}}$

7	-2.193696073	-4.550484209	-10.102895857
1	-1.777646887	-5.408467647	-10.477615511
6	-3.513040679	-4.216786237	-10.293063125
6	-1.605478746	-3.608084898	-9.352260501
1	-0.569416654	-3.638653409	-9.029961536
7	-2.482698020	-2.660342738	-9.030405113
6	-3.680030111	-3.026806100	-9.609820021
1	-4.573425027	-2.428349294	-9.458776091
7	-0.698515456	2.288614861	-9.390597064
1	-0.223315138	3.185443920	-9.318138771
6	-1.583605998	1.908342517	-10.372312176
6	-0.655308890	1.313108951	-8.462405860
1	-0.032518974	1.358310091	-7.577839563

7	-1.472487502	0.318163947	-8.780797730
6	-2.057025348	0.676398159	-9.974638812
1	-2.780157315	0.022885810	-10.450284649
16	-9.007336753	-2.167596485	-1.313110200
6	-7.595711715	-1.546192367	-2.392828139
8	-6.981604709	-0.617517009	-1.957278125
6	-7.177469888	-2.383727475	-3.582149667
6	-6.256103118	-1.647617495	-4.563037590
6	-6.994889428	-0.698875053	-5.530557696
6	-6.792427906	-0.923508255	-6.991051851
6	-7.542019079	-0.067214427	-7.946503499
1	-6.654539645	-3.247023244	-3.121457479
1	-8.050891940	-2.837093215	-4.082323727
1	-5.492459982	-1.090407087	-3.996908243
1	-5.705411681	-2.406836773	-5.138998949
1	-8.097710167	-0.778332218	-5.374609441
1	-6.779281339	0.357871244	-5.301981991
1	-3.283138305	-2.700265706	-6.447172603
1	-8.574212403	-0.475132953	-8.035793254
1	-7.132611845	-0.101389853	-8.966679906
1	-7.640660724	0.975308029	-7.608348034
6	1.748403324	-1.898765277	-6.013989519
6	0.441693814	-1.471524325	-6.658744075
8	-0.305259547	-0.584100942	-6.143197061
8	0.021727801	-2.062249460	-7.698444324
1	1.600016573	-2.915916410	-5.609831771
1	1.991270445	-1.242005674	-5.165137898
26	-1.893670369	-1.317294388	-7.577985907
8	-2.356944831	-2.533056390	-6.213576831
17	-4.236390973	-0.398811904	-7.294820844
1	-4.218137872	-4.952378803	-10.680114594
1	-1.919865336	2.543972734	-11.191440499
1	-10.062790799	-2.118949464	-2.200802622
1	-6.597370352	-1.940206730	-7.332144345
1	2.584020651	-1.952871269	-6.711759931

$^{3}TSCl_{model\ 4,\ A}$

7	-2.119667732	-4.418590013	-10.222983452
1	-1.669660606	-5.285807313	-10.529330316
6	-3.464491450	-4.176702828	-10.381955299
6	-1.558397782	-3.363805551	-9.602449561
1	-0.509586012	-3.309670618	-9.319262955
7	-2.468600077	-2.433263350	-9.344663627
6	-3.665511387	-2.928646657	-9.824494346
1	-4.589071158	-2.362943669	-9.734410497
7	-0.290882013	2.230866674	-9.663049971
1	0.530607313	2.758505133	-9.951839792
6	-1.535597900	2.231673605	-10.250796670
6	-0.266171918	1.259554451	-8.727903685
1	0.619670242	1.016610255	-8.145957839
7	-1.439728800	0.645314086	-8.663132708
6	-2.243394696	1.248239223	-9.601708761
1	-3.281357211	0.949642451	-9.706279699
16	-9.104318930	-1.991162133	-1.277171285
6	-7.559246841	-1.920771891	-2.281355237
8	-6.663790145	-1.373347520	-1.701636750
6	-7.426625799	-2.639914771	-3.604374471
6	-5.971210063	-2.709443396	-4.075869833
6	-5.381150062	-1.436635367	-4.740289700
6	-5.855135309	-1.176242404	-6.136751513
6	-7.062869549	-0.369840826	-6.454436899
1	-7.840033174	-3.655549938	-3.468297224
1	-8.091340868	-2.150285980	-4.342885868
1	-5.329071310	-2.957833550	-3.214864381
1	-5.886928479	-3.564730549	-4.763845629
1	-5.562156441	-0.554516124	-4.101102126
1	-4.293402704	-1.594886692	-4.780299511
1	-3.179723715	-2.839527414	-6.901150404
1	-7.021801457	0.058179758	-7.470116676
1	-7.244066877	0.438630270	-5.727934624
1	-7.958545038	-1.032624514	-6.448500446
6	1.712409312	-2.502043920	-6.212450668
6	0.493377879	-1.625307631	-6.479210967
8	0.001835086	-0.944022070	-5.555130719
8	-0.009482814	-1.608681618	-7.662094017
1	1.487144526	-3.543714115	-6.503974197
1	1.927482382	-2.479644905	-5.133874434
26	-1.933583981	-1.038606482	-7.627583664
8	-2.324905318	-2.523457728	-6.572584330

17	-3.807713512	0.118981657	-6.890985565
1	-4.152494572	-4.954575206	-10.713064927
1	-1.871445607	2.820460936	-11.104379189
1	-10.148963213	-2.069328783	-2.175478538
1	-5.614613089	-1.934904643	-6.881498444
1	2.586231940	-2.187940895	-6.783275182

^{5}TSCl$_{\text{model 4, A}}$

7	-2.247301083	-4.411226247	-10.306435990
1	-1.827634107	-5.283430154	-10.642210348
6	-3.593213726	-4.143586090	-10.404145505
6	-1.650099601	-3.423105328	-9.620959732
1	-0.596912376	-3.413622265	-9.354405420
7	-2.540279940	-2.503589207	-9.259484256
6	-3.759694100	-2.942305818	-9.743048697
1	-4.668904393	-2.373907401	-9.568819236
7	-0.298286394	2.250476384	-9.566088210
1	0.501092156	2.823467721	-9.824676806
6	-1.510655856	2.170156099	-10.213194938
6	-0.270713142	1.299967506	-8.604192316
1	0.594748359	1.138938932	-7.962367709
7	-1.408745038	0.625176347	-8.575966049
6	-2.192912012	1.163483554	-9.568959605
1	-3.210087492	0.811344745	-9.713019819
16	-9.297074891	-2.082455580	-0.945199426
6	-7.934824959	-1.802667927	-2.201648861
8	-6.928366054	-1.339957600	-1.749477930
6	-8.098791916	-2.330568536	-3.613718286
6	-6.747150892	-2.652293052	-4.267582855
6	-5.975980781	-1.469945742	-4.924894073
6	-6.425577604	-1.168884486	-6.316453730
6	-7.567041019	-0.282158500	-6.642507859
1	-8.726335241	-3.236892109	-3.564129874
1	-8.678055384	-1.580693020	-4.191236898
1	-6.094864832	-3.109900089	-3.505878503
1	-6.926823952	-3.440114815	-5.014849490
1	-6.043575491	-0.571293265	-4.286987473
1	-4.912416613	-1.753249360	-4.969218758
1	-3.501774083	-2.764098886	-6.612365965
1	-7.457704908	0.186272175	-7.636490892
1	-7.737048259	0.505171875	-5.890350192
1	-8.499733293	-0.889408512	-6.707382328
6	1.702708541	-2.233900939	-6.108158109
6	0.420225797	-1.677789298	-6.706203876
8	-0.354766153	-0.960749776	-5.986273850
8	0.055126527	-1.954336413	-7.880824961
1	1.556234876	-3.318865353	-5.948711204
1	1.885010891	-1.776069838	-5.123090310
26	-2.022847647	-1.245001764	-7.491596949
8	-2.582993884	-2.671675550	-6.314898822
17	-3.974692726	0.030345235	-6.940174570
1	-4.299322362	-4.896955747	-10.753304602
1	-1.856283991	2.758200089	-11.063378906
1	-10.436422026	-1.649539578	-1.592362045
1	-6.150536517	-1.880393418	-7.095025988
1	2.571933650	-2.110559258	-6.754147161

^{1}TSOH$_{\text{model 4, A}}$

7	-2.213962043	-4.526195730	-10.145224358
1	-1.811892058	-5.388429964	-10.525833131
6	-3.529464452	-4.174186743	-10.325134124
6	-1.607849888	-3.589056509	-9.401038647
1	-0.561097401	-3.626472939	-9.117847097
7	-2.469328500	-2.631536022	-9.066343964
6	-3.677616783	-2.980732706	-9.640634134
1	-4.555770118	-2.353901543	-9.503475969
7	-0.532661479	2.370024605	-9.423649882
1	-0.146279054	3.300423224	-9.277257087
6	-1.383133632	1.989948401	-10.436966168
6	-0.435229089	1.339475498	-8.559528089
1	0.162365423	1.375403125	-7.657418783
7	-1.171710578	0.311395347	-8.957011420
6	-1.769098573	0.708089860	-10.133332460
1	-2.505078815	0.085110260	-10.629255967
16	-9.083001310	-2.001746556	-1.594785180
6	-7.793900845	-1.171841734	-2.630601542
8	-6.936028583	-0.637615817	-1.979674245
6	-7.719432880	-1.359723746	-4.128234721
6	-6.956999242	-2.666460852	-4.468856120
6	-5.415048458	-2.618055972	-4.283748781

6	-4.795469208	-1.571170560	-5.154974033
6	-4.277751303	-0.314741335	-4.568253621
1	-8.721927406	-1.399167935	-4.582834312
1	-7.211693236	-0.474056977	-4.543804632
1	-7.371599282	-3.506366934	-3.884353713
1	-7.186489355	-2.892948387	-5.523349582
1	-5.150008223	-2.458653174	-3.223158006
1	-5.038419669	-3.624278563	-4.553416872
1	-2.880201988	-3.015372310	-6.522203840
1	-5.027263025	0.106407856	-3.864518610
1	-4.027469949	0.421956154	-5.344613172
1	-3.387140368	-0.503077841	-3.943728003
6	1.720349839	-1.912941914	-5.973839328
6	0.390789577	-1.433454573	-6.548309212
8	-0.205858861	-0.431373530	-6.079402537
8	-0.159586676	-2.103022864	-7.486674785
1	1.603099104	-2.956406121	-5.636812032
1	1.986649246	-1.306585899	-5.094648498
26	-1.978754271	-1.138321605	-7.695814265
8	-2.620159716	-2.124159907	-6.237588699
17	-3.880610816	0.134622121	-7.952322977
1	-4.240841920	-4.901758782	-10.715836493
1	-1.793970872	2.644834966	-11.205343467
1	-10.207935604	-2.037639675	-2.393272589
1	-5.102033352	-1.568501067	-6.200956166
1	2.546030054	-1.916939737	-6.685389409

^{3}TSOH$_{\text{model 4, A}}$

7	-2.108107146	-4.527852478	-9.956210331
1	-1.651678095	-5.365889066	-10.327996814
6	-3.435090695	-4.232816900	-10.184860158
6	-1.563534458	-3.562537506	-9.201132717
1	-0.529480743	-3.539274525	-8.870677914
7	-2.477325707	-2.637133981	-8.915524061
6	-3.652287518	-3.042411225	-9.521764372
1	-4.569141803	-2.472902266	-9.398493373
7	-0.559302939	2.356043504	-9.608106440
1	0.053695171	3.165693908	-9.566525999
6	-1.561214604	2.121251676	-10.519557026
6	-0.547639418	1.332038626	-8.732028802
1	0.153346158	1.265153707	-7.906690896
7	-1.497013904	0.454619408	-9.020065794
6	-2.134692069	0.933087083	-10.132685350
1	-2.967919632	0.388138272	-10.558365922
16	-9.102609862	-2.181917762	-1.210250409
6	-7.567335136	-1.852467671	-2.202204306
8	-6.808288952	-1.095966327	-1.666688683
6	-7.246724611	-2.666444749	-3.439637730
6	-5.911478335	-2.239313078	-4.056939946
6	-5.999134032	-0.953803626	-4.902370008
6	-6.196185601	-1.133436050	-6.373256918
6	-6.437404409	0.046962036	-7.238326388
1	-7.222296943	-3.722817362	-3.112393482
1	-8.082618317	-2.617234123	-4.163030210
1	-5.187167568	-2.096084683	-3.242061863
1	-5.521373979	-3.066865617	-4.669550783
1	-6.825791331	-0.308378904	-4.527798899
1	-5.106112467	-0.319804984	-4.727420914
1	-3.978952412	-0.602852322	-6.332103290
1	-6.522313073	-0.239301980	-8.297453549
1	-5.633471939	0.798321518	-7.122216460
1	-7.384812414	0.555348149	-6.950704356
6	1.652474453	-2.677055626	-6.341150284
6	0.454006658	-1.721815649	-6.379884916
8	0.262674215	-0.972289867	-5.410018977
8	-0.303297729	-1.759269778	-7.423638096
1	1.415422668	-3.633825571	-6.837620950
1	1.880334869	-2.864268631	-5.282102231
26	-2.219006674	-1.057920181	-7.648835052
8	-3.938343292	-1.396779129	-6.894083290
17	-2.357500329	0.805623402	-6.157890038
1	-4.118632599	-4.982931937	-10.582560065
1	-1.900678083	2.783383306	-11.316057862
1	-10.121783251	-2.181021315	-2.140644096
1	-6.537454819	-2.094623524	-6.757602180
1	2.528652386	-2.259798434	-6.837412296

^{5}TSOH$_{\text{model 4, A}}$

7	-2.145570138	-4.415886864	-10.265112893
1	-1.670333487	-5.236548044	-10.653553294
6	-3.506524401	-4.253067846	-10.300225841
6	-1.604344613	-3.411390915	-9.561519775
1	-0.543213099	-3.347128001	-9.354771346
7	-2.542670852	-2.577144946	-9.119815796
6	-3.739815371	-3.092315868	-9.588899078
1	-4.686455673	-2.602679992	-9.383652920
7	-0.454042938	2.373887694	-9.428769360
1	0.081997999	3.237533784	-9.416442875
6	-1.445590475	2.033456603	-10.320542411
6	-0.337735721	1.373466230	-8.530004872
1	0.384992821	1.393912766	-7.716975994
7	-1.205839462	0.406779202	-8.784512258
6	-1.909676309	0.810264763	-9.895403028
1	-2.714063997	0.202017569	-10.298060802
16	-9.352002050	-2.243762988	-1.087594658
6	-7.896302528	-1.327099296	-1.712002062
8	-7.192442075	-0.928279687	-0.820693645
6	-7.557213520	-1.203197831	-3.185929101
6	-6.069880681	-0.882972833	-3.373087468
6	-5.674522981	-0.521221831	-4.807025317
6	-5.702882574	-1.598509407	-5.814919521
6	-6.017473813	-1.277306787	-7.221330834
1	-7.847790209	-2.133785736	-3.706162254
1	-8.193597471	-0.396887911	-3.606197238
1	-5.809511308	-0.030070264	-2.726699983
1	-5.474682304	-1.741572445	-3.016882132
1	-6.293285250	0.317973036	-5.177154815
1	-4.640020745	-0.110066079	-4.812215001
1	-3.311799667	-3.005810220	-6.884160116
1	-5.283962151	-0.534633230	-7.598455849
1	-7.006592241	-0.775952047	-7.292700695
1	-6.032938997	-2.158301148	-7.878468582
6	1.721087861	-2.290288765	-5.943926907
6	0.384831338	-1.817208246	-6.484299562
8	-0.301775074	-0.946940769	-5.861171470
8	-0.107082811	-2.312610625	-7.536521843
1	1.671392614	-3.384950691	-5.804540969
1	1.917805662	-1.826822328	-4.964881955
26	-2.007389168	-1.082723142	-7.344233764
8	-3.112491798	-2.273583703	-6.275403805
17	-2.946378159	0.893649085	-6.348473685
1	-4.156973316	-5.052133086	-10.655863682
1	-1.833976492	2.656147510	-11.126445420
1	-10.319917098	-2.131029945	-2.064722771
1	-5.646091774	-2.631037394	-5.470350100
1	2.549067843	-2.088022181	-6.623341879

$^{1}\mathbf{PrCl}_{\mathbf{model\ 4,\ A}}$

7	-2.240268582	-4.599437544	-10.132998538
1	-1.862349140	-5.484656290	-10.484152104
6	-3.536231941	-4.203653005	-10.356271570
6	-1.620095431	-3.675356164	-9.386565718
1	-0.594346417	-3.752108371	-9.038921247
7	-2.452634852	-2.678008436	-9.096374053
6	-3.656198582	-2.993713255	-9.695749157
1	-4.526416561	-2.351230388	-9.583368287
7	-0.652274974	2.301854563	-9.418911841
1	-0.136894699	3.177611557	-9.374094407
6	-1.601896290	1.956289484	-10.352183728
6	-0.578217629	1.310395166	-8.509782104
1	0.110199448	1.317716619	-7.673356877
7	-1.435959449	0.339171146	-8.792678500
6	-2.085032885	0.732684108	-9.942115324
1	-2.855102598	0.107120970	-10.382685447
16	-9.089917281	-2.145114643	-1.148230364
6	-7.671425914	-1.614435330	-2.272262209
8	-7.026736702	-0.674541948	-1.913428032
6	-7.311839189	-2.538913958	-3.412183215
6	-6.568870072	-1.871134313	-4.570657160
6	-7.465416254	-1.026660554	-5.494324888
6	-7.021355937	-0.971236085	-6.955397258
6	-7.852566705	-0.034949935	-7.814796145
1	-6.662069467	-3.296127173	-2.926743752
1	-8.184698212	-3.115241969	-3.761009143
1	-5.739691339	-1.257168184	-4.181806419
1	-6.103076065	-2.686301025	-5.144966031
1	-8.486614402	-1.459150706	-5.520221806
1	-7.578722617	-0.001268247	-5.100351590
1	-3.412931668	-2.489327891	-6.557220462

1	-7.868359995	0.988227659	-7.409174068
1	-8.892900009	-0.401057182	-7.848438064
1	-7.495258146	-0.015557435	-8.856509002
6	1.889970642	-1.992969596	-6.057733470
6	0.604196533	-1.627269000	-6.762596301
8	-0.163547759	-0.721921759	-6.302608125
8	0.231979685	-2.269574878	-7.785374413
1	1.732245855	-2.958462536	-5.546015630
1	2.136983756	-1.249425522	-5.285076514
26	-1.691729504	-1.399967565	-7.677405337
8	-2.501462328	-2.384893131	-6.241980235
17	-5.217329978	-0.357616408	-7.012146713
1	-4.263492215	-4.920709847	-10.737046176
1	-1.938501302	2.588803318	-11.173579269
1	-10.127105239	-2.099622143	-2.057361371
1	-7.061059635	-1.973704597	-7.381481550
1	2.714496473	-2.131557371	-6.757031616

^{3}PrCl$_{\text{model 4, A}}$

7	-2.126215745	-4.247866320	-10.612480990
1	-1.686335542	-5.090925329	-10.993806650
6	-3.486272301	-4.115378916	-10.473202051
6	-1.521613270	-3.199930004	-10.027689318
1	-0.443878904	-3.097462609	-9.927361572
7	-2.424322319	-2.372830933	-9.507621480
6	-3.659309303	-2.931609849	-9.783883575
1	-4.582525124	-2.453364810	-9.466243888
7	-0.329953778	2.227668707	-9.590881450
1	0.453232985	2.833070674	-9.826378926
6	-1.545185930	2.150088899	-10.232783598
6	-0.277228706	1.242327906	-8.670596324
1	0.601225531	1.054797718	-8.058200396
7	-1.399735774	0.537144944	-8.666527575
6	-2.202986272	1.101915930	-9.629247065
1	-3.204859846	0.719703713	-9.800322894
16	-9.142317460	-2.069862829	-1.207656615
6	-7.577439690	-1.842786018	-2.145625257
8	-6.729068964	-1.314824311	-1.480996169
6	-7.363380847	-2.417900684	-3.527799469
6	-5.871826041	-2.488239183	-3.873369126
6	-5.190437190	-1.156355330	-4.289375237
6	-4.957174261	-1.072265912	-5.799140953
6	-6.198948980	-0.821687747	-6.636317871
1	-7.820580946	-3.423811462	-3.554119439
1	-7.936103314	-1.804707011	-4.252341169
1	-5.334465680	-2.912081134	-3.010954569
1	-5.753499828	-3.238358173	-4.671994783
1	-5.778357157	-0.282962341	-3.954741844
1	-4.213096951	-1.095394604	-3.788303507
1	-2.779601721	-3.418600680	-7.440002447
1	-5.952167181	-0.741401795	-7.707431902
1	-6.722546595	0.098952558	-6.328209662
1	-6.896741829	-1.673577164	-6.524355861
6	1.862888520	-2.349953484	-6.254641451
6	0.657125058	-1.762303805	-6.961955426
8	-0.185715697	-1.084260778	-6.273670411
8	0.434812425	-1.973712640	-8.179279982
1	1.620172141	-3.394465626	-5.980864024
1	2.055630342	-1.797273039	-5.319653532
26	-1.665190165	-1.358963974	-7.848461301
8	-2.205910643	-2.877960230	-6.877988247
17	-3.757859818	0.386359480	-6.118097841
1	-4.172718081	-4.912579689	-10.758386969
1	-1.886414846	2.752531939	-11.074622377
1	-10.159934613	-2.114345918	-2.138690559
1	-4.443936211	-1.973926216	-6.133300674
1	2.748839515	-2.384755926	-6.888634309

^{5}PrCl$_{\text{model 4, A}}$

7	-2.213509776	-4.493913309	-10.326496687
1	-1.789570689	-5.369009868	-10.648197650
6	-3.562587645	-4.248087161	-10.403168152
6	-1.616534338	-3.461102477	-9.709469294
1	-0.558567457	-3.426906070	-9.462813100
7	-2.509369379	-2.536827413	-9.375948760
6	-3.731723505	-3.015552543	-9.806302195
1	-4.647319968	-2.445540442	-9.661361430
7	-0.397734748	2.150913251	-9.514897032

1	0.431470650	2.666623939	-9.802177396
6	-1.621305860	2.116577728	-10.145759357
6	-0.408734787	1.234369413	-8.522783778
1	0.454785469	1.048203497	-7.885597245
7	-1.578820087	0.612847247	-8.462462583
6	-2.345251400	1.160116066	-9.466920389
1	-3.374162576	0.840255952	-9.609852942
16	-9.223763321	-2.076542876	-0.968451480
6	-7.925411107	-1.806602714	-2.284658827
8	-6.848912101	-1.511093676	-1.851054688
6	-8.233879141	-2.108725421	-3.737936549
6	-6.987686246	-2.503258625	-4.542309092
6	-6.017017255	-1.375423969	-4.969446971
6	-6.217084356	-0.902883212	-6.413223120
6	-7.488619951	-0.117763396	-6.683735974
1	-8.968603608	-2.932104974	-3.762828226
1	-8.741638560	-1.217580080	-4.160315341
1	-6.432714247	-3.259790026	-3.964304410
1	-7.352870561	-3.039886129	-5.435386052
1	-6.078862030	-0.510164217	-4.286893824
1	-4.984440446	-1.748951001	-4.906690021
1	-3.737102269	-2.573161945	-6.796852955
1	-7.532848501	0.241587655	-7.724279387
1	-7.590726934	0.747502325	-6.009327119
1	-8.361792252	-0.780259497	-6.531239600
6	1.782208146	-2.404696664	-6.133300643
6	0.516640357	-1.783777347	-6.693844587
8	-0.203774273	-1.042090944	-5.958182434
8	0.142287465	-2.035218234	-7.879810787
1	1.633045811	-3.498404827	-6.067218845
1	1.964198038	-2.025167671	-5.115914640
26	-1.873636274	-1.369971613	-7.577639968
8	-2.857163511	-2.517964292	-6.394973257
17	-4.727526173	0.218552220	-6.787852724
1	-4.263193087	-5.015562546	-10.732107153
1	-1.931412264	2.687941252	-11.020656527
1	-10.407291083	-1.640680405	-1.528481445
1	-6.184757463	-1.753792206	-7.093646646
1	2.644621564	-2.229992409	-6.776564850

^{1}PrOH$_{\text{model 4, A}}$

7	-2.217120601	-4.599121943	-10.249759138
1	-1.861981854	-5.484427664	-10.624180840
6	-3.524434246	-4.200318945	-10.399626533
6	-1.561445103	-3.685321878	-9.524445056
1	-0.512269999	-3.753662353	-9.260318806
7	-2.381674749	-2.698224463	-9.173260904
6	-3.614453816	-3.004184926	-9.714190857
1	-4.465590404	-2.353804005	-9.534939266
7	-0.441002577	2.498521700	-9.496885341
1	-0.039405612	3.423709775	-9.363332227
6	-1.381874211	2.150257865	-10.438336833
6	-0.294753654	1.453685559	-8.655577760
1	0.387785665	1.458810233	-7.813293297
7	-1.077437148	0.449357116	-9.010878637
6	-1.770335155	0.873701690	-10.119429582
1	-2.536120231	0.254068242	-10.573622594
16	-9.307905700	-2.069029602	-1.537615896
6	-8.066007904	-1.016857196	-2.421510070
8	-7.249010842	-0.543846191	-1.678989054
6	-7.995242724	-0.963915171	-3.932703273
6	-7.529303447	-2.313213617	-4.531006739
6	-6.189736290	-2.864254624	-4.014515166
6	-4.904636019	-2.203290469	-4.542965632
6	-4.716394030	-0.742412047	-4.159309645
1	-8.976230778	-0.686734557	-4.359360320
1	-7.296418879	-0.153483606	-4.173198933
1	-8.307094708	-3.071191578	-4.334335750
1	-7.490353853	-2.187402033	-5.629115064
1	-6.157187397	-2.854029564	-2.908923772
1	-6.152000528	-3.932834419	-4.301256981
1	-3.860735082	-3.854663176	-4.181433514
1	-4.939430630	-0.564767419	-3.093880269
1	-5.344858149	-0.088601533	-4.781599986
1	-3.674292369	-0.453261174	-4.363277364
6	1.789898478	-1.977593187	-6.029266078
6	0.477172297	-1.598186773	-6.684243412
8	-0.195903106	-0.600967763	-6.328280080
8	0.004320304	-2.343540639	-7.616125632
1	1.703135587	-3.003857943	-5.636763041
1	2.007027475	-1.308243669	-5.183412076
26	-1.706364171	-1.214501406	-7.932695386

8	-3.775395419	-2.909949735	-3.971373990
17	-3.727656217	-0.243872457	-7.465944796
1	-4.264789837	-4.902403436	-10.783056175
1	-1.794423509	2.796641472	-11.212971350
1	-10.431069864	-2.011391986	-2.337320705
1	-4.911538427	-2.244969644	-5.632131608
1	2.620873687	-1.986499637	-6.734580329

^{3}PrOH$_{\text{model 4, A}}$

7	-2.182967793	-4.548890898	-9.869851504
1	-1.718854881	-5.401593761	-10.193289999
6	-3.475730725	-4.221116875	-10.212000558
6	-1.663527527	-3.569475173	-9.112147700
1	-0.656582509	-3.576114966	-8.703331424
7	-2.558163294	-2.599810888	-8.922050150
6	-3.696466574	-2.997482325	-9.607198022
1	-4.602331392	-2.396959828	-9.606044363
7	-0.575687897	2.381446997	-9.610865427
1	-0.041422164	3.243214454	-9.533377717
6	-1.502935408	2.068161968	-10.577733185
6	-0.559948726	1.384241756	-8.709043623
1	0.083391762	1.384328715	-7.837150497
7	-1.433701233	0.441657206	-9.033686880
6	-2.017370811	0.848653371	-10.205317505
1	-2.768744967	0.230734441	-10.681912712
16	-8.988278223	-2.023996660	-1.289050796
6	-7.471447371	-1.807491531	-2.358475015
8	-6.674510726	-1.030974491	-1.917953408
6	-7.230292737	-2.734600705	-3.525168062
6	-5.953853192	-2.422405945	-4.312783038
6	-6.068848695	-1.174262890	-5.206897804
6	-5.244129562	-1.175382941	-6.501535193
6	-5.420214461	0.118645921	-7.277777812
1	-7.180760873	-3.749457579	-3.088078364
1	-8.117496332	-2.763500281	-4.184707972
1	-5.105431226	-2.321041176	-3.616239556
1	-5.744791841	-3.317292908	-4.917505292
1	-7.120909048	-1.044641706	-5.526874670
1	-5.819534634	-0.269616533	-4.622692037
1	-3.508562580	-0.540337888	-5.727988807
1	-4.816915217	0.076750212	-8.200091851
1	-5.081933734	0.986366897	-6.691368509
1	-6.475099837	0.268878617	-7.564308512
6	1.682975881	-2.684211749	-6.225107281
6	0.457878838	-1.768667034	-6.286805093
8	0.212039466	-1.035299039	-5.312141316
8	-0.268376803	-1.811149913	-7.345425789
1	1.460152782	-3.671289892	-6.666253513
1	1.934419547	-2.811883867	-5.163383331
26	-2.130933476	-0.973911203	-7.658338816
8	-3.804845667	-1.360662787	-6.210769340
17	-2.236099406	0.970614905	-6.120483324
1	-4.154649419	-4.972664988	-10.614880401
1	-1.862473073	2.728676057	-11.366733262
1	-10.040188289	-2.084649464	-2.180204016
1	-5.582730276	-1.987781727	-7.144516603
1	2.537281939	-2.273826881	-6.763449874

^{5}PrOH$_{\text{model 4, A}}$

7	-2.173683886	-4.529999689	-10.258232896
1	-1.764397093	-5.390031159	-10.637560457
6	-3.515487573	-4.249644953	-10.327546569
6	-1.563155906	-3.575982556	-9.550662159
1	-0.504195410	-3.587492208	-9.324155204
7	-2.440106083	-2.664185877	-9.134006827
6	-3.669218740	-3.070968741	-9.623777140
1	-4.575863788	-2.508878530	-9.412860027
7	-0.505022154	2.629107177	-9.351106413
1	-0.161676789	3.582092627	-9.225613346
6	-1.403286003	2.232400827	-10.317172944
6	-0.364221577	1.614042416	-8.471879546
1	0.287083806	1.661345925	-7.601045012
7	-1.120112536	0.583393919	-8.823205343
6	-1.778072102	0.959004121	-9.970633612
1	-2.499769807	0.301967472	-10.447171510
16	-9.598566152	-2.468944322	-0.558570764
6	-8.163672608	-1.681483172	-1.455578135
8	-7.302152631	-1.228802998	-0.756651202

6	-8.085630442	-1.777959916	-2.957368800
6	-7.137967330	-0.718341769	-3.520209353
6	-7.323416809	-0.461740895	-5.015215067
6	-6.861006463	-1.620107719	-5.903008816
6	-6.899176547	-1.232062545	-7.377601538
1	-7.754632269	-2.812404138	-3.183040169
1	-9.092629838	-1.690749124	-3.403136793
1	-7.313942727	0.215492025	-2.963171377
1	-6.092784652	-1.010959748	-3.329791672
1	-8.375824329	-0.198638582	-5.237311838
1	-6.719733007	0.419976507	-5.294462224
1	-4.896764889	-1.308238731	-5.848871194
1	-6.249915189	-0.357542037	-7.557046533
1	-7.924229084	-0.983906731	-7.705857975
1	-6.535304121	-2.070047305	-7.995977058
6	1.764139722	-2.384902826	-5.973259207
6	0.451745991	-1.863023254	-6.514480498
8	-0.177212174	-0.929340439	-5.950220692
8	-0.055666637	-2.398386728	-7.554832117
1	1.716035377	-3.485534257	-5.906058745
1	1.949278087	-1.975897295	-4.968322300
26	-1.770375356	-1.129864802	-7.746016125
8	-5.517848970	-1.994642489	-5.522199225
17	-3.386797556	0.057948777	-6.547295425
1	-4.211105678	-5.008916164	-10.684883942
1	-1.799572229	2.836513813	-11.133305314
1	-10.675823877	-1.842743536	-1.151644102
1	-7.530244536	-2.470056685	-5.769687868
1	2.592099854	-2.134252730	-6.636375986

$^{5}Re_{model\ 4,\ AB}$

6	2.885630241	-6.374622608	-11.467548142
1	3.768179807	-5.833253576	-11.072284668
6	2.375677038	-5.609970358	-12.701318337
1	2.203926868	-4.555324995	-12.441392325
1	1.411152641	-6.010050769	-13.069769622
1	3.084208385	-5.610230395	-13.549857305
6	1.852682612	-6.445903382	-10.326474557
1	0.955242369	-7.011934767	-10.647151223
1	2.276809567	-7.005594404	-9.477199339
6	1.415409137	-5.080228926	-9.792454902
1	0.946942753	-4.457938998	-10.576133096
1	2.277894211	-4.508828402	-9.398110915
1	0.694066763	-5.199037532	-8.964542427
6	-4.554613506	-5.156003997	-10.855751264
1	-5.533006067	-4.785300263	-10.523525204
1	-4.339279091	-6.089633978	-10.310572362
7	-2.209882779	-4.240363009	-10.462599202
1	-1.761786132	-5.091722444	-10.818475104
6	-3.580330916	-4.080482375	-10.432294978
6	-1.620489673	-3.237932233	-9.799067215
1	-0.552023122	-3.171773446	-9.634823916
7	-2.536520961	-2.392503123	-9.332106203
6	-3.764511206	-2.902083976	-9.729262523
1	-4.697073863	-2.407346021	-9.472019318
6	-5.742358133	-6.010275891	-6.936987621
1	-5.743602292	-6.209854344	-5.849161300
6	-4.772100720	-7.019748837	-7.565720108
1	-5.024346343	-8.048435197	-7.260778120
1	-4.775090502	-7.010322375	-8.666667056
1	-3.735955685	-6.819719403	-7.244607770
6	-5.306128196	-4.559511835	-7.155451643
1	-5.358130962	-4.249897043	-8.209400799
1	-4.248225128	-4.440752215	-6.865373884
1	-5.913252137	-3.841979136	-6.573826446
6	-4.878900828	8.936185109	-9.242097381
1	-5.311328436	8.173448687	-9.906084916
1	-4.303316761	9.637326433	-9.871162382
6	-3.964175788	8.314897686	-8.211109337
6	-4.208170813	7.030410208	-7.702474564
1	-5.070522482	6.465618346	-8.067237288
6	-3.379441140	6.474731836	-6.720750731
1	-3.567120541	5.456379397	-6.366875877
6	-2.305183257	7.218864149	-6.219281029
1	-1.682889741	6.803474286	-5.425860724
6	-2.848323478	9.023056593	-7.734461492
1	-2.627626185	10.019161241	-8.133385043
6	-2.039495939	8.492066171	-6.732003384
1	-1.207523066	9.090418348	-6.355390206
6	-1.960675623	2.933523730	-11.377546232
1	-2.794561668	3.576128889	-11.038548782

1	-2.371526918	2.227573402	-12.113301282
7	-0.351952513	2.222352254	-9.453658773
1	0.389126992	2.903844268	-9.593302764
6	-1.489190463	2.086395173	-10.222763028
6	-0.327066005	1.248368962	-8.521921732
1	0.477780803	1.132219678	-7.799257537
7	-1.405717636	0.484431074	-8.640486504
6	-2.139573861	1.000349988	-9.682009022
1	-3.100292453	0.573142454	-9.943383815
6	-6.224016853	4.601203382	-0.678823622
1	-6.160378415	5.210938771	-1.600000497
1	-5.218589319	4.629809011	-0.220868540
6	-6.628923556	3.156739176	-1.039057483
1	-6.414776662	2.466810251	-0.204035332
1	-7.710559593	3.080237328	-1.230247853
7	-5.922333358	2.661199412	-2.224953150
1	-4.905493313	2.510701413	-2.126671245
6	-6.437884759	2.542198810	-3.466293928
7	-7.752237911	2.415117201	-3.665707866
1	-8.409158532	2.236837095	-2.902646391
1	-8.184665197	2.558930823	-4.603520787
7	-5.600642254	2.516229093	-4.515329631
1	-4.653183675	2.912340738	-4.431036082
1	-5.950479254	2.372252780	-5.474660952
16	-9.081759114	-2.188431585	-1.091433654
6	-7.514471075	-2.127111097	-2.096093669
8	-6.634112975	-1.529487787	-1.545098330
6	-7.344451330	-2.870250593	-3.400285057
6	-7.127389490	-1.917452478	-4.589090340
6	-8.351792040	-1.062587207	-4.926046631
6	-8.186391759	-0.144350097	-6.137677542
6	-7.839803712	-0.855030047	-7.443035559
1	-6.431776810	-3.476342332	-3.257833468
1	-8.182383406	-3.557004407	-3.592396904
1	-6.250130385	-1.273622184	-4.390400714
1	-6.866810431	-2.546184362	-5.456138907
1	-9.217496164	-1.732855842	-5.101840689
1	-8.619222490	-0.433944141	-4.053704858
1	-9.128180818	0.416639346	-6.261562772
1	-8.513119525	-1.712304932	-7.623561785
1	-6.802050164	-1.236147163	-7.452110552
1	-7.948219965	-0.170211466	-8.303093821
6	1.716080310	-2.100453317	-6.149435199
6	0.438091483	-1.607054085	-6.792054916
8	-0.339657259	-0.826513097	-6.159928371
8	0.048143699	-2.017565712	-7.924458054
1	1.561887331	-3.165544405	-5.894646995
1	1.897322639	-1.555484058	-5.210304483
26	-1.971585252	-1.214181471	-7.580413672
8	-2.389030927	-2.480772053	-6.627938768
17	-3.596608043	0.145301706	-6.629057692
8	-1.714697914	0.825494643	-3.742818643
1	-1.547142600	-0.134199065	-3.632217042
1	-2.232704691	0.872851932	-4.569677732
8	-1.573387167	-5.183204623	-7.203602269
1	-1.698507969	-4.214351662	-7.169808566
1	-1.929995873	-5.505834440	-6.340829767
8	-3.981848461	-3.854606601	-4.111706410
1	-3.323491542	-3.165743757	-3.854164341
1	-4.383509731	-3.531524793	-4.935755954
8	-1.806226289	-2.154470566	-3.925568365
1	-1.106293094	-2.855050570	-3.846018853
1	-1.947044800	-2.057736070	-4.898371778
8	-3.413412177	3.390233261	-7.788629367
1	-3.466466540	2.458622371	-7.499503455
1	-4.253580651	3.590342738	-8.268263259
1	3.331874541	-7.334530389	-11.727375764
1	-4.643885909	-5.439371598	-11.904465026
1	-6.749773696	-6.187318622	-7.313600243
1	-5.675315435	9.549543727	-8.820692233
1	-1.306969711	3.614347102	-11.922720916
1	-6.919337279	5.104227326	-0.006838695
1	-10.113926715	-2.195830668	-2.007361005
1	-7.420224929	0.599393752	-5.918815033
1	2.586498079	-2.041936468	-6.802899811

^{5}TS$_{model\ 4,\ AB}$

6	2.888655738	-6.380288345	-11.458183483
1	3.772323661	-5.835695268	-11.069972835

6	2.365691379	-5.614863517	-12.684779902
1	2.202898174	-4.560811066	-12.420607798
1	1.395357753	-6.010827659	-13.042318934
1	3.064716348	-5.615792233	-13.541183820
6	1.869404274	-6.462765784	-10.306178709
1	0.983622341	-7.056912632	-10.607316093
1	2.319776177	-7.001345671	-9.456467810
6	1.396111048	-5.106755666	-9.779815874
1	0.868630759	-4.518009188	-10.551744941
1	2.245659871	-4.490112181	-9.427196148
1	0.711626446	-5.249114395	-8.924802925
6	-4.602264684	-5.173376960	-10.840752180
1	-5.585060688	-4.802146303	-10.525422797
1	-4.412660571	-6.123943916	-10.316948048
7	-2.285573282	-4.299218828	-10.303714654
1	-1.825058924	-5.136160697	-10.676574970
6	-3.649800152	-4.117355012	-10.344672163
6	-1.727783917	-3.331436895	-9.571755656
1	-0.666318677	-3.293383415	-9.374222558
7	-2.650938141	-2.486807663	-9.117604052
6	-3.860290589	-2.965282308	-9.605793496
1	-4.803697946	-2.472944405	-9.382156565
6	-5.725430287	-6.256277514	-6.919071653
1	-5.763457261	-6.616442598	-5.875715954
6	-4.773670164	-7.187596526	-7.668347759
1	-5.079322514	-8.237034122	-7.575895181
1	-4.706262116	-6.976309179	-8.744418117
1	-3.752913715	-7.108073192	-7.255988102
6	-5.201855327	-4.825694717	-6.894737480
1	-5.359267884	-4.281283603	-7.840978041
1	-4.110034686	-4.828263740	-6.734148743
1	-5.672947911	-4.258341997	-6.079579346
6	-4.876521414	8.927993573	-9.235249203
1	-5.309408343	8.160595866	-9.894413319
1	-4.306968142	9.626681694	-9.872449071
6	-3.947408495	8.312346988	-8.212624293
6	-4.185088541	7.034068950	-7.687029965
1	-5.067683573	6.477499454	-8.013107134
6	-3.317549596	6.467269649	-6.746531882
1	-3.499095250	5.451874881	-6.385217287
6	-2.207001362	7.192535258	-6.300814616

1	-1.541884611	6.759288329	-5.551221322
6	-2.807530902	9.011146797	-7.780481709
1	-2.596069197	10.006142734	-8.186899568
6	-1.955187653	8.466358021	-6.821883060
1	-1.095874244	9.050764520	-6.488310128
6	-1.938009728	2.865561675	-11.416020185
1	-2.767911857	3.487396887	-11.029074144
1	-2.361732699	2.171509026	-12.156271178
7	-0.491877778	2.329277740	-9.339270893
1	-0.028094629	3.231319417	-9.246980126
6	-1.417898619	1.995117320	-10.306724455
6	-0.408376143	1.318789303	-8.452503954
1	0.244041222	1.341920482	-7.584364583
7	-1.231837464	0.335072272	-8.788847383
6	-1.863985282	0.745469967	-9.942611074
1	-2.629016334	0.136266153	-10.409997914
6	-6.220633410	4.633335750	-0.677490280
1	-6.159929932	5.252634287	-1.593268970
1	-5.214976864	4.664241707	-0.220418797
6	-6.608663455	3.190930350	-1.054746552
1	-6.391140061	2.492144649	-0.228423725
1	-7.687896853	3.099502005	-1.254149329
7	-5.881533263	2.735387702	-2.242547409
1	-4.857272512	2.643235035	-2.139987789
6	-6.375165634	2.682025225	-3.496375887
7	-7.679711708	2.483681041	-3.717665642
1	-8.324408700	2.263931023	-2.958140187
1	-8.117385739	2.636860222	-4.653383878
7	-5.538007909	2.799268032	-4.533201089
1	-4.569816652	3.126514313	-4.404186216
1	-5.860125588	2.677876047	-5.506796537
16	-9.186009578	-2.343742657	-1.095764001
6	-8.294925447	-1.132112350	-2.206466565
8	-7.446281892	-0.510456456	-1.628180312
6	-8.506803001	-1.106616998	-3.705446299
6	-7.723584672	-2.250030034	-4.401519067
6	-6.185573327	-2.039194202	-4.488880553
6	-5.731202500	-1.575115222	-5.874988203
6	-6.178857935	-0.194639679	-6.284930515
1	-9.578370123	-1.195353286	-3.952156590
1	-8.166155000	-0.117336022	-4.050795971

1	-7.945234082	-3.203497246	-3.891347589
1	-8.141480936	-2.355964985	-5.417927060
1	-5.854946901	-1.305923121	-3.729837105
1	-5.680350852	-2.981324977	-4.227677795
1	-4.531487015	-1.639984320	-5.917411682
1	-5.884039404	0.563555043	-5.547379722
1	-7.283269550	-0.170329805	-6.390704739
1	-5.755242086	0.100733308	-7.257027396
6	1.667753669	-2.166091378	-5.969391937
6	0.323436886	-1.765589964	-6.539493167
8	-0.338841268	-0.788405334	-6.084553670
8	-0.229080487	-2.439224479	-7.467041579
1	1.621822285	-3.241996309	-5.729928945
1	1.859005232	-1.610234028	-5.038443175
26	-2.064914058	-1.154457479	-7.415595443
8	-3.095977049	-2.061790018	-6.211583955
17	-3.050397589	0.905888362	-6.503624829
8	-1.662454678	0.919310699	-3.552260005
1	-1.538376340	-0.050739938	-3.465440566
1	-2.074246644	1.026168096	-4.436375295
8	-1.352021243	-5.186676624	-7.189813011
1	-0.937623508	-4.305503480	-7.122326280
1	-1.635572119	-5.448136371	-6.280905428
8	-3.560958097	-4.087832176	-4.082072489
1	-3.083543342	-3.375019531	-3.608056909
1	-3.687596568	-3.705186610	-4.973420521
8	-1.790558377	-1.945579027	-3.733002416
1	-1.057018627	-2.607535780	-3.790789164
1	-2.173320373	-1.862471327	-4.646172277
8	-3.174870376	3.630199352	-8.215399658
1	-3.202962100	2.769032490	-7.747281621
1	-4.080446613	3.819261249	-8.560134902
1	3.334322282	-7.338923444	-11.723640456
1	-4.665319831	-5.435495417	-11.896870205
1	-6.741525331	-6.319490488	-7.308458747
1	-5.672441039	9.543230986	-8.815651550
1	-1.297691657	3.568347338	-11.949079658
1	-6.921342676	5.124919744	-0.002642870
1	-10.426336278	-2.552355463	-1.663582630
1	-6.003999367	-2.285608208	-6.655275911
1	2.503024231	-2.024931362	-6.655295914

^{5}Int$_{\text{model 4, AB}}$

6	2.901835797	-6.382628483	-11.450165882
1	3.793897879	-5.839840785	-11.079024402
6	2.357113109	-5.615878886	-12.665956030
1	2.197636713	-4.562485854	-12.397310029
1	1.381691031	-6.012551533	-13.008528887
1	3.042858719	-5.614806212	-13.533049709
6	1.909016779	-6.464002037	-10.276818924
1	1.004984379	-7.036614451	-10.565682893
1	2.368156165	-7.022919915	-9.445692941
6	1.481989885	-5.107207367	-9.716914696
1	0.960401281	-4.488094714	-10.468149964
1	2.356069016	-4.523904530	-9.367703443
1	0.810175215	-5.246214456	-8.851374406
6	-4.600260949	-5.166567846	-10.865553257
1	-5.574165966	-4.761803904	-10.561733416
1	-4.441208133	-6.115497897	-10.327780936
7	-2.261078784	-4.409598894	-10.272348593
1	-1.839888715	-5.278888977	-10.617288528
6	-3.608248551	-4.140381086	-10.384231904
6	-1.670057738	-3.455732191	-9.552198800
1	-0.616969771	-3.470269944	-9.310064759
7	-2.555892441	-2.536273973	-9.171485070
6	-3.771408428	-2.948605752	-9.700723740
1	-4.687270051	-2.390835104	-9.527382519
6	-5.752000600	-5.951685291	-6.932584110
1	-5.745516051	-6.142041407	-5.843076206
6	-4.739169077	-6.927553491	-7.548301000
1	-4.945855413	-7.965167146	-7.236643832
1	-4.735972699	-6.929219426	-8.649175711
1	-3.713575197	-6.681800688	-7.220010654
6	-5.389890932	-4.481098753	-7.159736815
1	-5.477948820	-4.176172915	-8.213922475
1	-4.335138814	-4.302339053	-6.890574030
1	-6.017435326	-3.794024538	-6.564428862
6	-4.856984534	8.925867423	-9.227726202
1	-5.281665644	8.179692264	-9.914978761

1	-4.267712895	9.637106909	-9.832543280	6	-8.240413189	-1.185174694	-3.165403919
6	-3.963333195	8.275964126	-8.195073595	6	-7.190813172	-2.192033118	-3.730343273
6	-4.195230133	6.965321295	-7.750576644	6	-6.234119810	-1.562332400	-4.753896091
1	-5.028885022	6.395245979	-8.171981278	6	-6.775632895	-1.176155037	-6.092722553
6	-3.383053188	6.382426930	-6.769966168	6	-8.206984311	-0.951776237	-6.427597257
1	-3.562181904	5.347588720	-6.465754540	1	-9.222195141	-1.320170869	-3.644005915
6	-2.331172036	7.118412294	-6.210619085	1	-7.921525756	-0.148144626	-3.358718014
1	-1.713465857	6.674129290	-5.427328947	1	-6.567861142	-2.579796647	-2.906318426
6	-2.871531858	8.979994445	-7.658625085	1	-7.703955038	-3.070150935	-4.160301069
1	-2.654547929	9.994867332	-8.008494280	1	-5.749471831	-0.673971400	-4.285555080
6	-2.076220015	8.417912621	-6.662701702	1	-5.395656334	-2.273452340	-4.887134096
1	-1.262432731	9.014085038	-6.246184141	1	-3.524802940	-1.655526880	-5.865837056
6	-1.924041497	2.895171407	-11.440001831	1	-8.563227128	0.050073699	-6.092298666
1	-2.756041448	3.514265645	-11.052944816	1	-8.874250940	-1.694396679	-5.952832939
1	-2.348323972	2.196313765	-12.174814158	1	-8.373206537	-0.995083145	-7.518482444
7	-0.500367575	2.386266388	-9.344893221	6	1.735162579	-2.144512572	-5.975368550
1	-0.086091079	3.309774770	-9.227433737	6	0.423549210	-1.720099639	-6.592801732
6	-1.395418048	2.030179933	-10.333300414	8	-0.209683363	-0.702418048	-6.204210950
6	-0.407625992	1.374793793	-8.460013699	8	-0.117031348	-2.421247418	-7.514917324
1	0.228512733	1.405307889	-7.580790510	1	1.653595208	-3.205221401	-5.683161122
7	-1.191997540	0.369899279	-8.822651676	1	1.939431125	-1.545819260	-5.074458296
6	-1.809846565	0.765453330	-9.988924037	26	-1.933647805	-1.220071071	-7.548478931
1	-2.547786165	0.138464620	-10.475758993	8	-2.758795556	-2.208915856	-6.112133770
6	-6.221654568	4.618562100	-0.693628128	17	-3.340917857	0.598588963	-6.679428476
1	-6.152193715	5.240015637	-1.606682074	8	-1.666595315	0.861878881	-3.660403938
1	-5.216579970	4.634300445	-0.234455377	1	-1.478049700	-0.098702638	-3.555641813
6	-6.633641473	3.182967222	-1.075609481	1	-2.107312835	0.925450828	-4.531399193
1	-6.438823588	2.480187931	-0.245987610	8	-1.323536859	-5.165389527	-7.096169461
1	-7.713476529	3.116573893	-1.280012285	1	-0.935428248	-4.274633184	-7.017342853
7	-5.915458875	2.701169308	-2.258365158	1	-1.637245060	-5.425978302	-6.198029339
1	-4.895399230	2.570800666	-2.155147479	8	-3.590696366	-4.030876199	-4.128378091
6	-6.428979658	2.484390297	-3.484685108	1	-3.099066973	-3.353772805	-3.620445102
7	-7.742739380	2.317981192	-3.678261426	1	-3.525020765	-3.682838899	-5.044586076
1	-8.407281932	2.248426833	-2.905076283	8	-1.607962825	-1.994814578	-3.702161219
1	-8.171754367	2.407244096	-4.624915042	1	-0.909173346	-2.692521662	-3.646918205
7	-5.590774490	2.388747489	-4.527115128	1	-1.915234236	-2.013653484	-4.657435450
1	-4.645951790	2.793202211	-4.465042425	8	-3.312455439	3.492916592	-8.219725597
1	-5.923671277	2.133903933	-5.468602598	1	-3.293062220	2.614517644	-7.784806850
16	-9.410596080	-2.509375882	-0.793055284	1	-4.241714481	3.637198584	-8.519224214
6	-8.309539962	-1.306115455	-1.662516909	1	3.341142297	-7.342399212	-11.722043187
8	-7.541693243	-0.760904919	-0.913931417	1	-4.665234972	-5.436692363	-11.919535439

1	-6.752000490	-6.167163330	-7.308930693
1	-5.659320396	9.535155038	-8.811681625
1	-1.286273282	3.596080496	-11.978560536
1	-6.917242960	5.113503576	-0.015943057
1	-10.555327725	-2.622980427	-1.555309239
1	-6.033924971	-0.867686860	-6.829461355
1	2.566739315	-2.060545811	-6.675009200

^{5}PrOH$_{\text{model 4, AB}}$

6	2.908239323	-6.376628211	-11.452520408
1	3.804419002	-5.838257386	-11.084656784
6	2.362474479	-5.609113413	-12.667453861
1	2.193747430	-4.557032386	-12.396594338
1	1.390154322	-6.009987669	-13.013187281
1	3.051537416	-5.601344641	-13.532022222
6	1.917279263	-6.446985348	-10.277549691
1	0.993130989	-6.980777229	-10.576859863
1	2.356515509	-7.036941356	-9.457742952
6	1.545183687	-5.085964028	-9.689161053
1	1.076741058	-4.418964448	-10.434388424
1	2.439042029	-4.560134216	-9.300925264
1	0.842977717	-5.213767144	-8.847545052
6	-4.591060933	-5.176935672	-10.872865598
1	-5.563161984	-4.773208441	-10.562147507
1	-4.423112287	-6.122449165	-10.329882585
7	-2.239733525	-4.379558079	-10.390040899
1	-1.823918108	-5.244513480	-10.752115557
6	-3.596260522	-4.141892956	-10.417810841
6	-1.632111432	-3.428433450	-9.681573837
1	-0.566168903	-3.418189520	-9.503215555
7	-2.514518728	-2.540161451	-9.227680746
6	-3.747873318	-2.968347699	-9.701473796
1	-4.665726208	-2.434885527	-9.468024988
6	-5.759141884	-6.072870913	-6.920620132
1	-5.788457631	-6.254791844	-5.830232455
6	-4.807056496	-7.114943982	-7.518044751
1	-5.111094713	-8.135058100	-7.233700040
1	-4.761380599	-7.090673463	-8.618664287
1	-3.778521787	-6.960322142	-7.146777396
6	-5.264272810	-4.650767817	-7.162291270
1	-5.183331477	-4.418606407	-8.233778391
1	-4.251420967	-4.530788549	-6.739015628
1	-5.895995206	-3.883203440	-6.694807584
6	-4.856382870	8.926527752	-9.222897788
1	-5.277375450	8.171014297	-9.902467693
1	-4.269896434	9.633913647	-9.834686585
6	-3.962132259	8.293262555	-8.180717990
6	-4.208055664	6.998916531	-7.699349264
1	-5.052536663	6.430040807	-8.098963597
6	-3.398789385	6.433082756	-6.707052412
1	-3.587159419	5.408457740	-6.374129807
6	-2.337208049	7.172445139	-6.170291323
1	-1.722968557	6.740942574	-5.377455944
6	-2.861306307	8.999793961	-7.666977809
1	-2.636151839	10.003120015	-8.044053075
6	-2.069732455	8.456635648	-6.657800679
1	-1.249737530	9.055576826	-6.257981449
6	-1.866612753	3.099396423	-11.457563155
1	-2.673353582	3.753019492	-11.073379223
1	-2.325400813	2.386846330	-12.156357270
7	-0.388559328	2.579466393	-9.394148277
1	0.001024593	3.509943706	-9.248604753
6	-1.315783640	2.244702547	-10.359669554
6	-0.269046461	1.543078636	-8.537873971
1	0.400642572	1.550839216	-7.680357125
7	-1.064088530	0.550846936	-8.902610850
6	-1.718049648	0.974049910	-10.035351953
1	-2.464822582	0.357871657	-10.524160815
6	-6.201484491	4.634123568	-0.702228322
1	-6.127779287	5.269771956	-1.604998459
1	-5.201347931	4.652951784	-0.232308495
6	-6.588088900	3.200058986	-1.113494138
1	-6.404416277	2.487251733	-0.290088988
1	-7.660333020	3.120644895	-1.349502216
7	-5.826693154	2.748157167	-2.282097609
1	-4.810109161	2.624108850	-2.141625450
6	-6.289972246	2.579547977	-3.534811730
7	-7.593747372	2.424931698	-3.782065148
1	-8.279628594	2.247560173	-3.038796114

1	-7.996624480	2.518663202	-4.738215410
7	-5.417456595	2.532912533	-4.553270396
1	-4.472810059	2.929239272	-4.449712356
1	-5.733207066	2.309792285	-5.509397311
16	-9.401939634	-2.377000685	-0.877468644
6	-8.458108893	-1.157799164	-1.919980088
8	-7.518802120	-0.687132297	-1.338475501
6	-8.791365479	-0.927123398	-3.374224469
6	-8.461144624	-2.149503587	-4.249300252
6	-6.970482858	-2.422873362	-4.426333781
6	-6.208392265	-1.364365848	-5.233796164
6	-6.847296814	-1.032890722	-6.588329835
1	-9.860454263	-0.672392996	-3.480198081
1	-8.224649565	-0.035717051	-3.680703473
1	-8.947236950	-3.048907606	-3.832923008
1	-8.926524100	-1.971130271	-5.232759649
1	-6.467685873	-2.552286918	-3.449035394
1	-6.866944133	-3.389692748	-4.945410449
1	-4.303578217	-1.217212540	-5.765006036
1	-7.769357054	-0.434039604	-6.476738021
1	-7.091065578	-1.946871849	-7.158643265
1	-6.141876637	-0.429185848	-7.184625566
6	1.724311879	-2.385337931	-5.985240759
6	0.405163799	-1.945061074	-6.582748258
8	-0.351451414	-1.113537938	-5.989292674
8	0.005858267	-2.423064692	-7.685506993
1	1.716133593	-3.484945844	-5.883265096
1	1.859434162	-1.943922057	-4.986055654
26	-1.772874507	-1.100122093	-7.814164069
8	-4.897093497	-1.931167207	-5.445589415
17	-3.142717625	0.381480206	-6.493054387
8	-1.650116096	0.859575314	-3.489102547
1	-1.472182399	-0.069737264	-3.240184845
1	-2.089482969	0.808006054	-4.363722820
8	-1.316133156	-5.172260322	-7.313302581
1	-0.852872501	-4.317794362	-7.256383985
1	-1.695708589	-5.368584954	-6.420322218
8	-3.650216096	-3.822755909	-3.998242214
1	-2.960411527	-3.198820695	-3.694885223
1	-4.214390969	-3.252317700	-4.577811049
8	-1.361396856	-2.044580581	-3.589965149
1	-0.765618507	-2.827697165	-3.486849242
1	-1.147573025	-1.701647540	-4.491624771
8	-3.277864419	3.455611887	-7.991084666
1	-3.280671429	2.548365667	-7.624971402
1	-4.167903875	3.611280694	-8.387242265
1	3.342739031	-7.338822916	-11.723553009
1	-4.662120478	-5.446275017	-11.926655984
1	-6.765751314	-6.222150196	-7.311158821
1	-5.660134556	9.536217472	-8.810188451
1	-1.225988786	3.757442514	-12.044626696
1	-6.905503639	5.116900037	-0.024473765
1	-10.633647858	-2.535348494	-1.479272972
1	-6.157055203	-0.439842266	-4.658741412
1	2.562159186	-2.120739374	-6.630273170

^{5}PrCl$_{model\ 4,\ AB}$

6	2.911481799	-6.373297956	-11.452966854
1	3.812896157	-5.834987102	-11.097860143
6	2.352521464	-5.607931004	-12.663638445
1	2.182034880	-4.556050682	-12.392112355
1	1.379534777	-6.014172345	-13.002126233
1	3.034580518	-5.599357205	-13.533692945
6	1.937404434	-6.435964777	-10.263427427
1	0.995494078	-6.942281669	-10.555833349
1	2.373245109	-7.048832360	-9.459199290
6	1.616049643	-5.074221500	-9.646610445
1	1.148705930	-4.380659477	-10.367692977
1	2.536407807	-4.584164563	-9.274342432
1	0.935653296	-5.193614518	-8.785609861
6	-4.578688848	-5.203717724	-10.978860838
1	-5.523076252	-4.768291395	-10.636334215
1	-4.438774341	-6.170676834	-10.466939350
7	-2.216914100	-4.509859229	-10.375759003
1	-1.810443430	-5.385489574	-10.715817988
6	-3.558981710	-4.219904583	-10.496791932
6	-1.631618594	-3.573022322	-9.621514960
1	-0.584140138	-3.594281261	-9.351771832
7	-2.513939951	-2.656595480	-9.244394566
6	-3.719606330	-3.042376723	-9.796038585
1	-4.635155029	-2.486171455	-9.615048224

6	-5.663122074	-6.077536600	-6.902717663
1	-5.674284057	-6.346608758	-5.830988739
6	-4.729494532	-7.068746099	-7.608078476
1	-5.043211480	-8.111775687	-7.441987481
1	-4.681740159	-6.921925930	-8.698489384
1	-3.698418068	-6.973467894	-7.225009056
6	-5.164180653	-4.637371991	-7.031116982
1	-5.270347035	-4.224818385	-8.047385871
1	-4.084041600	-4.601172198	-6.807840299
1	-5.679401191	-3.960260892	-6.333116157
6	-4.948440185	8.899934933	-9.214537544
1	-5.404652051	8.133470335	-9.858642979
1	-4.370750252	9.577448291	-9.867456887
6	-4.021384029	8.273691236	-8.195416740
6	-4.266455078	6.992913544	-7.677514443
1	-5.151121622	6.441170754	-8.009642526
6	-3.403221974	6.417836884	-6.736567760
1	-3.595488495	5.405078113	-6.370453549
6	-2.286243908	7.133242010	-6.287212406
1	-1.623172327	6.692607572	-5.539607474
6	-2.874099109	8.960682799	-7.761501381
1	-2.652761168	9.954339885	-8.165957511
6	-2.024046990	8.406245544	-6.805782853
1	-1.156652585	8.980438964	-6.473245330
6	-2.008887772	2.882949757	-11.471524777
1	-2.856816725	3.518450162	-11.150926593
1	-2.395858276	2.174034191	-12.217683082
7	-0.596640321	2.332578028	-9.373683114
1	-0.034526958	3.180081677	-9.341594256
6	-1.563631393	2.038663739	-10.311778833
6	-0.601405398	1.368623057	-8.431074191
1	0.079674287	1.345953623	-7.588845778
7	-1.525130250	0.457721487	-8.699963068
6	-2.124551181	0.857440243	-9.875253176
1	-2.912397460	0.265119987	-10.328038519
6	-6.191173567	4.637128670	-0.725436766
1	-6.122775863	5.280438598	-1.622718563
1	-5.190644680	4.659226662	-0.256874300
6	-6.567255134	3.202768331	-1.148387609
1	-6.378697231	2.485363549	-0.330821846
1	-7.639044918	3.118818287	-1.383901997
7	-5.806040466	2.756582860	-2.321895532
1	-4.791857859	2.610141645	-2.184230335
6	-6.271570303	2.613785072	-3.577537374
7	-7.577178129	2.483458442	-3.825255849
1	-8.262718116	2.255526201	-3.092979456
1	-7.980223795	2.587666826	-4.779936226
7	-5.401408856	2.562454398	-4.599733180
1	-4.465668574	2.984791924	-4.497119070
1	-5.739132676	2.394435372	-5.561974659
16	-9.467722616	-2.534741846	-0.707242276
6	-8.289159193	-1.338554031	-1.458916233
8	-7.513834453	-0.890924732	-0.655374873
6	-8.173981055	-1.112955171	-2.943924788
6	-7.264586492	-2.199058713	-3.572850477
6	-6.449208120	-1.693701827	-4.774230118
6	-7.179169251	-1.588730841	-6.115090992
6	-8.325623792	-0.596210751	-6.207990482
1	-9.158657527	-1.063208445	-3.433113387
1	-7.706821660	-0.121412682	-3.046000327
1	-6.527692641	-2.524487375	-2.818814448
1	-7.852509427	-3.097344793	-3.835024783
1	-6.008319835	-0.710307186	-4.527621159
1	-5.605655563	-2.391030323	-4.888971230
1	-3.849099893	-1.409330890	-6.100446324
1	-8.053018015	0.397316555	-5.819033768
1	-9.194896115	-0.966420270	-5.633674942
1	-8.648209470	-0.480107833	-7.255974614
6	1.805866750	-2.224565427	-5.979550394
6	0.508282283	-1.730795597	-6.581143179
8	-0.031071272	-0.665294089	-6.205342627
8	-0.056091328	-2.443517715	-7.488810132
1	1.709952675	-3.298845583	-5.752580187
1	2.025035515	-1.679360928	-5.048246237
26	-1.886827700	-1.379084198	-7.640314504
8	-2.938121349	-1.750435931	-6.056598385
17	-5.858253412	-0.983388756	-7.332141365
8	-1.531038176	0.893039631	-3.617755099
1	-1.434033922	-0.093702410	-3.535744193
1	-1.783711247	1.012601531	-4.547493585

8	-1.212608639	-5.116498550	-7.084602430
1	-0.763585112	-4.248868121	-7.037639896
1	-1.556673337	-5.327804788	-6.185507307
8	-3.634329708	-3.820806013	-4.224576932
1	-3.163804121	-3.177402584	-3.656598487
1	-3.571736570	-3.372748212	-5.100093192
8	-1.745526912	-1.785545190	-3.758441061
1	-1.041709270	-2.475709943	-3.811965070
1	-2.166805508	-1.708350597	-4.692264362
8	-3.386976624	3.469334533	-8.064725850
1	-3.335785959	2.500679034	-8.131461917
1	-4.255948041	3.715597008	-8.470814122
1	3.343196081	-7.336611548	-11.724475646
1	-4.665226913	-5.438267295	-12.039787312
1	-6.677178383	-6.197633626	-7.283944399
1	-5.727070345	9.538644808	-8.797581793
1	-1.346920646	3.578141853	-11.987833594
1	-6.895161118	5.112388488	-0.042357744
1	-10.580203840	-2.576038166	-1.522734612
1	-7.530739444	-2.571970926	-6.427668431
1	2.631246302	-2.114106296	-6.682838886

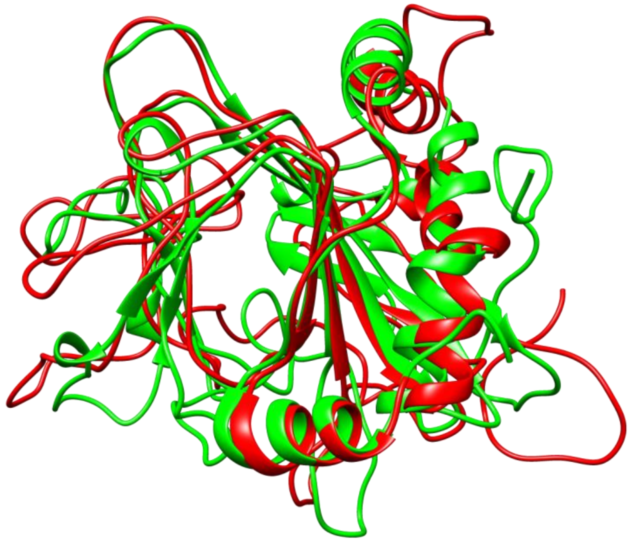

Supplementary Figure 4. Structural overlay of SyrB2 in green and HctB in red.

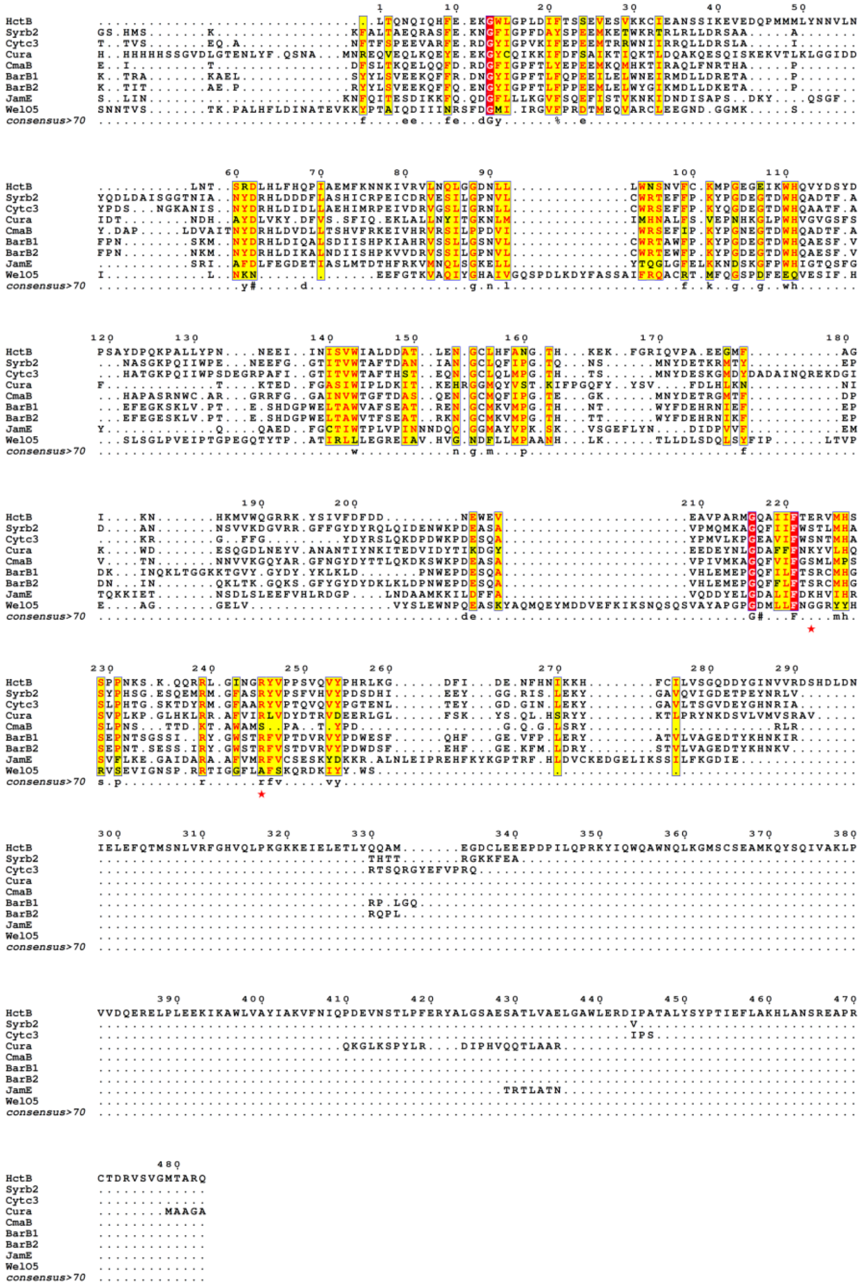

Supplementary Figure 5. Sequence comparison between HctB and known nonheme iron halogenases. Residues Arg_{254} and Glu_{223} are highlighted with a red star.
